# Carbodiimide Ring-Opening Metathesis Polymerization

**DOI:** 10.1021/acscentsci.3c00032

**Published:** 2023-05-11

**Authors:** J. Drake Johnson, Samuel W. Kaplan, Jozsef Toth, Zian Wang, Mitchell Maw, Sergei S. Sheiko, Aleksandr V. Zhukhovitskiy

**Affiliations:** † Department of Chemistry, University of North Carolina at Chapel Hill; Chapel Hill, North Carolina 27599, United States

## Abstract

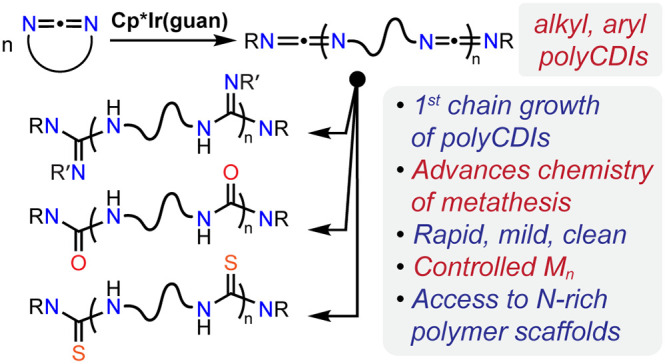

Controlled incorporation of nitrogen into macromolecular skeletons is a long-standing challenge whose resolution would enable the preparation of soft materials with the scalability of man-made plastics and functionality of Nature’s proteins. Nylons and polyurethanes notwithstanding, nitrogen-rich polymer backbones remain scarce, and their synthesis typically lacks precision. Here we report a strategy that begins to address this limitation founded on a mechanistic discovery: ring-opening metathesis polymerization (ROMP) of carbodiimides followed by carbodiimide derivatization. An iridium guanidinate complex was found to initiate and catalyze ROMP of *N*-aryl and *N*-alkyl cyclic carbodiimides. Nucleophilic addition to the resulting polycarbodiimides enabled the preparation of polyureas, polythioureas, and polyguanidinates with varied architectures. This work advances the foundations of metathesis chemistry and opens the door to systematic investigations of structure-folding-property relationships in nitrogen-rich macromolecules.

## Abstract 2

ROMP of aryl and alkyl carbodiimides initiated and catalyzed by an iridium guanidinate produced polymeric carbodiimides that were derivatized to a series of heteroatom-rich polymers.

A remarkable feature of polymers produced in Nature is the abundance of heteroatoms in their backbones. Proteins, whose backbones are one-third nitrogen, are an excellent example of this general fact (Figure 1A). Backbone nitrogen abundance is pivotal for protein folding, which in turn determines its enzymatic, signaling, and structural functions.^1,2^ In contrast, the backbones of synthetic polymers are, by and large, composed of carbon and have disordered 3D structures. Nitrogen-rich backbones are precedented among synthetic polymers but are relatively scarce: some key examples are polyamides,^3^ polyurethanes,^4^ polyethylenimine,^5^ polycarbodiimides,^6−8^ and polyphosphazenes^9^ (Figure 1B). Furthermore, most of them remain challenging to prepare with high structural precision. New strategies toward the synthesis of nitrogen-rich polymer backbones that could lend themselves to greater precision would unlock untapped functional potential of synthetic polymers. Here we demonstrate such a strategy founded on the discovery of carbodiimide ring-opening metathesis polymerization (CDI ROMP).

**Figure 1:**
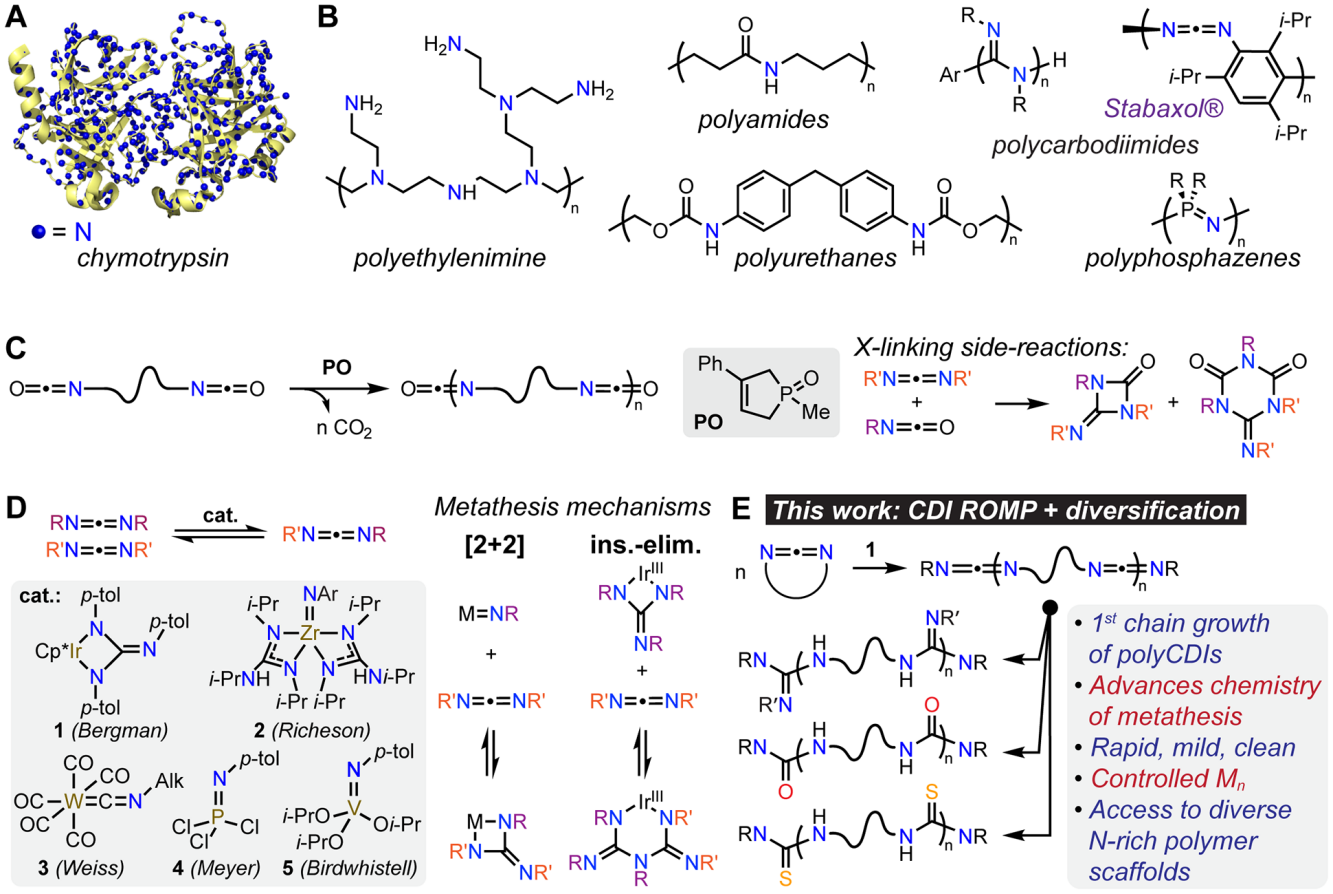
A. Ribbon diagram of chymotrypsin^14^ with nitrogen atoms highlighted. B. Examples of synthetic polymers with nitrogen-rich backbones utilized in industry and academia.^3−5,9,10^ C. State-of-the-art synthesis of polyCDIs and associated side-reactions.^10,15^ D. Cross-metathesis precedents and two proposed mechanisms involved.^16−20^ E. Summary of the present work.

Carbodiimides (CDIs) stand out as some of the most versatile nitrogen-containing functional groups. A notable feature of CDIs is that through nucleophilic addition, they can be converted quantitatively into a broad range of other nitrogen-containing functional groups: for example, ureas, thioureas, guanidines, and a long list of heterocycles.^10−12^ This feature would be particularly valuable in a macromolecular context: numerous classes of nitrogen-rich polymer backbones could be derived in one step from main-chain poly(carbodiimide)s (polyCDIs). Indeed, existing polyCDIs have already demonstrated industrial utility as antihydrolysis stabilizers in foams and cross-linkers for fiber reinforcement and insulating coatings.^10,13^ Yet, the full potential of polyCDIs and their derivatives remains unrealized due to the challenges posed by their synthesis.

PolyCDIs are currently synthesized via decarboxylative step-growth polycondensation of diisocyanates (Figure 1C).^10,15^ This method has been state-of-the-art since 1962 despite the fact that it suffers from cross-linking side-reactions, as well as the limitations inherent in step-growth methods: high molecular weight dispersity (*Đ* ∼ 2), difficulty to attain high-molecular weight, and poor control over polymer architecture. Chain growth methods for polyCDI synthesis are unknown at present. Note that although chain growth coordination/insertion polymerization of carbodiimides has been thoroughly investigated by the Novak group, the resulting polymers no longer have carbodiimide functional groups and are more akin to poly(guanidine)s.^6−8^

Given the unsaturated nature of CDIs and inspired by the far-reaching impacts of ROMP of cyclic alkenes^21,22^ and allenes,^23,24^ we envisioned that CDI ROMP could, likewise, prove highly impactful: it could grant entry to entirely new compositions and architectures of polyCDIs with previously inaccessible levels of precision and molecular weights. This precision could in principle, also be inherited by the derivatives of polyCDIs obtained through postpolymerization modification. Catalytic CDI metathesis has a handful of precedents in literature, though these examples are exclusively of the cross-metathesis variety (Figure 1D): transition metal (TM) imido, iminocarbene, and guanidinate complexes as well as iminophosphoranes have been established as capable catalysts in this context.^16−20^ Notably, these examples present a mechanistic dichotomy of the metathesis process: the imido/iminocarbene complexes and iminophosphoranes mediate [2 + 2] cycloaddition/elimination, while the iridium guanidinate complex **1** is believed to undergo insertion/elimination with carbodiimides.^16−20^ Incidentally, the former generally requires temperatures ≥100 °C to proceed, while the latter is rapid even at ∼20 °C.^16−20^ We envisioned that the high cross-metathesis activity of **1** under mild conditions would enable it to be coopted for ROMP of cyclic CDIs (Figure 1E).^16^

Two known cyclic CDIs—**M1**^25^ and **M2**(26,27) (Figure 2A)—representative of *N*,*N*-diaryl and *N*,*N*-dialkyl CDIs were selected as monomers. On the basis of computations of homodesmotic reactions (Figure S1 of the Supporting Information, SI), we predicted both of these CDIs to have moderate ring-strain energies—9.5 and 8.1 kcal/mol for **M1** and **M2**, respectively—and sufficient overall free energies of ring-opening (−2.3 and −1.9 kcal/mol, respectively) to drive ROMP.^28^ On the basis of ^1^H nuclear magnetic resonance (NMR) spectroscopy, both were found to polymerize rapidly and nearly quantitatively at 23 °C and [monomer] = 0.5 M in the presence of **1** in a range of organic solvents, including ethers, haloalkanes, and aromatics (Figures 2B, S2, and S3). The polymerization of both **M1** and **M2** follows first-order kinetics characteristic of chain-growth (Figure 2C), with deviation at the very end of the polymerization likely due to onset of ring–chain equilibrium (Figure 2C).^29−31^ For **M1**, the number-average molecular weight (*M*_n_) increases linearly with monomer conversion throughout most of the polymerization (Figures 2D and S4), and the degrees of polymerization (DPs) are in close agreement with the theoretical values based on monomer-to-initiator ratios and observed conversions (Figures 2E, S6, and S7; Table S1). Meanwhile, *Đ* increases from ∼1.2 to 2 with conversion (Figures 2D and S4), which suggests that chain transfer is operative in the polymerization of **M1**. Some chain transfer was anticipated due to the presence of linear CDI units in the growing polymer chain in addition to di-*p*-tolyl CDI (**L1**) generated at the initiation stage (vide infra) from **1** (Figure S10). To validate this hypothesis, **polyM1** was prepared and treated in situ with additional **L1** (five equivalents relative to **1**) before termination, which resulted in an ∼80% reduction in *M*_n_ over 16 h at 23 °C (Figure S11). Nonetheless, though the polymerization of **M1** is not living due to chain transfer, it exhibits good molecular weight control, matching closely with theoretical values up to experimental DPs of at least 750. Notably, though **L1** generated during initiation could lead to eventual halving of the polymer *M*_n_, crude ^1^H NMR spectra of the polymerization of **M1** reveal that **L1** is only partially consumed during the time scale of polymerization, which explains the close agreement of the theoretical and experimental DPs (Figure S12).

**Figure 2:**
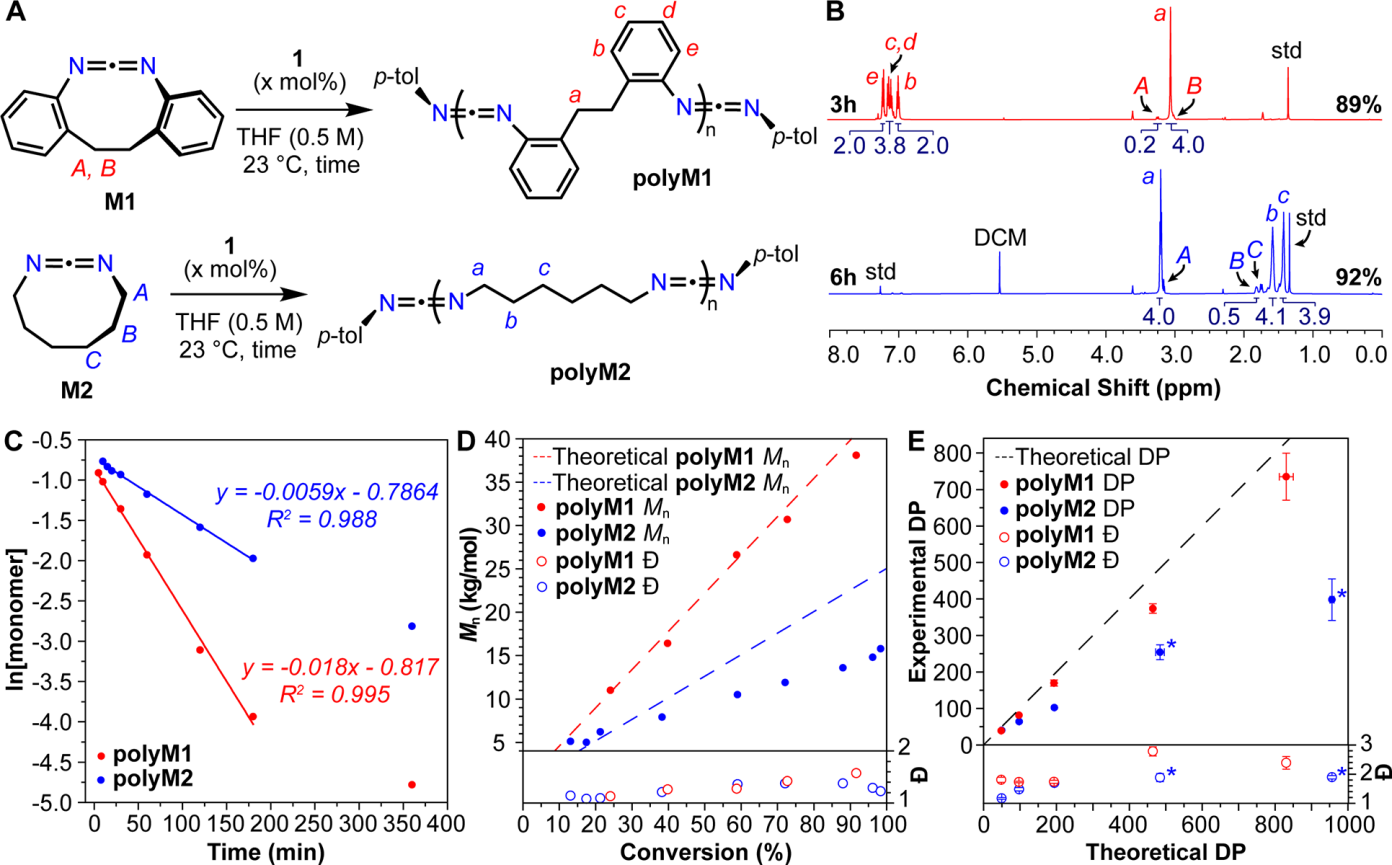
A. Polymerization of **M1** and **M2**, with key hydrogen atoms labeled. B. ^1^H NMR spectra of crude polymerization reaction mixtures for **M1** and **M2** at 3 and 6 h, respectively, in perdeuterated tetrahydrofuran (THF-*d*_8_); key resonances are assigned, and % monomer conversions are indicated at the bottom right side of the spectra; std = 1,3,5-tri*tert*-butylbenzene. C. Semilogarithmic plot of monomer concentration versus time in the polymerization of **M1** and **M2** ([monomer]:[**1**] = 200:1, [monomer] = 0.5 M, temperature = 23 °C, Figures S2 and S3). D. Evolution of *M*_n_ and *Đ*, measured by gel permeation chromatography with multiangle light scattering (GPC-MALS) as a function of monomer conversion for the polymerization of **M1** and **M2** ([monomer]:[**1**] = 200:1, [monomer] = 0.5 M, temperature = 23 °C, Figures S4 and S5). E. Experimental DP and *Đ*, as measured by GPC-MALS vs theoretical based on variation in [monomer]:[**1**] loadings and near-terminal conversions for the polymerization of **M1** and **M2** (Figures S6–S9, Tables S1 and S2); * = data for high-MW fraction only (Figure S8).

In contrast to **M1**, experimental *M*_n_-s of **M2** deviate negatively from the theoretical ones (Figures 2D and S5). The negative deviation is consistent with chain transfer and/or establishment of ring–chain equilibrium earlier in the polymerization.^29^ The same trend is observed for experimental vs theoretical DPs of **polyM2** obtained by varying the [**M2**]:[**1**] ratio and taking monomer conversion into account (Figures 2E, S8, and S9; Table S2). In addition to previous experiments displaying clear chain transfer with **L1** in **M1** polymerization, this hypothesis is also supported by the growth of characteristic *p*-tolyl-CDI polymer end-group resonances and the disappearance of **L1** ones in the crude ^1^H NMR spectra during the first 2 h (∼71% conversion) of the polymerization of **M2** (Figures S13–S15). Furthermore, for [**M2**]:[**1**] = 500:1 and 1000:1, low-molecular weight species are observed in the differential refractive index (dRI) GPC trace of crude **polyM2**, integrating to 19 and 25% of the sample, respectively (Figure S8). Nonetheless, despite the extensive chain transfer/backbiting, the DP of **polyM2** could be controlled by tuning the [**M2**]:[**1**] up to 500:1, and DPs of up to ∼400 can be obtained experimentally (Figure 2E).

## CDI ROMP Mechanism

**Figure 3:**
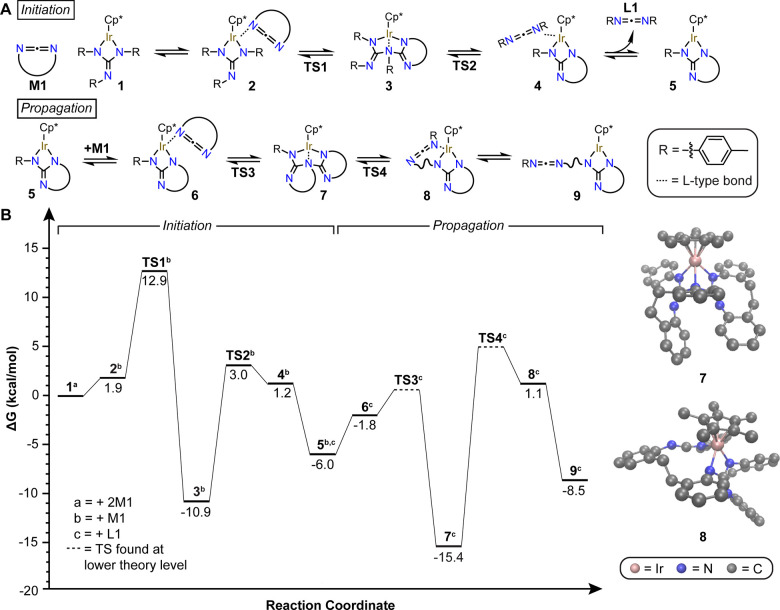
A. Proposed mechanism for CDI ROMP. B. Reaction coordinate diagram for **M1** based on DFT computations using a PBE-D2 functional with a split basis set: 6-311+G(d,p) for nitrogen, 6-311G(d,p) for carbon and hydrogen, and LANL2DZ with included ECP augmented with one f-polarization function (0.938) for Ir and CPCM solvation model for THF.^42^ Computed structures of key intermediates **7** and **8** are shown on the right, with hydrogen atoms omitted for the sake of clarity.

At the outset, we hypothesized that CDI ROMP proceeds via an insertion-elimination mechanism analogous to the one described by Bergman and Holland (Figures 1D, 3A, and 3B).^16^ In our proposed mechanism, initiation begins with the insertion of a CDI monomer into the iridium–nitrogen bond of **1**, converting the four-membered iridocycle into a six-membered one. Next, **L1** is eliminated from the six-membered iridocycle to form a four-membered one, which now has the monomer incorporated; at this point, initiation is complete. Another round of insertion and elimination leads to the ring-opening of the first inserted monomer. Propagation proceeds through further monomer insertion and elimination/ring-opening, which we believed at the outset would be driven by the release of ring strain energy. All the steps described above are expected to be reversible. Insertion of linear CDIs in growing polymers via backbiting or intermolecular cross-metathesis leads to chain transfer (Figure S10). Our kinetic studies described above are consistent with this mechanism.

We turned to theoretical analysis to gain further insights into the insertion and elimination processes and catalyst selectivity for cyclic versus linear CDIs. Density functional theory (DFT)^32^ was utilized to model the metathesis mechanism in both the case of **M1** (Figures 3A and 3B) and a linear analogue di-*o*-tolyl CDI (**L2**, Figures S16) with complex **1**. Specifically, we used the PBE-D2 functional^33−35^ with a split basis set: 6-311+G(d,p) for N,^36,37^ 6-311G(d,p) for C and H,^37^ and LANL2DZ with included effective core potential (ECP)^38^ augmented with one *f*-polarization function (0.938) for Ir^39^ and CPCM solvation model for THF.^40,41^

These computations shed light on the mechanism of insertion: the transition state (TS) for a concerted mechanism could not be located; meanwhile, initial coordination of the CDI to Ir allowed us to locate the insertion TS (**TS1**) with an overall activation barrier of 12.9 kcal/mol. Notably, insertion is exergonic for **M1** (Δ*G* = −10.9 kcal/mol; Figures 3A and 3B) but endergonic for **L2** (Δ*G* = 2.2 kcal/mol, Figure S16). This contrasting behavior suggests that the ring strain present in **M1** is largely alleviated upon insertion due to the change in the N–C–N bond angle from 170° to 130°.

Insertion of **M1** was found to form the tetra-coordinate irido-bicyclic intermediate **3**, as opposed to the tricoordinate intermediate 3* (Figure S17) previously postulated by Holland and Bergman; 3* is a viable structure in principle, but it was computed as both a higher-energy intermediate and one that is formed via a higher-barrier insertion (Figure S17).^16^ Completing initiation, elimination of **L1** via **TS2** (Figures 3A and 3B), followed by dissociation of **L1**, was computed to have a relatively low barrier (13.9 kcal/mol) and to be 4.9 kcal/mol “uphill” from **3**, though still overall 6 kcal/mol “downhill” from the start of initiation.

Propagation begins with coordination/insertion of another **M1** into **5**, forming irido-bicylic intermediate **7** through **TS3**. Elimination/ring-opening of **M1** is expected to occur directly from **7** to coordinated intermediate **8** via **TS4**. Both **TS3** and **TS4** are expected to be viable transition states because they have been located and their energies computed at lower levels of theory (see “Computational Methodology” in SI). All in all, during propagation, 2.5 kcal/mol are released, which is remarkably close to the calculated Δ*G* for the homodesmotic reaction of **M1** (−2.3 kcal/mol, Figure S1). The overall weakly exergonic propagation is consistent with experimentally observed establishment of ring–chain equilibria at high monomer conversions.

Lastly, computations support the hypothesis of chain transfer via competitive insertion between linear and cyclic CDIs, with a ΔΔ*G*^‡^ of 5.8 kcal/mol in favor of **L2** (Figures 3A, 3B, and S16). Importantly, while the use of **L2** is informative of chain transfer with monomeric linear CDIs, it does not fully capture the steric bulk of polyCDIs. Therefore, conclusions drawn about chain transfer behavior with polyCDIs—either via backbiting or to other polymer chains (Figure S10B, S10C)—from this model should be viewed in this context.

## Postpolymerization Modification

**Figure 4:**
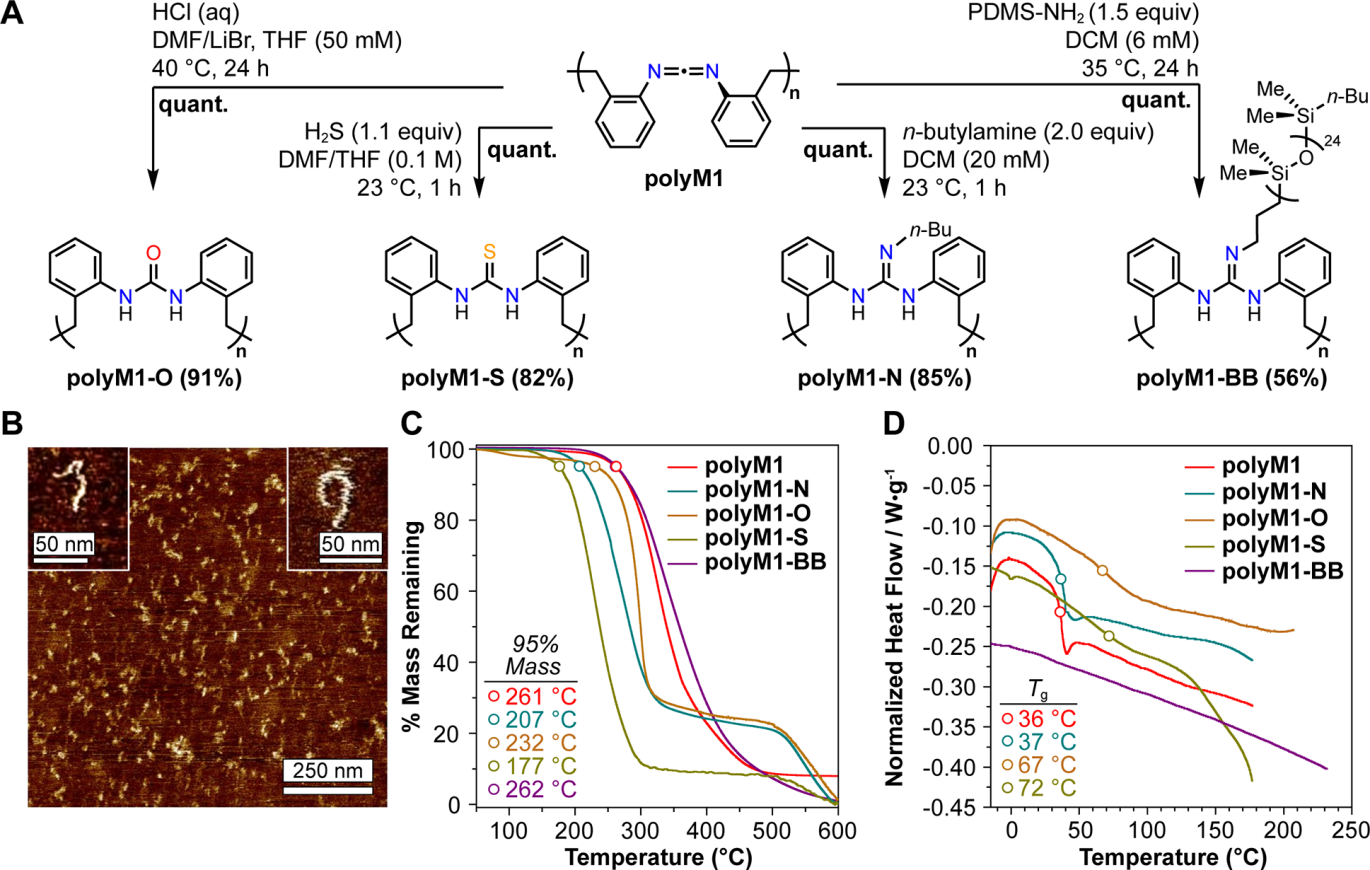
A. Derivatization of **polyM1** with various nucleophiles; conversion is indicated adjacent to reaction conditions, and isolated yields are indicated in parentheses. B. AFM micrograph of spin-casted (from DCM) **polyM1-BB**. Inset: Enhanced images of single **polyM1-BB** bottlebrush in worm-like conformations. C. TGA data (10 °C/min) of **polyM1** derivatives. D. DSC data (10 °C/min, second heating) for **polyM1** derivatives.

Postpolymerization modification of **polyM1** enabled the synthesis of analogous polyguanidines, polythioureas, and polyureas following modified procedures previously reported for other polyCDIs or small molecule CDIs (Figure 4A). In the presence of *n*-butylamine, complete conversion of **polyM1** to the corresponding polyguanidine **polyM1-N** was observed after 7 min at 23 °C (Figure 4A),^43^ judging from changes in the ^1^H and ^13^C NMR and attenuated total reflection Fourier transform infrared (ATR-FTIR) spectra (Figures S18–S20). Quantitative conversion of **polyM1** into bottlebrush polymer **polyM1-BB** was also achieved after a 1-day reaction of **polyM1** at 40 °C with monoamine-terminated polydimethylsiloxane (PDMS) with *M*_n_ = 2 kg/mol (Figures S18, S21, and S22).^43^ The bottlebrush architecture of **polyM1-BB** was confirmed by atomic force microscopy (AFM) (Figure 4B).

**PolyM1** was also quantitatively converted to the corresponding polythiourea **polyM1-S** by treating an *N*,*N*-dimethylformamide (DMF)/THF solution of the former with H_2_S (1 M in THF) for 1 h, as validated by ^1^H and ^13^C NMR and ATR-FTIR spectroscopy (Figures S18, S23, and S24).^44^ Lastly, treatment of **polyM1** in a solution of 25 mM LiBr in DMF with 5 M HCl_(aq)_ furnished polyurea **polyM1-O** after 1 h at 40 °C.^44^ Quantitative conversion in this case was confirmed by solid-state ^13^C NMR and ATR-FTIR spectroscopy (Figures S18 and S25). Attempts to determine the molecular weight of the modified polymers by GPC-MALS proved unsuccessful either due to the interactions of the polymers with the column stationary phase,^45−47^ intramolecular hydrogen-bonding interactions/aggregation,^48^ poor solubility in the mobile phase, or a combination thereof, leading to distorted elution profiles (Figure S26) or inability to collect a spectrum altogether.

## Thermal Properties

Derivatization of **polyM1** led to substantial changes in the materials’ thermal properties as measured by thermogravimetric analysis (TGA) and differential scanning calorimetry (DSC) (Figure 4C and 4D). With the exception of **polyM1-BB**, all derivatives of **polyM1** displayed reduced temperatures at which 5% mass loss was observed, with **polyM1-S** being the least thermally stable of the series (Figure 4C). Interestingly, in contrast to **polyM1-O**, **polyM1-N**, and **polyM1-BB**, both **polyM1** and **polyM1-S** displayed incomplete (∼90%) ultimate mass loss which suggests that these two polymers undergo carbonization at high temperatures. The mechanistic factors responsible for this dichotomy are unclear at present.

DSC revealed a surprising similarity in glass transition temperatures (*T*_g_-s) for **polyM1** and **polyM1-N**: 36 °C vs 37 °C, respectively (Figure 4D). However, **polyM1-S** and **polyM1-O** have broader glass transitions centered at higher temperatures of 72 and 67 °C; further changes in the DSC profile of **polyM1-S** at higher temperatures are believed to be due to polymer degradation, based on TGA (Figure 4C). **PolyM1-BB** does not display any thermal transitions within the accessible temperature range of the instrument, and no melting transitions are observed for any of the derivatives.

In contrast to **polyM1** and its derivatives, **polyM2** retains ≥95% of its mass until 321 °C after which it loses all of the mass in three stages (Figure S27). The corresponding DSC data reveals a small exotherm at 75 °C, and a large exotherm at ∼150 °C, which is attributed to CDI cycloaddition events (Figure S28). Upon a second heating, the exotherm is no longer present, no new thermal transitions are observed at temperatures below that initial exotherm, and a new *T*_g_ is observed at 137 °C.

## Conclusions

In this report, we demonstrate rapid, clean, and efficient CDI ROMP of both *N*,*N*-diaryl and *N*,*N*-dialkyl cyclic CDIs using an iridium guanidinate catalyst. Experimental and computational mechanistic analysis are consistent with an insertion/elimination metathesis mechanism, as well as the presence of competitive chain transfer reactions. Nonetheless, this method can produce polyCDIs with DPs up to 750 in a controlled manner. Furthermore, the *N*,*N*-diaryl polyCDIs can be transformed quantitatively into polyureas and polythioureas, as well as both linear and bottlebrush polyguanidines. Lastly, thermal stability and phase transitions of all of these materials have been measured and found to vary considerably depending on the identity of the nitrogen-containing functional groups. Thus, we lay the foundation for the development of controlled synthesis of a broad array of existing and novel nitrogen-rich polymer backbones, which will enable a systematic evaluation of structure-folding/morphology-property relationships in these valuable classes of materials.

## Supporting Information Available

Materials and methods, experimental procedures, supplementary Figures S1–S28 referenced in the main text, Tables S1 and S2, GPC characterization, spectral characterization, thermal property characterization, and crystallographic data (PDF)
Computational data (PDF)
Crystallographic data for **1** (CIF)

## Supplementary Material

oc3c00032_si_002.pdf

oc3c00032_si_003.cif
