# Correction to “Carbodiimide Ring-Opening Metathesis Polymerization”

**DOI:** 10.1021/acscentsci.3c00873

**Published:** 2023-08-03

**Authors:** J. Drake Johnson, Samuel W. Kaplan, Jozsef Toth, Zian Wang, Mitchell Maw, Sergei S. Sheiko, Aleksandr V. Zhukhovitskiy

Figure 2C presented the kinetic analysis of the polymerization of **M1** and **M2** to **polyM1** and **polyM2**, respectively. Following publication, the authors realized that kinetic data for the polymerization of **M1** was presented at [monomer]:[initiator] = 100:1 rather than the stated 200:1. Figure 2C and its caption have been corrected here to show the kinetic data for the polymerization of **M1** at [monomer]:[initiator] = 100:1 and 200:1. Additionally, ^1^H NMR data for this polymerization have been added to Figure S2, and its caption has been updated. Similarly, Figure S15’s caption incorrectly referenced a 100:1 [monomer]:[initiator] loading for **M2** rather than the 200:1 loading corresponding to the presented data. Figure S15’s caption has now been updated. Lastly, Figure S12 presented incorrect conversions due to a typographical error which has now been corrected. The above corrections do not change any analysis or conclusions in the original report. The revised Figures 2 is provided here and the revised Figures S2 and S12, as well as their captions, and the caption for Figure S15 are provided as Supporting Information.

**Figure 2:**
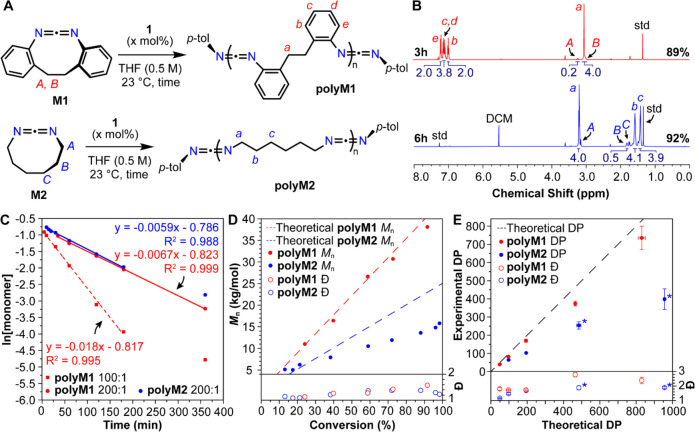
A. Polymerization of **M1** and **M2**, with key hydrogen atoms labeled. B. ^1^H NMR spectra of crude polymerization reaction mixtures for **M1** and **M2** at 3 and 6 h, respectively, in perdeuterated tetrahydrofuran (THF-*d*_8_). Key resonances are assigned, and % monomer conversions are indicated at the bottom right side of the spectra. std = 1,3,5-tri*tert*-butylbenzene. C. Semilogarithmic plot of monomer concentration versus time in the polymerization of **M1** and **M2** ([monomer]:[**1**] = 200:1, [monomer] = 0.5 M, temperature = 23 °C, Figures S2 and S3). The polymerization of **M1** in the same conditions, but with [monomer]:[**1**] = 100:1, is also shown. D. Evolution of *M*_n_ and *D̵*, measured by gel permeation chromatography with multiangle light scattering (GPC-MALS) as a function of monomer conversion for the polymerization of **M1** and **M2** ([monomer]:[**1**] = 200:1, [monomer] = 0.5 M, temperature = 23 °C, Figures S4 and S5). E. Experimental DP and *D̵*, as measured by GPC-MALS vs theoretical based on variation in [monomer]:[**1**] loadings and near-terminal conversions for the polymerization of **M1** and **M2** (Figures S6–S9, Tables S1 and S2); * = data for high-MW fraction only (Figure S8).

## Supporting Information Available

Corrected Figures S2 and S12 and the corrected caption for Figure S15 (PDF)

