# Supplementary material for: Carbodiimide Ring-Opening Metathesis Polymerization
Source: ACS Cent Sci. 2023 May 11;9(6):1104–10. doi: 10.1021/acscentsci.3c00032 (PMC10311665; doi:10.1021/acscentsci.3c00032)
Supplement: Supplementary file 1 — oc3c00032_si_001.pdf [file oc3c00032_si_001.pdf]

Supplementary Information

Carbodiimide Ring-opening Metathesis Polymerization

J. Drake Johnson,[1] Samuel W. Kaplan,[1] Jozsef Toth,[1] Zian Wang,[1] Mitchell Maw,[1] Sergei S. Sheiko,[1] Aleksandr V. Zhukhovitskiy[1]*

Affiliations:

[1] Department of Chemistry, University of North Carolina at Chapel Hill; Chapel Hill, NC 27599, USA.

*Corresponding author. Email: alexzhuk@email.unc.edu

MATERIALS AND METHODS 7

Purchased materials. 7

Nuclear magnetic resonance (NMR) spectroscopy. 7

Infrared spectroscopy. 8

Mass spectrometry. 8

Crystallographic Details 8

Analytical gel-permeation chromatography with multi-angle light scattering (GPC-MALS) 9

Analytical gel-permeation chromatography with multi-angle light scattering, with DMF mobile phase (GPC-MALS-DMF) 9

Preparative gel-permeation chromatography (prep-GPC). 9

Thermogravimetric analysis (TGA). 9

Differential scanning calorimetry (DSC). 10

Computational methodology. 10

Atomic force microscopy (AFM). 10

General experimental information. 10

Safety statement. 11

EXPERIMENTAL PROCEDURES 12

Synthesis 12

Synthesis of polyM1 via CDI ROMP of M1. 19

Synthesis of polyM2 via CDI ROMP of M2. 20

CDI ROMP setup for Figure 2C and Figure 2D from main text. 20

CDI ROMP setup for Figure 2E from main text. 20

CDI ROMP optimization procedures. 21

SUPPLEMENTARY FIGURES REFERENCED IN MAIN TEXT 22

Figure S1. 22

Figure S2. 23

Figure S3. 24

Figure S4. 25

Figure S5. 26

Figure S6. 27

Figure S7. 28

Figure S8. 29

Figure S9. 30

Figure S10. 31

Figure S11. 32

Figure S12. 33

Figure S13. 34

Figure S14. 35

Figure S15. 36

Figure S16. 37

Figure S17. 38

Figure S18. 39

Figure S19. 40

Figure S20. 41

Figure S21. 42

Figure S22. 43

Figure S23. 44

Figure S24. 45

Figure S25. 46

Figure S26. 47

Figure S27. 48

Figure S28. 49

SUPPLEMENTARY TABLES 50

Table S1 50

Table S2. 51

GPC CHARACTERIZATION 52

Figure S29. 52

Figure S30. 53

Figure S31. 54

Figure S32. 55

SPECTRAL CHARACTERIZATION 56

Figure S33. 56

Figure S34. 57

Figure S35. 58

Figure S36. 59

Figure S37. 60

Figure S38. 61

Figure S39. 62

Figure S40. 63

Figure S41. 64

Figure S42. 65

Figure S43. 66

Figure S44. 67

Figure S45. 68

Figure S46. 69

Figure S47. 70

Figure S48. 71

Figure S49. 72

Figure S50. 73

Figure S51. 74

Figure S52. 75

Figure S53. 76

Figure S54. 77

Figure S55. 78

Figure S56. 79

Figure S57. 80

Figure S58. 81

Figure S59. 82

Figure S60. 83

Figure S61. 84

Figure S62. 85

Figure S63. 86

Figure S64. 87

Figure S65. 88

Figure S66. 89

Figure S67. 90

Figure S68. 91

Figure S69. 92

Figure S70. 93

Figure S71. 94

Figure S72. 95

Figure S73. 96

Figure S74. 97
Figure S75. 98
Figure S76. 99
Figure S77. 100
Figure S78. 101
Figure S79. 102
Figure S80. 103
Figure S81. 104
Figure S82. 105
Figure S83. 106
Figure S84. 107
Figure S85. 108
Figure S86. 109
Figure S87. 110
Figure S88. 111
Figure S89. 112

THERMAL PROPERTY CHARACTERIZATION 113

Figure S90. 113
Figure S91. 114

CRYSTALLOGRAPHIC DATA....................115

Figure S92. 115

REFERENCES....................116

MATERIALS AND METHODS

Purchased materials.

Unless otherwise noted, solvents were used as received from VWR International. Deuterated solvents were used as received from Cambridge Isotope Laboratories, Inc. Anhydrous and deoxygenated tetrahydrofuran (THF), dichloromethane (DCM), benzene, toluene, and diethyl ether (Et_2O) were dried with a Pure Process Technologies solvent purification system[1] and stored in a nitrogen glovebox over 3Å molecular sieves. Triethylamine (Et_3N) was also dried with a Pure Process Technologies solvent system and used without further drying. Magnesium sulfate and sodium bicarbonate, and sodium nitrite were purchased from Fisher Chemicals and used without further purification. Pentamethylcyclopentadienyl iridium(III) chloride dimer ($[Cp^*IrCl_2]_2$) and tetrakis(triphenylphosphine)palladium ($Pd(PPh_3)_4$) were purchased from Strem, Inc. and stored within a nitrogen glovebox at -35 °C with no further purification. Potassium bis(trimethylsilyl)amide (KHMDS) as a 1 M solution in toluene was purchased from Sigma-Aldrich in a Sure/Seal container that was stored within a nitrogen glovebox with no further purification. *Para*-tolyl and *ortho*-tolyl isocyanate, *ortho*-tolyl isothiocyanate, triphenylphosphine (PPh_3), 4-dimethylaminopyridine (DMAP), di-*tert*-butyl dicarbonate (Boc_2O), sodium azide, palladium-on-carbon (Pd/C, 5 and 10% w/w Pd), *n*-hexylamine, methanesulfonyl chloride (MsCl), and lithium bromide were purchased from Sigma-Aldrich and used without further purification. Phenyl isothiocyanate was purchased from Sigma-Aldrich, freeze-pump-thawed, and dried over 3Å molecular sieves in a nitrogen glovebox prior to use. Trimethyloxonium tetrafluoroborate was purchased from Sigma-Aldrich and stored within a nitrogen glovebox at -35 °C with no further purification. 1,3,5-tri-*tert*-butylbenzene was purchased from Oakwood Chemicals and dried *in vacuo* for 16 h at 35 °C prior to use. Tetra-*n*-butylammonium fluoride (TBAF) as a 1 M solution in THF and pyridine was purchased from Sigma-Aldrich in Sure/Seal containers and used without further purification. Hydrogen sulfide as a 0.8 M solution in THF was purchased from Sigma-Aldrich and used without further purification. *n*-butylamine was purchased from Sigma-Aldrich, freeze-pump-thawed, and dried over 3Å molecular sieves for 16 hours prior to use. *Para*-toluidine was purchased from Alfa-Aesar and was purified via sublimation prior to use. 2-azacyclooctanone and 3-methyl-1-phenyl-2-phospholene-1-oxide was purchased from Combi-Blocks and were dried *in vacuo* for 16 hours at 35 °C prior to use. 1,2-bis(2-nitrophenyl)ethane, 4-biphenylboronic acid, and 4-(4-bromophenyl)-2-methylbut-3-yn-2-ol were purchased from Combi-Blocks and used without further purification. Di-*para*-tolyl (**L1**) and di-*ortho*-tolyl (**L2**) carbodiimide were prepared from *para*-tolyl and *ortho*-tolyl isocyanate following literature procedures.[2] Ultra-high purity grade nitrogen and hydrogen gases were purchased from Airgas, Inc. Aqueous hydrogen chloride (12.1 M) was purchased from Fisher Scientific. Mono-aminopropyl-terminated polydimethylsiloxane (2000 g/mol) was purchased from Gelest Inc. (MCR-A12).

Nuclear magnetic resonance (NMR) spectroscopy. Solution proton (^1H) and carbon-13 (^{13}C) spectra were collected on instruments operating at 400, 500 and 600 MHz for ^1H (100, 125, and 150 MHz for ^{13}C, respectively). These instrument model numbers and corresponding federal grant numbers are listed herein: Bruker AVANCE III 500 MHz (NSF Grant No. CHE-0922858) and Bruker AVANCE III 600 MHz (NSF Grant No. CHE-0922858). Solid-state ^{13}C spectra were collected on an instrument operating at 75 MHz of model number Bruker AVANCE II 300 MHz.

Chemical shifts in the presented spectra are reported in parts-per-million (ppm), and splitting patters are reported as singlet (s), doublet (d), triplet (t), quartet (q), quintet (quint), sextet (sext), septet (sept), octet (oct), multiplet (m), broad (b), and combinations thereof. Scalar coupling constants *J* are reported in Hertz (Hz). Spectra were processed and analyzed using MestReNova software v.12.0.3-21384 (Mestrelab Research, S.L.). The presented spectra are referenced to residual monoproteo-solvent peaks as reported in literature.[3]

Infrared spectroscopy. Attenuated total reflection-Fourier transform infrared spectroscopy (ATR-FTIR) spectra were acquired of solids, liquids, and oils with a Thermo Scientific Nicolet iS5 FTIR spectrometer with an iD7 diamond ATR accessory over 16 scans. Four background scans were performed before each sample was analyzed. Data was collected and baseline-corrected using Thermo Scientific OMNIC software.

Mass spectrometry. For **M2**, **S9**, and **L2-N**, high-resolution mass spectroscopy was obtained via a Thermo Scientific Exactive GC spectrometer equipped with an electron ionization (EI) source. The samples were analyzed in positive mode with splitless injections, a scan range of 50-600 m/z, an automatic gain control (AGC) target value of $1.0x10^6$, a resolution of 60,000, an ion source temperature of 250 °C, an inlet and MS transfer line temperature of 280 °C, a filament delay of 3.0 min, and a carrier gas flow rate of 1.50 mL/min. The results were analyzed with Xcalibur (ThermoFisher, Breman, Germany) software. Molecular formula assignments were determined with Molecular Formula Calculator (v 1.2.3). For **M2**, the initial GC oven temperature is set at 40 °C (held for 1 min), after which it is ramped to 300 °C at a rate of 25 °C/min (held 2.6 min). For **S9** and **L2-N**, the initial GC oven temperature is set at 150 °C (held for 1 min), after which it is ramped to 300 °C at a rate of 50 °C/min, then to 305 °C (held for 1 min) at a rate of 1.0 °C/min, and then to 325 °C (held for 1 min) at a rate of 5.0 °C/min.

For **S5** and **S6**, high-resolution mass spectroscopy was obtained via a 6220 ESI-TOF (Agilent Technologies) mass spectrometer equipped with an electrospray ionization source (ESI). The samples were analyzed at a flow rate of 25 µL/min. ESI source conditions were set as: nebulizer temperature 400 deg C, sheath gas (nitrogen) 20 arb, auxillary gas (nitrogen) 0 arb, sweep gas (nitrogen) 0 arb, capillary temperature 320 °C, RF voltage 45 V. The mass range was set to 100-3000 m/z. Solutions were analyzed at 0.1 mg/mL or less based on responsiveness to the ESI mechanism. Xcalibur (ThermoFisher, Breman, Germany) was used to analyze the data. Molecular formula assignments were determined with Molecular Formula Calculator (v 1.2.3). All observed species were singly charged, as verified by unit m/z separation between mass spectral peaks corresponding to the 12C and 13C12Cc-1 isotope for each elemental composition.

Crystallographic Details

Data collection. A single crystal of **1** was selected and mounted on a polymer tip covered in paratone oil on a Bruker APEX-II CCD diffractometer. The crystal was kept at 150 K during data collection. The data collection was carried out using Cu Kα radiation (λ = 1.54175 Å) (graphite monochromator) with a theta-dependent frame window of 10-20 seconds and a detector distance of 4.0 cm. Sections of frames were collected with 1.0° steps in ω scans. Data to a resolution of 0.82

Å were considered in the reduction. Integration of the data was accomplished using SAINT.[4] The intensity data were corrected for absorption (SADABS).[5] Please refer to Figure S90 for the resulting ORTEP diagram.

Structure solution and refinement. The space group P-1 was determined based on intensity statistics and systematic absences. The structure was solved using Superflip[6] and refined (full-matrix-least squares) using the Oxford University Crystals for Windows system.[7] The charge-flipping solution provided all non-hydrogen atoms from the E-map. Structure **1** did not contain any disorder or solvent accessible voids. All non-hydrogen atoms were refined with anisotropic displacement parameters. The hydrogen atoms were placed in ideal positions and refined as riding atoms. The final full matrix least squares refinement converged to R1 = 0.022 and wR2 = 0.054 (F^2, all data).

Analytical gel-permeation chromatography with multi-angle light scattering (GPC-MALS). Analytical GPC was performed on an Agilent Technologies 1260 Infinity II instrument equipped with two PL-gel 10 μm mixed-B light-scattering (LS) columns connected in series, with HPLC-grade THF stabilized with butylated hydroxytoluene (BHT) as the mobile phase at 35 °C, a Wyatt Technologies DAWN 8-angle light scattering (MALS) detector (λ = 658 nm), a Wyatt Technologies ViscoStar differential viscometer, and a Wyatt Technologies Optilab T-rEX differential refractometer. The samples were prepared by dissolution of the polymer samples at a concentration of ~3.0 mg/mL in the same THF as used for the mobile phase, followed by filtration through a 0.22-µm syringe filter. The *dn/dc* values were calculated for purified polymers in Astra software using the 100% mass recovery method.

Analytical gel-permeation chromatography with multi-angle light scattering, with DMF mobile phase (GPC-MALS-DMF). Analytical GPC for **polyM1-S** and **polyM1-N** was performed using a Tosoh Bioscience LLC EcoSEC HLC-8320GPC equipped with a TSKgel SuperH-RC 6.0 mm I.D. x 15 cm column and a differential refractive index (dRI) detector. Molecular masses were calculated using a calibration curve determined from polystyrene standards (PStQuick, Tosoh Bioscience, LLC) with 0.025 M LiBr in HPLC-grade DMF as the eluent flowing at 0.3 mL/min at 40 C, and sample concentrations ranging from 0.1 to 3.0 mg/mL in the same solvent as used for the mobile phase (filtered through a 0.22-µm syringe filter).

Preparative gel-permeation chromatography (prep-GPC). This purification technique was performed at room temperature with Recycling Preparative HPLC LaboACE LC-5060 Series equipped with JAIGEL-2HR column and ultra-violet (UV) and differential refractive index (dRI) detectors, with dichloromethane (DCM) as the eluent. The samples were prepared by dissolution of the polymer up to a concentration of 40 mg/mL in HPLC-grade DCM stabilized with amylene, followed by filtration through a 0.22-µm syringe filter. Samples were injected into the sample loop and recycled through the columns until baseline separation was obtained for the desired peak.

Thermogravimetric analysis (TGA). This analysis was carried out using a TA Instruments Q5000 Thermogravimetric Analyzer. Characterization was performed under flow of N_2 using

aluminum pans with a heating rate of 10 °C/min from room temperature to 600 °C. Values for the decomposition onset temperature (5% mass loss) were obtained from weight percent vs temperature (°C) plots.

Differential scanning calorimetry (DSC). This analysis was carried out on a TA Instruments Discovery DSC using aluminum pans, in air, and with variable heating and cooling rates between 10 and 40 °C/min, as specified. The data was analyzed in the TRIOS software v. 5.1.1.46572 by TA Instruments using the least-squares moving window technique for derivative and smoothing functions. Glass-transition temperatures (T_g) were calculated by the software based on the inflection transition type between two set bounds.

Computational methodology. Gaussian 16 suite[8] was employed for all Density Functional Theory (DFT) calculations, using computational parameters primarily set forth in the cited reference.[9] Initial geometry optimizations were performed with the M062X functional[10] with basis sets 6-31G* for C, H, and N,[11] and LANL2DZ with included effective core potential (ECP) for Ir.[12] Final energies and frequencies were calculated using the PBE functional[13,14] with empirical dispersion correction D2.[15] Basis sets used were: 6-311+G(d,p) for nitrogen,[11,16] 6-311G(d,p) for carbon and hydrogen,[16] and LANL2DZ with included ECP[12] augmented with one f-polarization function (0.938) for Ir.[17] The CPCM solvation model for THF[18,19] was also employed. All computations were done under SCF convergence criteria in combination with an ultrafine integration grid.

Atomic force microscopy (AFM). Mono-aminopropyl-terminated polydimethylsiloxane side chains (2 kg/mol, DP = 24) were grafted to **polyM1** with DP = 134 that was purified via prep-GPC (*vide infra,* **polyM1-BB** in Synthesis section). All glassware used in the synthesis and preparation of these samples was treated with 3:1 HCl/HNO_3 for 30 minutes, washed thoroughly with water, then oven-dried 16 h before use. Solutions of **polyM1-BB** at concentrations of 0.01 and 0.001 mg/mL in dichloromethane were prepared, and the bottlebrush was spin-cast onto a muscovite sheet. Atomic force microscopy imaging was performed in PeakForce QNM mode with a multimode AFM (Brüker) with a nanoscope V controller and silicon nitride tip (Scanasyst-Air by Brüker, resonance frequency = 50-90Hz, spring constant = 0.4 N/m).

General experimental information. All air-sensitive reactions were carried out in flame- or oven-dried glassware in a nitrogen-filled glove box or using standard Schlenk techniques under nitrogen gas, unless otherwise noted. Reaction mixtures were stirred with aqua regia-treated Teflon-coated magnetic stir bars. Reactions were monitored via NMR and TLC, and the TLC plates were visualized under UV irradiation or via standard staining procedures. Unless otherwise indicated, filtration was carried out using medium-porosity fritted glass-funnels. Removal of solvents *in vacuo* was achieved using an IKA-brand rotary evaporator or a Schlenk line (~30-60 mTorr, dynamic vacuum). Purification via flash chromatography was carried out following standard procedures. For reaction monitoring, kinetics, and precise monomer loading calculations, 1,3,5-tri-*tert*-butylbenzene was used as an internal standard. In the polymerization of **M1**, conversions were determined by integrating its 3.30 ppm ^1H NMR resonance ($CDCl_3$) at each time point and

comparing this to its integration before addition of **1**, with respect to the internal standard. In the polymerization of **polyM2**, conversions were determined by integrating at half-width its 3.25 ppm ^{1}H NMR resonance ($CDCl_3$) at each time point and comparing this to its integration before addition of **1**, with respect to the internal standard. Volumes smaller than 250 µL were transferred with Hamilton gas-tight syringes, which were washed following each transfer with solvents appropriate for each reaction. All vials used in polymerization studies were capped tightly and additionally sealed with electrical tape to prevent solvent evaporation. Pierced septa caps were replaced shortly after use, if multiple aliquots from a single vial were required.

Safety statement. Safety concerns for individual procedures are indicated in the relevant experimental procedures in red font. No unexpected safety hazards were encountered.

EXPERIMENTAL PROCEDURES

Synthesis

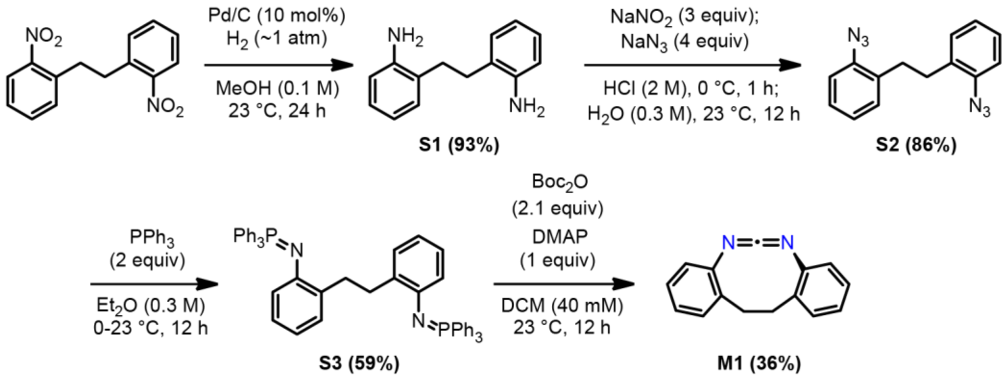

1,2-bis(2-aniline)ethane (S1) was prepared from a modified literature procedure.[20] To a 2.0 L round-bottom flask was added 1,2-bis(2-nitrophenyl)ethane (40.0 g, 147 mmol), palladium on carbon (Pd/C, 10% palladium by weight, 15.6 g, 14.7 mmol Pd), DCM (295 mL), and methanol (1.30 L). The headspace was briefly evacuated on a Schlenk line and backfilled with H_2 using a balloon of H_2. This mixture was stirred at 23 °C under positive pressure H_2 in balloons (~1 atm) until the reaction was complete (~ 16 h). At this point, the balloons with residual H_2 were removed and Celite® was added to the stirring mixture to mitigate the fire hazard during subsequent filtration. This mixture was then filtered through a pad of Celite® and concentrated to yield **S1** (29.0 g, 93%) as a dark brown solid that was used without further purification (95% purity). If additional purification is necessary, pure **S1** can be isolated via silica column chromatography (1:4 EtOAc/Hex, R_f = 0.20), yielding an off-white powder. The ^{1}H NMR spectrum of this material matched the spectrum reported in the cited reference.[21]

1,2-bis(2-azidophenyl)ethane (S2) was prepared from **S1** (29.0 g, 137 mmol), sodium nitrite (28.3 g, 410 mmol), sodium azide (35.5 g, 564 mmol), and hydrochloric acid (2 M, 970 mL, 1.94 mol) as reported in the cited reference (31.0 g, 86%).[22] Caution: solutions of sodium azide added to acidic solutions produces hydroazoic acid (HN_3), an odorless and highly toxic gas, explosive when mixed with air at low concentrations – ensure adequate ventilation when following this procedure. Ensure adequate ventilationThe ^{1}H NMR spectrum of this material matched the spectrum reported in the cited reference.[22]

1,2-bis(2-triphenylphosphaniminophenyl)ethane (S3) was prepared from **S2** (31.0 g, 117 mmol) and triphenylphosphine (60.9 g, 232 mmol) as reported in the cited reference[22], and was used without further purification (51.0 g, 59%). The ^{1}H NMR spectrum of this material matched the spectrum reported in the cited reference.[22]

M1 was prepared from **S3** (53.8 g, 73.4 mmol)**,** di-*tert*-butyl dicarbonate (36.3 mL, 158 mmol) and 4-dimethylaminopyridine (8.97 g, 73.4 mmol) as reported in the cited reference (5.50 g,

34%).[22] The ^{1}H and ^{13}C NMR spectra of this material matched the spectra reported in the cited reference.[22]

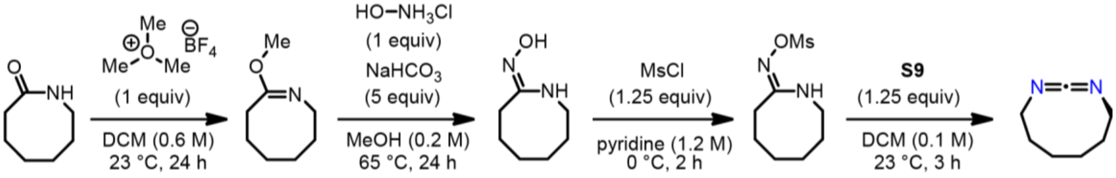

1-aza-2-methoxy-1-cyclooctene (S4) was prepared from 2-azacyclooctanone (8.34 g, 65.6 mmol) and trimethyloxonium tetrafluoroborate (9.70 g, 65.6 mmol) as reported in the cited reference (7.0 g, 76%).[23] The ^{1}H NMR spectrum of this material matched the spectrum reported in the cited reference.[23]

(*Z*)-azocan-2-one oxime (S5) was prepared according to a modified literature procedure.[24,25] To a 500 mL round-bottom flask was added hydroxylamine hydrochloride (3.20 g, 46.0 mmol), then methanol (220 mL). **S4** (6.50 g, 46.0 mmol) and sodium bicarbonate (19.3 g, 230 mmol) were added to the solution as solids and the mixture was stirred under reflux for 24 h. The crude reaction mixture was then filtered, and the filtrate was concentrated to yield crude **S5**, which contained sodium bicarbonate. This crude product was triturated on a fritted filtration funnel with DCM (220 mL), and the filtrate was concentrated to yield **S5** (6.05 g, 98%) as a white flakey solid. ^{1}H NMR (600 MHz, D_2O): δ 3.40 (t, J = 4.3 Hz, 2H), 3.36 (s, 1H), 2.40 (t, J = 6.2, 2H), 1.70 (ddt, J = 12.4, 8.7, 5.0 Hz, 2H), 1.60 (q, J = 6.0 Hz, 2H), 1.55 (m, 2H), 1.49 (m, 2H) ppm. ^{13}C {^{1}H} NMR (100 MHz, D_2O): δ 159.4, 39.5, 31.1, 29.7, 25.2, 24.5, 23.5 ppm. HRMS (ESI): calcd. for $^{12}C_7H_{15}{}^{14}N_2{}^{16}O$ $[M+H]^+$, m/z = 143.1184; found 143.1207.

(*Z*)-azocan-2-one O-methylsulfonyl oxime (S6) was prepared from **S5** (6.05 g, 42.6 mmol) and methanesulfonyl chloride (5.85 g, 51.1 mmol) as reported in the cited reference (8.62 g, 92%).[26] ^{1}H NMR (400 MHz, $CDCl_3$): δ 5.46 (bs, 1H), 3.32 (m, 2H), 3.13 (bs, 3H), 2.36 (t, J = 6.3 Hz, 2H), 1.77 (m, 2H), 1.56 (m, 6H) ppm. ^{13}C {^{1}H} NMR (100 MHz, $CDCl_3$): δ 163.4, 40.7, 36.0, 31.7, 30.4, 26.6, 25.4, 24.0 ppm. HRMS (ESI): calcd. for $^{12}C_8H_{17}{}^{14}N_2{}^{16}O_3{}^{32}S$ $[M+H]^+$, m/z = 221.0960; found 221.0982.

M2 was prepared via a modified literature procedure using **S9** (*vide infra*) in place of potassium *tert*-butoxide.[26,27] To a 500 mL round-bottom flask in an N_2 glovebox was added **S9** (6.60 g, 18.6 mmol), and anhydrous and deoxygenated DCM (273 mL). The flask was sealed, removed from the glovebox, attached to a Schlenk line, and chilled to 0 °C. Under a flow of N_2 to the vigorously stirred suspension was added **S6** (3.08 g, 14.0 mmol) as a solid in four portions over 20 minutes. Following complete addition of **S6**, the flask was removed from the ice bath and allowed to heat to 23 °C, and was stirred for 2 h. Removal of solvent under reduced pressure (~250 Torr at 25 °C, due to the potential volatility of the product), followed by vacuum transfer of the product at 70 mTorr (utilizing a Schlenk line) into a vessel cooled by liquid N_2, afforded **M2** as a clear, colorless, and odorous liquid (472 mg, 20%). ^{1}H NMR (600 MHz, $CDCl_3$): δ 3.25 (m, 4H), 1.84 (m, 4H),

1.73 (m, 4H) ppm. $^{13}C\ \{^{1}H\}$ NMR (150 MHz, $CDCl_3$): δ 150.0, 48.7, 28.0, 27.3 ppm. HRMS (EI): calcd. for $^{12}C_7H_{12}N_2$ $[M]^+$, m/z = 124.1000; found 124.0952. Our ^{1}H NMR data disagree with a prior report,[26] but we are confident in our assignments based on full ^{1}H, ^{13}C, and 2D NMR and mass spectroscopy characterization.

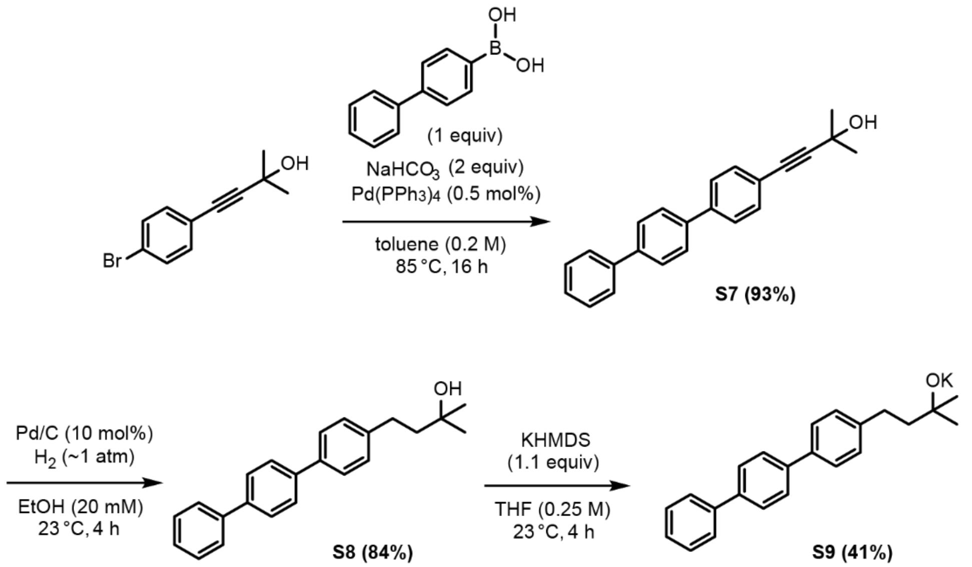

4-*p*-terphenyl-2-methylbut-3-yn-2-ol (S7) was prepared from a modified literature procedure.[28] To a 250 mL three-neck round-bottom flask was added toluene (120 mL), a suspension of sodium bicarbonate (3.51 g, 41.8 mmol) in water (21 mL), and a suspension of 4-biphenylboronic acid (4.14 g, 20.9 mmol) in 200-proof ethanol (63 mL). The headspace was briefly evacuated on the Schlenk line and backfilled with N_2, and the mixture was sparged with N_2 for an additional 30 minutes. Next, 4-(4-bromophenyl)-2-methylbut-3-yn-2-ol (5.0 g, 20.9 mmol) and tetrakis(triphenylphosphine) palladium(0) (100 mg, 0.09 mmol) were added under a stream of N_2 and the yellow mixture was stirred at 85 °C for 16 h. The crude mixture was then diluted with water (100 mL) and washed with ethyl acetate (3 x 100 mL). The organic layers were combined and washed with brine (3 x 100 mL). At this point, crystalline white solids had begun to precipitate from the organic layer—these solids were collected via filtration and washed with 50 mL water and 50 mL ethyl acetate to yield pure **S7** upon drying. The mother liquor was dried over magnesium sulfate, filtered, and concentrated to yield crude **S7** that was further purified via recrystallization from boiling ethyl acetate (initial precipitate and recrystallized portions combined, 6.08 g, 93%). The ^{1}H NMR spectrum of this material matches the spectrum reported in the cited reference.[29]

4-*p*-terphenyl-2-methylbutan-2-ol (S8) was prepared via a modified literature procedure.[30] To a 2.0 L round-bottom flask was added **S7** (5.57 g, 17.8 mmol), 200-proof ethanol (1.0 L), and palladium on carbon (Pd/C, 5% palladium by weight, 3.80 g, 1.78 mmol Pd). The headspace was briefly evacuated on the Schlenk line and backfilled with H_2 using a balloon of H_2. This mixture was stirred at 23 °C under positive pressure H_2 from balloons (~1 atm) until the reaction was complete (~ 3 h). At this point, the balloons with residual H_2 were removed and Celite® was added

to the stirring mixture to mitigate the fire hazard during subsequent filtration. This mixture was then filtered through a pad of Celite® and concentrated to yield **S8** (4.74 g, 84%) as a white powder.^{1}H NMR (500 MHz, $CDCl_3$): δ 7.67 (bs, 4H), 7.64 (m, 2H), 7.57 (dt, J = 8.3, 1.9 Hz, 2H), 7.46 (tt, J = 7.5, 1.8 Hz, 2H), 7.36 (tt, J = 7.4, 1.4 Hz, 1H), 7.30 (d, J = 8.2 Hz, 2H), 2.77 (m, 2H), 1.85 (m, 2H), 1.32 (s, 6H), 1.26 (bs, 1H) ppm. ^{13}C {^{1}H} NMR (100 MHz, $CDCl_3$): δ 141.9, 140.9, 140.1, 140.0, 138.4, 129.0, 127.6, 127.5, 127.4, 127.2, 127.1, 71.1, 45.9, 30.5, 29.5 ppm. HRMS (EI): calcd. for $^{12}C_{23}H_{24}{}^{16}O$ $[M]^+$, m/z = 316.1827; found 316.1822.

Potassium 4-*p*-terphenyl-2-methylbutan-2-olate (S9) was prepared by under an N_2 atmosphere. To a 100 mL Schlenk flask, **S8** (6.60 g, 20.9 mmol) is added, followed by anhydrous and deoxygenated tetrahydrofuran. To this stirring suspension is added dropwise KHMDS (1.0 M in THF, 22.9 mL, 22.9 mmol). After stirring at 23 °C for 2 more hours, the mixture is concentrated *in vacuo* to a brown solid. Trituration of this solid with anhydrous and deoxygenated hexanes (100 mL) in the glove box followed by drying for 24 h *in vacuo* afforded **S9** (3.05 g, 41%) as a tan powder. ^{1}H NMR (500 MHz, THF-d_8): δ 7.68 (s, 4H), 7.66 (d, J = 7.0 Hz, 2H), 7.57 (d, J = 8.2 Hz, 2H), 7.41 (t, J = 7.8 Hz, 2H), 7.29 (m, 3H), 2.74 (m, 2H), 1.74 (m, 2H), 1.21 (s, 3H) ppm. ^{13}C {^{1}H} NMR (125 MHz, THF-d_8): δ 143.5, 141.5, 140.8, 140.4, 138.6, 129.4, 129.3, 127.9, 127.8, 127.7, 127.4, 127.3, 69.2, 46.9, 31.1, 29.7, 26.2 ppm.

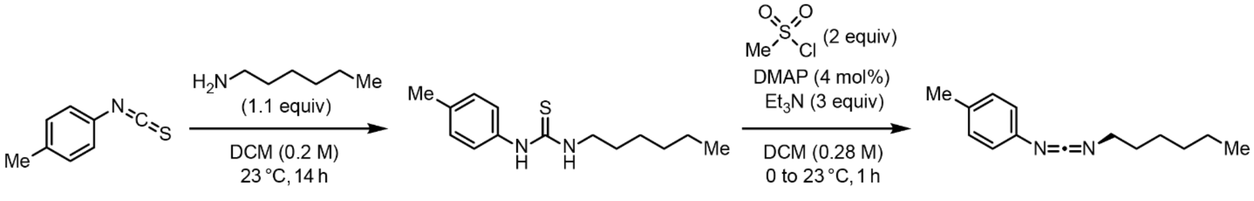

1-hexyl-3-(*p*-tolyl)thiourea (S10) To a 50 mL 2-neck flask under an N_2 atmosphere, 1-isothiocyanato-4-methylbenzene (714 mg, 4.79 mmol) was added, followed by anhydrous and deoxygenated DCM (23.9 mL). *n*-hexylamine (695 uL, 5.26 mmol) was then added to the flask, and the solution was stirred at 23 °C for 14 h. The solution was concentrated under reduced pressure and passed through a plug of silica, and the silica was washed with additional DCM (5 mL) to afford **S10** (1.13 g, 94%) as a yellow solid. ^{1}H NMR (600 MHz, CD_2Cl_2): δ 7.24 (d, J = 8.0 Hz, 2H), 7.12 (d, J = 8.2 Hz, 2H), 3.51 (t, J = 7.3 Hz, 2H), 2.36 (s, 3H), 1.57 (quint, J = 7.0 Hz, 2H), 1.49-1.04 (m, 6H), 0.88 (t, J = 6.8 Hz, 3H). ^{13}C {^{1}H} NMR (150 MHz, CD_2Cl_2): δ 138.6, 133.6, 131.0, 126.2, 47.1, 31.7, 29.0, 26.8, 22.9, 21.2, 14.2 ppm. HRMS (EI): calcd. for $^{12}C_{14}H_{22}{}^{14}N_2{}^{32}S$ $[M]^+$, m/z = 250.1504; found 250.1500.

***N*-hexyl-*N*-(*p*-tolyl)methanediimine (L3)** was prepared following a modified literature procedure.[31] To a 20 mL round-bottom flask, **S10** (140 mg, 0.56 mmol) was added, followed by triethylamine (233 uL, 1.68 mmol), 4-dimethylaminopyridine (2.7 mg, 22 umol), and DCM (2.0 mL). This solution was cooled to 0 °C, and methanesulfonyl chloride (86.5 uL, 1.12 mmol) was added dropwise, during which the solution color transitioned from clear to pale orange. The reaction was returned to 23 °C and stirred for 1 h. The solution was then passed through a plug of

silica, and the silica was washed with additional DCM (5 mL). The solution was concentrated under reduced pressure and purified via column chromatography (silica, 75% hexanes, 25% ethyl acetate) to afford **S11** (40 mg, 33%) as a white solid. ^{1}H NMR (600 MHz, $CDCl_3$): δ 7.09 (d, J = 8.0 Hz, 2H), 6.98 (d, J = 8.2 Hz, 2H), 3.39 (t, J = 6.9 Hz, 2H), 2.31 (s, 1H), 1.67 (quint, J = 7.2 Hz, 2H), 1.41 (quint, J = 7.3 Hz, 2H), 1.31 (m, 4H), 0.89 (t, J = 7.0 Hz, 3H) ppm. ^{13}C {^{1}H} NMR (150 MHz, $CDCl_3$): δ 138.0, 136.8, 134.4, 130.1, 123.4, 47.1, 31.5, 31.4, 26.6, 22.7, 21.0, 14.1 ppm. HRMS (EI): calcd. for $^{12}C_{14}H_{20}{}^{14}N_{2}{}^{32}$ $[M]^+$, m/z = 216.1626; found 216.1623.

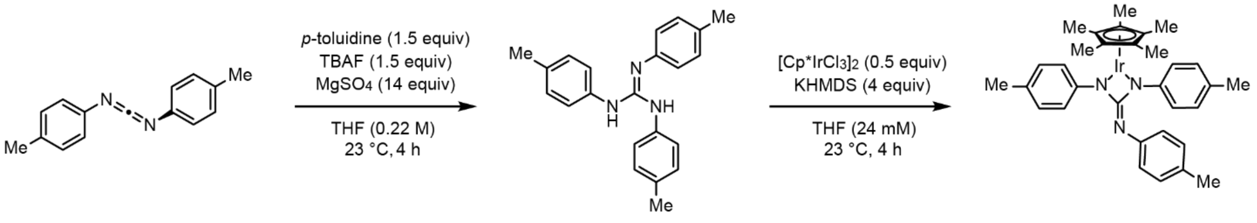

S11 (50%) **1 (51%)**

***N,N',N''*-tri-(p-tolyl)guanidine (S11)** was prepared from a modified literature procedure.[32] To a 200 mL Schlenk flask under an N_2 atmosphere, *p*-tolyl carbodiimide (3.50 g, 15.7 mmol), *p*-toluidine (2.53 g, 23.6 mmol), and magnesium sulfate (27.2 g, 220 mmol) were added. Anhydrous and deoxygenated THF (70 mL) was then added, followed by the dropwise addition of a solution of tetra-*n*-butylammonium fluoride (1 M in THF, 24.4 mL, 24.4 mmol). The pale-yellow solution was stirred for 4 h at 23 °C, and then poured into 250 mL of an aqueous pH ~7 phosphate buffer solution, forming a cloudy white suspension. This suspension was placed in a 1.0 L separatory funnel and extracted with DCM (3 x 200 mL). The organics were dried with magnesium sulfate, then filtered and concentrated under reduced pressure to yield an orange oil. This oil is dissolved in Et_2O (50 mL), then extracted with a saturated NH_4Cl solution (3 x 50 mL) to remove remaining tetra-*n*-butylammonium fluoride. The organics are again dried with magnesium sulfate, filtered, and concentrated under reduced pressure. The resulting oil is then washed with toluene (20 mL at 100 °C), which cause**d** precipitation of a white solid (urea side-product) that is removed via filtration. The filtrate is concentrated under reduced pressure, and the resulting oil is subjected to sublimation (20 mTorr static vacuum, heated to 85 °C, deposition surface cooled to -78 °C with solid CO_2) to remove remaining *p*-toluidine to afford **S12** (2.61 g, 50% yield) as a pale-yellow solid. The ^{1}H NMR spectrum of this material matches the spectrum reported in the cited reference.[33]

Pentamethylcyclopentadienyl iridium *N,N',N''*-tri-(*p*-tolyl)guanidinate (1) was prepared following a modified literature procedure.[34] To a 100 mL Schlenk flask under an N_2 atmosphere, $[Cp^*IrCl_3]_2$ (300 mg, 0.377 mmol) was added. Anhydrous and deoxygenated THF (14 mL) and *N,N',N''*-tri-(*p*-tolyl)guanidine (248 mg, 0.754 mmol) were then added, forming an orange suspension to which of KHMDS (0.5 M in toluene, 3.02 mL, 1.51 mmol) was added dropwise. This caused a color change of the solution to dark blue-green over the course of addition. After stirring for 4 more hours at 23 °C, the volatiles were removed *in vacuo*. The remaining solids were washed with anhydrous and deoxygenated benzene (2 x 20 mL) in the glove box and the washings were then filtered through a thick pad of Celite®. The filtrate was dried *in vacuo* to yield a dark

green solid. This material was dissolved in 3.5 mL of anhydrous and deoxygenated benzene and filtered through a 0.22 µm syringe filter into a 20 mL scintillation vial, which was then placed in a screw-cap 100 mL glass media bottle containing anhydrous and deoxygenated hexanes (35 mL) for recrystallization via via vapor diffusion. After 48 h, the mother liquor was decanted and the remaining solids were washed with anhydrous and deoxygenated hexanes (2 x 10 mL), then dried *in vacuo* to afford **1** (253 mg, 51% yield) as dark green crystals. ^{1}H NMR of this material matches the spectrum reported in the cited reference.[34]

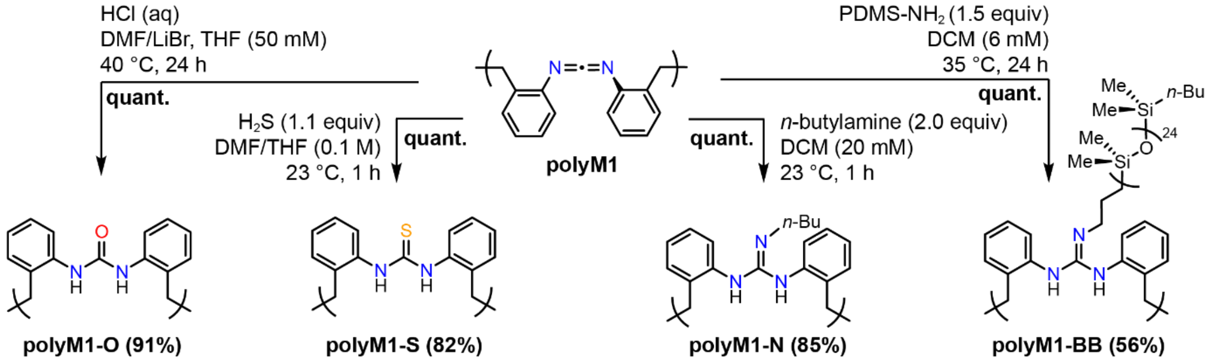

polyM1-O was prepared by first adding to a 2-dram vial **polyM1** (50.0 mg, 0.20 mmol). THF (975 µL) was then added, followed by the dropwise addition of a solution of 0.025 M lithium bromide in DMF (2.93 mL). This solution was heated to 40 °C to promote complete dissolution of **polyM1**, and then aqueous hydrochloric acid (5 M, 80 µL, 0.40 mmol) was added dropwise at this temperature. After stirring for 1 h at 40 °C, the mixture becomes gel-like and immobile. After the mixture returned to 23 °C, it was transferred to a 150 mL Erlenmeyer flask. Water (30 mL) was added, which caused a white precipitate to form that was then isolated via filtration, washed with ether (20 mL) to remove excess water and DMF, and dried *in vacuo* at 60 °C for 24 h to afford **polyM1-O** (49 mg, 91%) as a white powder. ^{13}C CP/MAS ssNMR (100 MHz, 12 KHz): δ 158.3, 136.2, 129.9, 126.7, 32.9. ATR-FTIR (v, cm^{-1}): 3273, 3059, 3045, 3016, 2958, 2870, 2786, 1637, 1584, 1537, 1450, 1290, 1237, 1182, 747.

polyM1-S was prepared from a modified literature procedure.[35] To a 2-dram vial under an N_2 atmosphere, **polyM1** (100 mg, 0.45 mmol) and anhydrous and deoxygenated THF (1.0 mL) were added. DMF (2.90 mL) was then added to this solution dropwise, causing the solution to turn pale yellow. Hydrogen sulfide (H_2S) in THF (0.8 M, 624 µL, 0.50 mmol) was then added dropwise (Caution: hydrogen sulfide is a toxic and highly malodorous reagent – handle with adequate ventilation), and the solution was stirred for 1 h at 23 °C. A vent needle attached to a bubbler containing iron(III) oxide suspended in water was then connected, and the reaction was purged with N_2 to remove excess H_2S. The purged solution was then poured into water (40 mL), causing a pale-yellow suspension to form. Filtration of this mixture yielded yellow crystalline solids, which were triturated with diethyl ether (20 mL) to remove excess water and DMF. These solids were placed in DCM (15 mL), vortexed for 10 minutes to remove any remaining DMF, then isolated via filtration and triturated with additional diethyl ether (20 mL). The solids were dried *in vacuo* for 24 h at 60 °C to afford **polyM1-S** (95 mg, 82%) as a yellow powder. ^{1}H NMR (600 MHz,

DMSO-d_6): δ 9.33 (bs, 2H), 7.29 (d, J = 7.7 Hz, 2H), 7.24 (d, J = 7.4 Hz, 2H), 7.19 (t, J = 7.5 Hz, 2H), 7.13 (t, J = 7.3 Hz, 2H). ^{13}C {1H} NMR (150 MHz, DMSO-d_6): δ 182.1, 139.0, 137.5, 129.7, 129.2, 126.9, 126.5, 32.1. ATR-FTIR (*v*, cm^{-1}): 3329, 3132, 3063, 2942, 1492, 1450, 1337, 1255, 1200, 749, 637.

polyM1-N was prepared from a modified procedure for the addition of primary amines to analogous small molecule carbodiimides.[36] To a 2-dram vial under an N_2 atmosphere, **polyM1** (100 mg, 0.45 mmol) was added. Anhydrous and deoxygenated DCM (20 mL) and *n*-butylamine (89 µL, 0.90 mmol) were then added, and the solution was stirred for 1 h at 23 °C. The volatiles were removed under reduced pressure, and the resulting oil was dried *in vacuo* at 30 °C for 16 h, furnishing a white solid. These solids were triturated with pentane (5 mL) to remove trace *n*-butylamine, then dried again *in vacuo* for 16 h at 40 °C to yield **polyM1-N** as a white flakey solid (113 mg, 85%). Approximately 5.7 mol% pentane remained in the sample. ^1H NMR (600 MHz, $CDCl_3$): δ 7.24-6.72 (m, 8H), 5.50 (bs, 1H), 3.81 (bs, 1H), 3.30 (s, 2H), 2.79 (bs, 4H), 1.49 (m, 2H), 1.29 (q, J = 7.4 Hz, 2H), 0.86 (t, J = 7.4 Hz, 3H). ^{13}C {1H} NMR (150 MHz, DMSO-d_6): δ 148.2, 136.9, 130.1, 127.1, 125.7, 123.2, 41.5, 32.8, 32.0, 20.3, 13.9. ATR-FTIR (*v*, cm^{-1}): 3396, 3056, 3013, 2953, 2925, 2867, 1628, 1586, 1478, 1286, 1237, 746.

polyM1-BB was prepared from a modified procedure for the addition of primary amines to analogous small molecule carbodiimides.[36] To a 20 mL vial under an N_2 atmosphere, **polyM1** (11 mg, 50 µmol) was added. Anhydrous and deoxygenated DCM (8.4 mL) and PDMS-NH_2 (M_n = 2 kg/mol, 100 µL, 50 µmol) were then added, and the solution was stirred for 16 h at 35 °C. Concentration under reduced pressure yielded an oil to which methanol (10 mL) was added, which caused phase-separation of a cloudy white oil over 10 minutes. Following centrifugation (10 minutes, 4000 rpm), the mother liquor was decanted and the white oil was washed with DCM (5 mL) to remove unreacted PDMS-NH_2. After drying *in vacuo* at 23 °C for 16 h, **polyM1-BB** (62 mg, 56%) is isolated as a viscous, colorless oil. ^1H NMR (600 MHz, CD_2Cl_2): δ 7.54-6.45 (m, 8H), 3.91 (bs, 0.6H), 3.28 (bs, 1.4H), 2.79 (bs, 4H), 1.32 (m, 3H), 0.88 (m, 2H), 0.58-0.46 (m, 3H), 0.10-0.0 (m, 117H). ^{13}C {1H} NMR (150 MHz, CD_2Cl_2): δ 26.8, 25.8, 24.2, 18.3, 15.9, 14.0, 1.4, 1.3, 1.26, 1.22, 1.20, 0.92, 0.25. ATR-FTIR (*v*, cm^{-1}): 2960, 1640, 1257, 1014, 794.

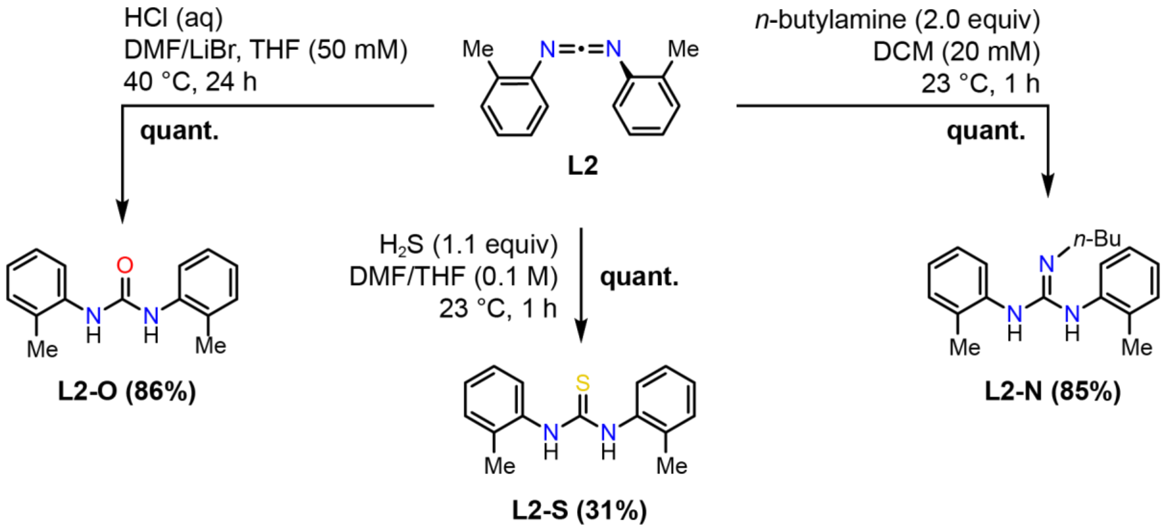

L2-O was prepared analogously to **polyM1-O**, but with **L2** used in place of **polyM1**, affording the product (25 mg, 86%) as a white solid. ^{1}H and ^{13}C NMR matched that which is reported in the cited reference.[37]

L2-S was prepared analogously to **polyM1-S**, but with **L2** used in place of **polyM1**, affording the product (36 mg, 31%) as a pale-yellow solid. ^{1}H and ^{13}C NMR matched that which is reported in the cited reference.[38]

L2-N was prepared analogously to **polyM1-N**, but with **L2** (100 mg, 0.45 mmol) used in place of **polyM1**, affording **L2-N** (113 mg, 85%) as a white crystalline solid. ^{1}H NMR (400 MHz, $CDCl_3$): δ 7.24-6.78 (m, 8H), 5.26 (bs, 1H), 3.77 (bs, 1H), 3.34 (bs, 2H), 2.22 (bs, 4H), 1.54 (quint, J = 7.1 Hz, 2H), 1.35 (sext, J = 7.1 Hz, 2H), 0.93 (t, J = 7.2 Hz, 3H) ppm. ^{13}C {^{1}H} NMR (100 MHz, $CDCl_3$): δ 147.7, 131.7, 130.8, 126.9, 125.8, 123.2, 41.3, 31.9, 20.2, 17.9, 13.9 ppm. HRMS (EI): calcd. for $^{12}C_{19}H_{25}{}^{14}N_3$ $[M]^+$, m/z = 295.2048; found 295.2048. ATR-FTIR (*v*, cm^{-1}): 3432, 3395, 3056, 3010, 2954, 2925, 2869, 1635, 1590, 1516, 1481, 1374, 1312, 1287, 1242, 1110, 1041, 754, 725.

Synthesis of polyM1 via CDI ROMP of M1. To a 20 mL scintillation vial under an N_2 atmosphere, **M1** (480 mg, 2.18 mmol) is added followed by anhydrous and deoxygenated THF (0.5 M). A solution of **1** (14.3 mg, 21.8 μmol) in anhydrous and deoxygenated THF (500 μL) is prepared and added to the **M1** solution rapidly. The dark green solution is stirred for 3 h at 23 °C. The polymerization is quenched with the addition of phenyl isothiocyanate (18 μL), and the solution rapidly turns yellow. **PolyM1** is then isolated via precipitation into methanol (50 mL, 0 °C), forming a yellow, highly adhesive paste that is dried *in vacuo* to afford **polyM1**(370 mg, 77%) as a yellow solid. The crude material can be further purified via prep-GPC (DCM), affording **polyM1** (230 mg, 48%) as a white, flakey solid. ^{1}H NMR (600 MHz, $CDCl_3$): δ 7.17 (d, J = 7.7 Hz, 2H), 7.10 (m, 4H), 7.00 (t, J = 7.4 Hz, 2H), 3.02 (s, 4H), 2.29 (s, 0.04H) ppm. ^{13}C {^{1}H} NMR (150 MHz, $CDCl_3$): δ 137.1, 135.9, 134.1, 130.3, 127.3, 125.5, 125.0, 32.9 ppm. ATR-FTIR (*v*,

cm^{-1}): 3058, 3018, 2953, 2925, 2857, 2096, 1574, 1483, 1446, 1214, 1178, 1154, 1084, 746, 602. *dn/dc* in THF at 30 °C = 0.2239 mL/g.

Synthesis of polyM2 via CDI ROMP of M2. To a 2-dram vial under an N_2 atmosphere, **M2** is added (161 mg, 1.30 mmol), followed by anhydrous and deoxygenated THF (2.6 mL). A stock solution of **1** in anhydrous and deoxygenated THF (10 mg, 250 µL, 0.061 M) is prepared and added (107 uL, 6.48 umol) rapidly to the **M2** solution. The dark green solution is stirred 24 h at 23 °C, with the solution color transitioning to brown several minutes following injection of **1**. Phenyl isothiocyanate (7.74 µL, 64.8 µmol) is then injected rapidly and the solution is stirred for 10 more minutes. A gradual change in solution color from dark brown to yellow-orange is observed during this time. The solution is added to a 50 mL plastic culture tube containing hexanes (40 mL at 0 °C), which caused a pale-yellow suspension to form. This suspension began to phase separate, and following centrifugation (10 min., 4000 rpm) and decantation of the hexanes mother liquor, **polyM2** (61 mg, 38%) is afforded as a highly viscous, pale-yellow oil. Smaller molecular weights were found to be soluble in hexanes and are not accounted for in the isolated yield (*vide infra*, Figure S8).^{1}H NMR (600 MHz, $CDCl_3$): δ 7.09 (d, J = 8.5 Hz, 0.04H), 6.97 (d, J = 8.4 Hz, 0.04H), 3.21 (t, J = 6.9 Hz, 4H), 2.31 (s, 0.05H), 1.57 (m, 4H), 1.38 (quint, J = 3.5 Hz, 4H) ppm. ^{13}C {^{1}H} NMR (150 MHz, $CDCl_3$): δ 140.4, 46.8, 31.4, 26.6 ppm. ATR-FTIR (*v*, cm^{-1}): 2930, 2856, 2113, 1673, 1454, 1337, 977. *dn/dc* in THF at 30 °C = 0.1172 mL/g.

CDI ROMP setup for Figure 2C and Figure 2D from main text.
Under an N_2 atmosphere, stock solutions of **1** (0.06 M), 1,3,5-tri-*tert*-butylbenzene (0.4 M), and phenyl isothiocyanate (0.8 M) in anhydrous and deoxygenated THF are prepared in 1-dram vials. **M2** (49 mg, 0.40 mmol) is added to a 1-dram vial containing a stir bar, followed by 40 µL (4.0 mg, 0.016 mmol) of the 1,3,5-tri-*tert*-butylbenzene stock. The solution is diluted to a volume of 200 µL, and 12 µL are withdrawn for a starting time point. The solution is further diluted with THF to a total volume of 710 µL, then 30.5 µL (1.21 mg, 1.85 µmol) of **1** stock is added rapidly. At reaction times of 0.17, 0.25, 0.33, 0.5, 1, 2, 3, 6, 12, and 24 hours, 64 µL aliquots are withdrawn and quenched with 5 µL (4 µmol) of phenyl isothiocyanate stock – this corresponds to ~10 equivalents of phenyl isothiocyanate per mol of **1** in the aliquot. The terminated aliquots transition from a brown color to a deep yellow over 1 minute. Three-quarters of each aliquot is diluted with HPLC-grade stabilized (BHT) THF to a concentration of ~3.0 mg/mL and analyzed via GPC-MALS for molecular weight determination. The remainder of the aliquot is taken into $CDCl_3$ and analyzed via ^{1}H NMR for conversion determination. The same procedure was utilized for studying **M1** at the same concentration, except **M1** is first dissolved with heating (30 °C) in anhydrous and deoxygenated THF before a starting time point was taken, and timepoints were taken at 0.58, 1, 2, 3, and 6 hours.

CDI ROMP setup for Figure 2E from main text. Under an N_2 atmosphere in anhydrous and deoxygenated THF, stock solutions of **1** (10 mg in 500 µL, 0.03 M), **M2** (80 mg in 500 µL, 1.28 M), 1,3,5-tri-*tert*-butyl benzene (10 mg in 100 µL,0.4 M), and phenyl isothiocyanate (10 µL in 240 µL, 0.26 M) were prepared in 1-dram vials with septa caps. Into individual 1-dram vials, **M2** stock (74.4 µL, 11.9 mg), 1,3,5-tri-*tert*-butyl benzene stock (10 µL, 1.0 mg), and anhydrous and

deoxygenated THF (16.6 µL) were added. An aliquot (10 µL) is removed from each vial to serve as a starting time point for conversion calculations. Anhydrous and deoxygenated THF (97.5 µL) was added to each vial, and then **1** stock (2.5 µL, 0.096 µmol **1**) was injected into each rapidly. The reactions were stirred for 48 h at 23 °C, and an aliquot (10 uL) was taken and quenched with phenyl isothiocyanate (10 equivalents relative to **1**) to check conversion by ^{1}H NMR in $CDCl_3$. An additional time point was taken 4 h later, which showed no additional conversion. All reactions were then quenched with phenyl isothiocyanate stock (10 equivalents relative to **1**) and stirred 5 minutes, during which the solution color transitions for brown to light yellow. An aliquot (40 µL) containing ~3 mg of **polyM2** was taken from each reaction, diluted to 1 mL with HPLC-grade stabilized (BHT) THF, filtered, and analyzed via GPC-MALS to determine M_n and Đ. The same procedure is utilized for studying **M1** at the same concentration, except **M1** is first dissolved with heating (30 °C) in anhydrous and deoxygenated THF before a starting timepoint was taken, and the reactions were run at 23 °C for 6-12 h rather than 48 h.

CDI ROMP optimization procedures. The same procedure listed above for the synthesis of **polyM1** was used for the investigation of temperature and solvent effects on the polymerization. At a **M1** concentration of 0.5 M and an **M1**-to-**1** loading of 100:1 in each reaction, this procedure was followed with DCM, chloroform, benzene, and chlorobenzene as the solvent. Furthermore, in THF, this procedure was followed, but at temperatures of 0 °C and -40 °C.

SUPPLEMENTARY FIGURES REFERENCED IN MAIN TEXT

A

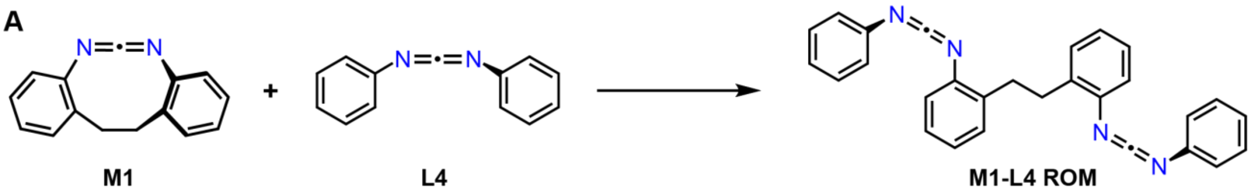

M1 **L4** **M1-L4 ROM**

	M1	L4	M1-L4 ROM
Gibbs Free Energy (kcal/mol)	-431372.4968	-382861.5718	-814236.3583
Enthalpy (kcal/mol)	-431339.0529	-382826.9539	-814175.5398

M1 Ring Strain
ΔG_{rxn} = -2.3 kcal/mol
ΔH_{rxn} = -9.5 kcal/mol

B

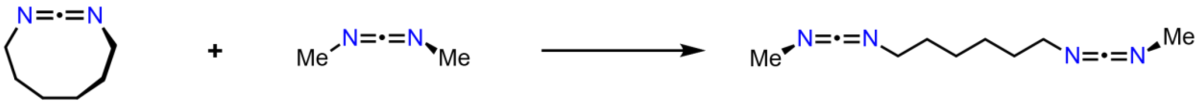

M2 **L5** **M2-L5 ROM**

	M2	L5	M2-L5 ROM
Gibbs Free Energy (kcal/mol)	-240278.5952	-142519.397	-382799.8452
Enthalpy (kcal/mol)	-240252.0074	-142495.4303	-382755.5864

M2 Ring Strain
ΔG_{rxn} = -1.9 kcal/mol
ΔH_{rxn} = -8.1 kcal/mol

Figure S1. Computational analysis of the ring-strain energies of **M1** and **M2**. Ring strain of **M1** and **M2** were calculated by measuring the ΔG and ΔH of ring opening metathesis for each monomer. To limit electronic effects of the resulting carbodiimides, **M1** was modeled to react with 1,3-diphenyl carbodiimide **L4**, whereas **M2** was modeled to react with 1,3-dimethyl carbodiimide **L5**. DFT calculations were performed at a PBE/D2 level of theory with basis sets 6-311+G(d,p) and 6-311G(d,p) for nitrogen, and carbon and hydrogen, respectively, in addition to the CPCM solvation model for THF.

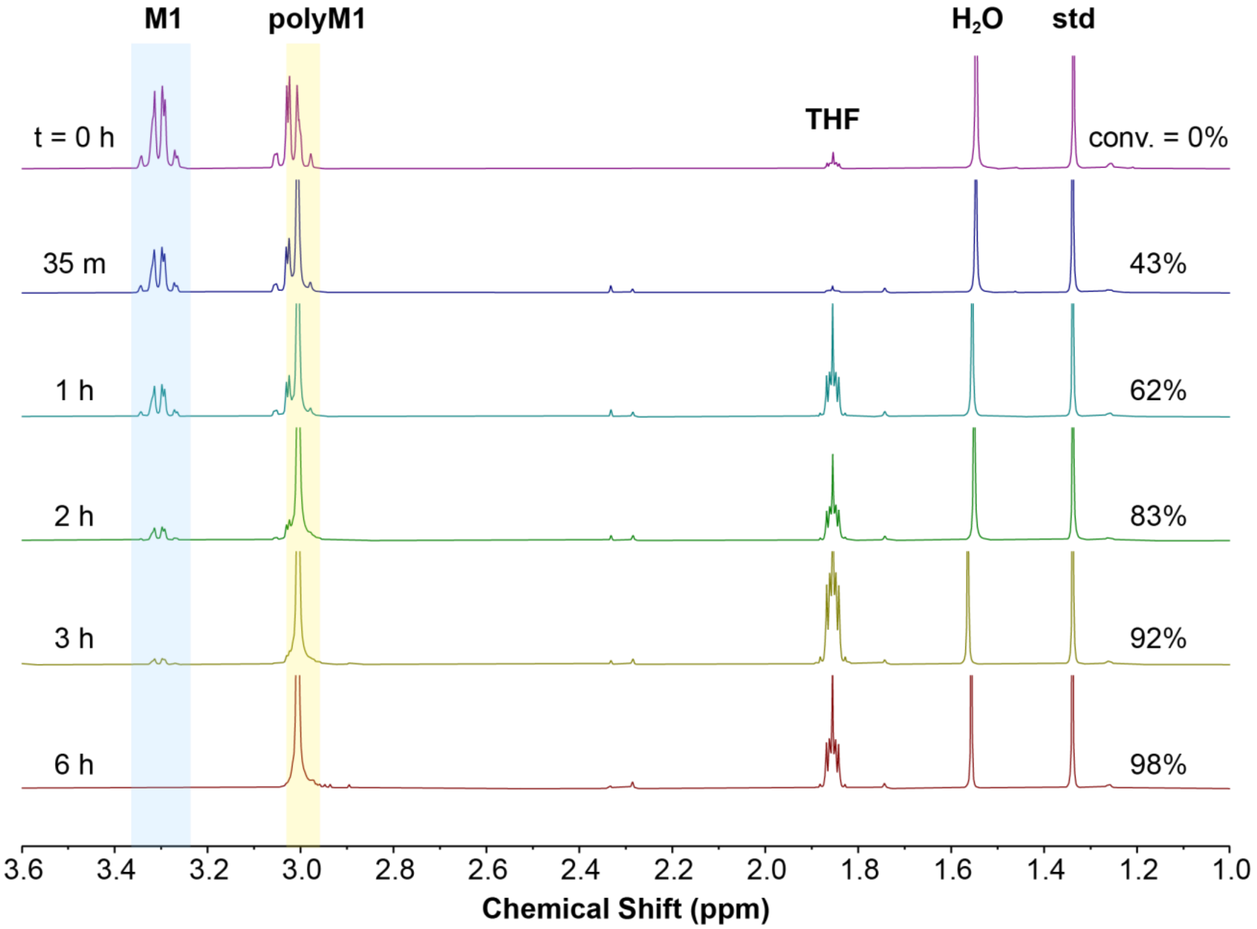

Figure S2. ^{1}H NMR (600 MHz, $CDCl_3$, 23 °C) spectra of a 100:1 [**M1**]:[**1**] polymerization at various timepoints (indicated on the left) with conversion of **M1** indicated on the right. Residual H_2O originates from the NMR solvent bottle.

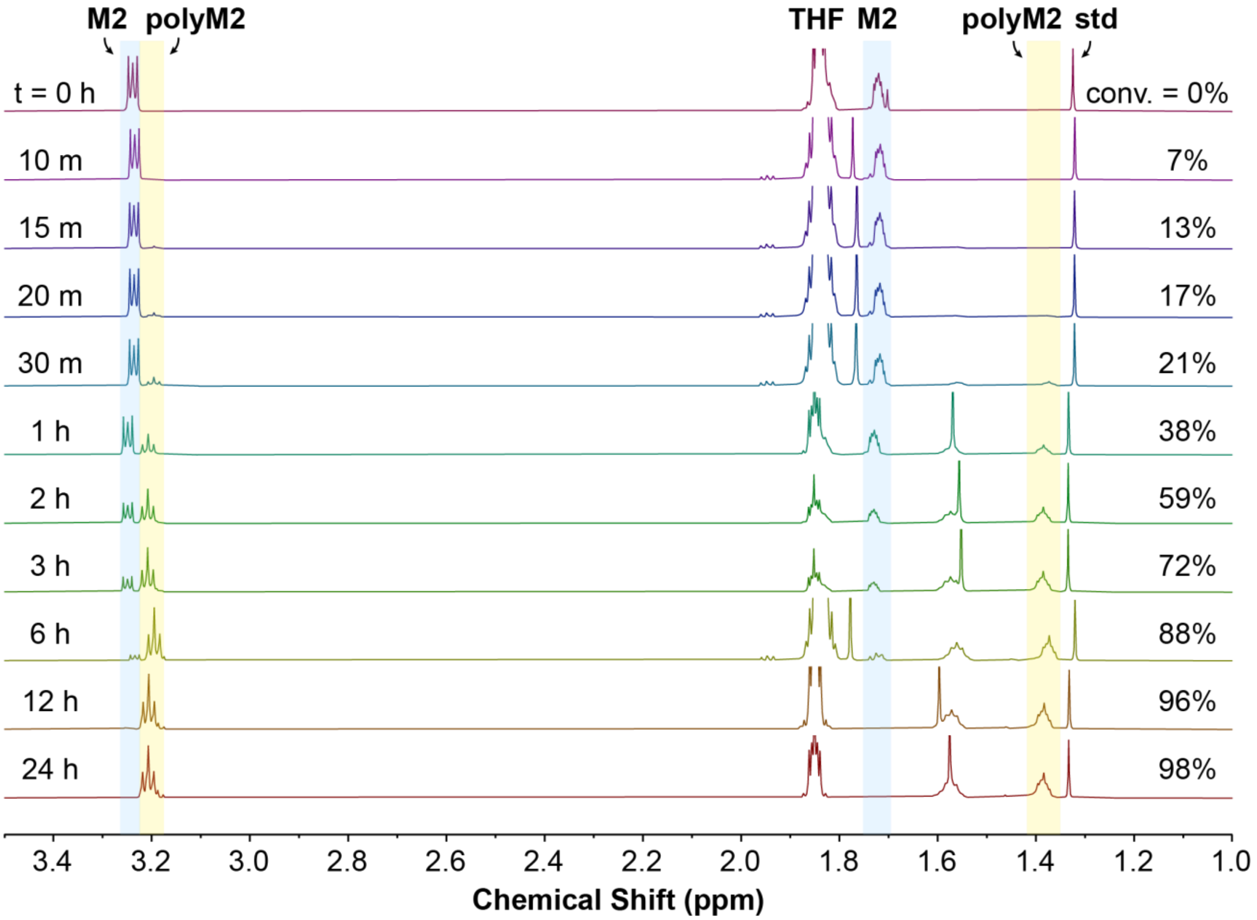

Figure S3. ^{1}H NMR (600 MHz, $CDCl_3$, 23 °C) spectra of a 200:1 [**M2**]:[**1**] polymerization at various timepoints (indicated on the left) with conversion of **M2** indicated on the right. Residual THF causes the resonance for H_2O from the NMR solvent to shift in several aliquots.

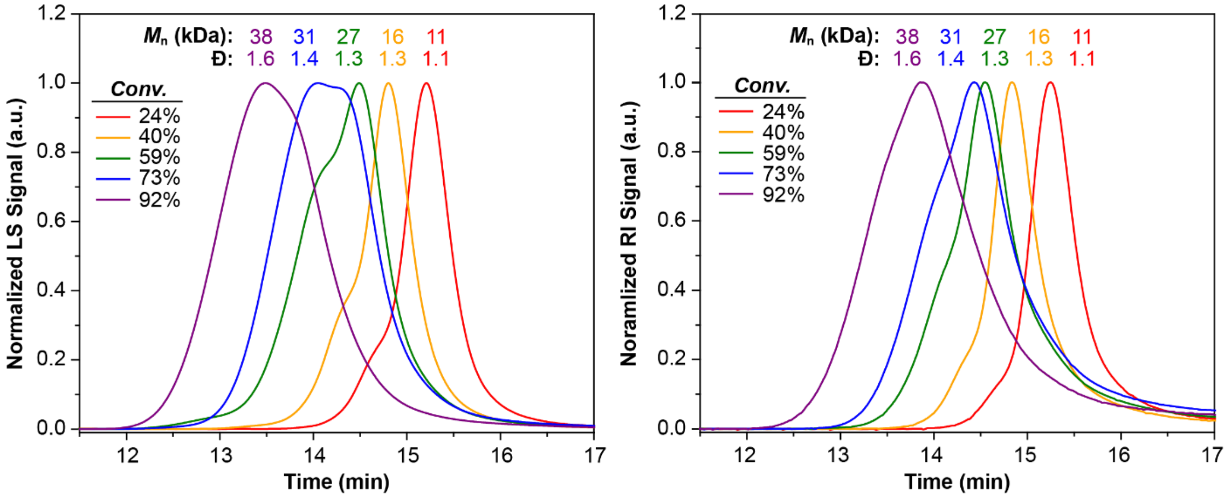

Figure S4. Normalized light-scattering (LS, left) and differential refractive index (dRI, right) data collected via GPC-MALS of aliquots of the polymerization of **M1** ([**M1**]:[**1**] = 200:1) at various conversions of **M1**.

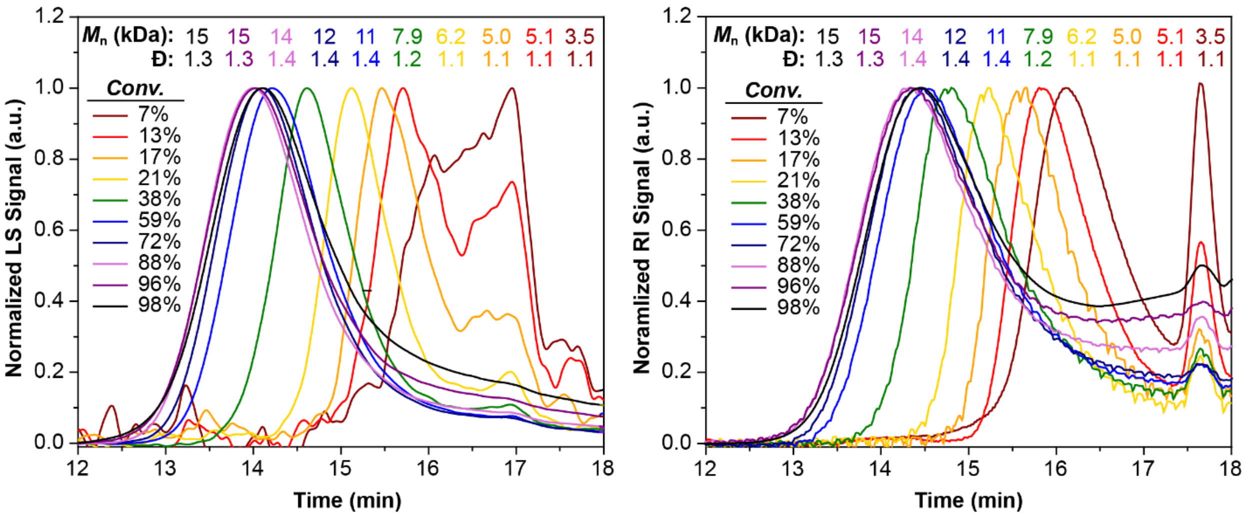

Figure S5. Normalized light-scattering (LS, left) and differential refractive index (dRI, right) data collected via GPC-MALS of aliquots of the polymerization of **M2** ([**M2**]:[**1**] = 200:1) at various conversions of **M2**.

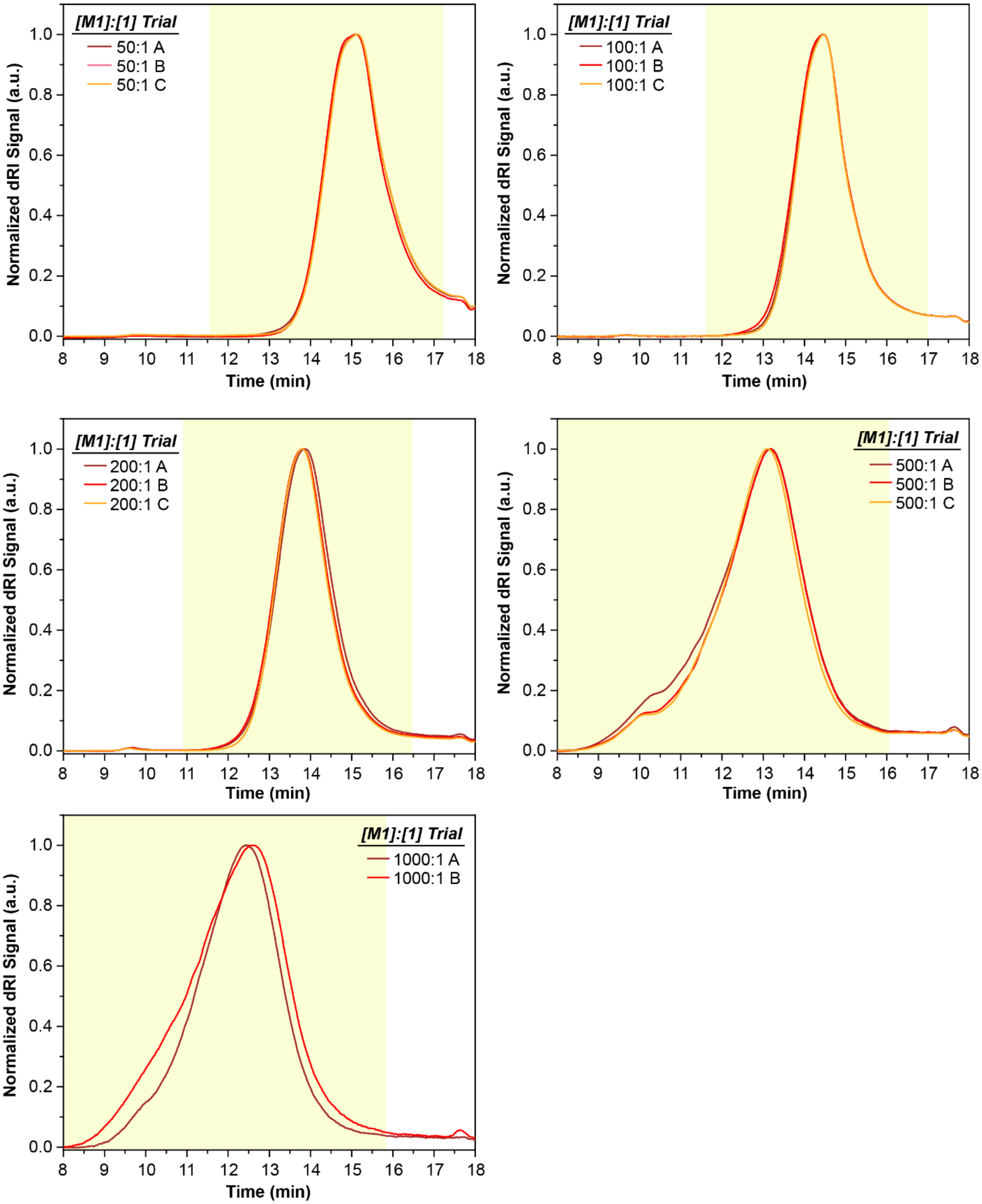

Figure S6. Normalized differential refractive index (dRI) data taken from GPC of triplicate (duplicate for 1000:1) syntheses of **polyM1** at various [**M1**]:[**1**] loadings. Peak-picking windows used for molecular weight calculations are shown as yellow overlays.

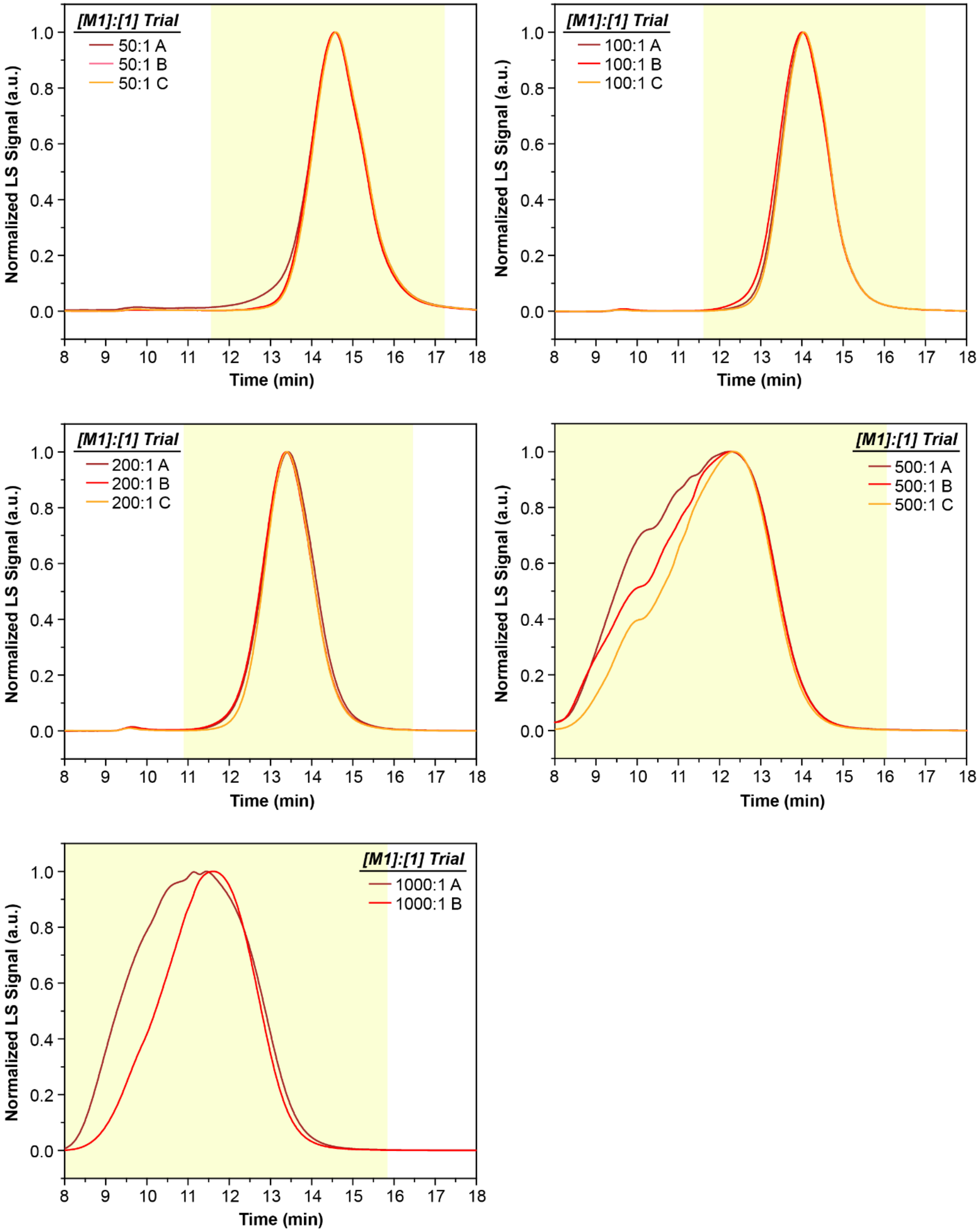

Figure S7. Normalized light-scattering (LS) data taken from GPC of triplicate (duplicate for 1000:1) syntheses of **polyM1** at various [**M1**]:[**1**] loadings. Peak-picking windows used for molecular weight calculations are shown as yellow overlays.

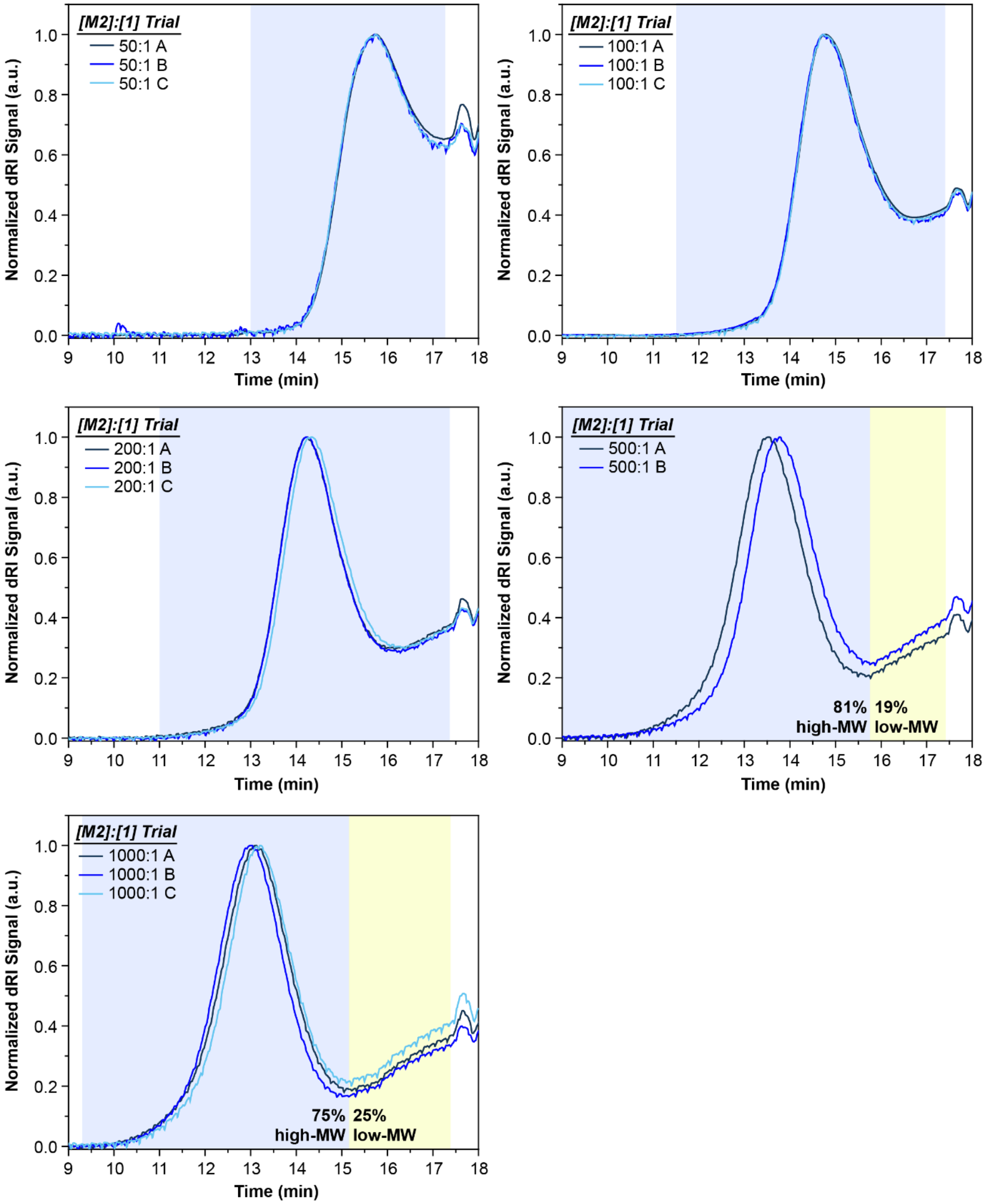

Figure S8. Normalized differential **r**efractive index (dRI) data taken from GPC of triplicate syntheses of **polyM2** at various [**M2**]:[**1**] loadings. Blue overlays are used to indicate integration bounds for molecular weight calculations. For 1000:1 and 500:1 loading, yellow overlays indicate integration bounds for low-MW species that were not included in molecular weight calculations. The corresponding ratios between high- and low-MW species based on the shown integration windows are displayed within each overlay.

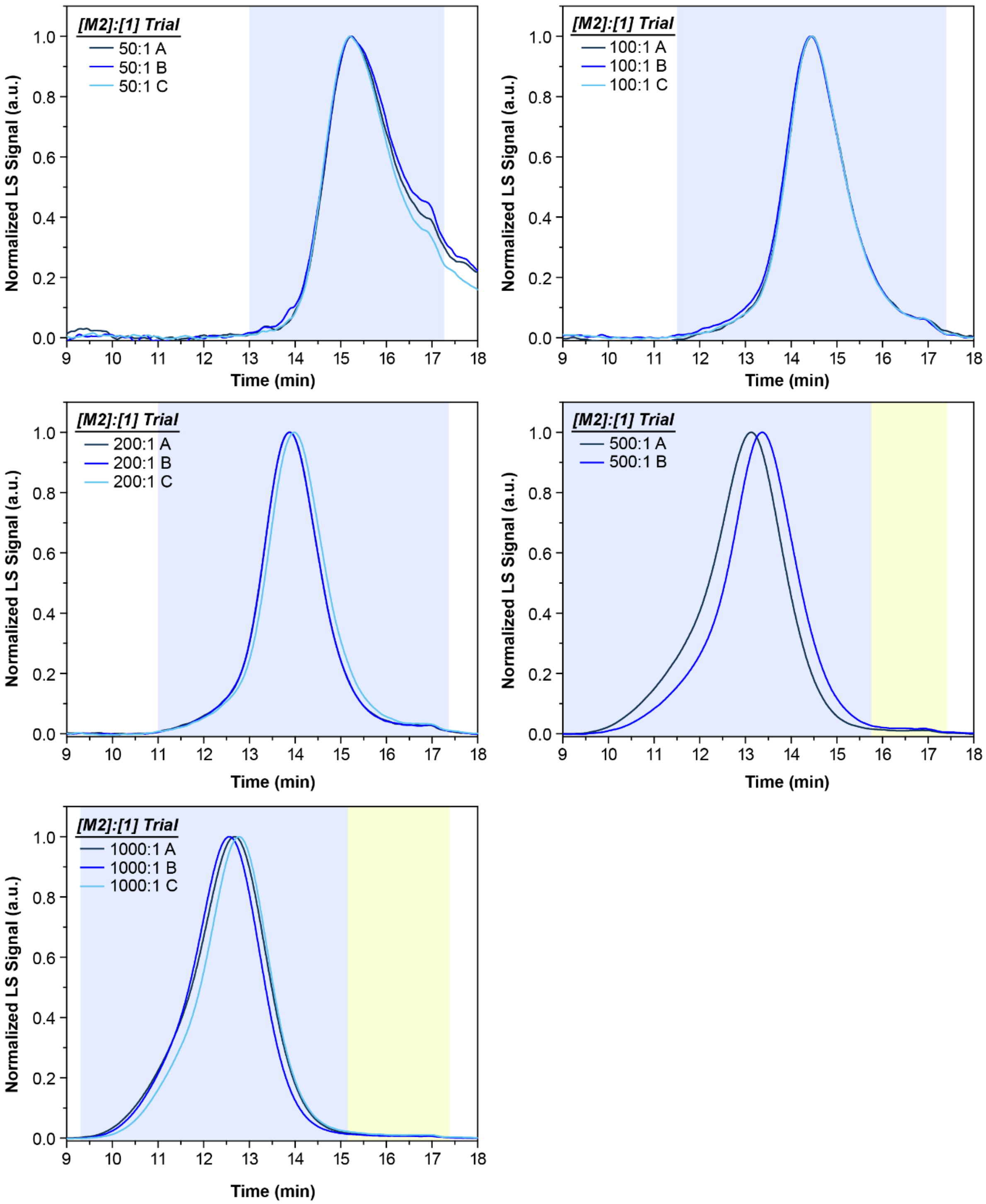

Figure S9. Normalized light-scattering (LS) data taken from GPC analysis of triplicate syntheses of **polyM2** at various [**M2**]:[**1**] loadings. Blue overlays are used to indicate integration bounds for molecular weight calculations. For 1000:1 and 500:1 loading, yellow overlays indicate integration bounds for low-MW species that were not included in molecular weight calculations.

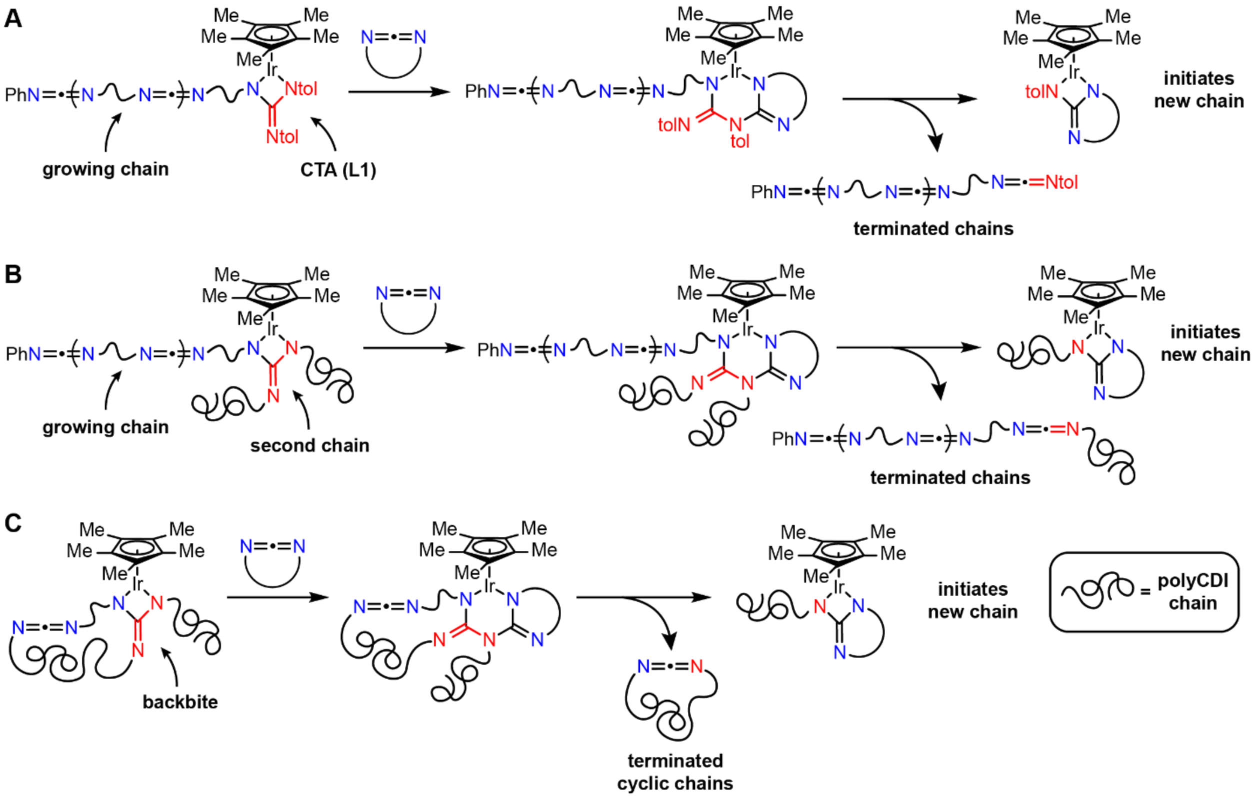

Figure S10. Chain transfer mechanisms for CDI-ROMP from the tri-coordinate four-membered iridocycle, excluding the prerequisite insertion of the chain transfer agent to the propagating chain-end (forming a six-membered iridocycle) and subsequent ring-opening of the previously incorporated monomer. A. Chain transfer arising from reaction of the chain end with **L1**. B. Intermolecular chain transfer with another growing or previously terminated polymer chain. C. Chain transfer arising from back-biting events. tol = *p*-MeC_6H_4

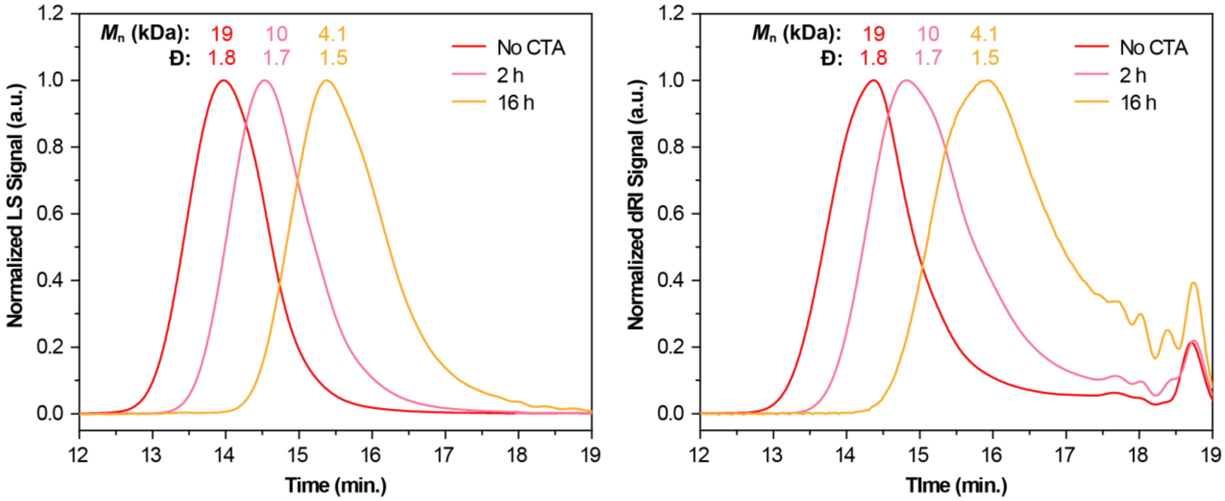

Figure S11. Normalized light-scattering (LS, left) and differential refractive index (dRI, right) data collected via GPC-MALS before and at several time points after the treatment of a non-terminated sample of polyM1 with five equivalents (relative to initiator) of di-*p*-tolyl CDI (L1) at 23 °C.

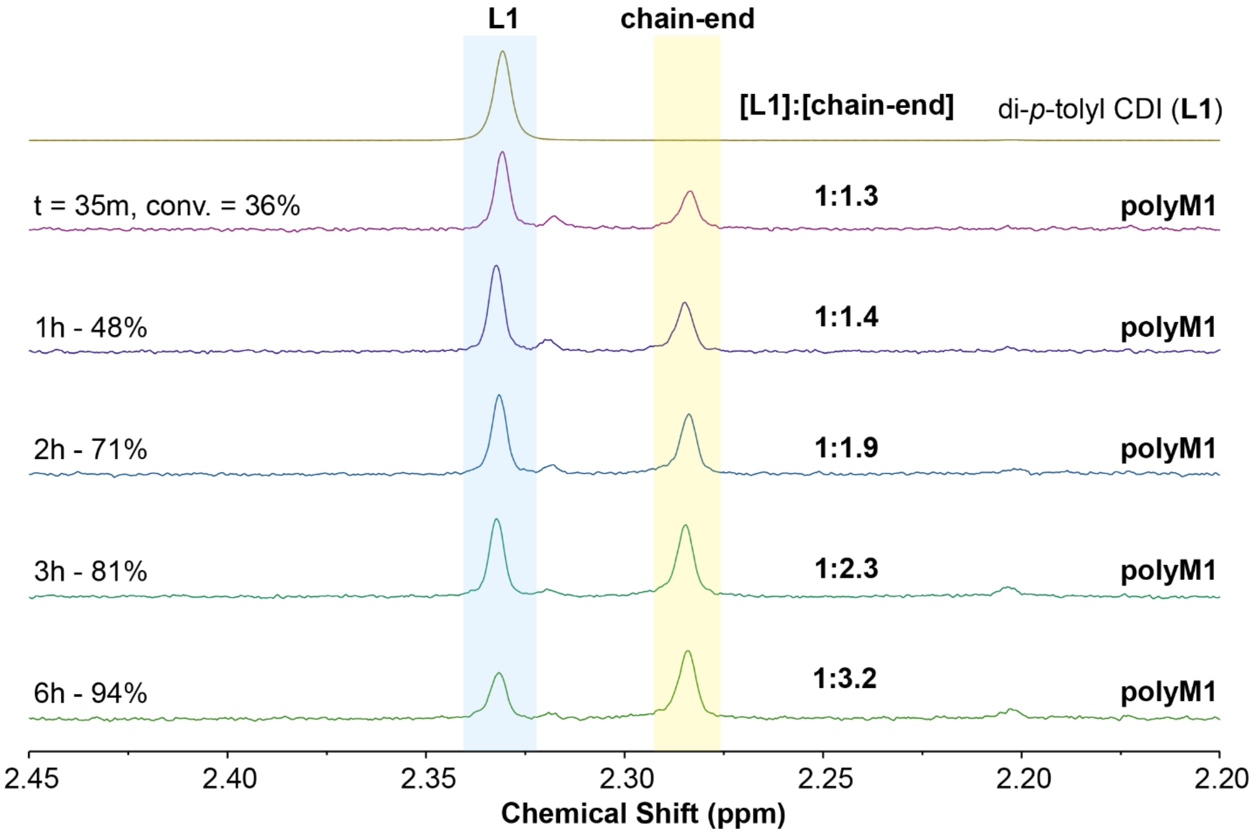

Figure S12. Stacked ^{1}H NMR (500 MHz, $CDCl_3$, 23 °C) spectra comparison of crude polymerization mixtures of a 100:1 [**M1**]:[**1**] experiment (at various times and conversions, listed left) and di-*p*-tolyl CDI **L1** (the chain-transfer agent). Molar ratios between **L1** and the chain-end at each time point are listed in the center.

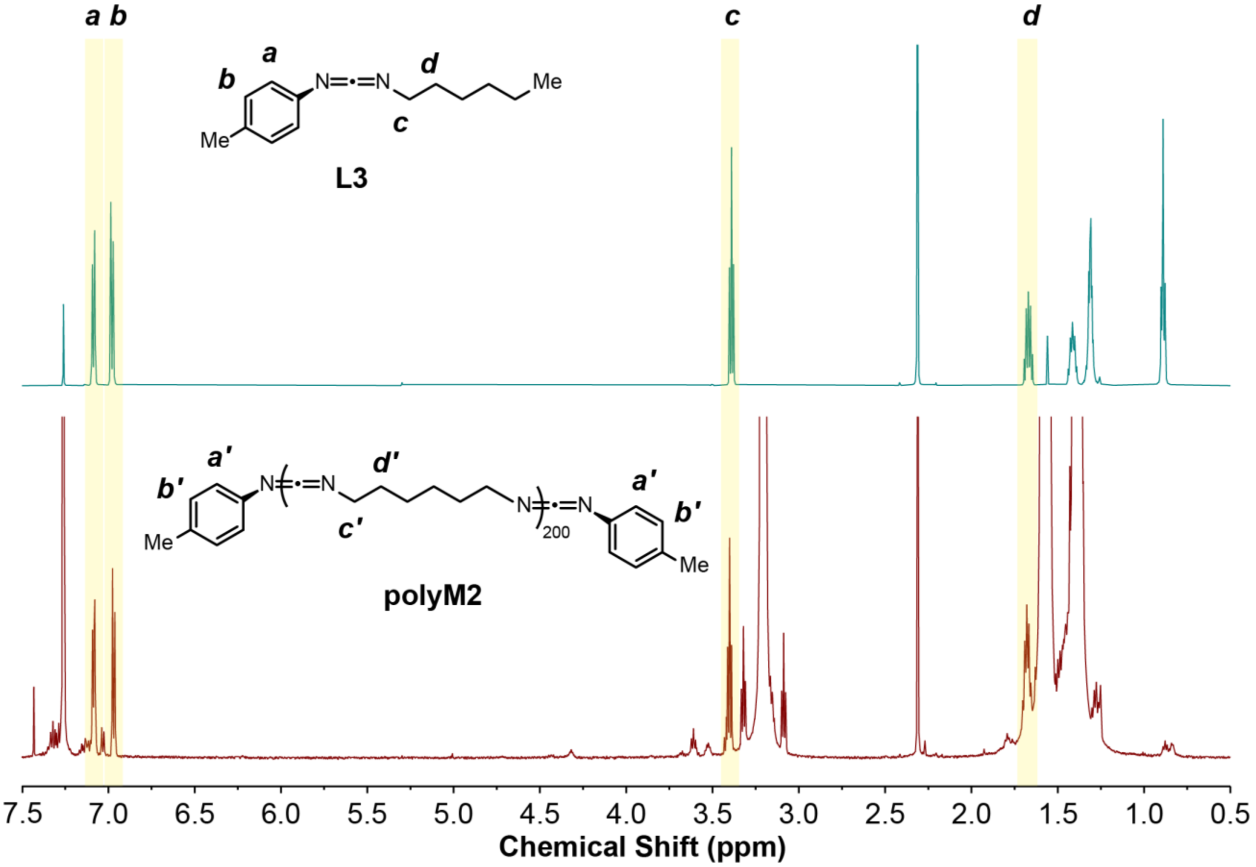

Figure S13. Stacked ^{1}H NMR (600 MHz, $CDCl_3$, 23 °C) spectra of chain-end model compound *N*-hexyl-*N'*-(*p*-tolyl)carbodiimide (**L3**, top) and a precipitated sample of **polyM2** (DP = 200, bottom).

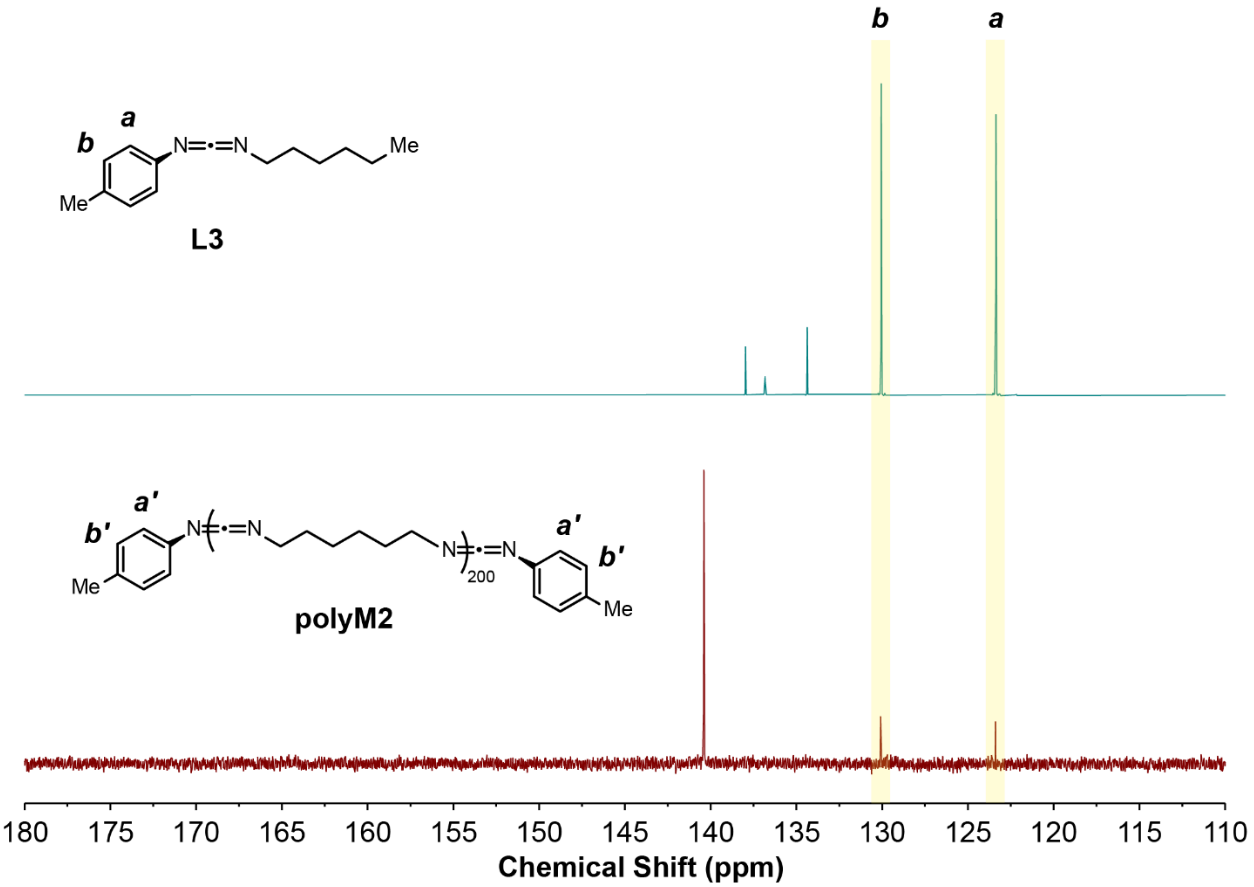

Figure S14. Stacked ^{13}C NMR (150 MHz, $CDCl_3$, 23 °C) spectra comparison of chain-end model compound N-*p*-tolyl-N'-hexylcarbodiimide (**L3**, top) and a precipitated sample of **polyM2** (DP = 200, bottom).

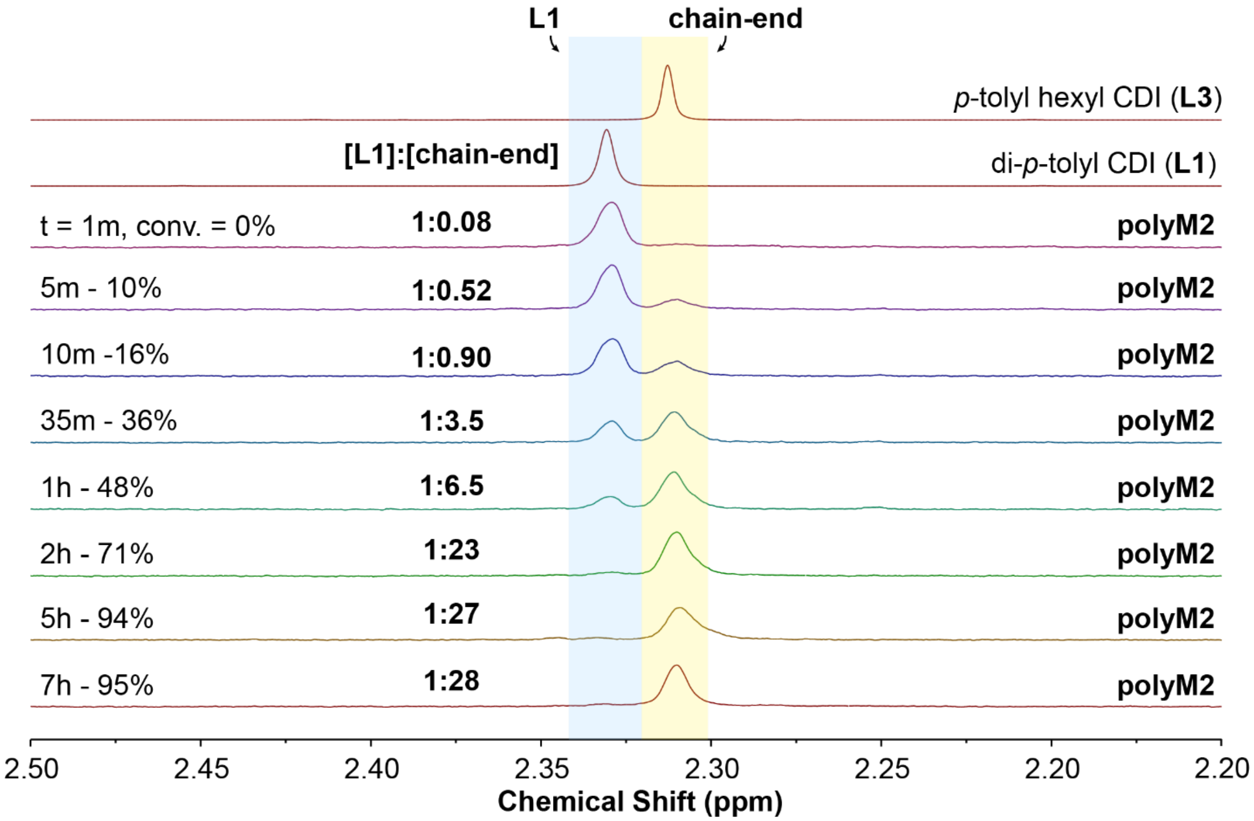

Figure S15. Stacked ^{1}H NMR (500 MHz, $CDCl_3$, 23 °C) spectra comparison of crude polymerization mixtures of a 100:1 [**M2**]:[**1**] experiment (at various times and conversions, listed left), chain-end model compound N-*p*-tolyl-N'-hexylcarbodiimide **L3**, and di-*p*-tolyl carbodiimide **L1** (the chain-transfer agent). Molar ratios between **L1** and the chain-end at each time point are listed center.

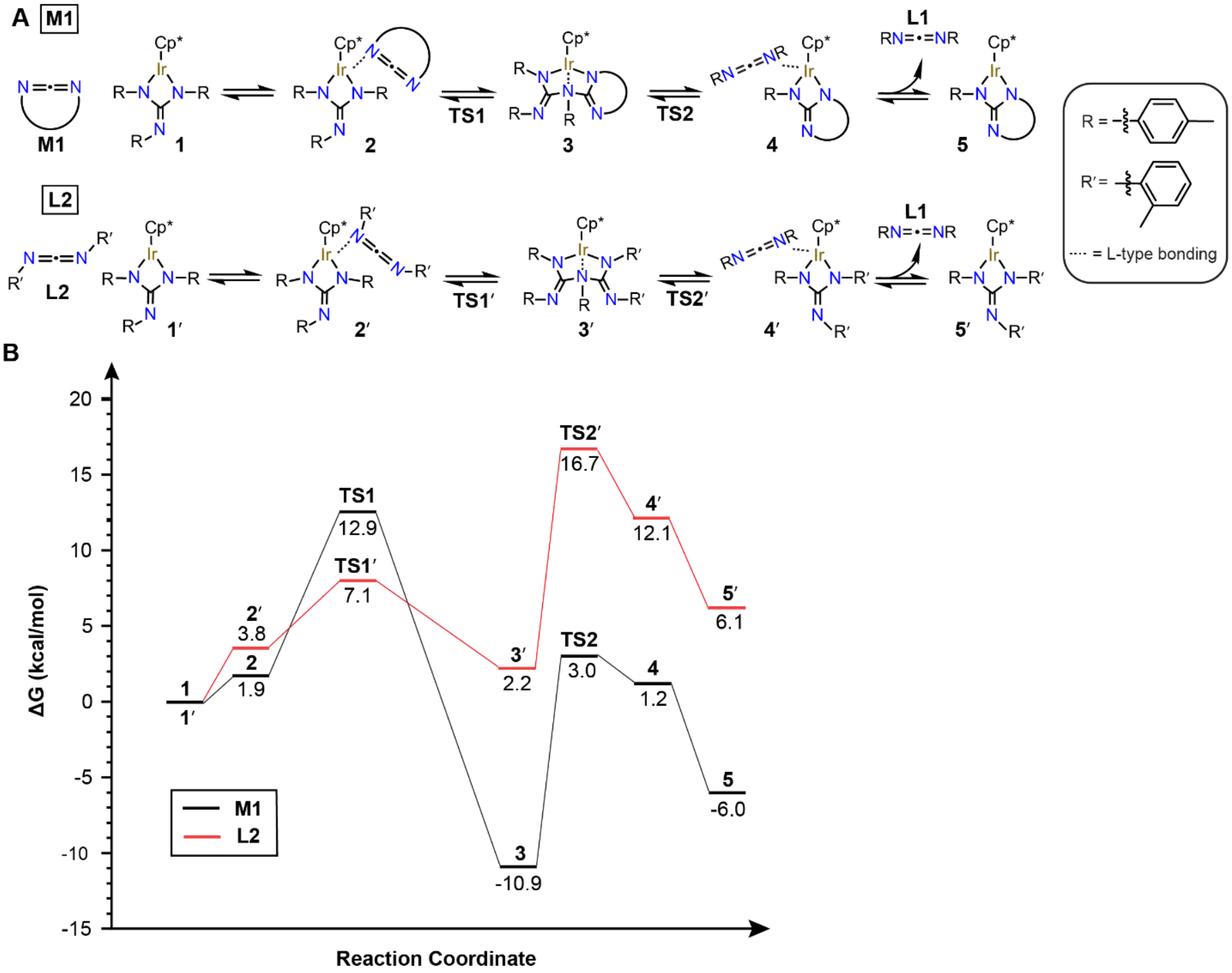

Figure S16. A. The proposed mechanisms for initiation of CDI-ROMP for **M1** and **L2**. B. Reaction coordinate diagram with calculated energy levels of intermediates and transition states for **M1** and **L2**.

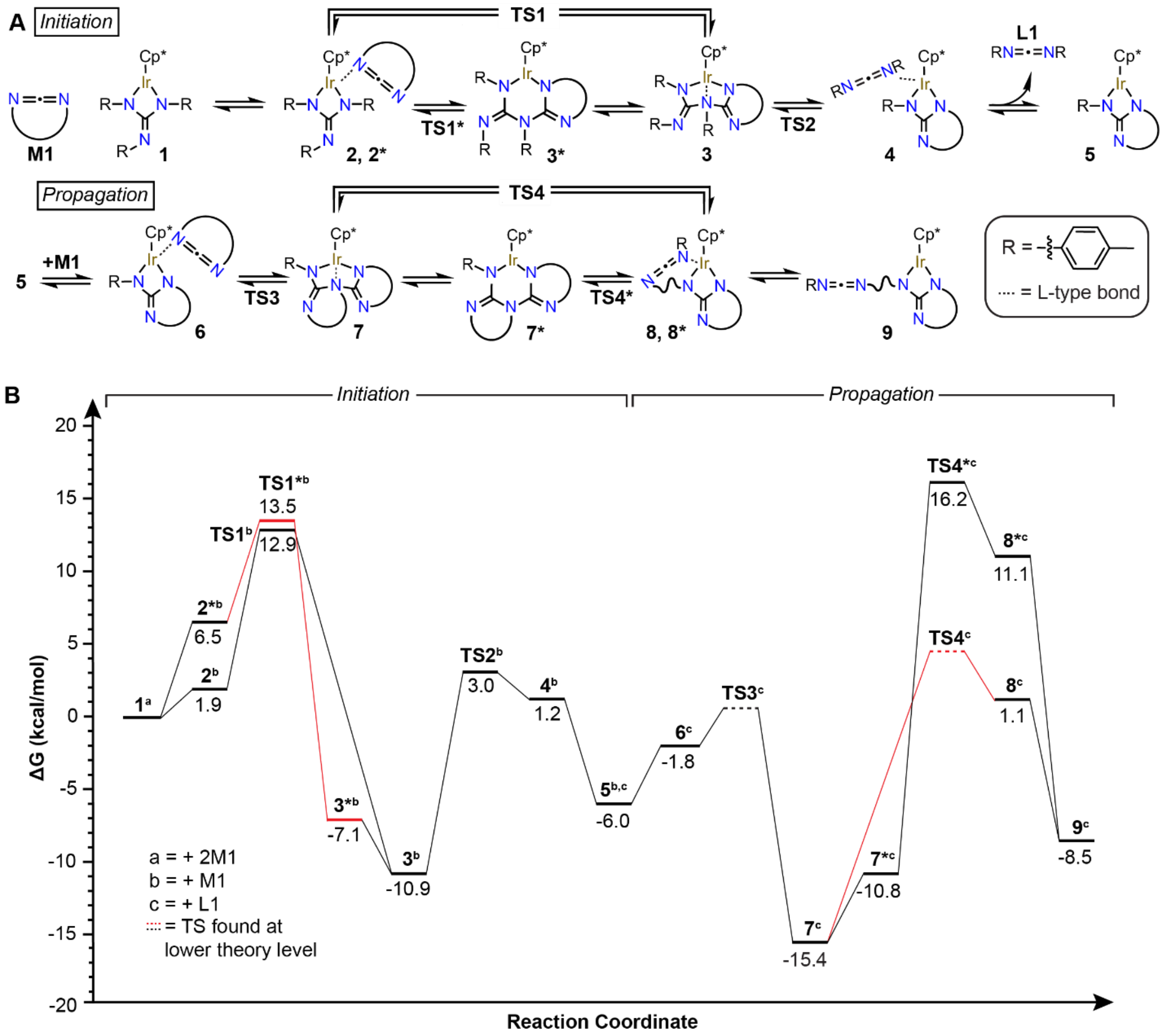

Figure S17. A. The proposed mechanism for CDI-ROMP, with additional intermediates **3*** and **7*** included for energy level comparisons to **3** and **7**. **2*** and **8*** are higher-energy conformations corresponding to the *syn* geometry of the inserted **M1** unit (see Computational SI for additional information). B. Reaction coordinate diagram with calculated energy levels of intermediates and transition states for CDI-ROMP, including higher-energy intermediates **3*** and **7***.

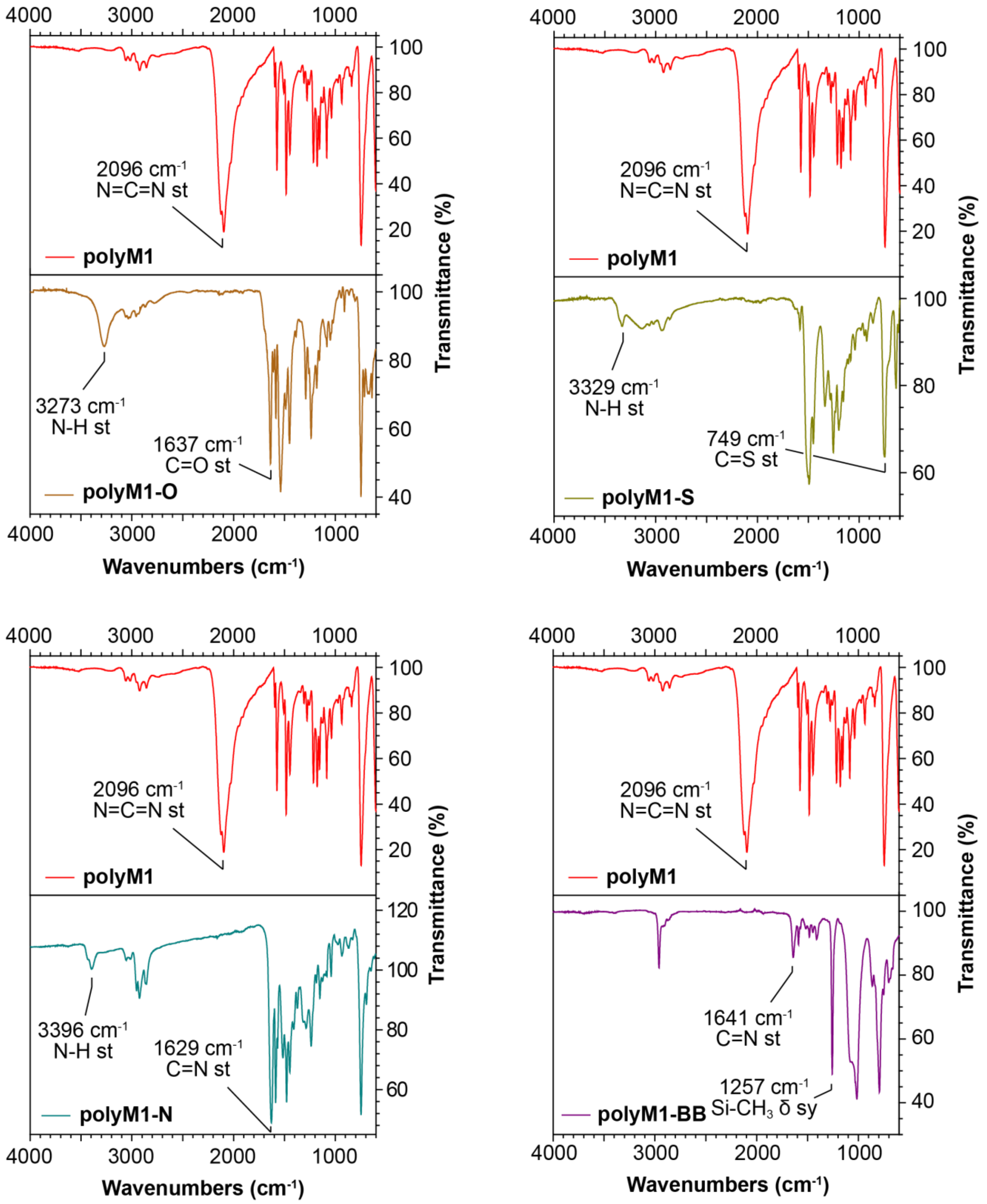

Figure S18. Stacked IR spectra of **polyM1** compared to its derivatives, with notable features labeled with lines.

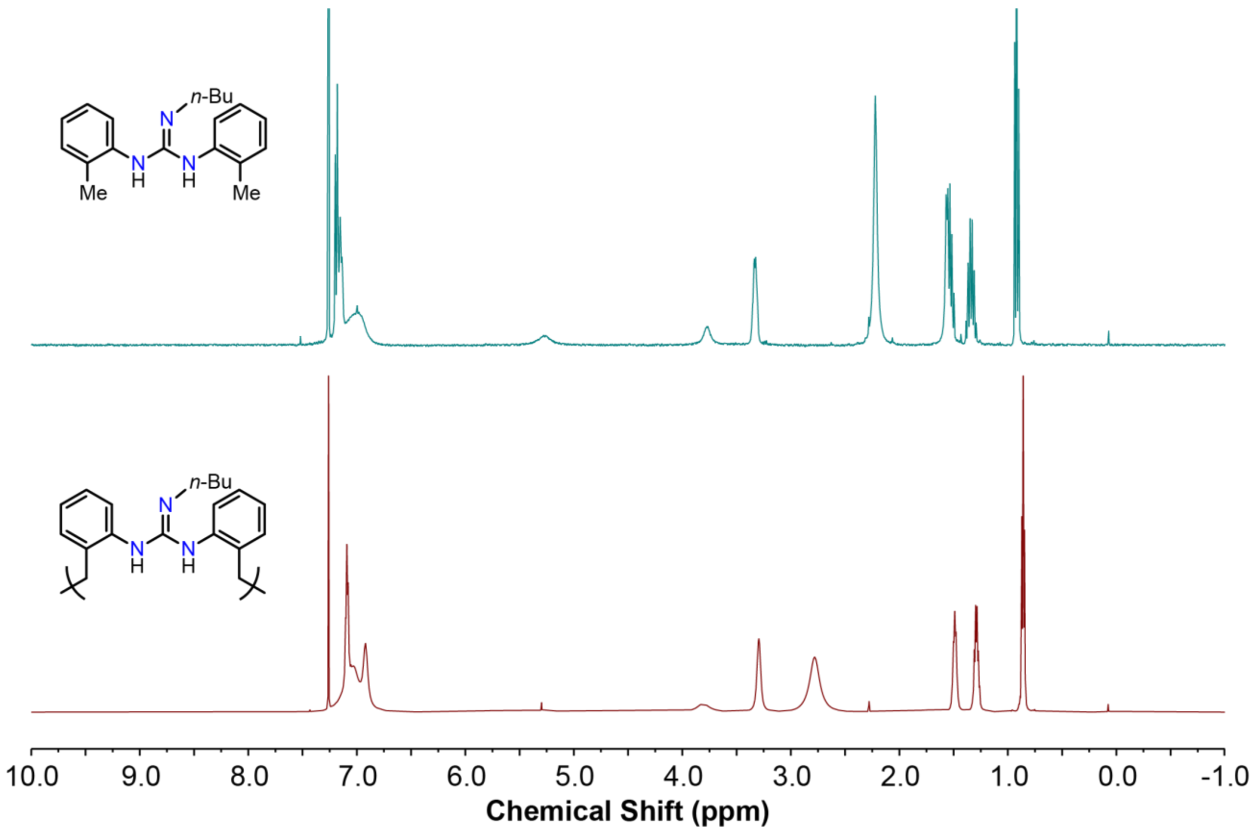

Figure S19. Stacked ^{1}H NMR (400 MHz, $CDCl_3$, 23 °C) spectra of **polyM1-N** and its small molecule model **L2-N**.

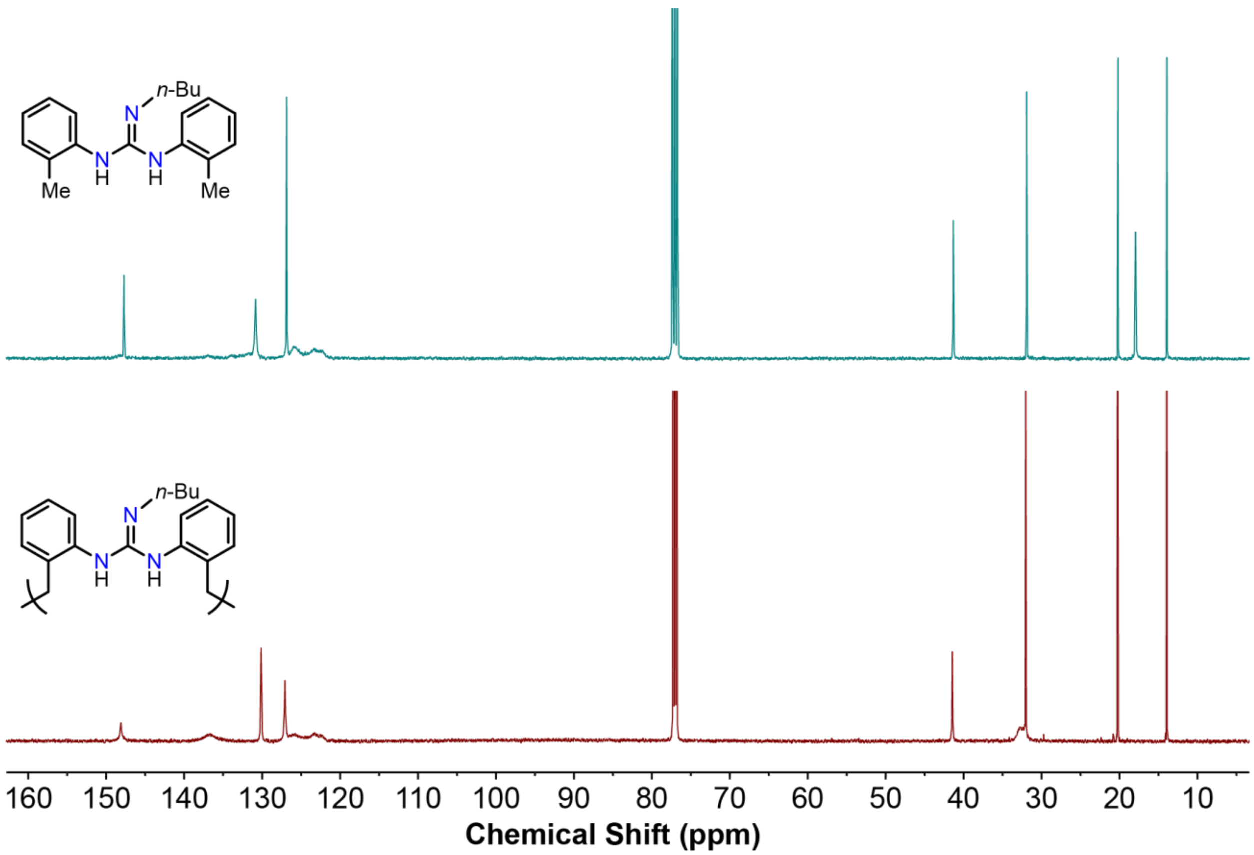

Figure S20. Stacked ^{13}C NMR (100 MHz, $CDCl_3$, 23 °C) spectra of **polyM1-N** and its small molecule model **L2-N**.

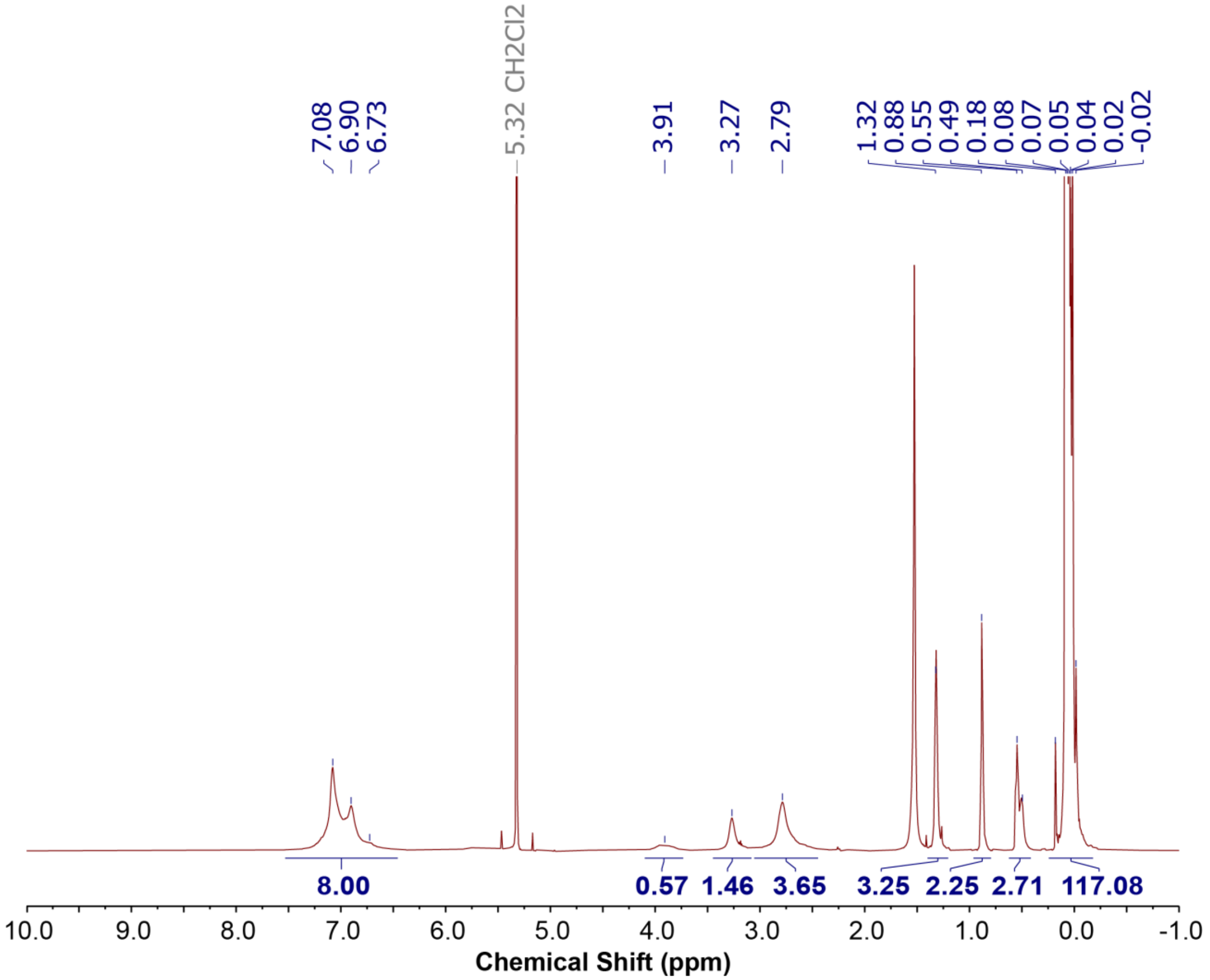

Figure S21. ^{1}H NMR (400 MHz, $CDCl_3$, 23 °C) spectrum of **polyM1-BB**.

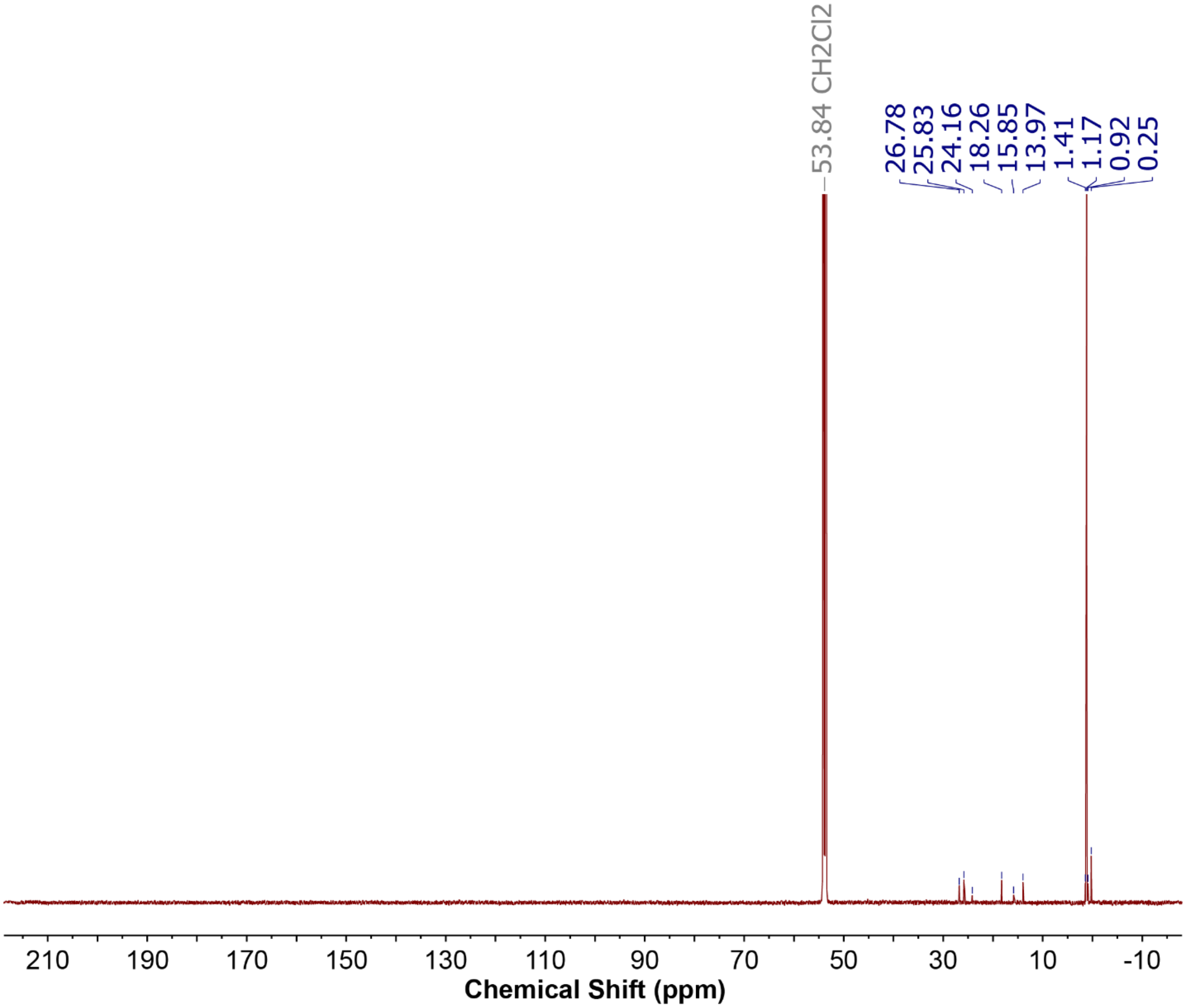

Figure S22. ^{13}C NMR (600 MHz, $CDCl_3$, 23 °C) spectrum of **polyM1-BB**.

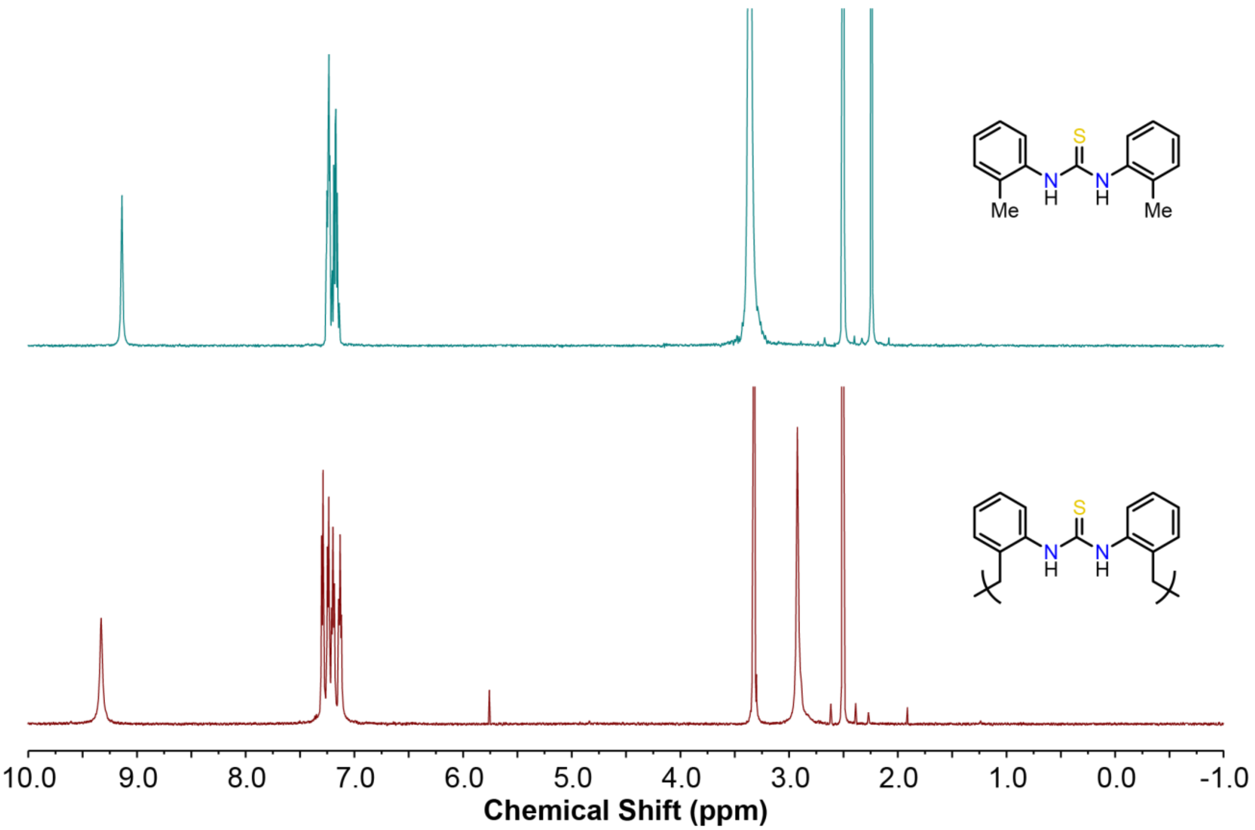

Figure S23. Stacked ^{1}H NMR (500 MHz, DMSO-d_6, 23 °C) spectra of **polyM1-S** and its small molecule model **L2-S**.

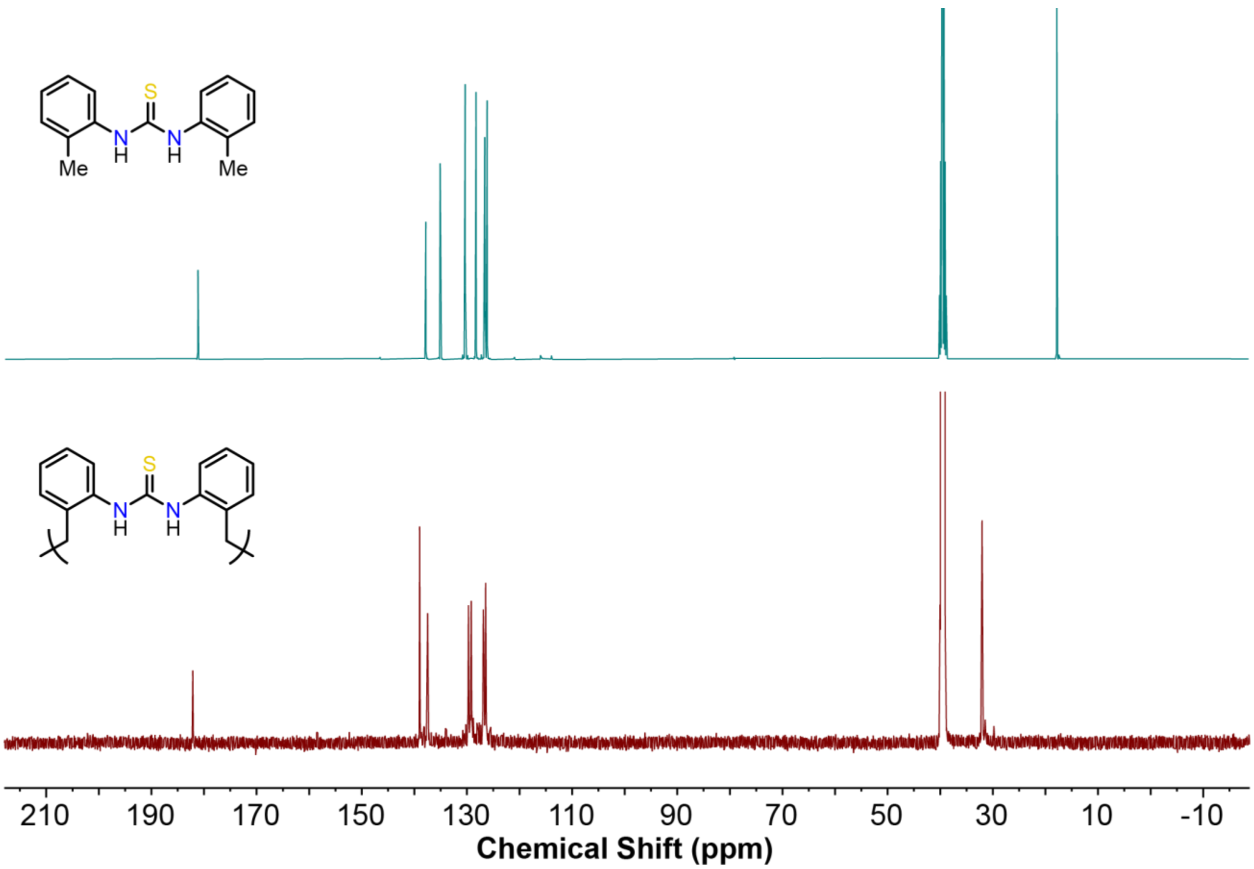

Figure S24. Stacked ^{13}C NMR (100 MHz, DMSO-d_6, 23 °C) spectra of **polyM1-S** and its small molecule model **L2-S**.

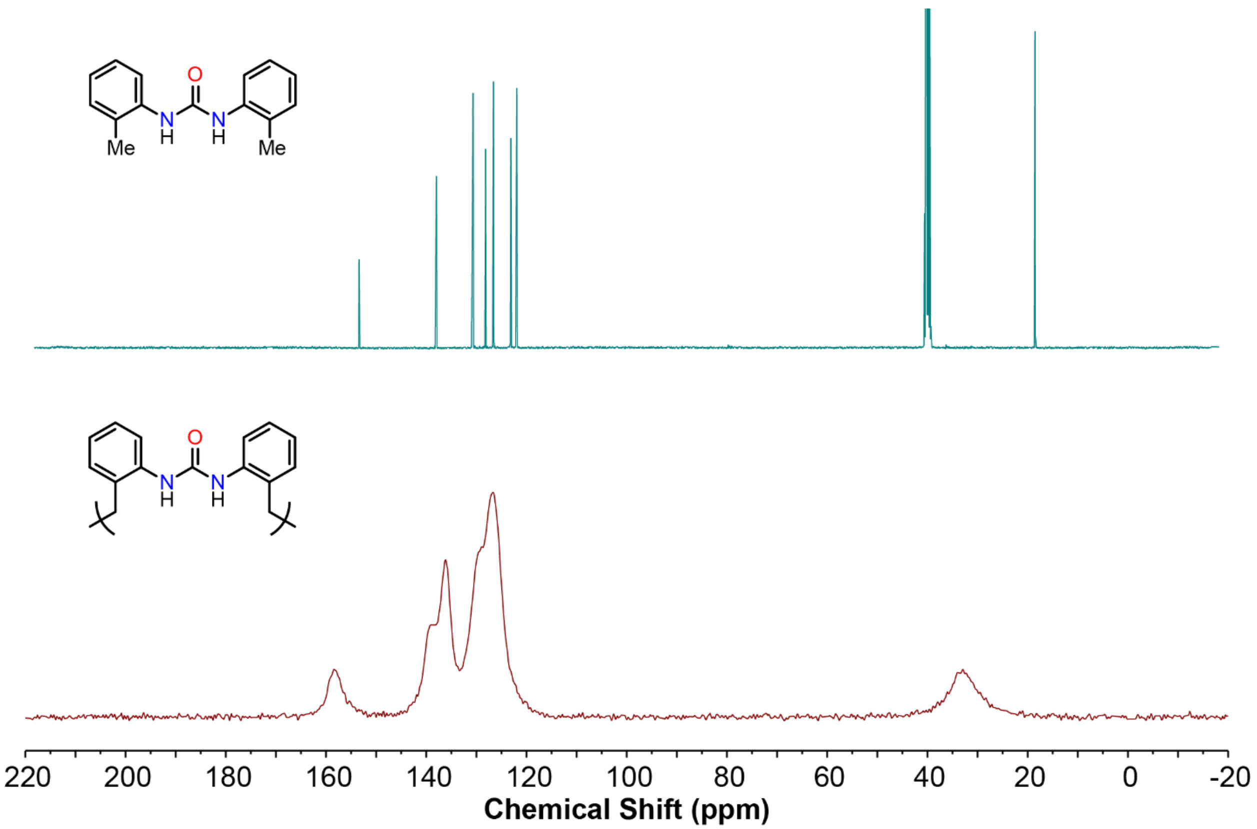

Figure S25. Stacked ^{13}C NMR (**L2-O**: 100 MHz, DMSO-d_6, 23 °C, **polyM1-O**: ssNMR CP/MAS, 75 MHz, 12 kHz) spectra of **polyM1-O** and its small molecule model **L2-O**.

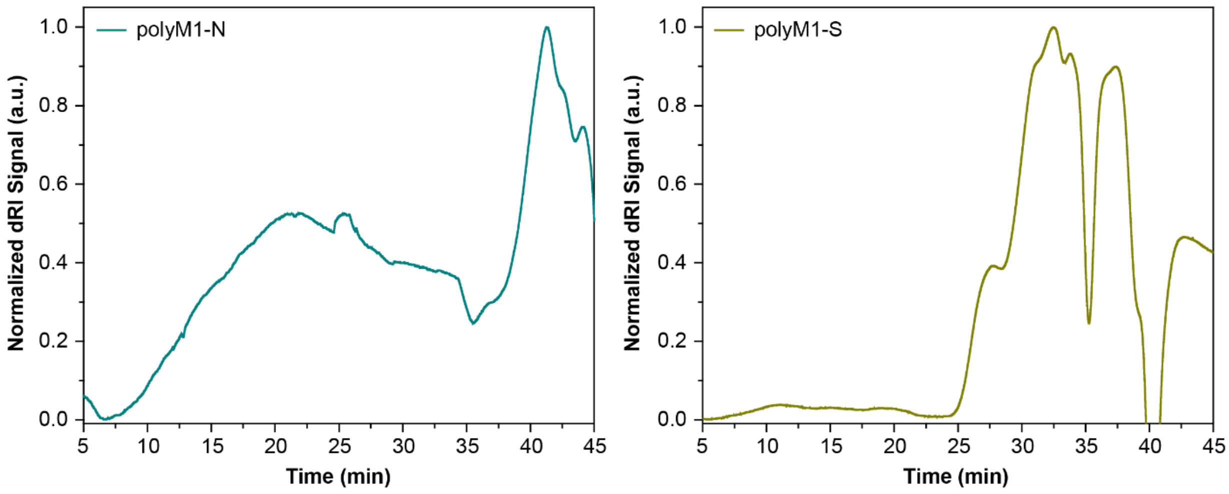

Figure S26. Normalized differential refractive index (dRI) data collected via GPC-MALS-DMF of **polyM1-N** (left) and **polyM1-S** (right) in 0.025 M LiBr in DMF.

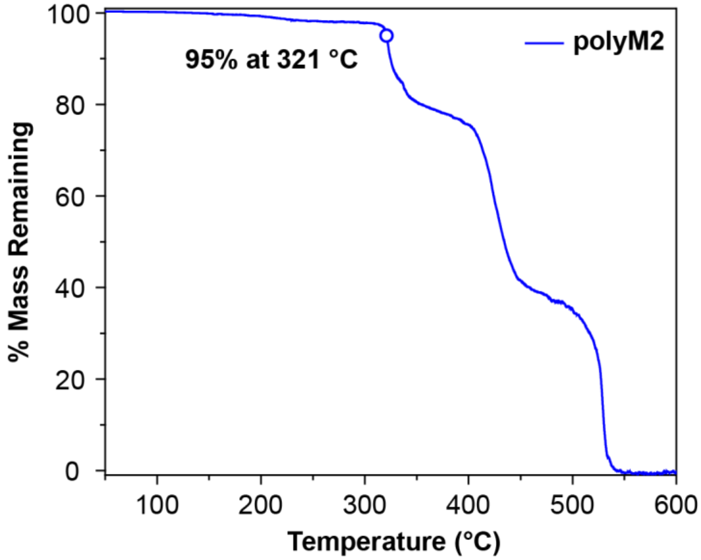

Figure S27. TGA data of **polyM2** (10 °C/min), with 5% mass loss temperature noted as a circle.

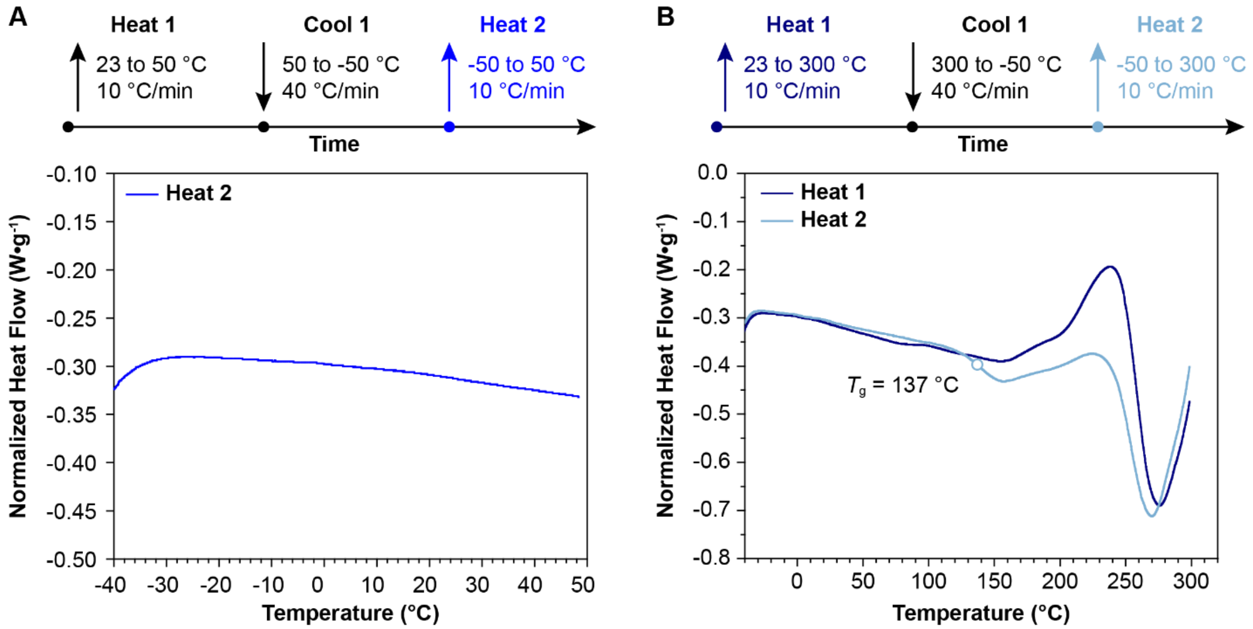

Figure S28. DSC data (bottom) with the corresponding heating/cooling profile (above) for **polyM2** at different maximum temperatures. A. DSC data for heating **polyM2** to temperatures lower than the first observed exotherm. B. DSC data for heating **polyM2** to approx. 20 °C below (300 °C) its 5% decomposition temperature.

SUPPLEMENTARY TABLES

Table S1. M_n, DP, and Đ as measured by GPC-MALS for various [**M1**]:[**1**] loadings in the polymerization of **M1**.

[M1]:[1]	Trial #	M_n (kg/mol)	DP	Đ
50:1	1	8.4	37	1.92
	2	9.0	40	1.79
	3	9.0	40	1.71
	Avg	8.8	39	1.81
100:1	1	18.4	83	1.69
	2	18.7	84	1.74
	3	17.2	77	1.77
	Avg	18.1	81	1.73
200:1	1	34.9	157	1.81
	2	38.8	175	1.72
	3	38.9	176	1.68
	Avg	37.5	169	1.74
500:1	1	83.7	379	2.84
	2	78.6	356	2.94
	3	85.2	386	2.56
	Avg	82.5	374	2.78
1000:1	1	148	671	2.60
	2	176	799	2.17
	Avg	162	735	2.38

Table S2. M_n, DP, and Đ as measured by GPC-MALS for various [M1]:[1] loadings in the polymerization of **M2**.

[M2]:[1]	Trial #	M_n (kg/mol)	DP	Đ
50:1	1	4.8	37	1.16
	2	5.8	45	1.14
	3	4.8	37	1.21
	Avg	5.1	40	1.17
100:1	1	8.3	65	1.42
	2	8.6	68	1.47
	3	7.5	59	1.56
	Avg	8.1	64	1.48
200:1	1	12.8	101	1.73
	2	13.1	104	1.74
	3	12.7	101	1.63
	Avg	12.9	102	1.70
500:1	1	36.0	288	1.88
	2	29.7	238	1.66
	Avg	32.9	263	1.77
1000:1	1	50.1	402	1.96
	2	58.1	466	1.82
	3	40.8	327	1.90
	Avg	49.7	398	1.89

GPC CHARACTERIZATION

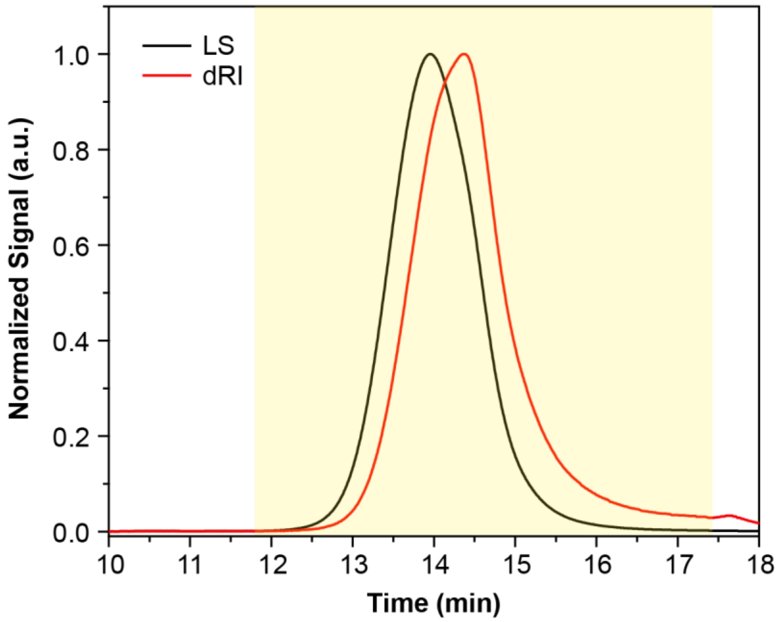

Figure S29. Normalized light-scattering (LS), differential refractive index (dRI), and the peak-picking window (yellow overlay) used to determine the *dn/dc* of **polyM1**, assuming 100% mass recovery of a purified sample at a concentration of 3.0 mg/mL.

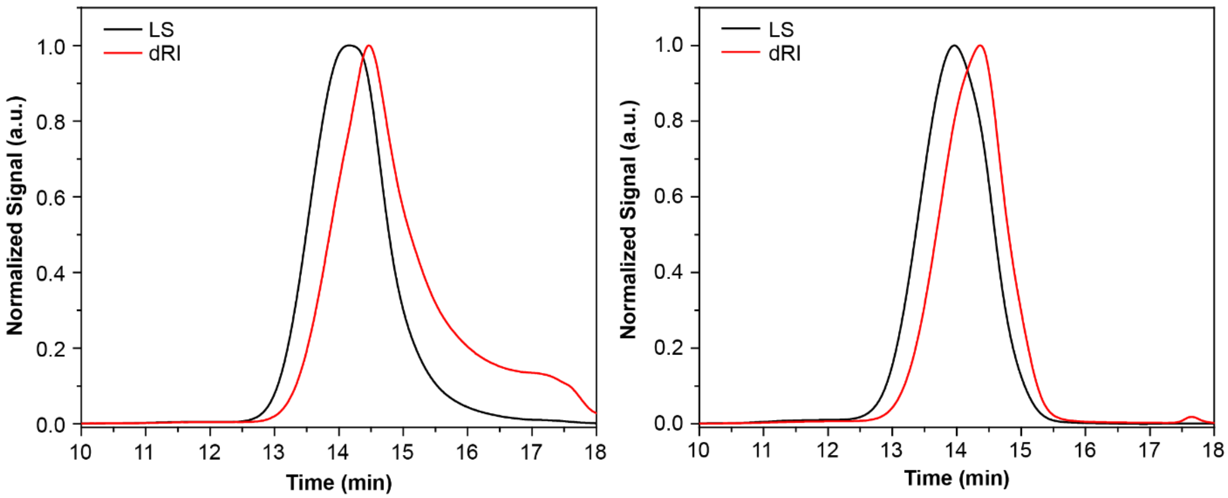

Figure S30. Normalized light-scattering (LS) and differential refractive index (dRI) data from GPC of a 100:1 [**M1**]:[**1**] polymerization after precipitation into cold MeOH (left) and following purification via prep-GPC (right).

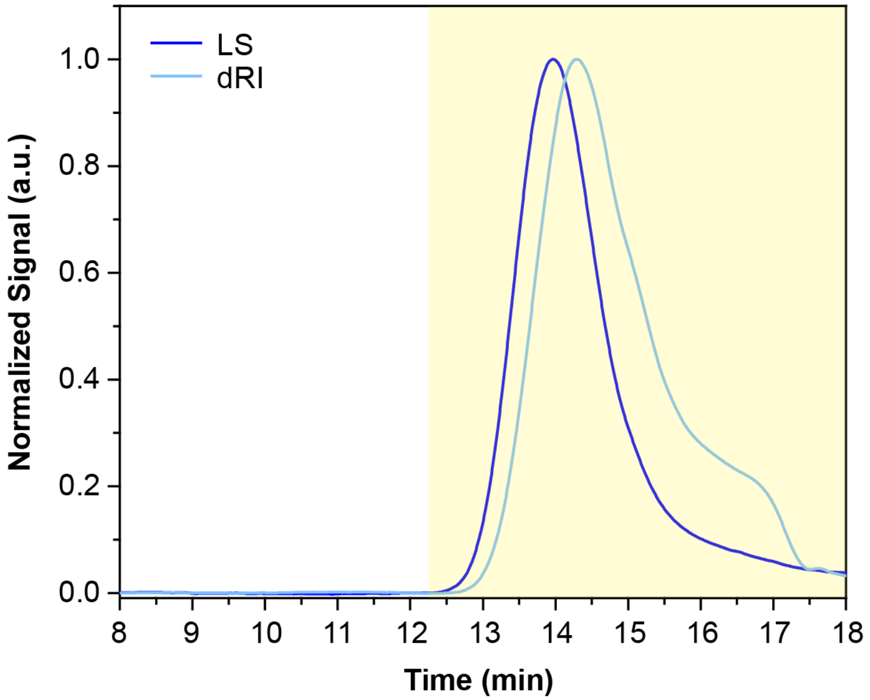

Figure S31. Normalized light-scattering (LS), differential refractive index (dRI), and the peak-picking window (yellow overlay) used to determine the *dn/dc* of **polyM2**, assuming 100% mass recovery of a purified sample at a concentration of 3.0 mg/mL.

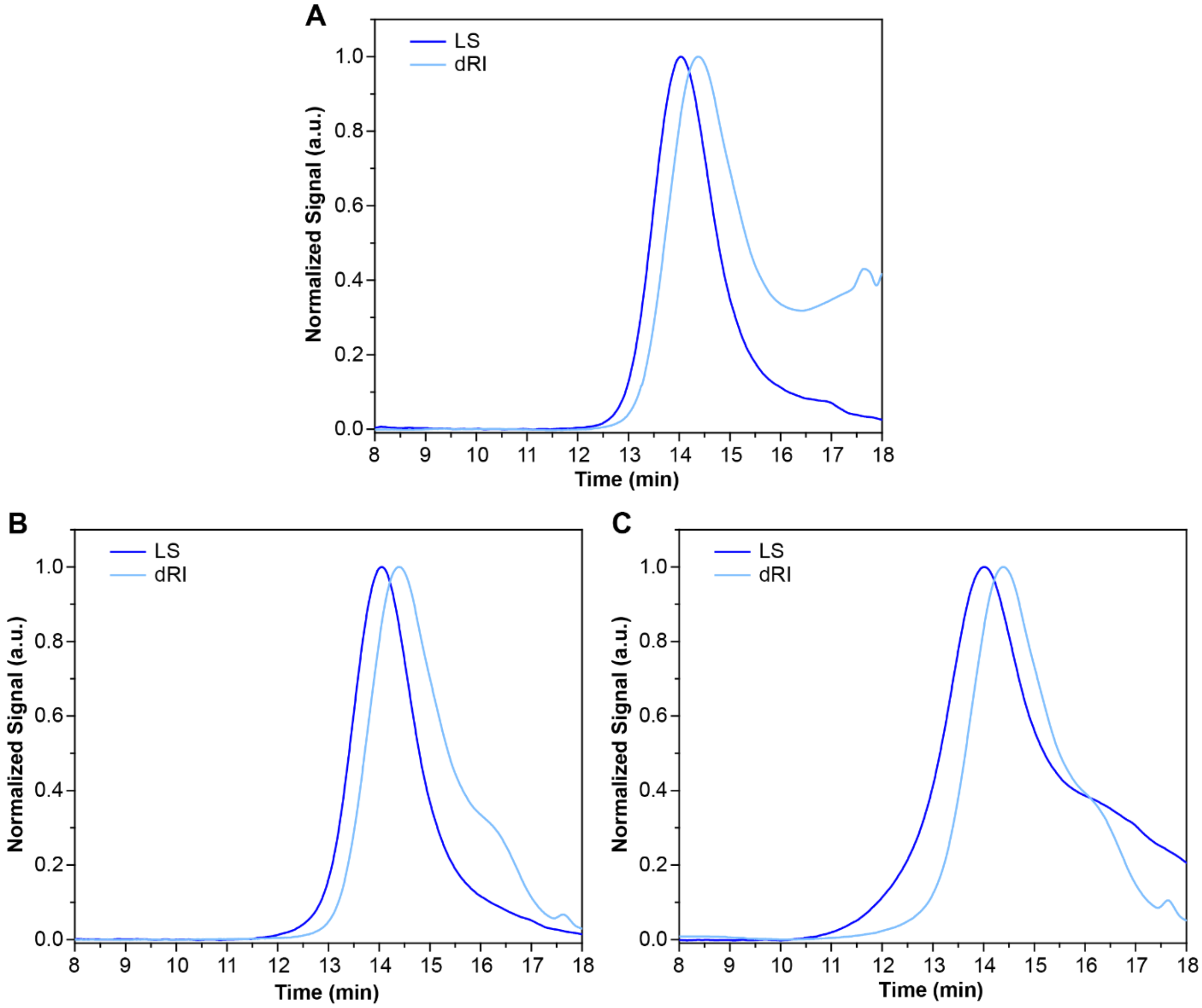

Figure S32. Normalized light-scattering (LS) and differential refractive index (dRI) data from GPC of a 200:1 [**M2**]:[**1**] polymerization. A. The crude material upon reaching 98% conversion of **M2**. B. The material following precipitation into hexanes and centrifugation (10 min, 4000 rpm). C. The purified material after being dried overnight at 23 °C at 50 mTorr.

SPECTRAL CHARACTERIZATION

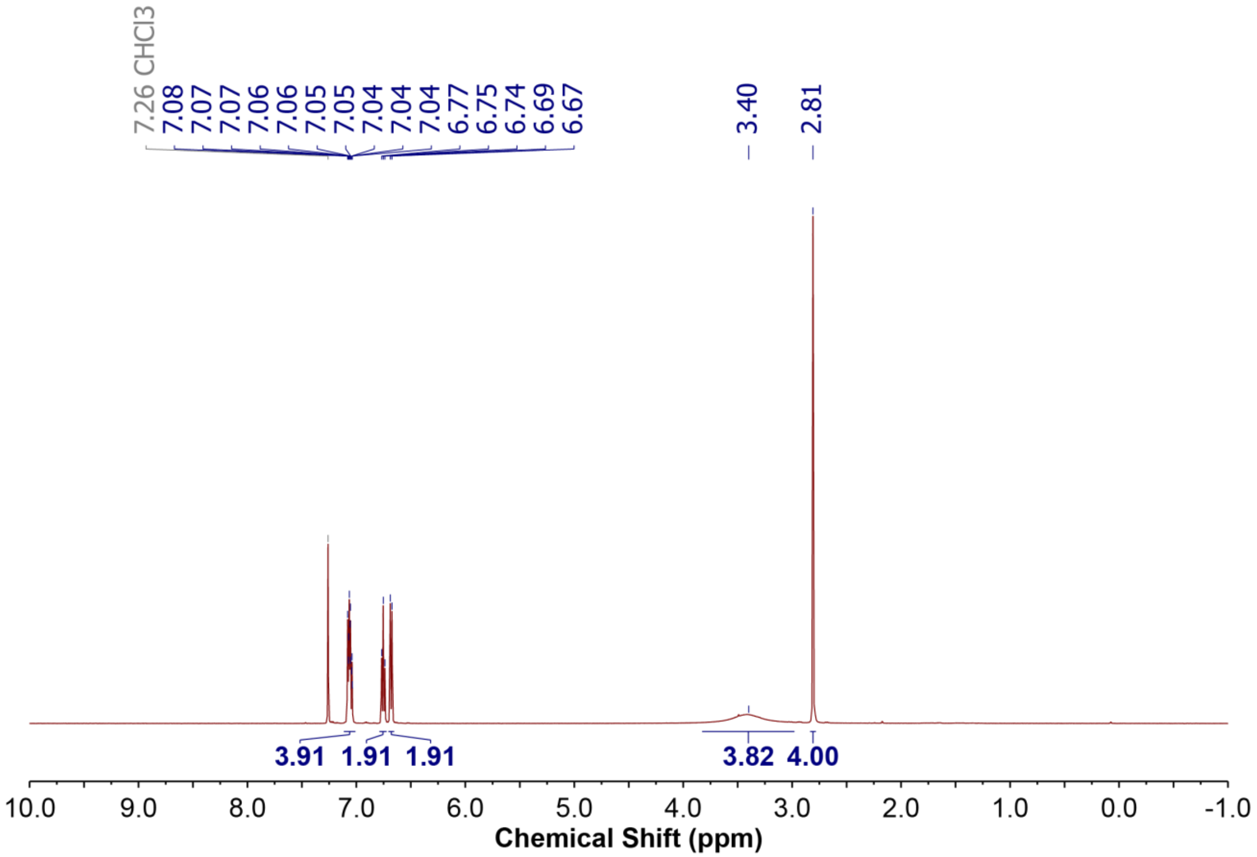

Figure S33. ^{1}H NMR (500 MHz, $CDCl_3$, 23 °C) spectrum of **S1**.

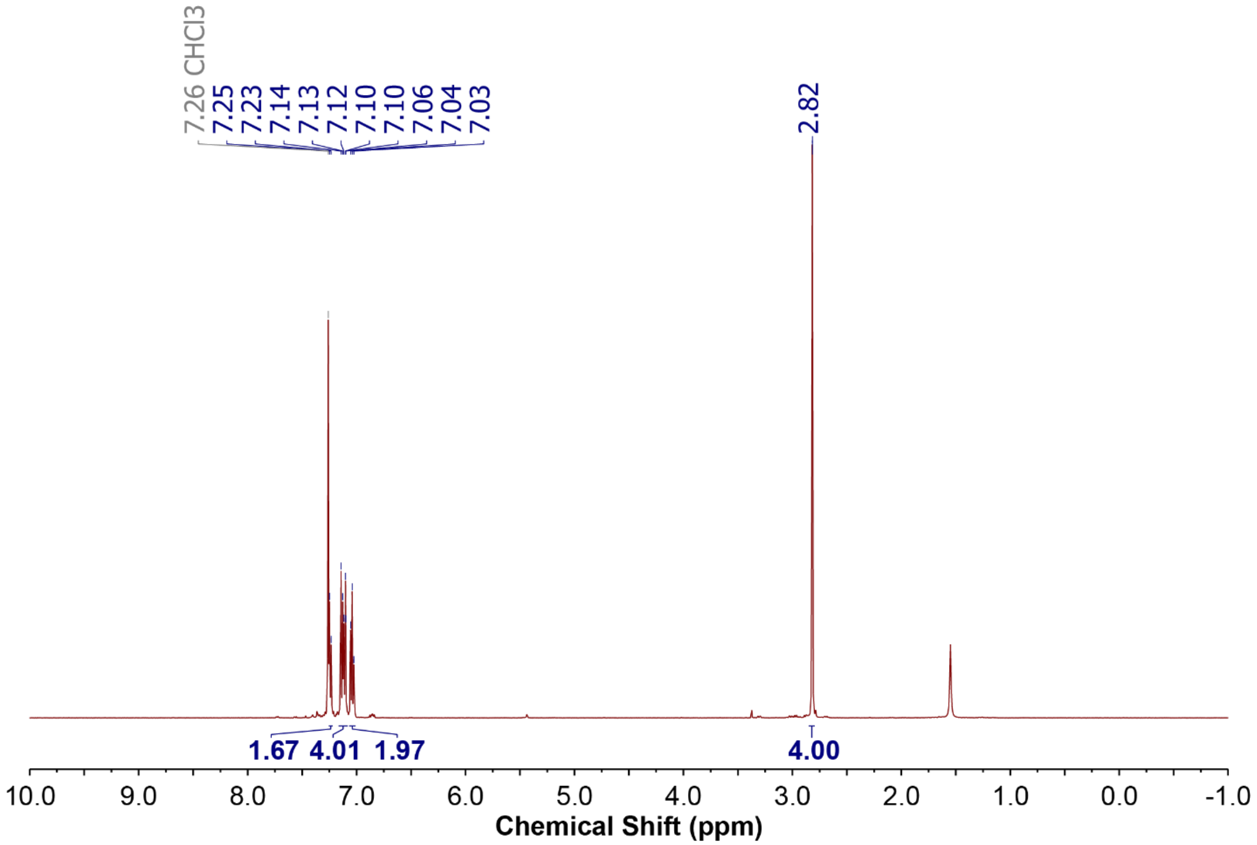

Figure S34. ^{1}H NMR (500 MHz, $CDCl_3$, 23 °C) spectrum of **S2**.

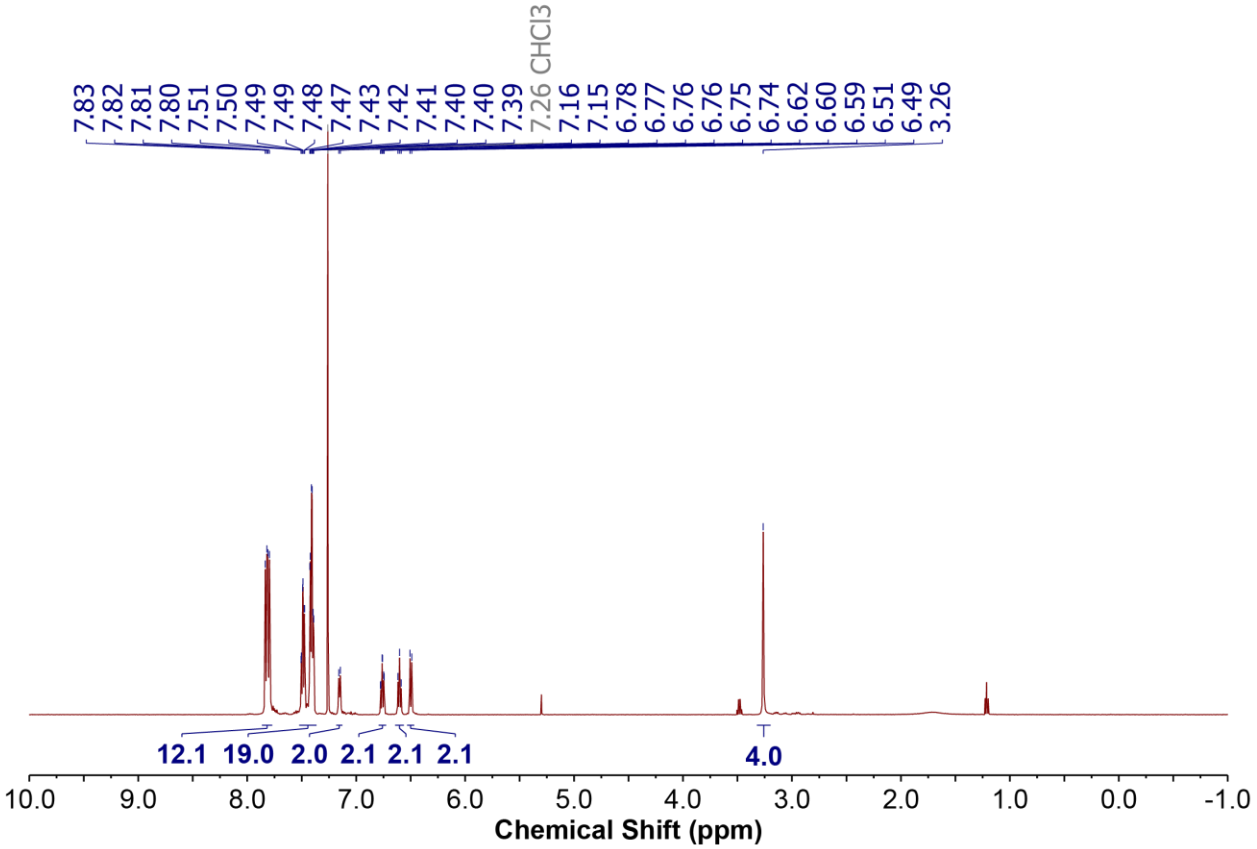

Figure S35. ^{1}H NMR (500 MHz, $CDCl_3$, 23 °C) spectrum of **S3**.

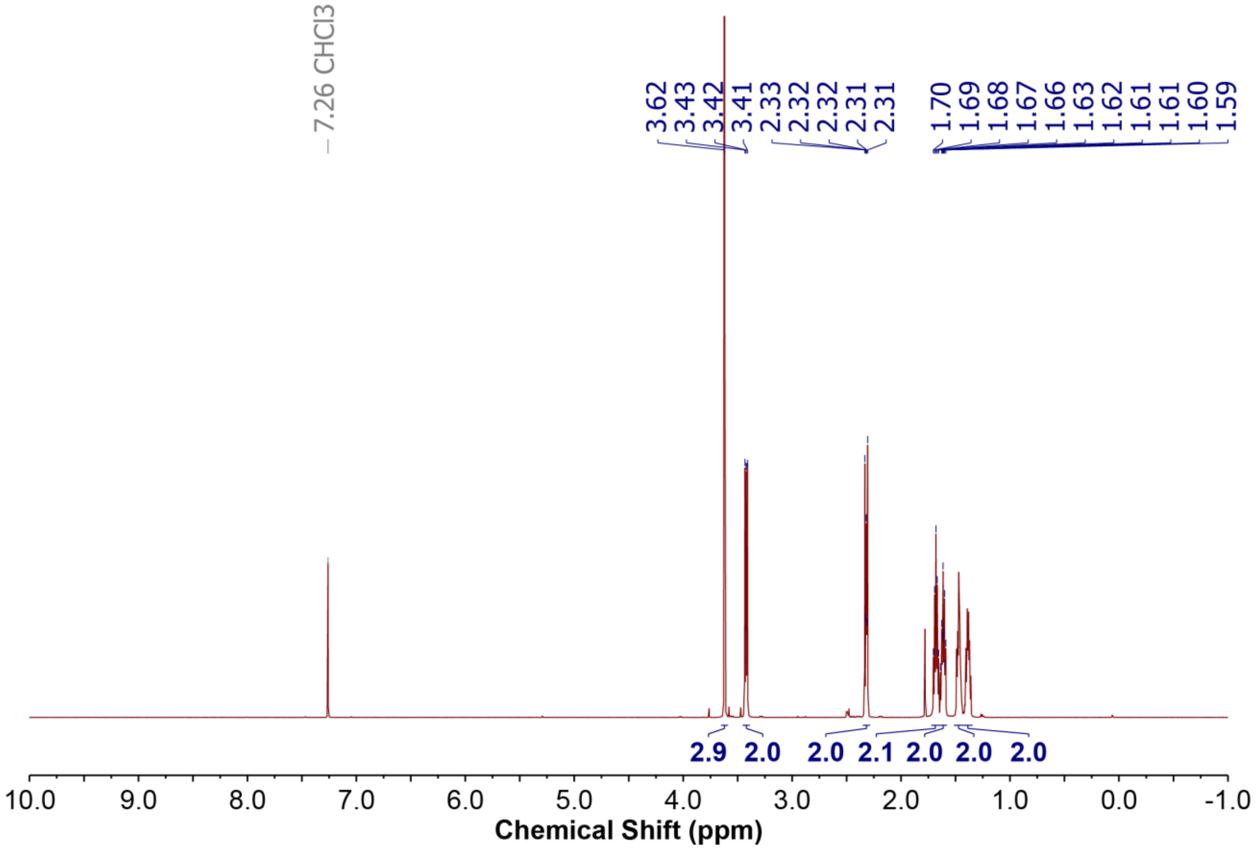

Figure S36. ^{1}H NMR (500 MHz, $CDCl_3$, 23 °C) spectrum of **S4**.

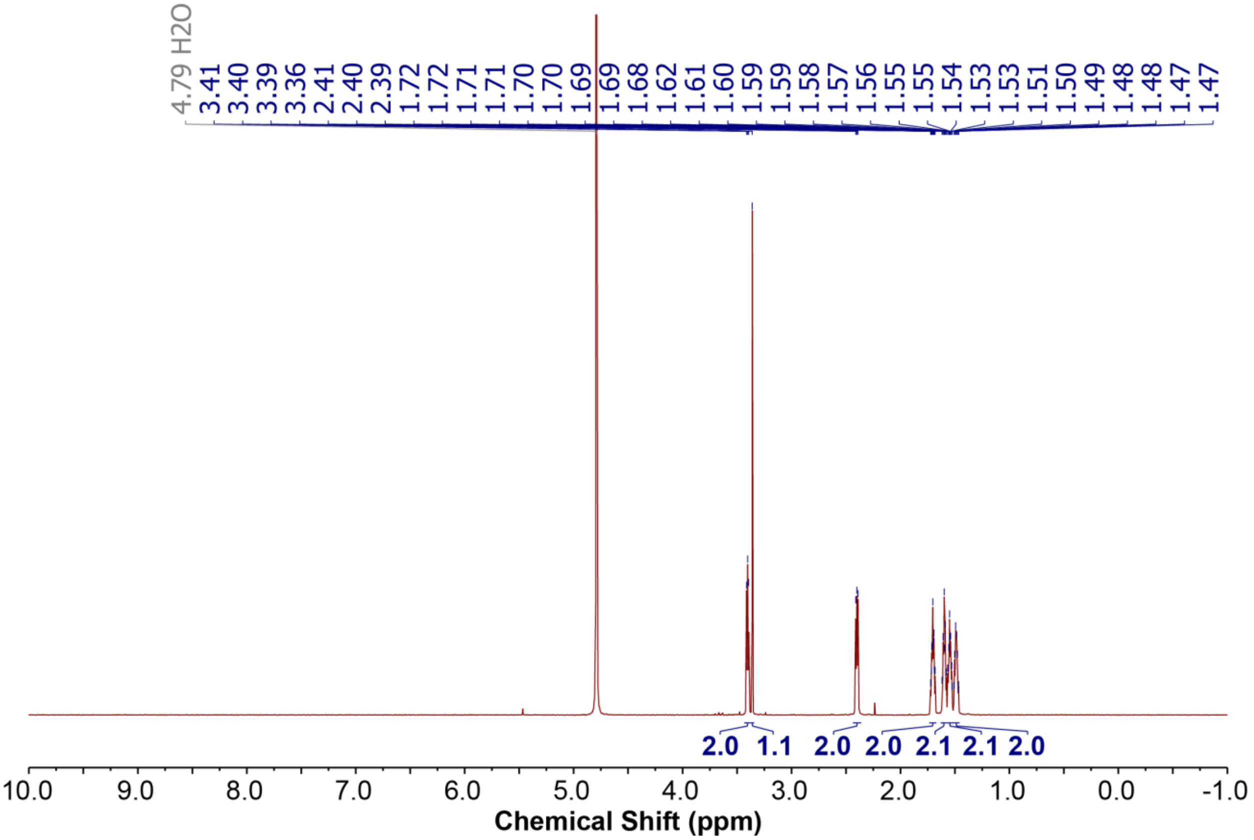

Figure S37. ^{1}H NMR (500 MHz, $CDCl_3$, 23 °C) spectrum of **S5**.

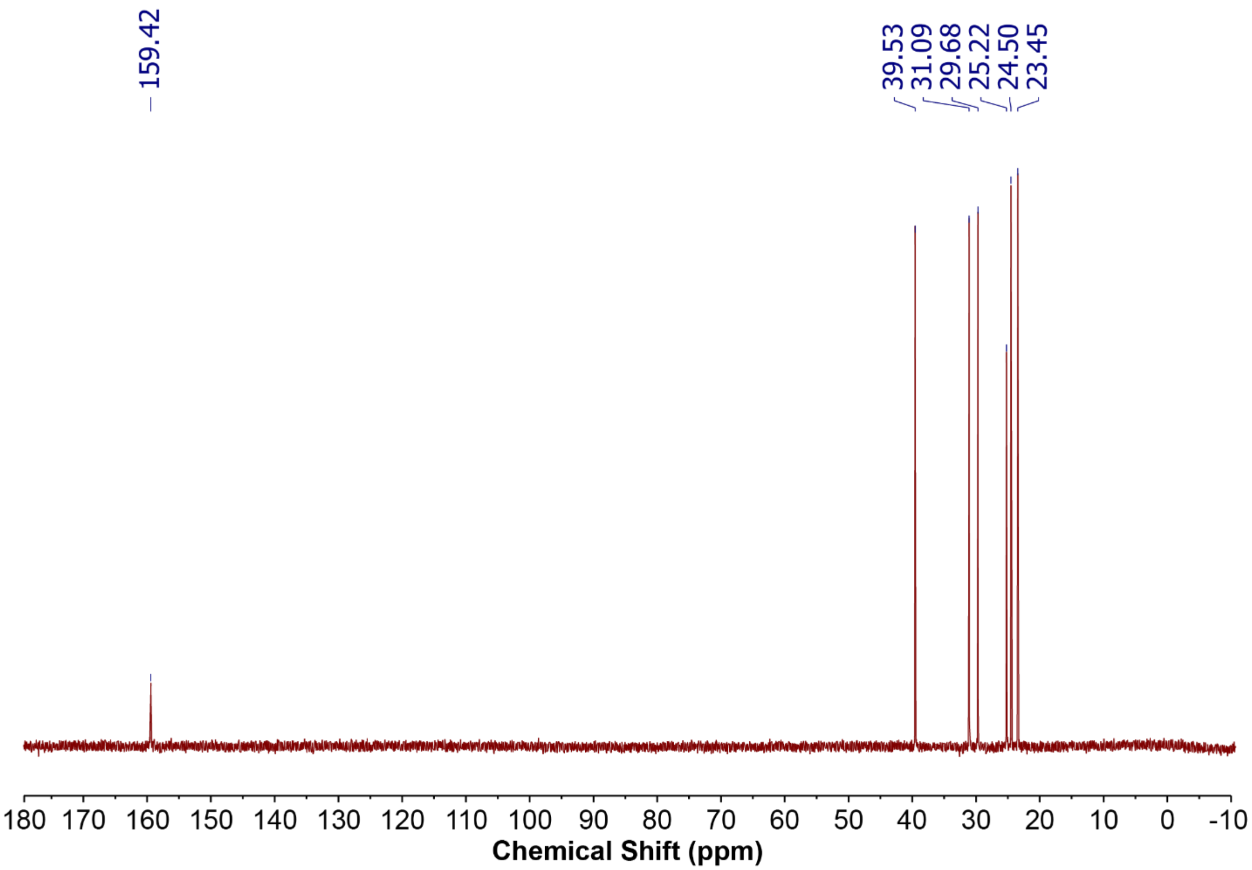

Figure S38. ^{13}C NMR (100 MHz, $CDCl_3$, 23 °C) spectrum of **S5**.

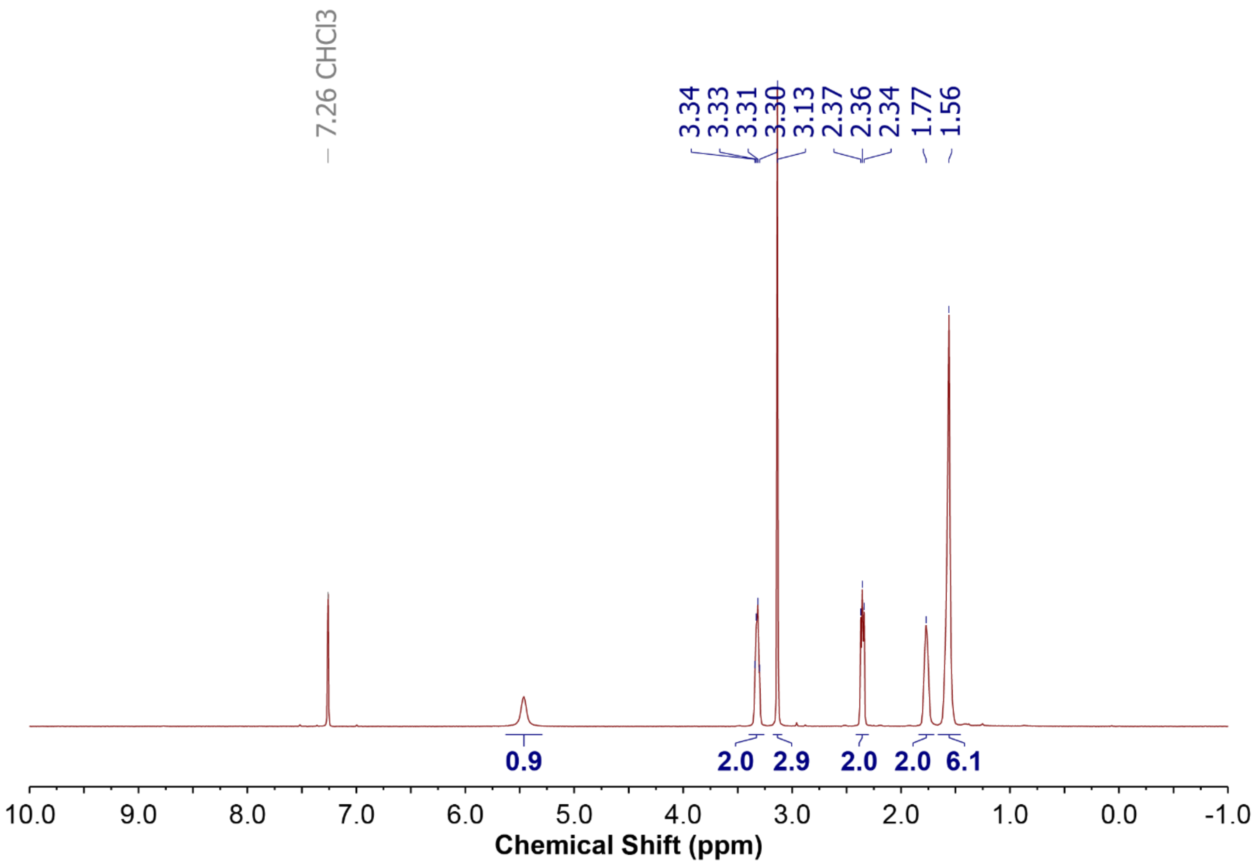

Figure S39. ^{1}H NMR (500 MHz, $CDCl_3$, 23 °C) spectrum of **S6**.

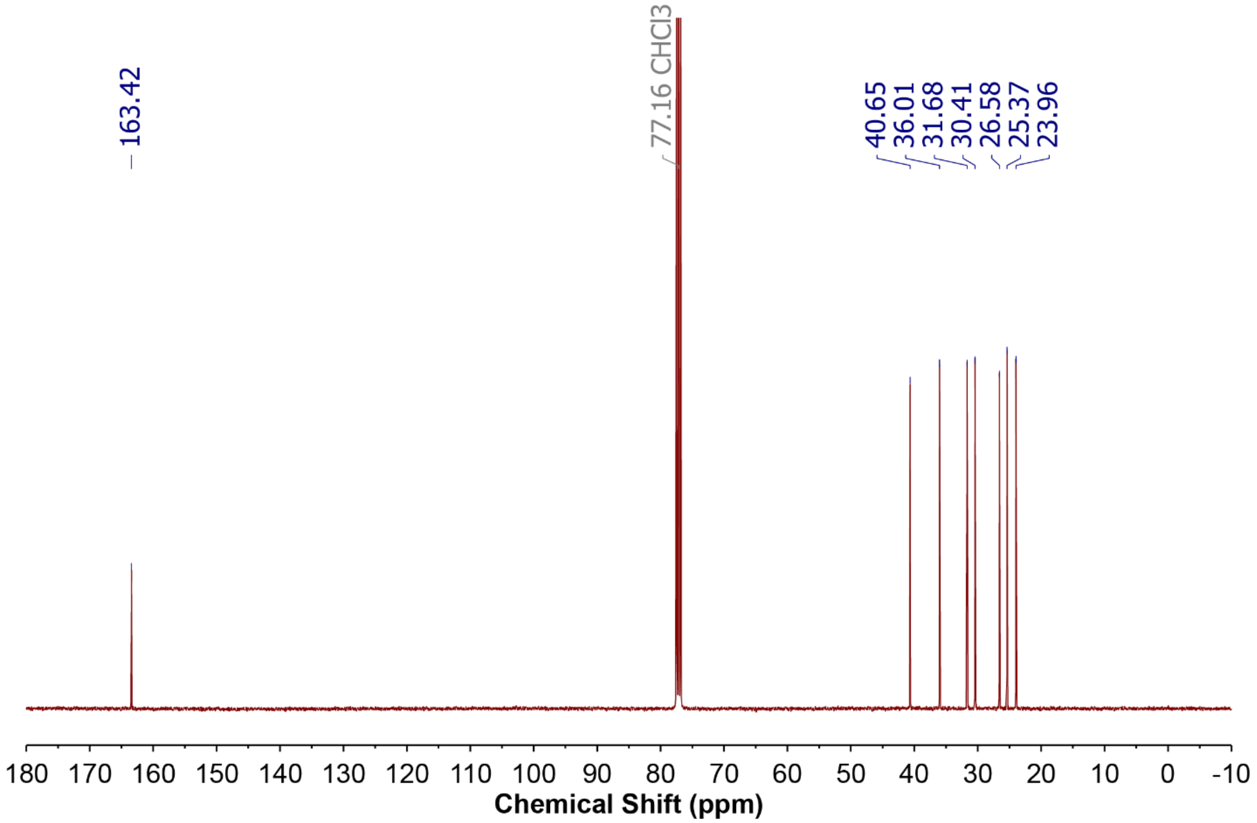

Figure S40. ^{13}C NMR (100 MHz, $CDCl_3$, 23 °C) spectrum of **S6**.

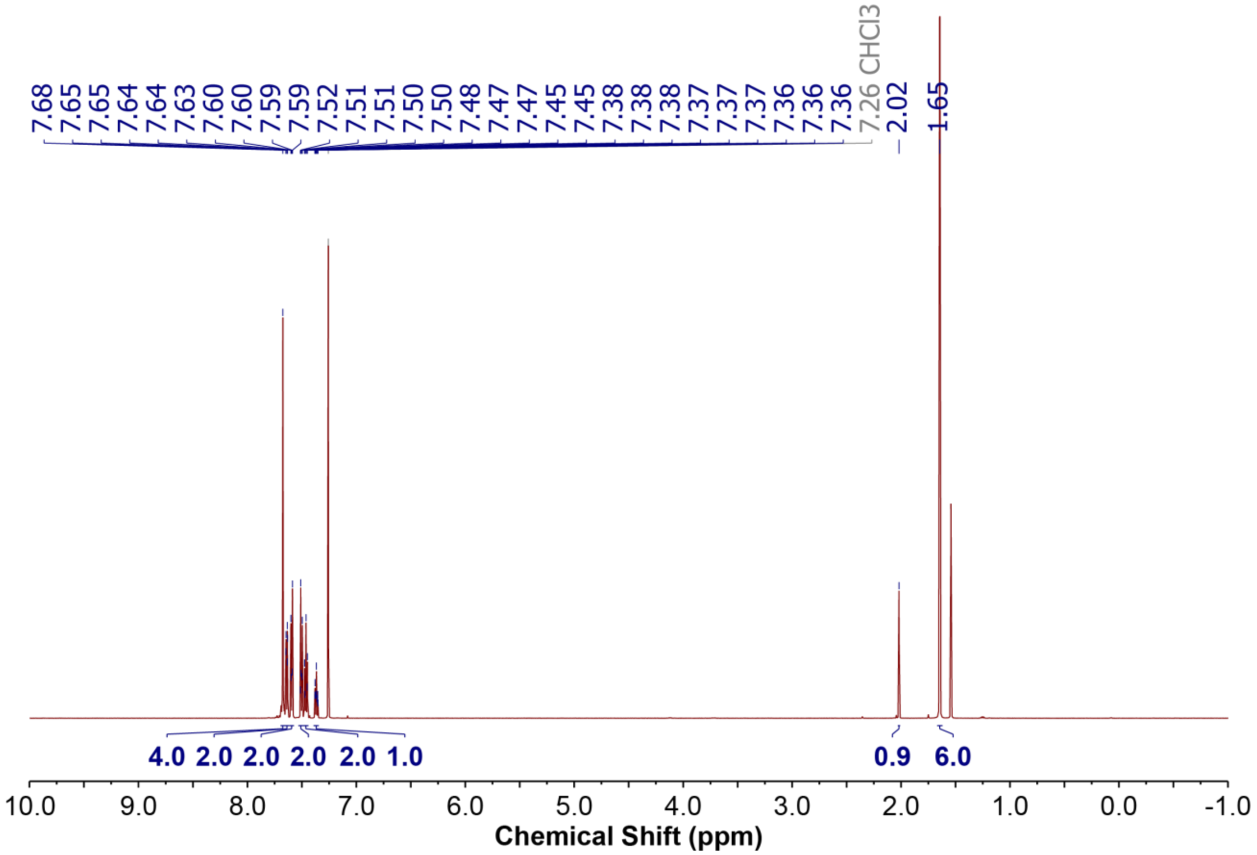

Figure S41. ^{1}H NMR (500 MHz, $CDCl_3$, 23 °C) spectrum of **S7**.

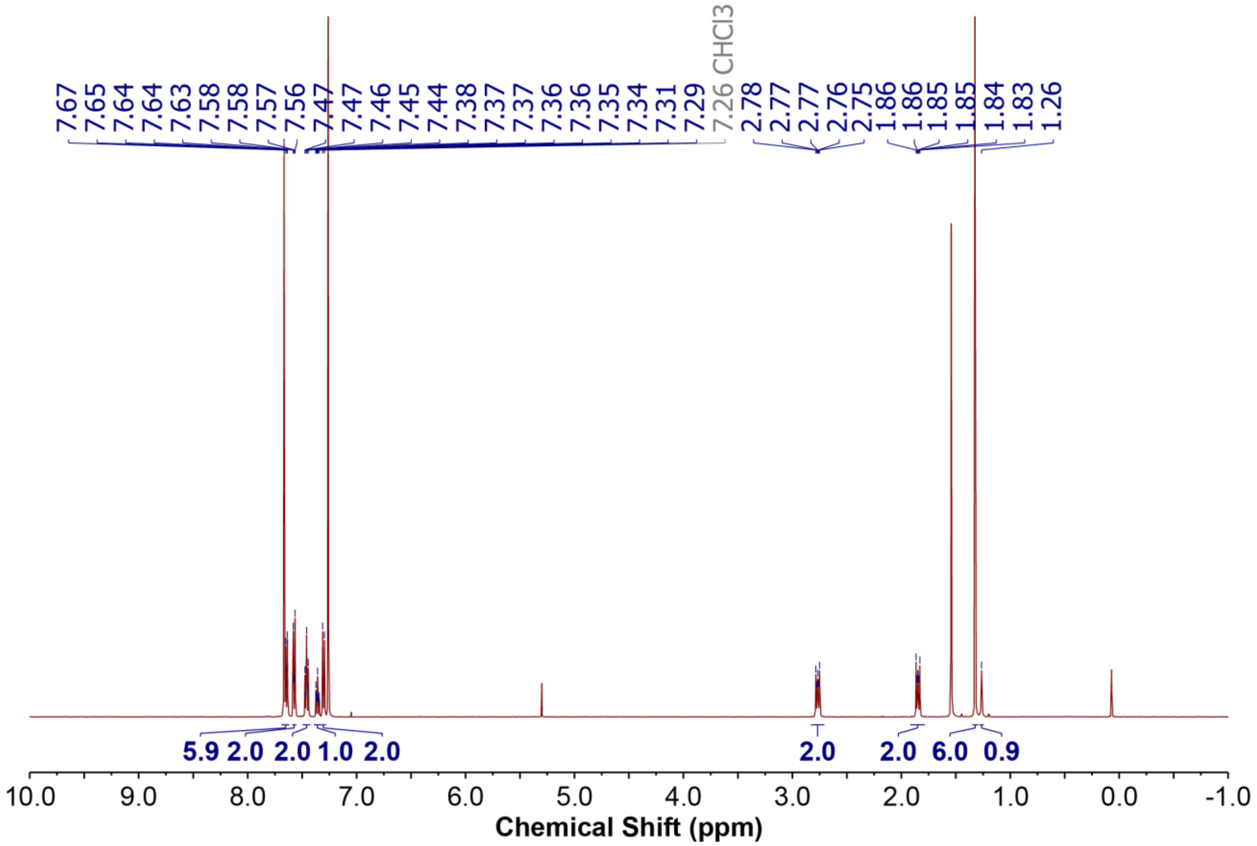

Figure S42. ^{1}H NMR (500 MHz, $CDCl_3$, 23 °C) spectrum of **S8**.

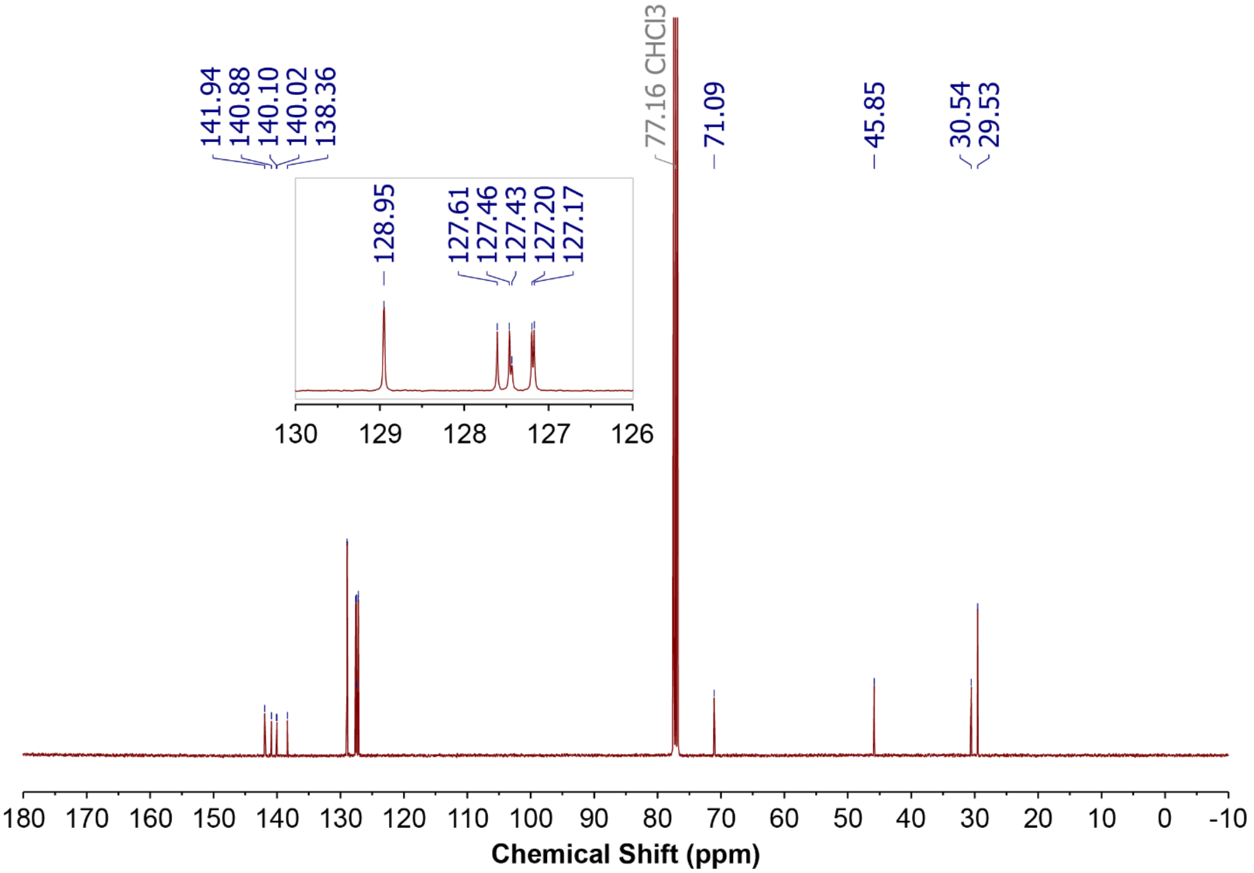

Figure S43. ^{13}C NMR (125 MHz, $CDCl_3$, 23 °C) spectrum of **S8**.

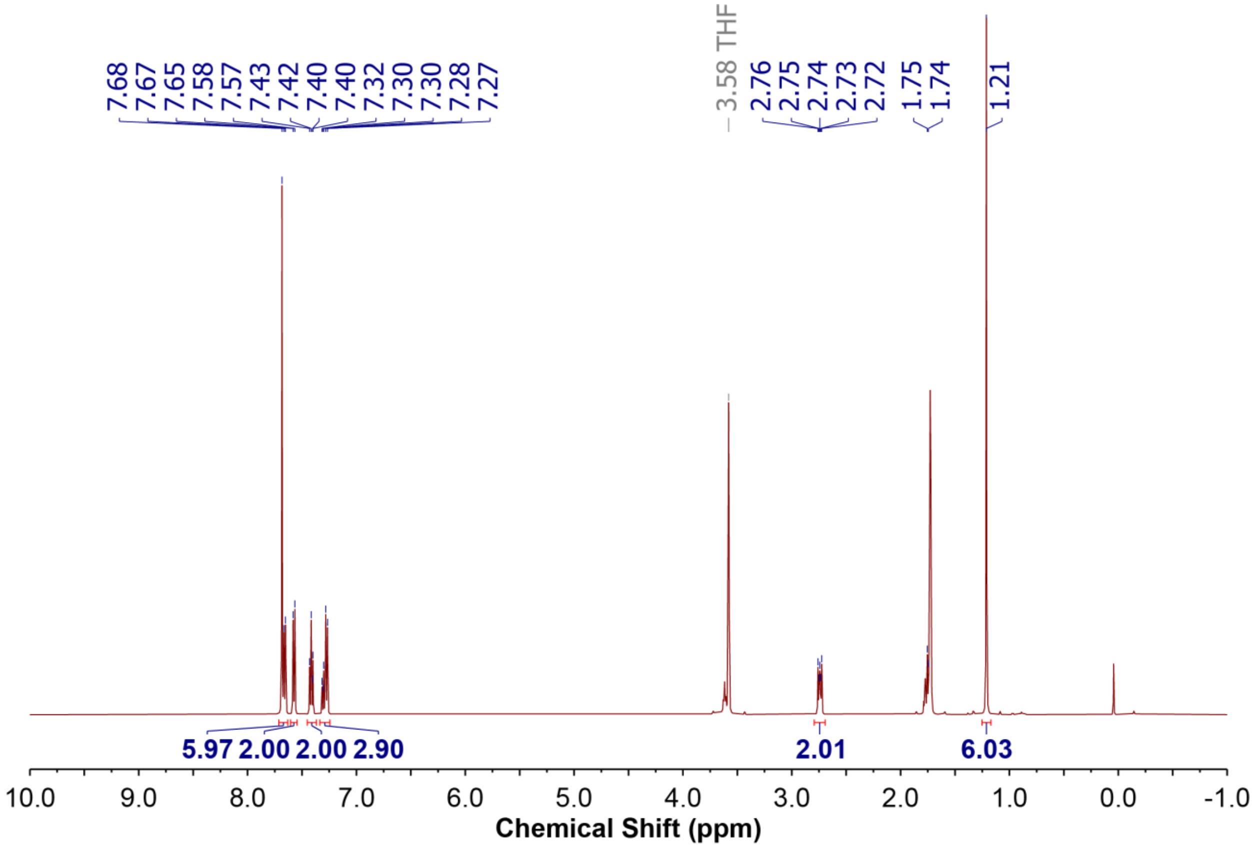

Figure S44. ^{1}H NMR (500 MHz, THF-d_8, 23 °C) spectrum of **S9**.

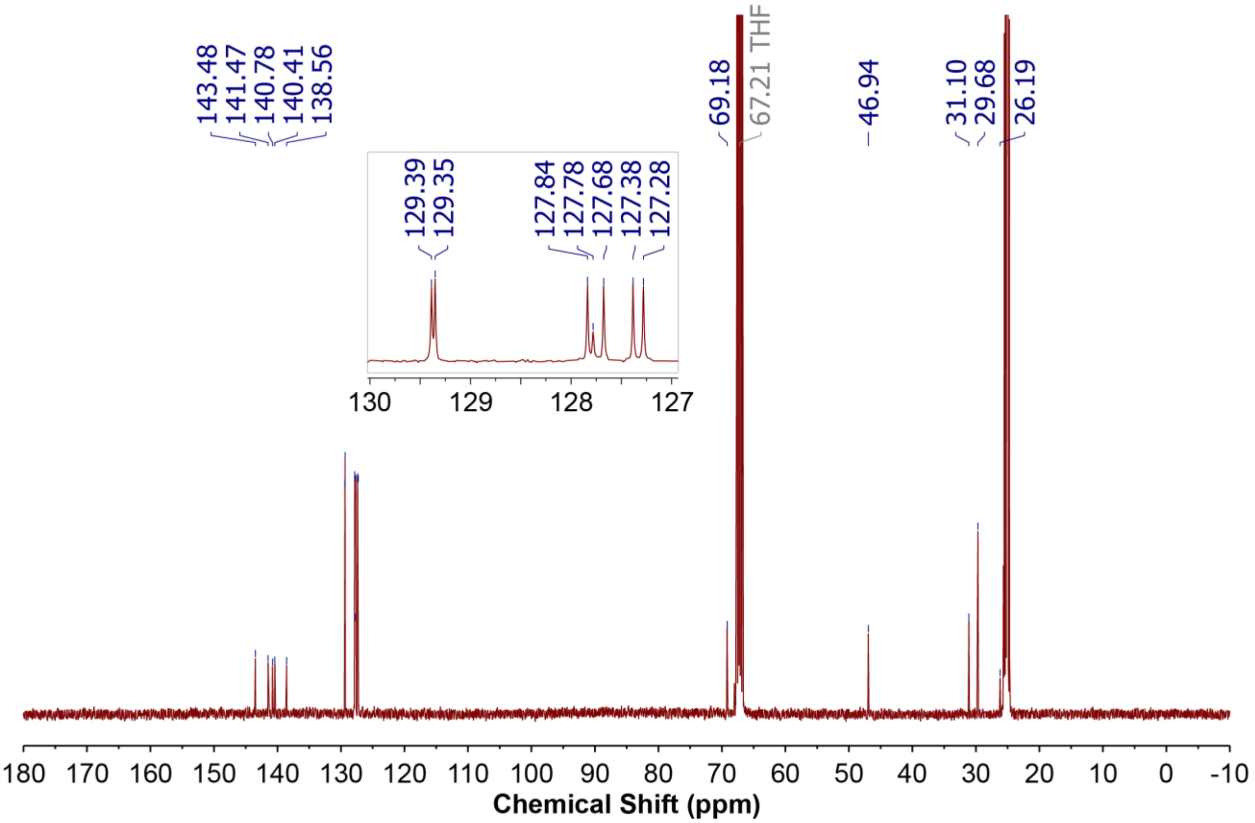

Figure S45. ^{13}C NMR (125 MHz, THF-d_8, 23 °C) spectrum of **S9**.

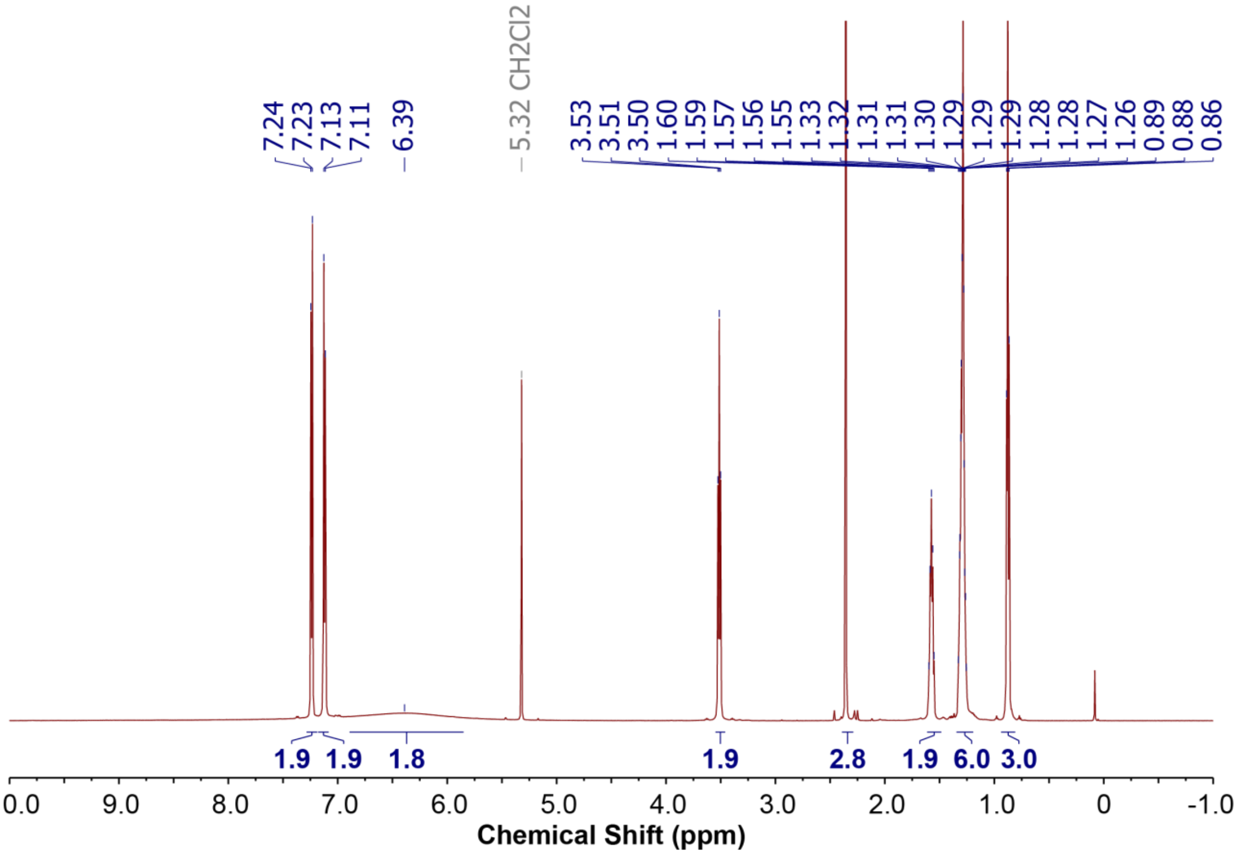

Figure S46. ^{1}H NMR (600 MHz, CD_2Cl_2, 23 °C) spectrum of **S10**.

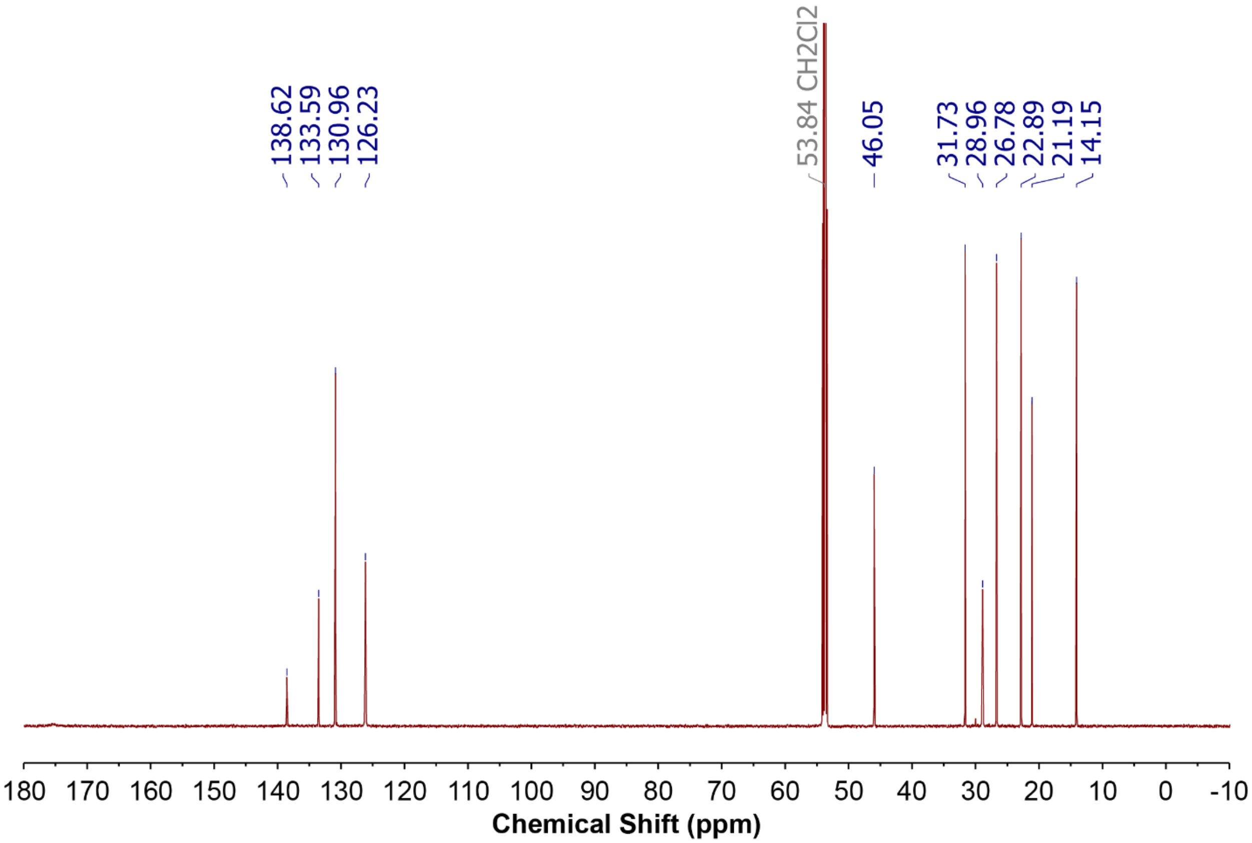

Figure S47. ^{13}C NMR (150 MHz, CD_2Cl_2, 23 °C) spectrum of **S10**.

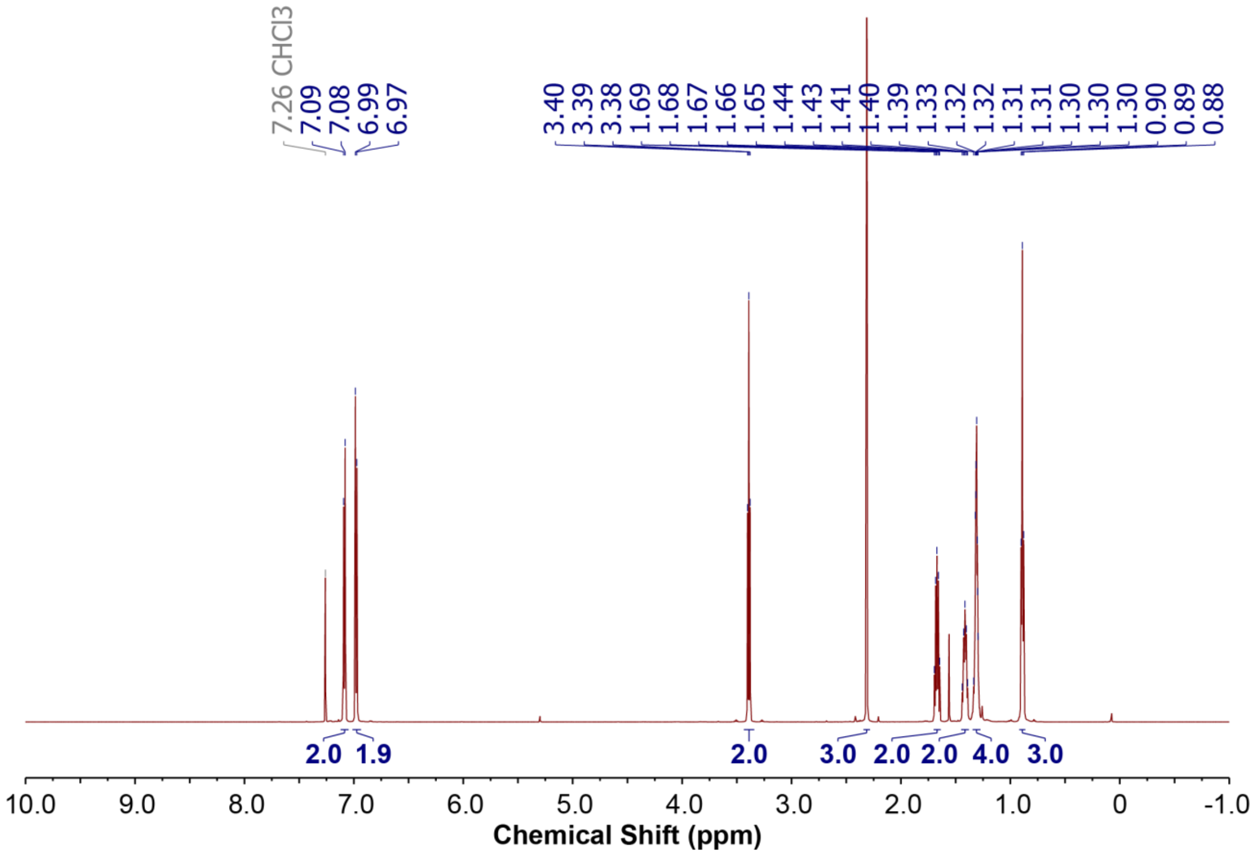

Figure S48. ^{1}H NMR (600 MHz, $CDCl_3$, 23 °C) spectrum of **S11**.

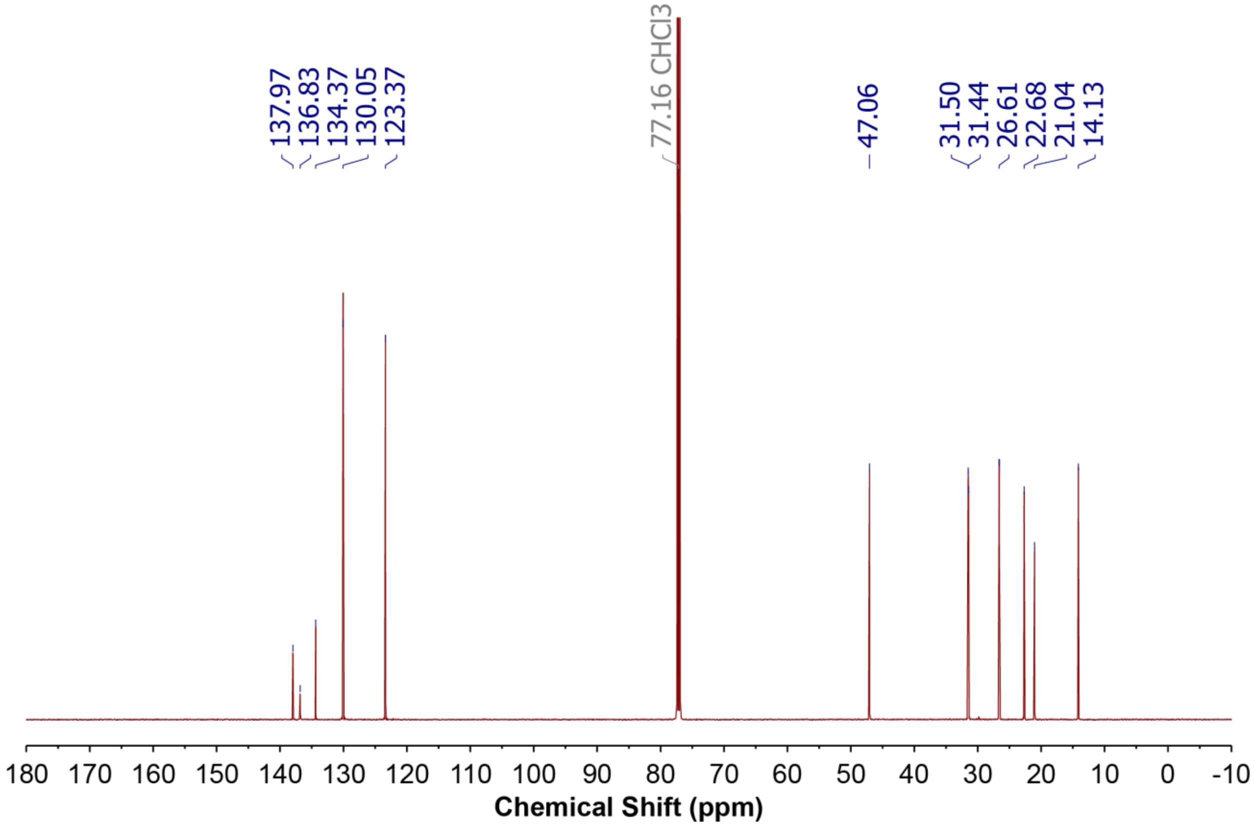

Figure S49. ^{13}C NMR (150 MHz, $CDCl_3$, 23 °C) spectrum of **S11**.

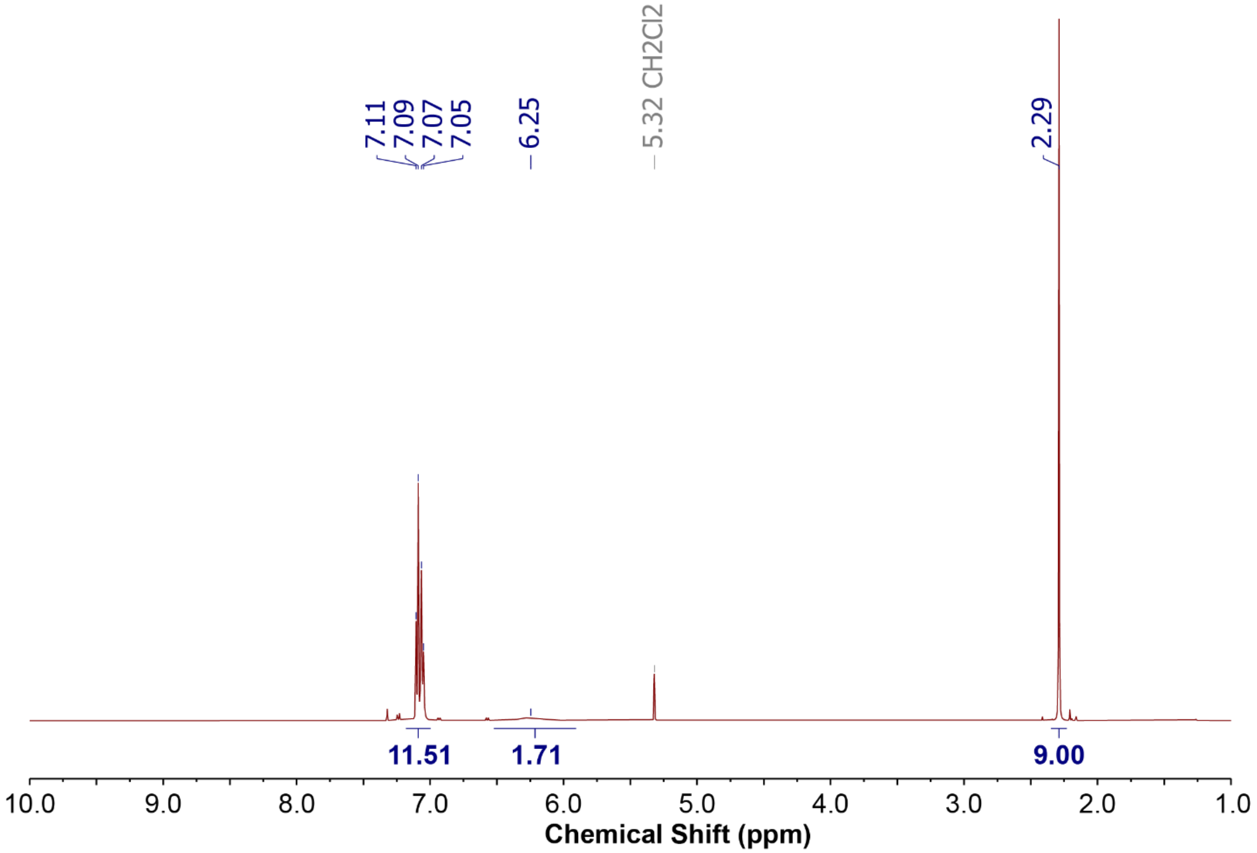

Figure S50. ^{1}H NMR (500 MHz, CD_2Cl_2, 23 °C) spectrum of **S12**.

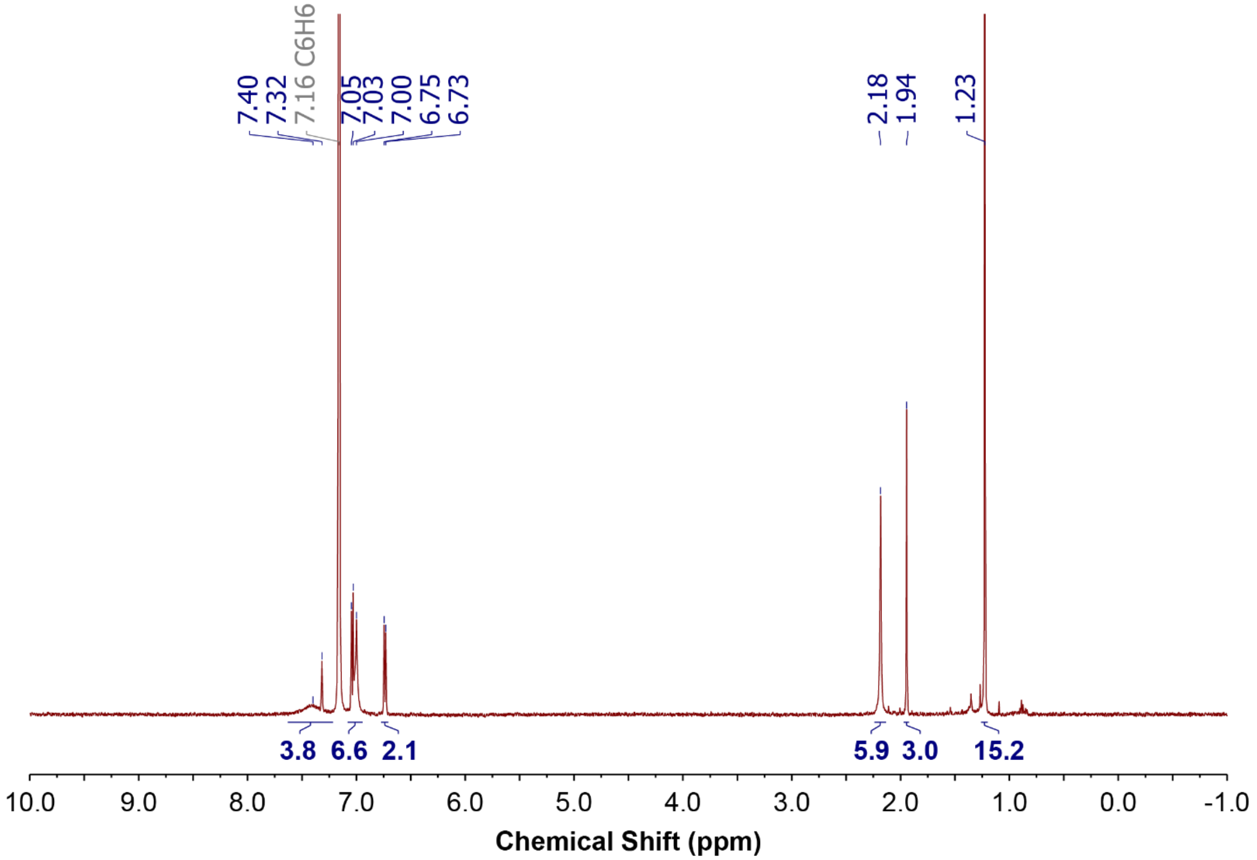

Figure S51. ^{1}H NMR (500 MHz, C_6D_6, 23 °C) spectrum of **1**.

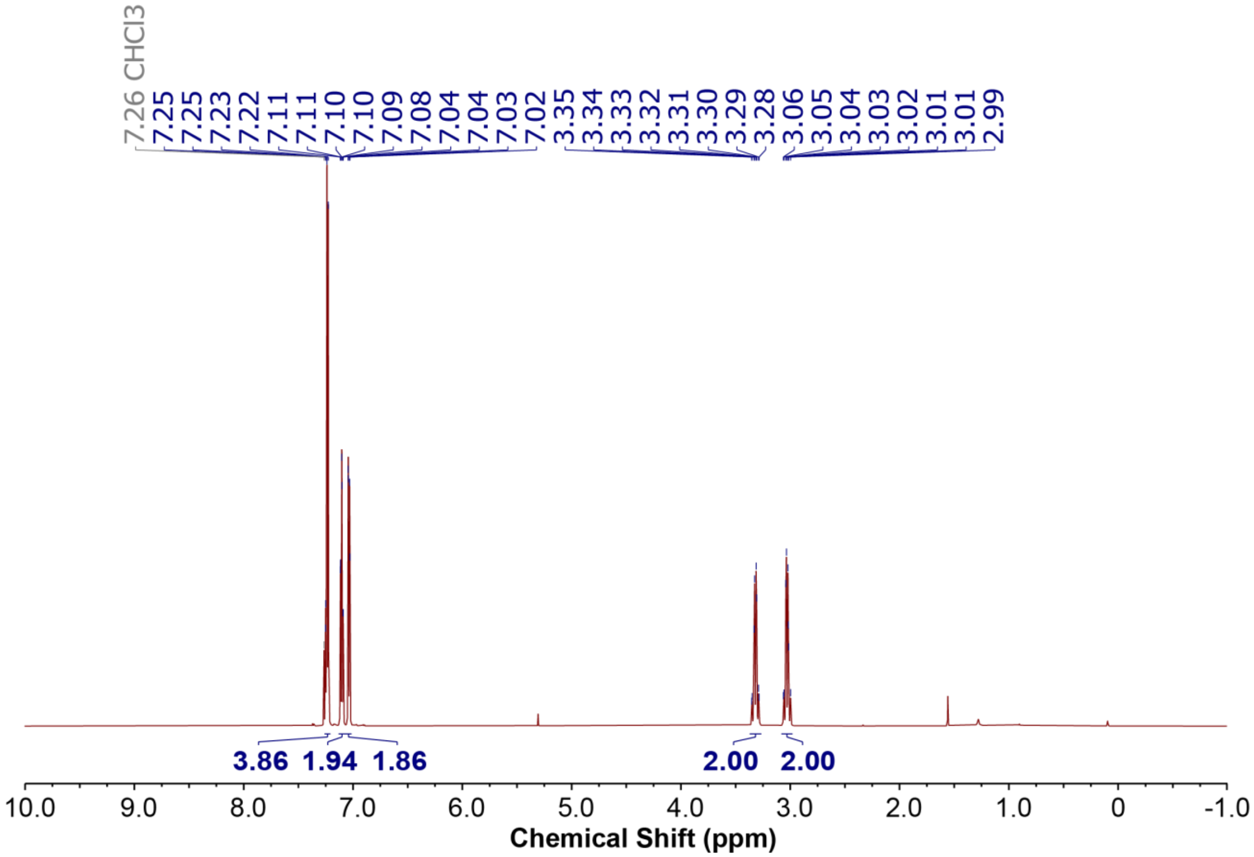

Figure S52. ^{1}H NMR (400 MHz, $CDCl_3$, 23 °C) spectrum of **M1**.

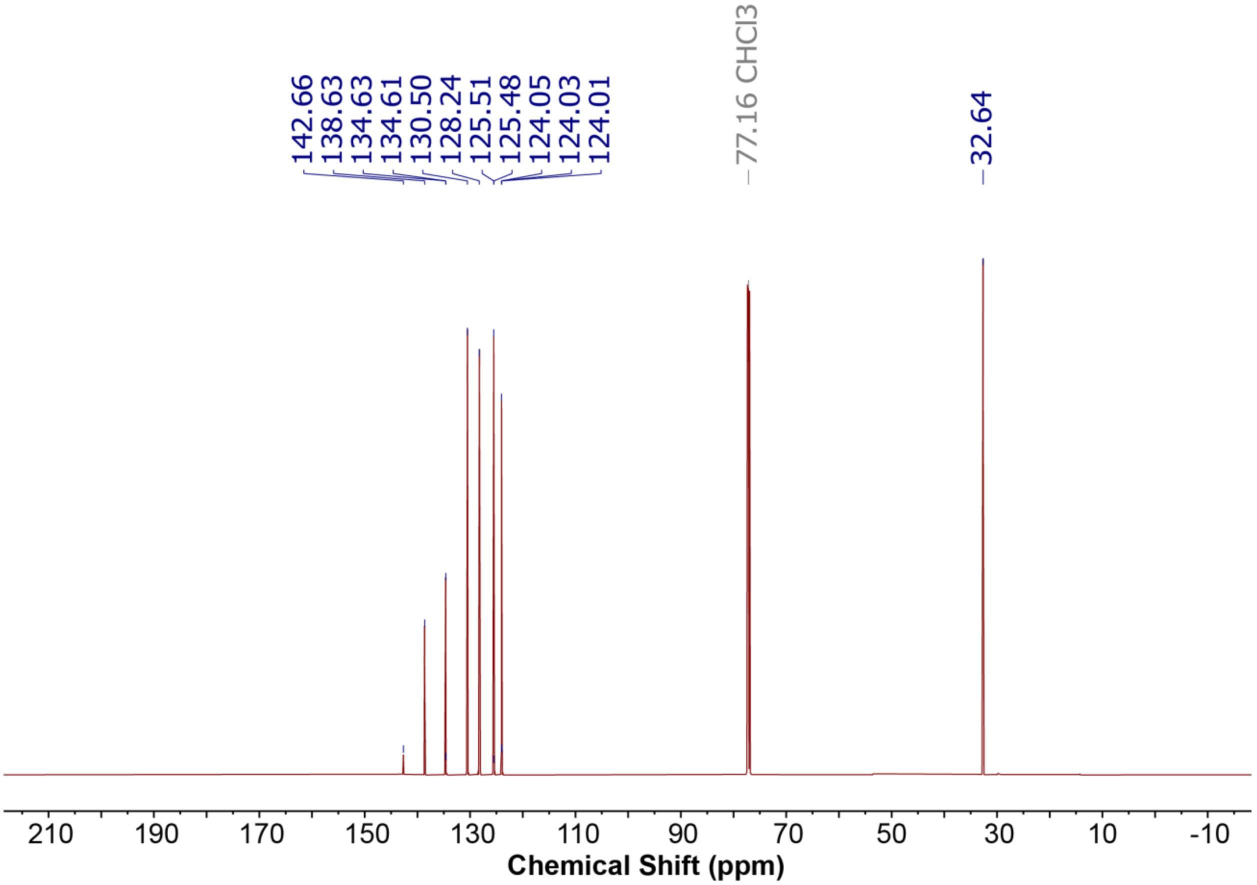

Figure S53. ^{13}C NMR (400 MHz, $CDCl_3$, 23 °C) spectrum of **M1**.

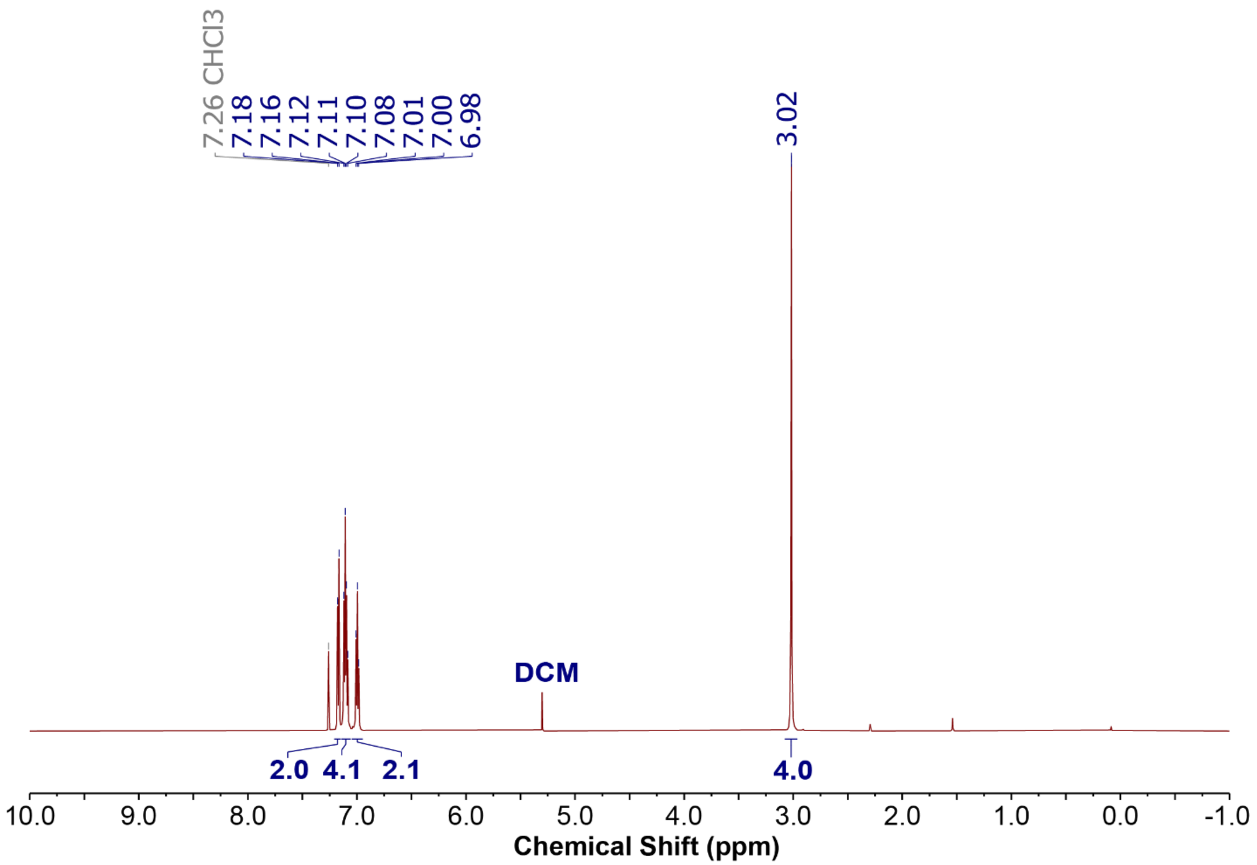

Figure S54. ^{1}H NMR (500 MHz, $CDCl_3$, 23 °C) spectrum of **polyM1** following precipitation into MeOH and purification via prep-GPC.

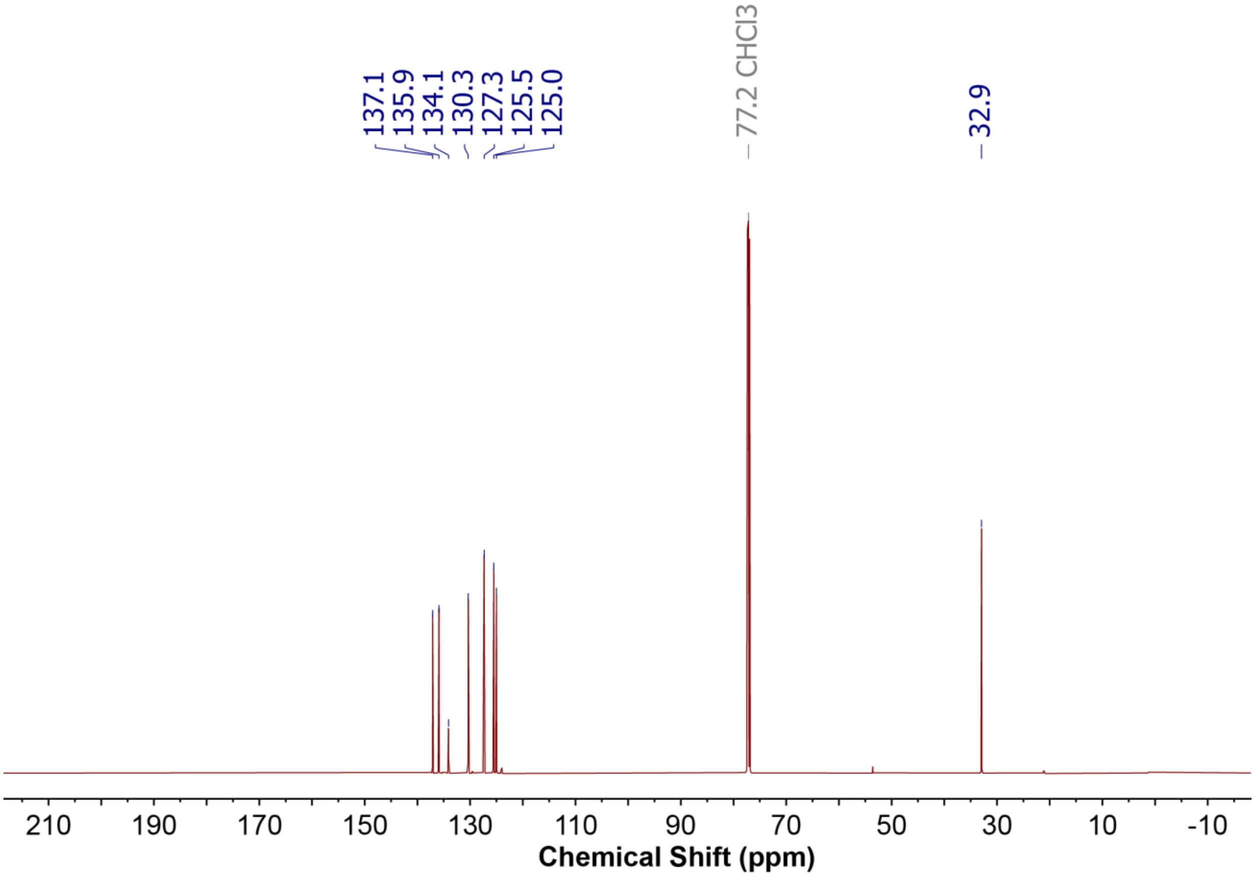

Figure S55. ^{13}C NMR (100 MHz, $CDCl_3$, 23 °C) spectrum of **polyM1** following precipitation into MeOH and purification via prep-GPC.

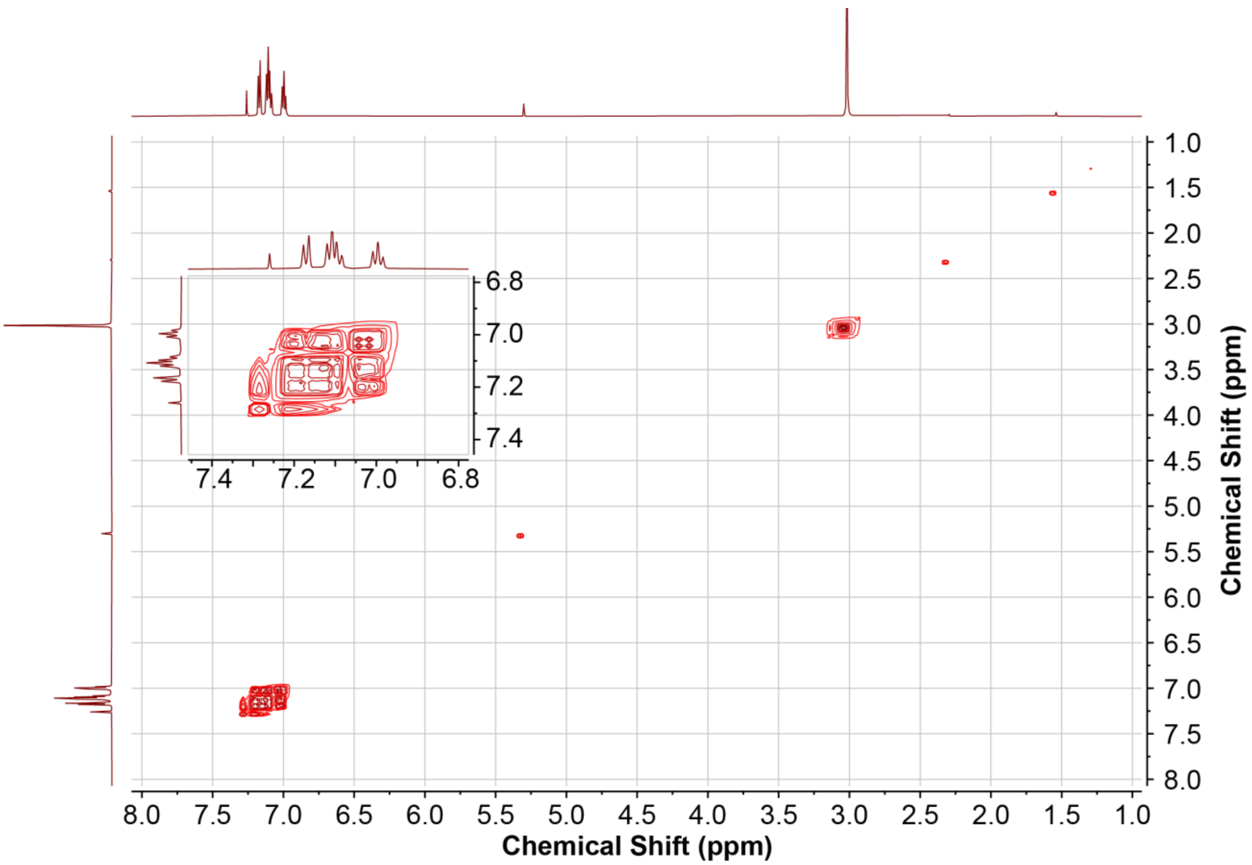

Figure S56. ^1H-^1H correlated spectroscopy (COSY, 400 MHz, $CDCl_3$, 23 °C) spectrum of **polyM1** following precipitation into MeOH and purification via prep-GPC.

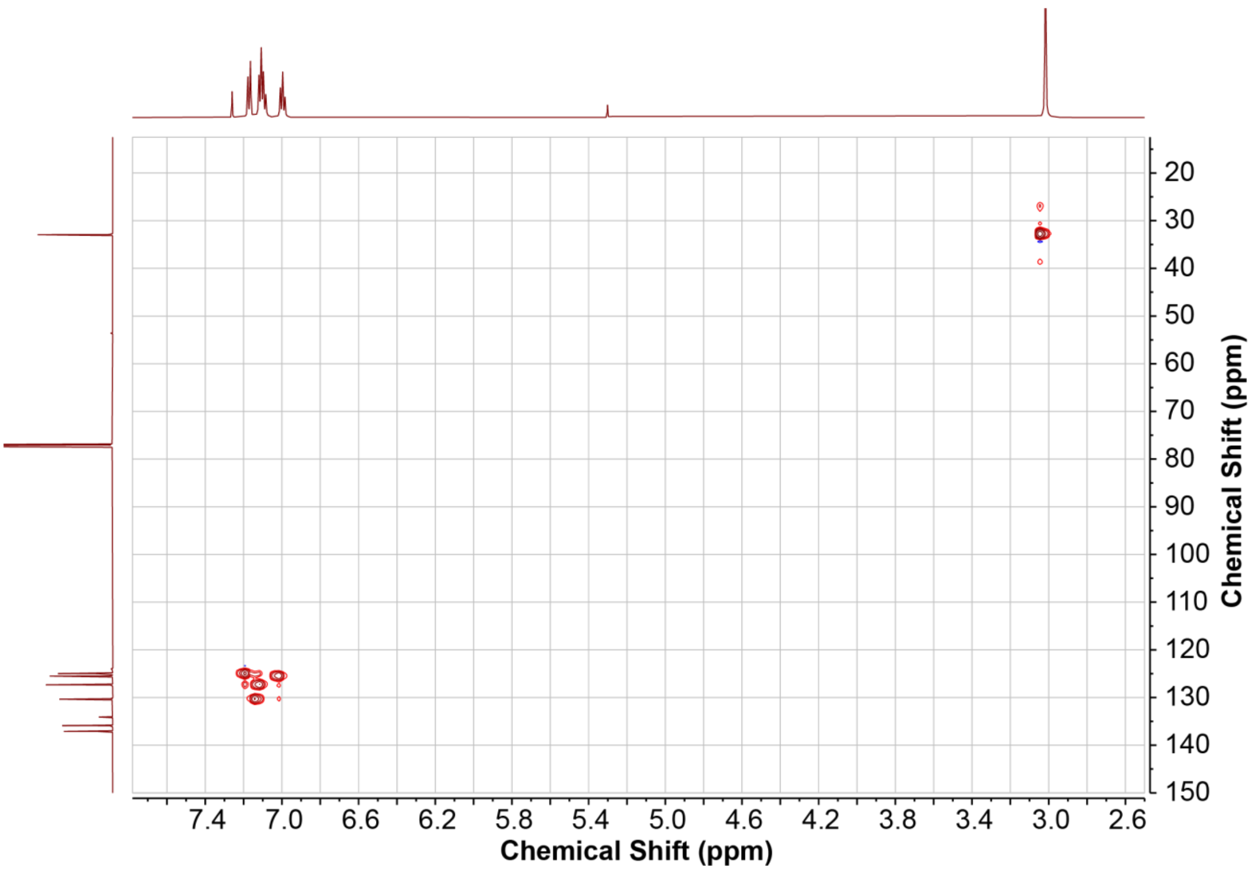

Figure S57. ^{1}H-^{13}C heteronuclear single quantum coherence (HSQC, 400 MHz ^{1}H/100 MHz ^{13}C, $CDCl_3$, 23 °C) spectrum of **polyM1** following precipitation into MeOH and purification via preparatory GPC.

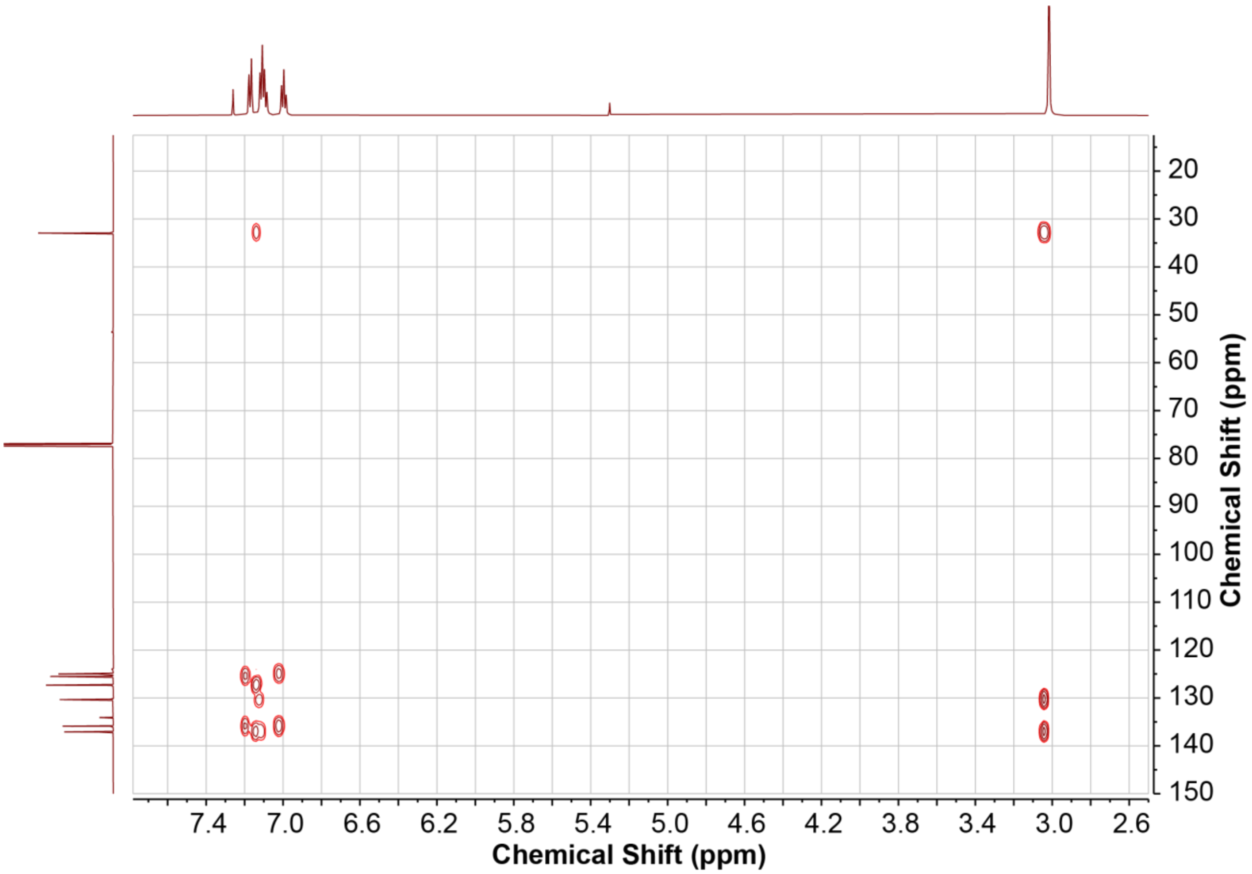

Figure S58. ^{1}H-^{13}C heteronuclear multiple bond correlation (HMBC, 400 MHz ^{1}H/100 MHz ^{13}C, $CDCl_3$, 23 °C) spectrum of **polyM1** following precipitation into MeOH and purification via prep-GPC.

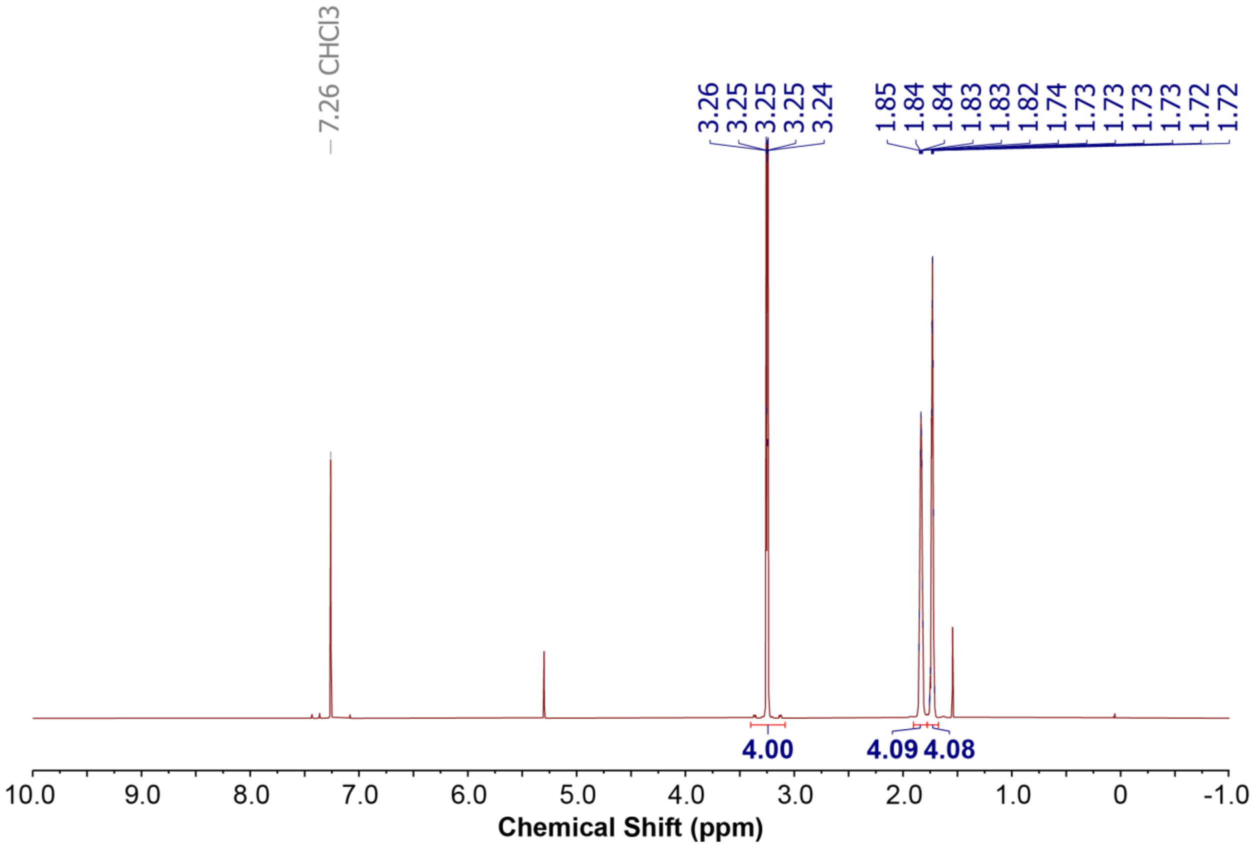

Figure S59. ^{1}H NMR (400 MHz, $CDCl_3$, 23 °C) spectrum of **M2**.

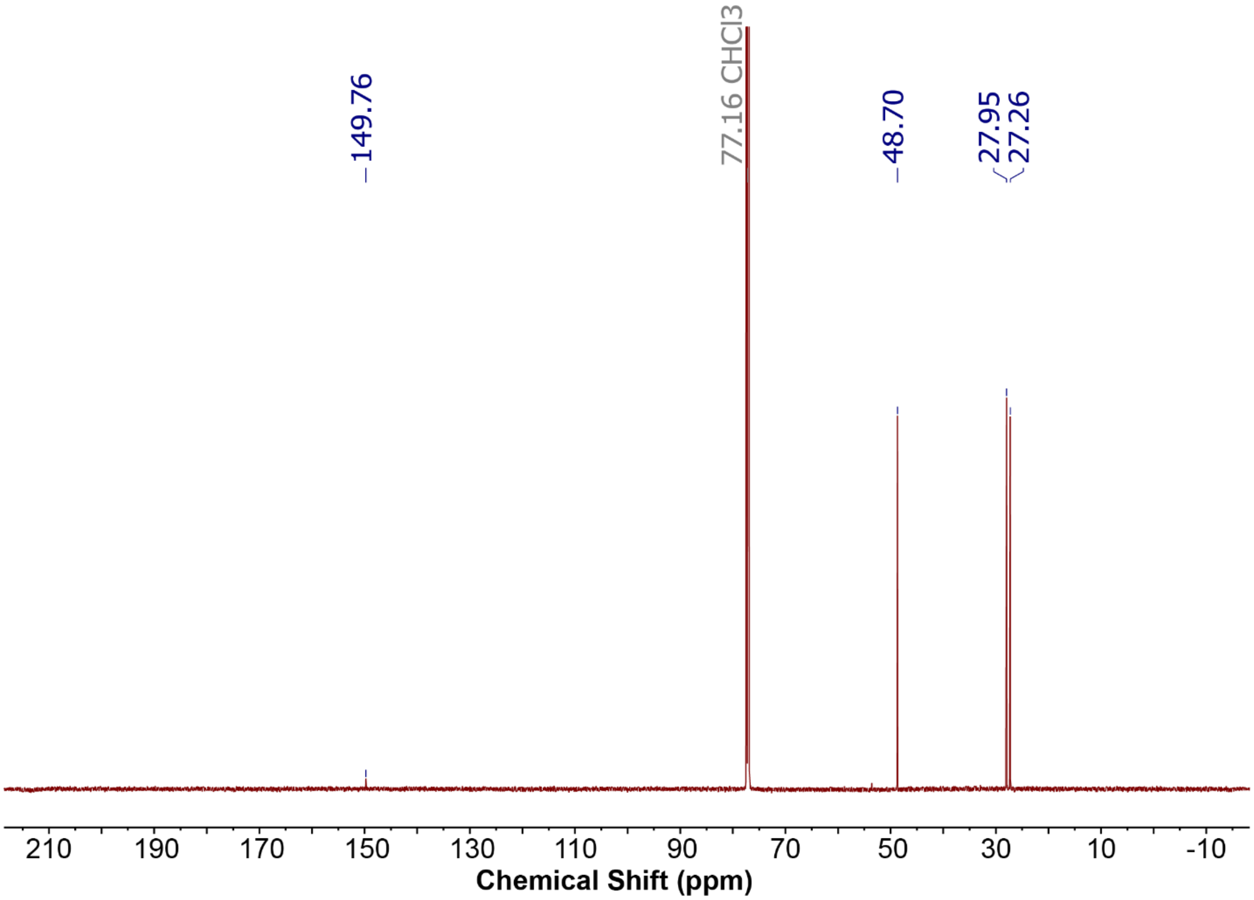

Figure S60. ^{13}C NMR (100 MHz, $CDCl_3$, 23 °C) spectrum of **M2**.

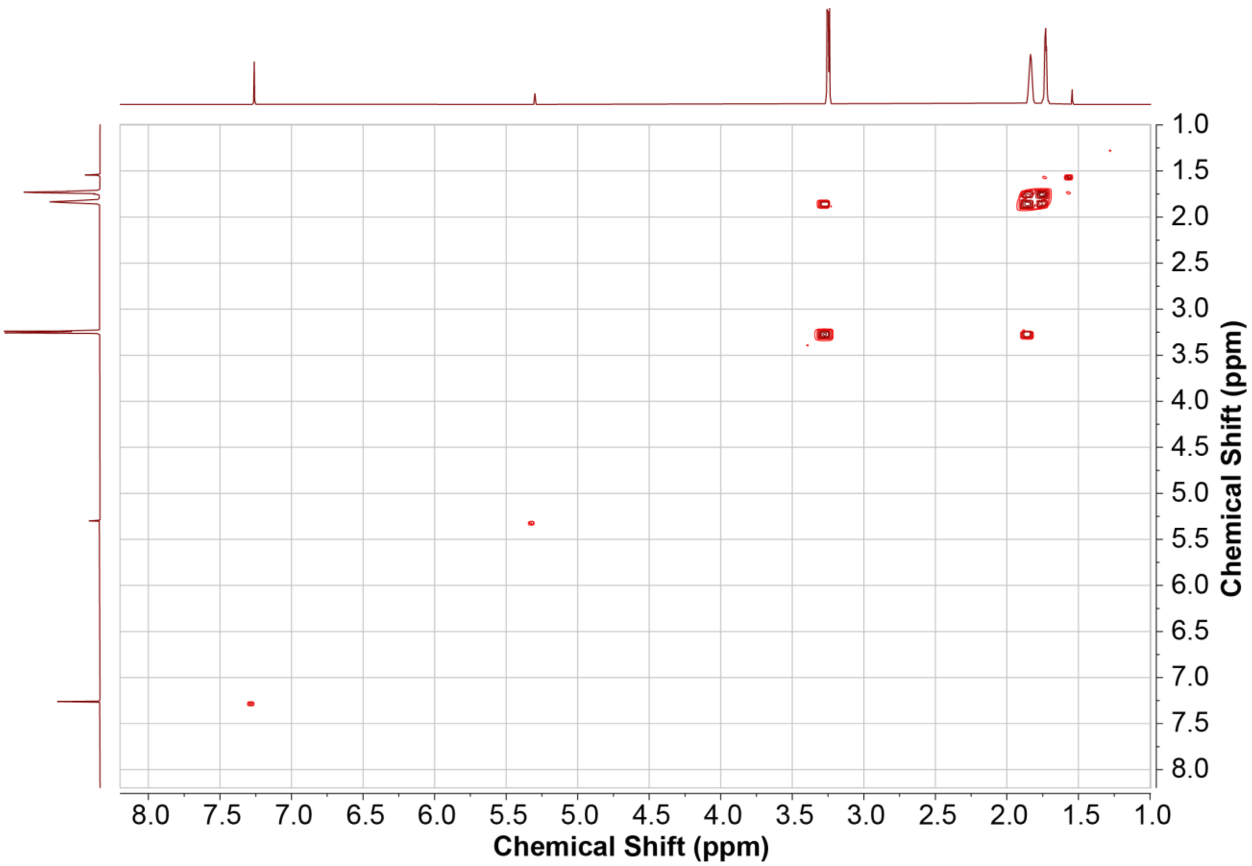

Figure S61. ^{1}H-^{1}H COSY (400 MHz, $CDCl_3$, 23 °C) spectrum of **M2**.

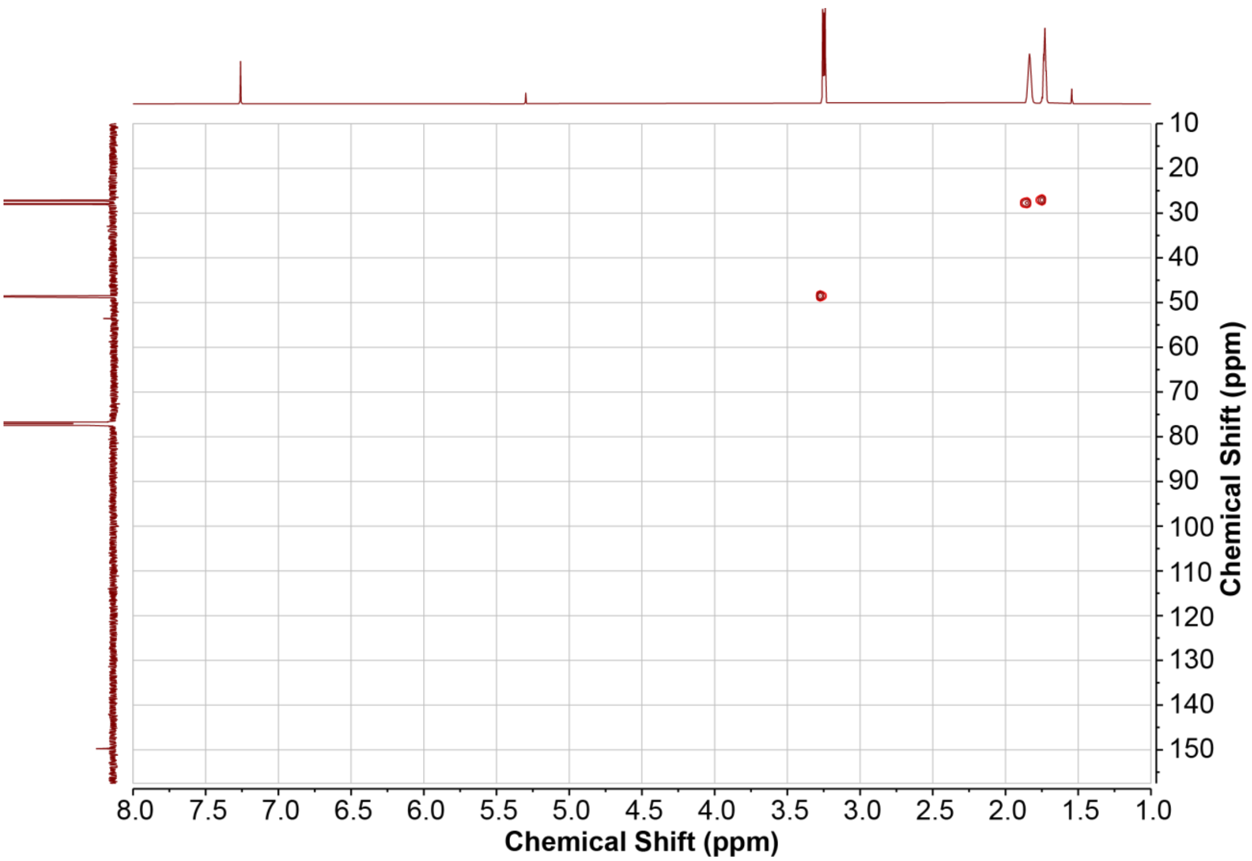

Figure S62. ^{1}H-^{13}C HSQC (400 MHz ^{1}H/100 MHz ^{13}C, $CDCl_3$, 23 °C) spectrum of **M2**.

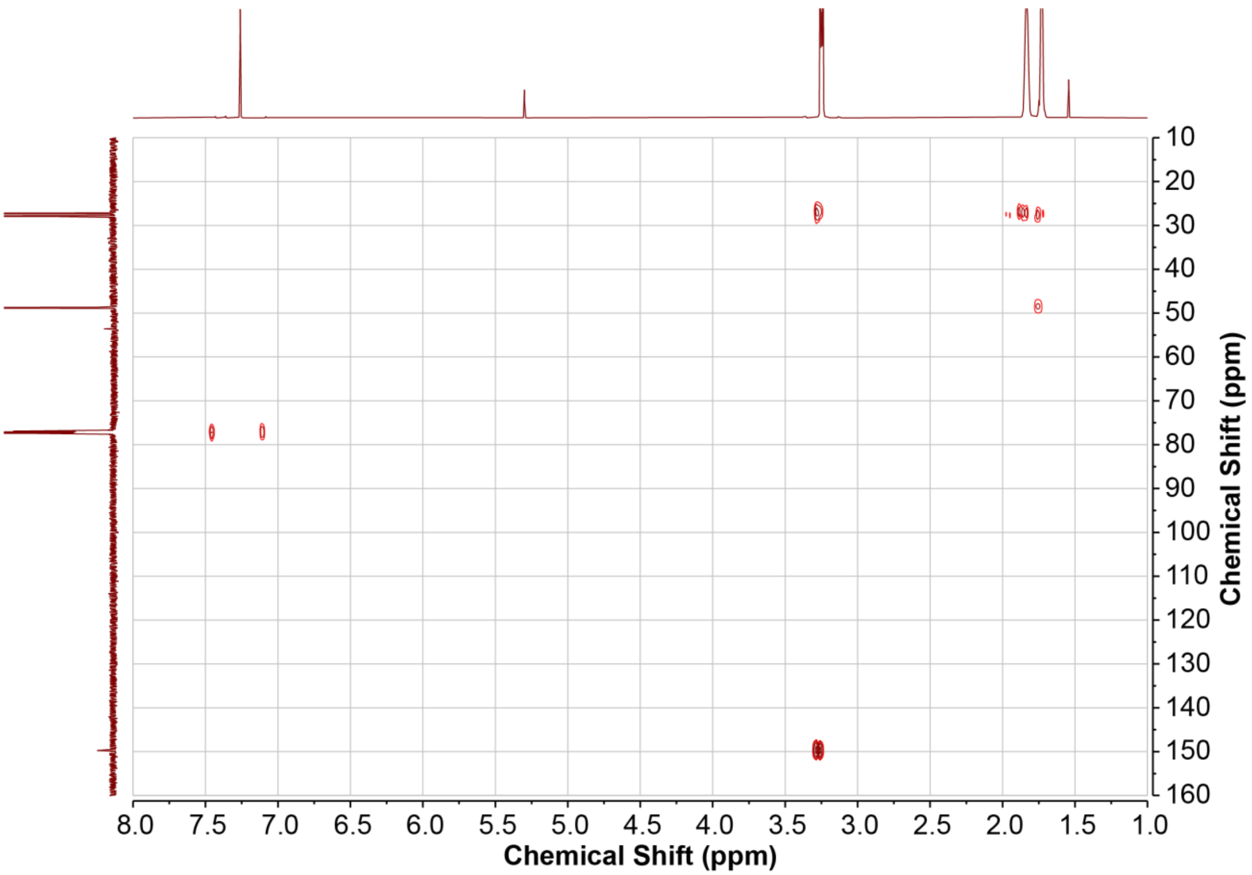

Figure S63. ^{1}H-^{13}C HMBC (400 MHz ^{1}H/100 MHz ^{13}C, $CDCl_3$, 23 °C) spectrum of **M2**.

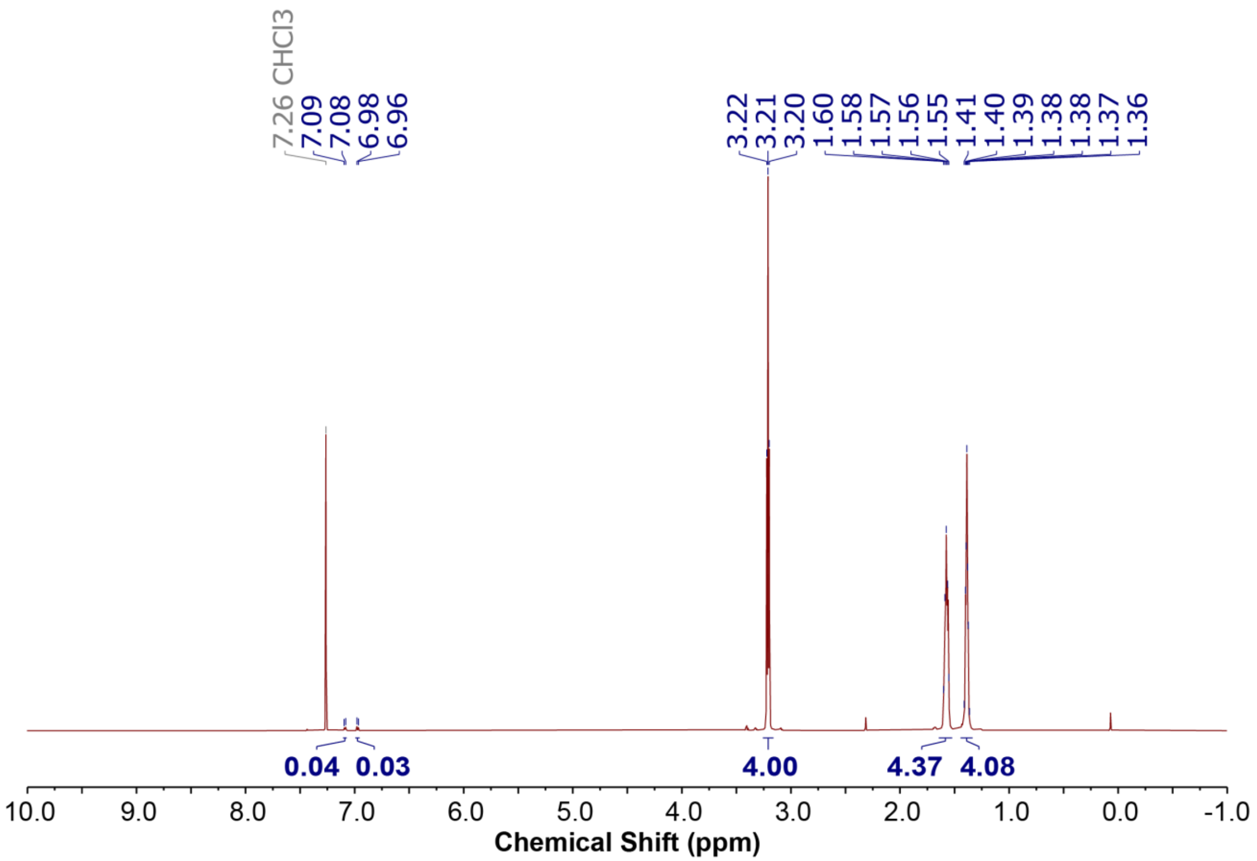

Figure S64. ^{1}H NMR (600 MHz, $CDCl_3$, 23 °C) spectrum of **polyM2** following precipitation into hexanes and centrifugation (10 min, 4000 rpm).

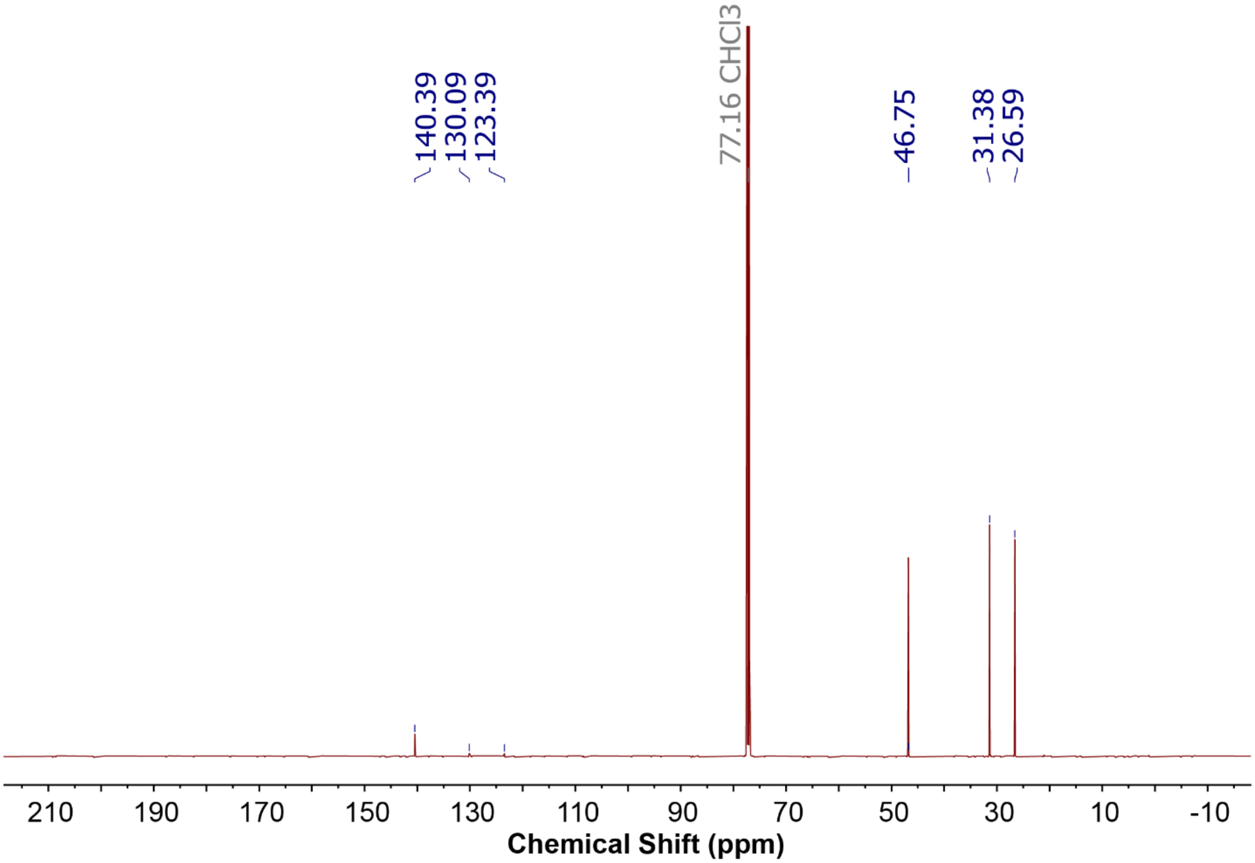

Figure S65. ^{13}C NMR (150 MHz, $CDCl_3$, 23 °C) spectrum of **polyM2** following precipitation into hexanes and centrifugation (10 min, 4000 rpm).

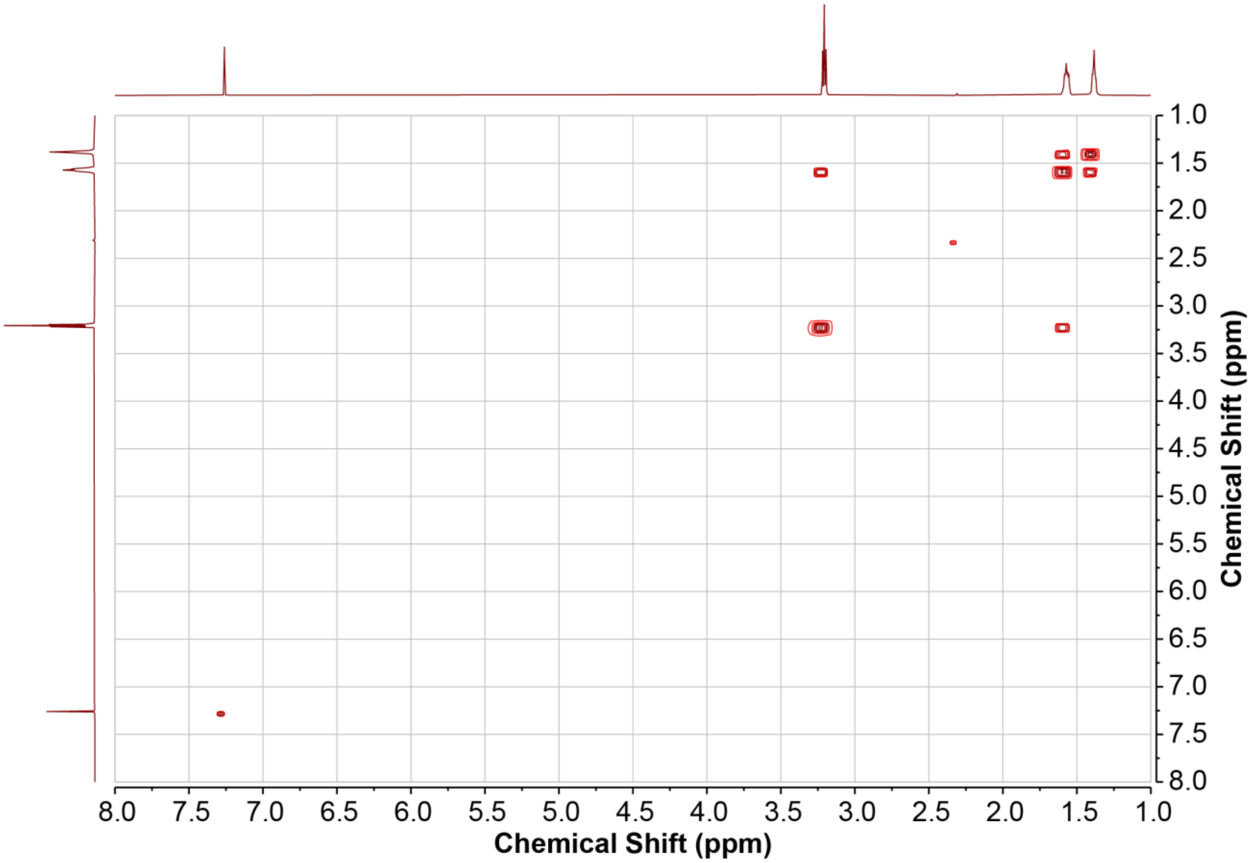

Figure S66. ^{1}H-^{1}H COSY (600 MHz, $CDCl_3$, 23 °C) spectrum of **polyM2** following precipitation into hexanes and centrifugation (10 min, 4000 rpm).

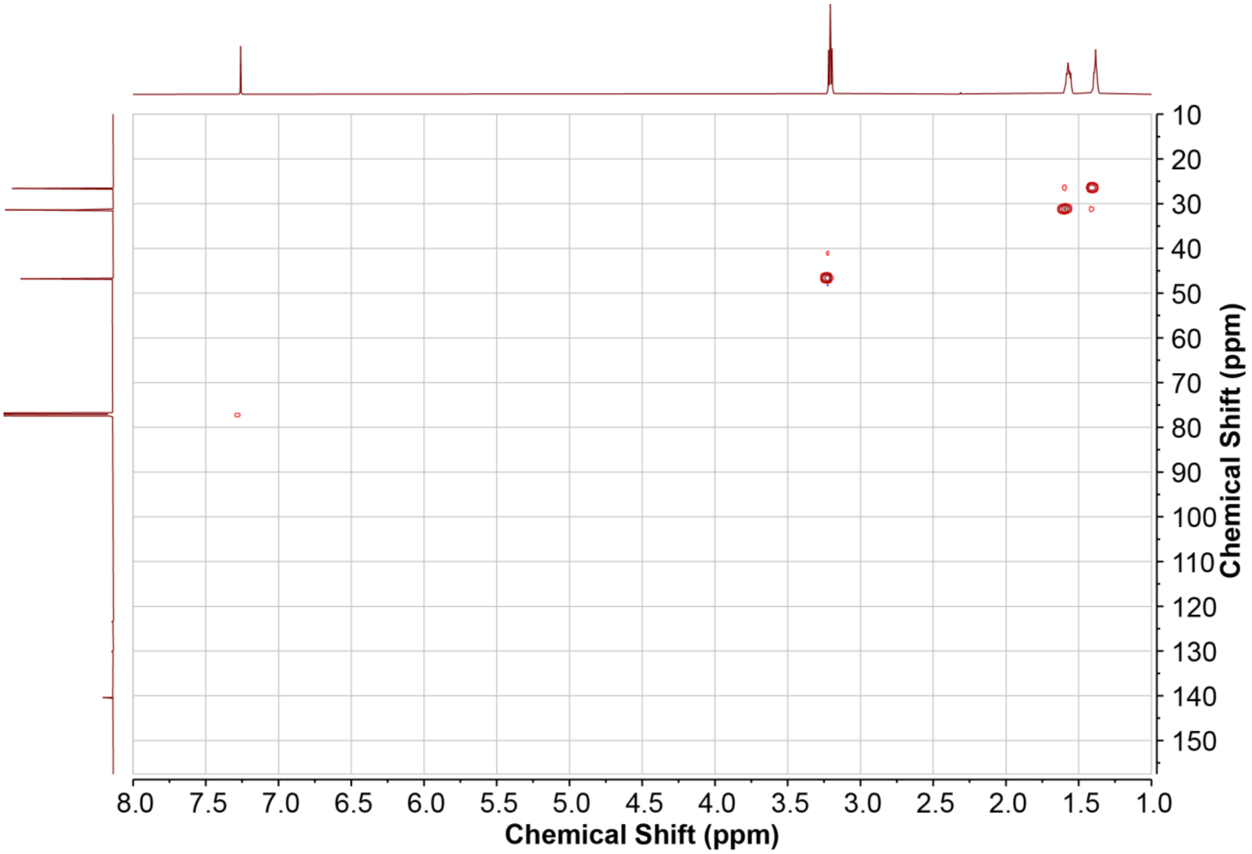

Figure S67. ^{1}H-^{13}C HSQC (^{1}H: 600 MHz, ^{13}C: 150 MHz, $CDCl_3$, 23 °C) spectrum of **polyM2** following precipitation into hexanes and centrifugation (10 min, 4000 rpm).

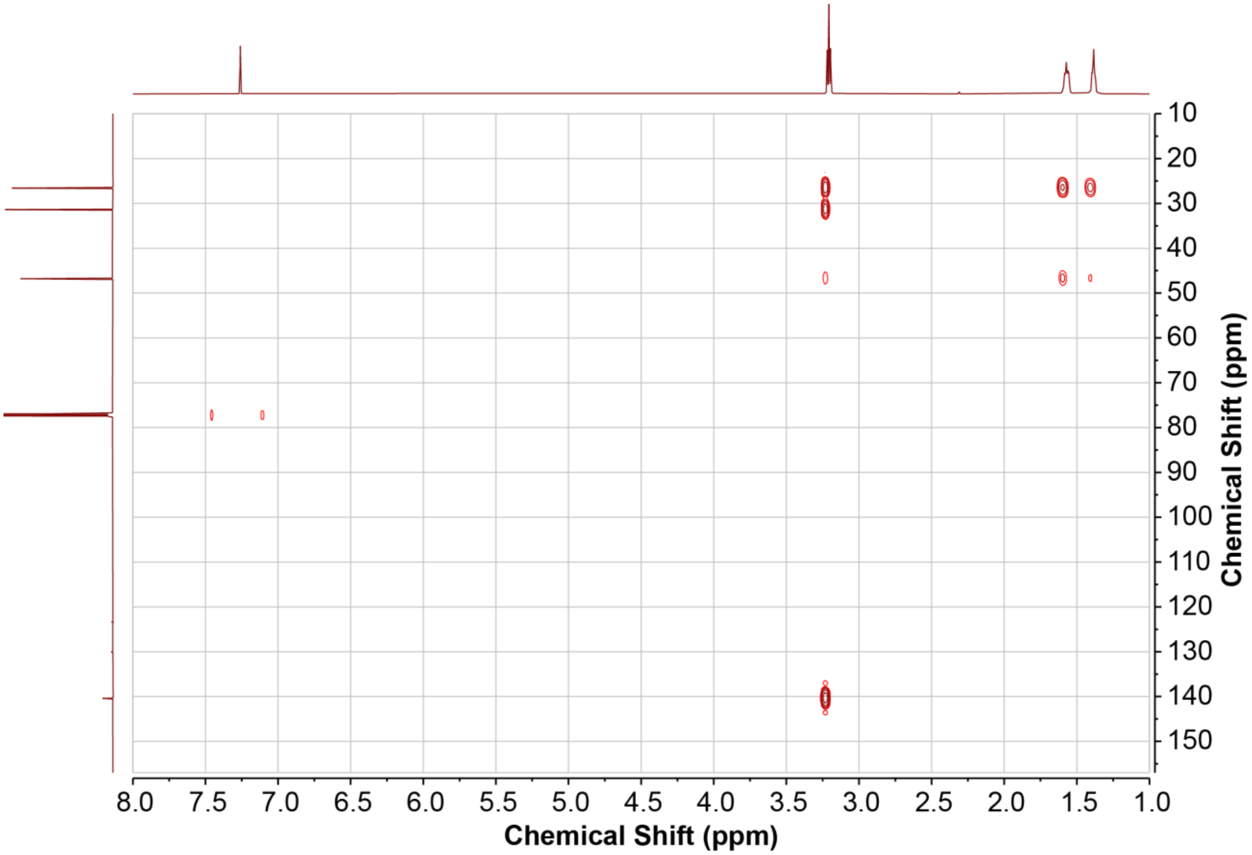

Figure S68. ^{1}H-^{13}C HMBC (^{1}H: 600 MHz, ^{13}C: 150 MHz, $CDCl_3$, 23 °C) spectrum of **polyM2** following precipitation into hexanes and centrifugation (10 min, 4000 rpm).

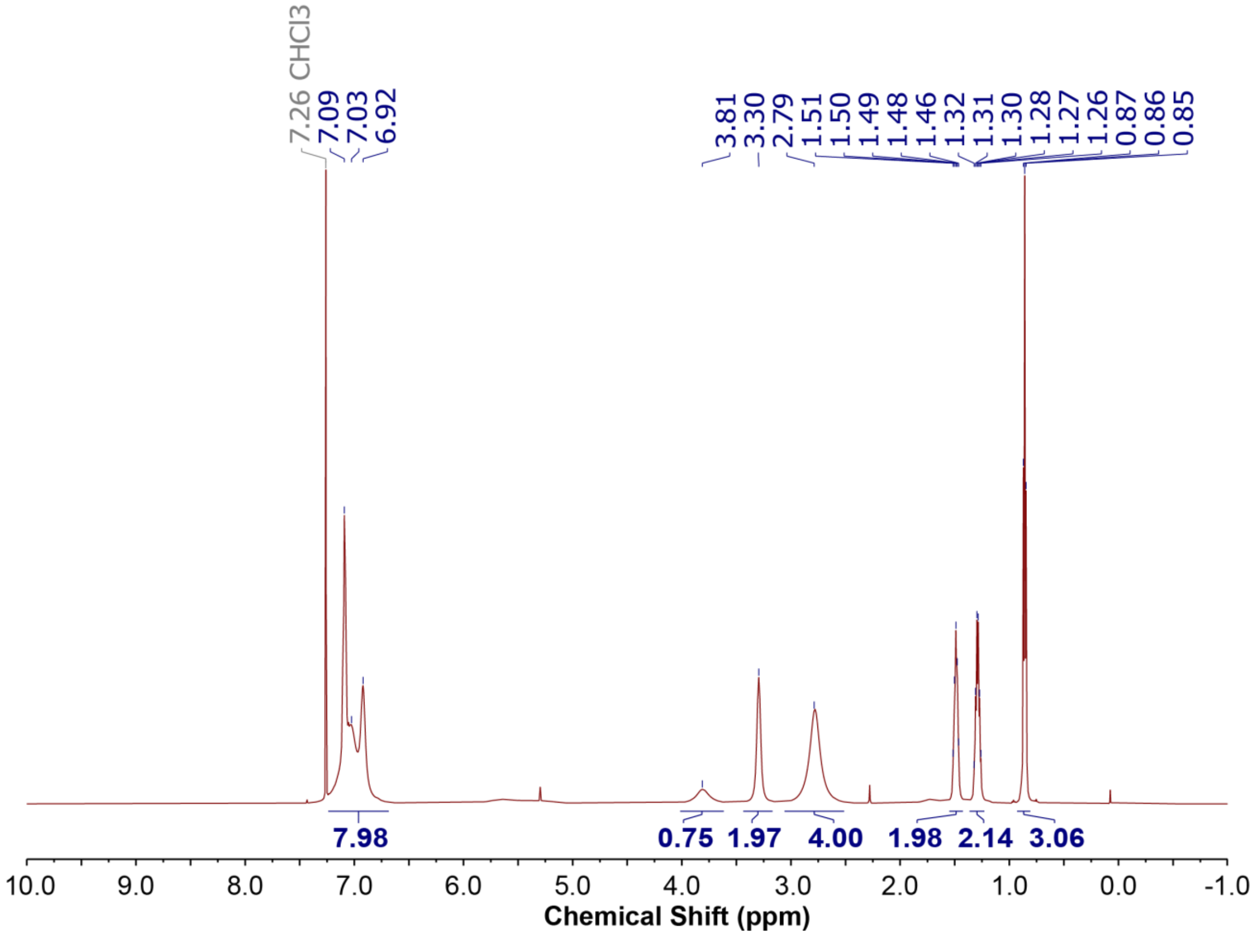

Figure S69. ^{1}H NMR (500 MHz, $CDCl_3$, 23 °C) spectrum of **polyM1-N**.

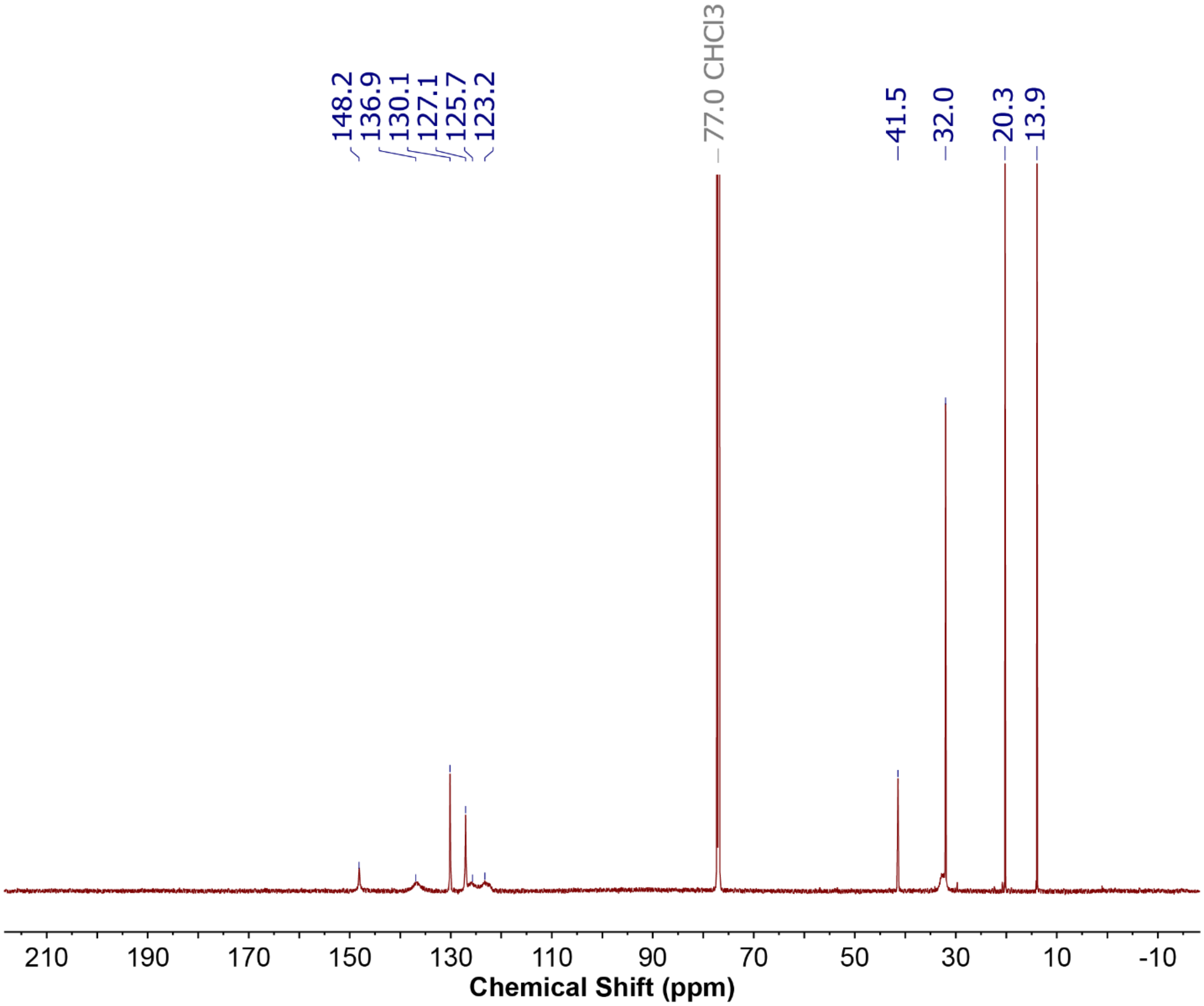

Figure S70. ^{13}C NMR (100 MHz, $CDCl_3$, 23 °C) spectrum of **polyM1-N**.

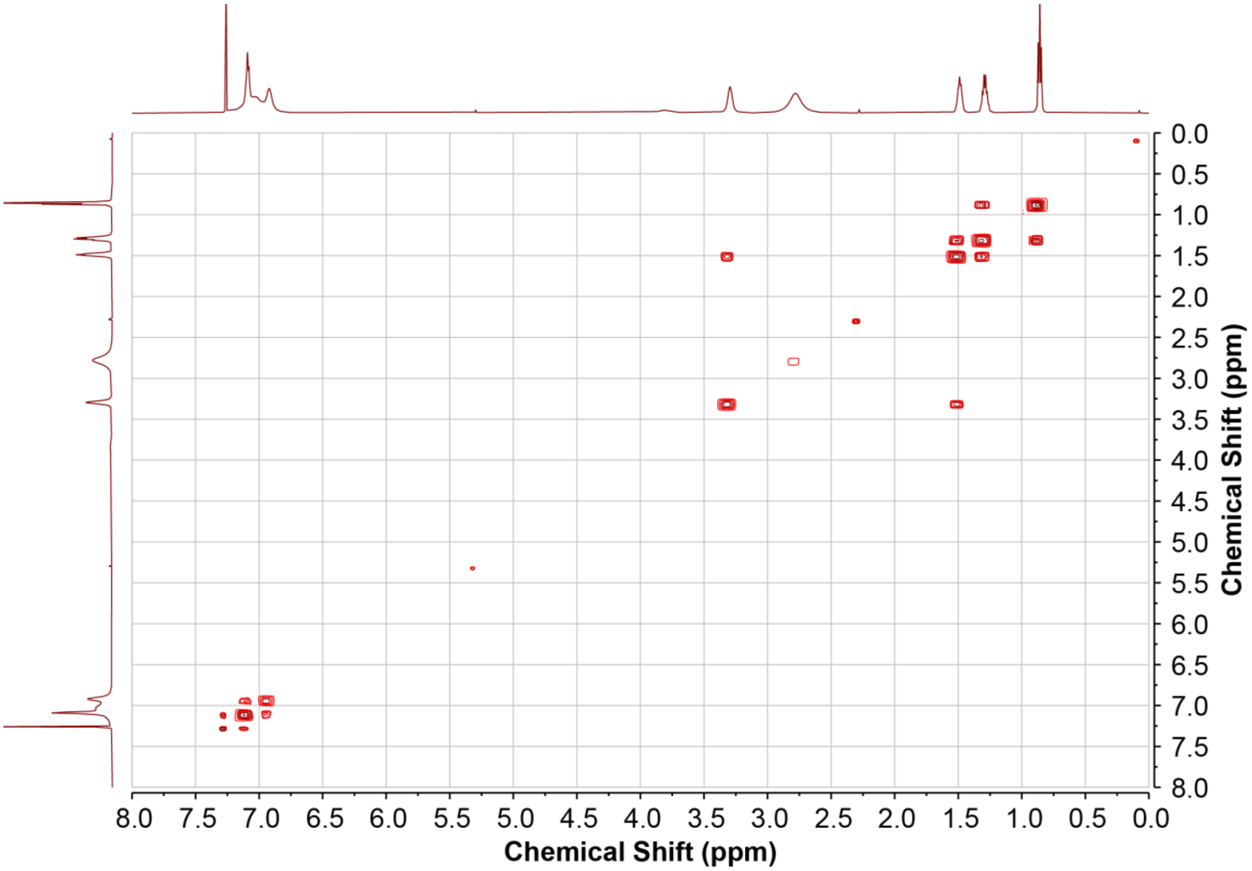

Figure S71. ^1H-^1H COSY (600 MHz, $CDCl_3$, 23 °C) spectrum of **polyM1-N**.

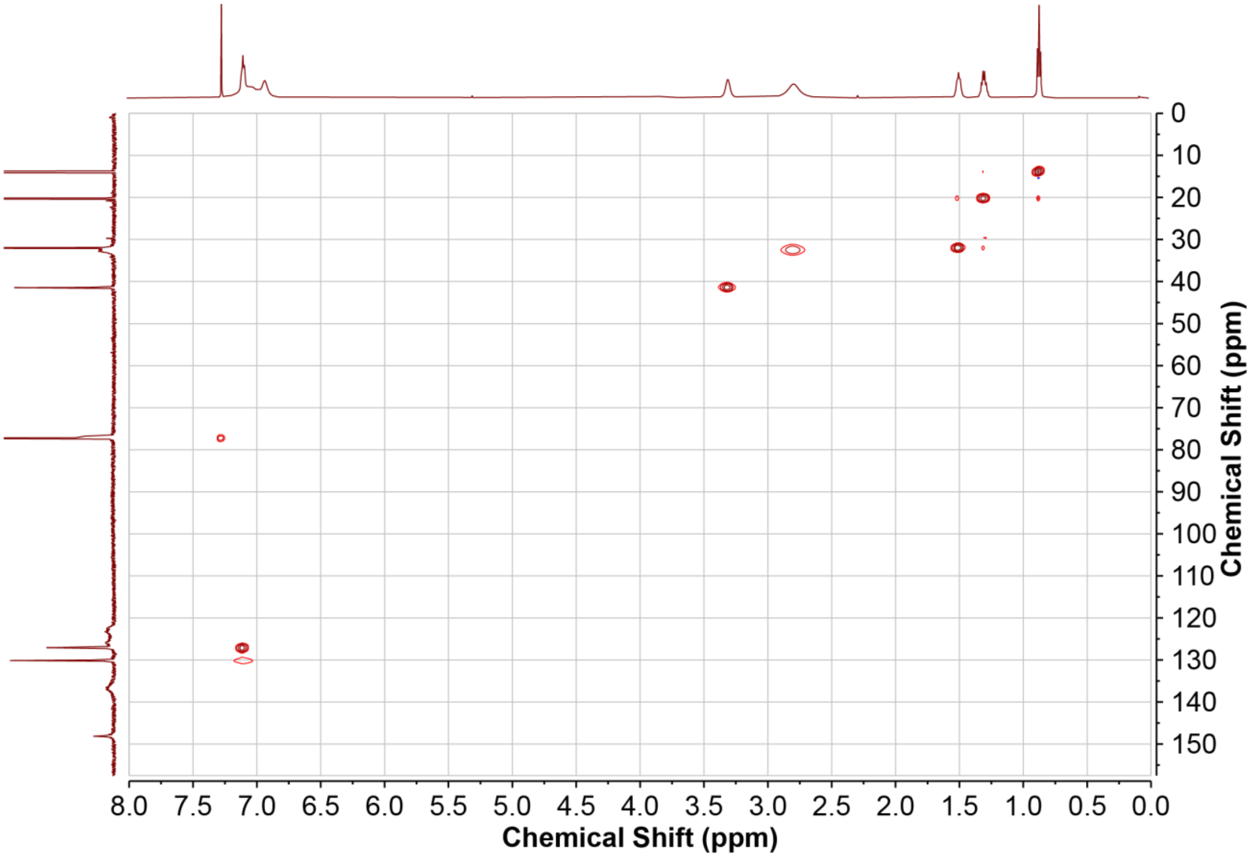

Figure S72. ^{1}H-^{13}C HSQC (^{1}H: 600 MHz, ^{13}C: 150 MHz, $CDCl_3$, 23 °C) spectrum of **polyM1-N**.

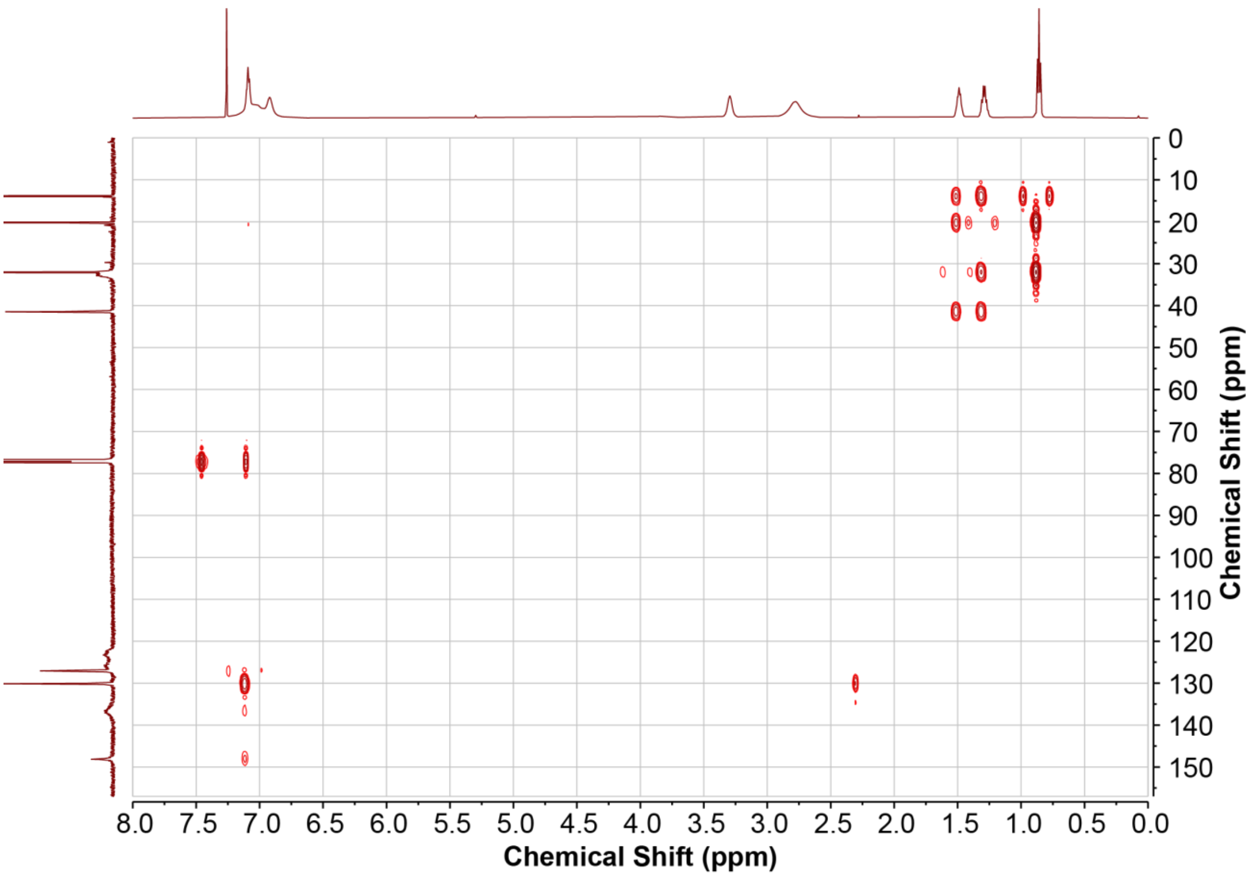

Figure S73. ^{1}H-^{13}C HMBC (^{1}H: 600 MHz, ^{13}C: 150 MHz, $CDCl_3$, 23 °C) spectrum of **polyM1-N**.

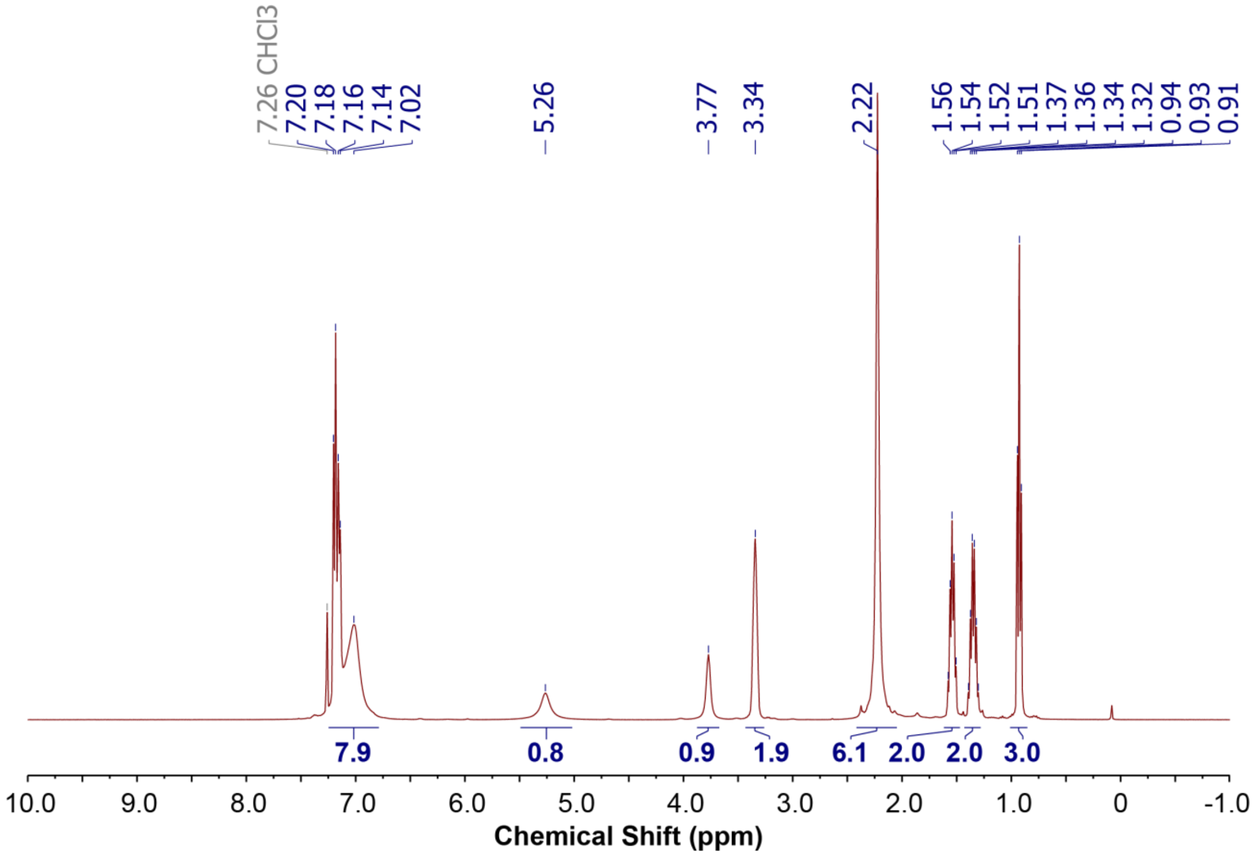

Figure S74. ^{1}H NMR (400 MHz, $CDCl_3$, 23 °C) of **L2-N**.

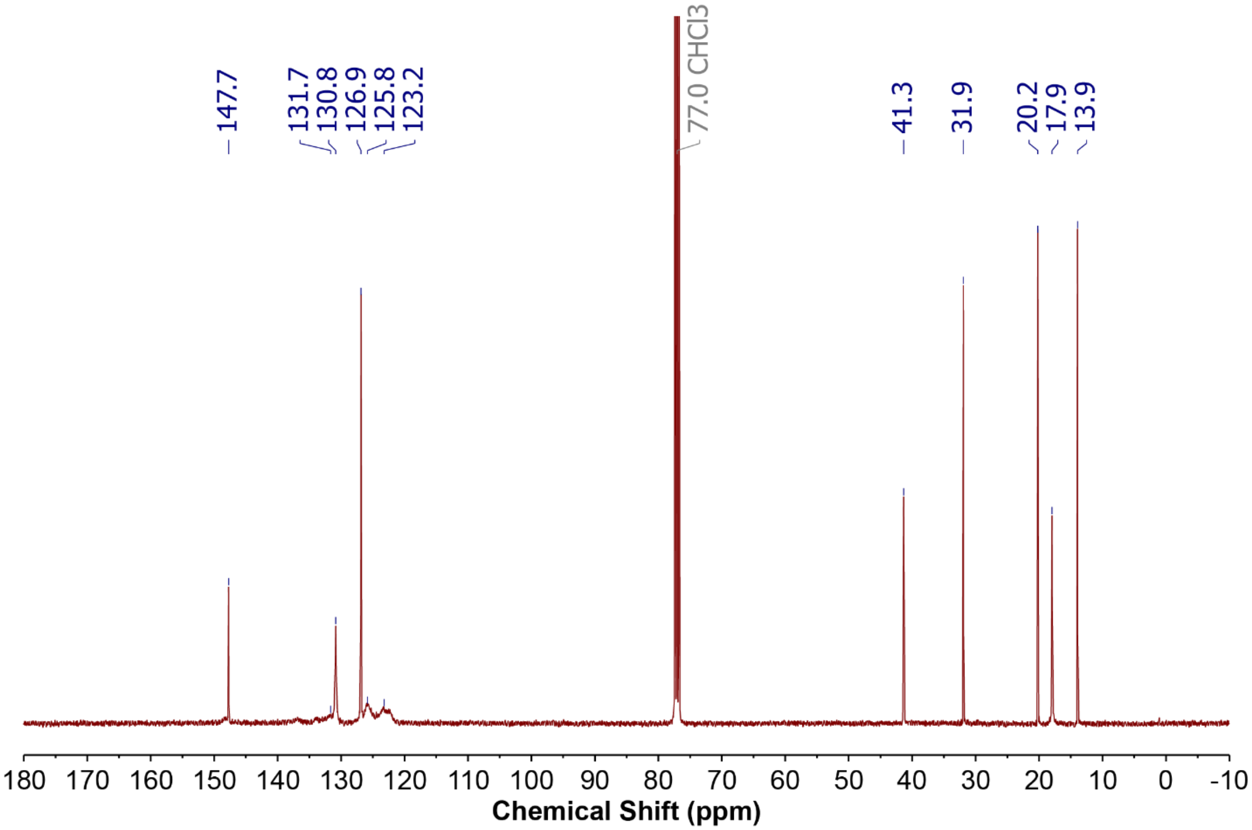

Figure S75. ^{13}C NMR (100 MHz, $CDCl_3$, 23 °C) of **L2-N**.

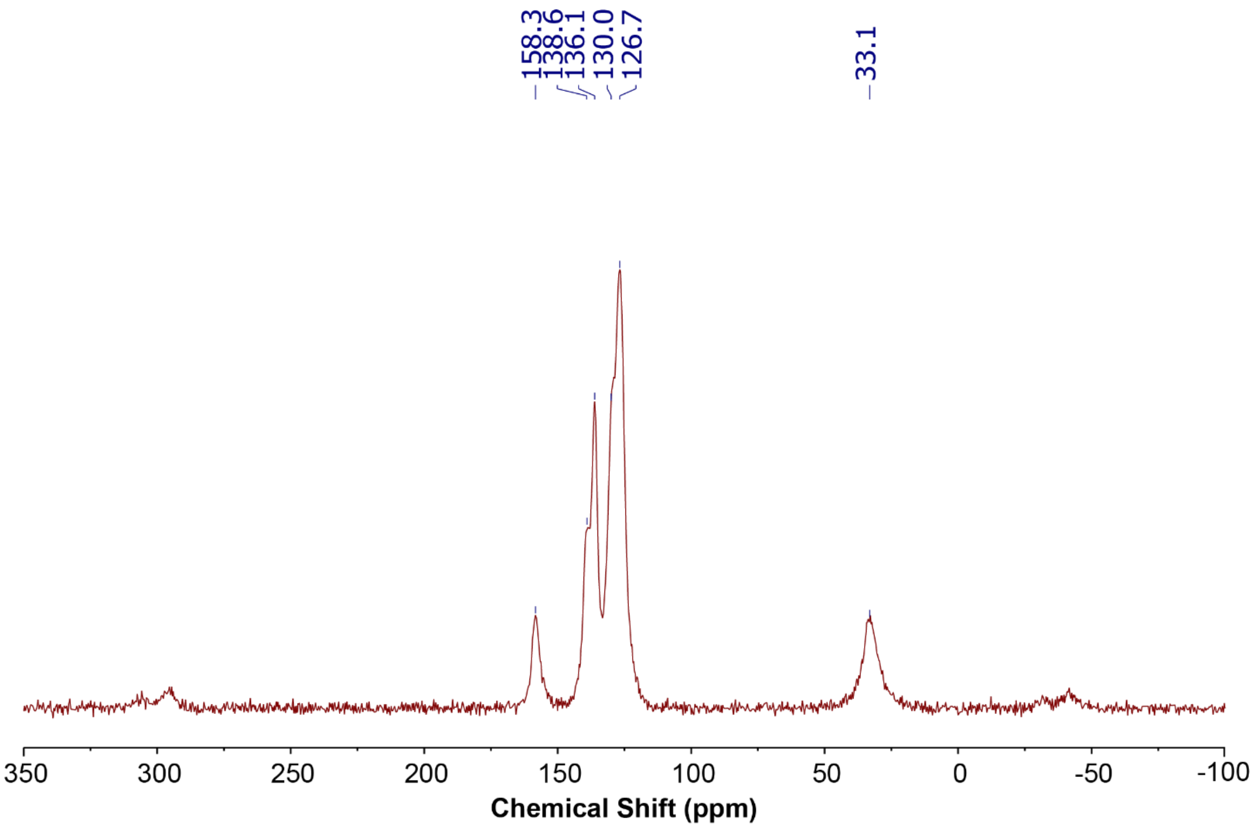

Figure S76. ^{13}C ssNMR CP/MAS (100 MHz, 12 kHz) spectrum of **polyM1-O**.

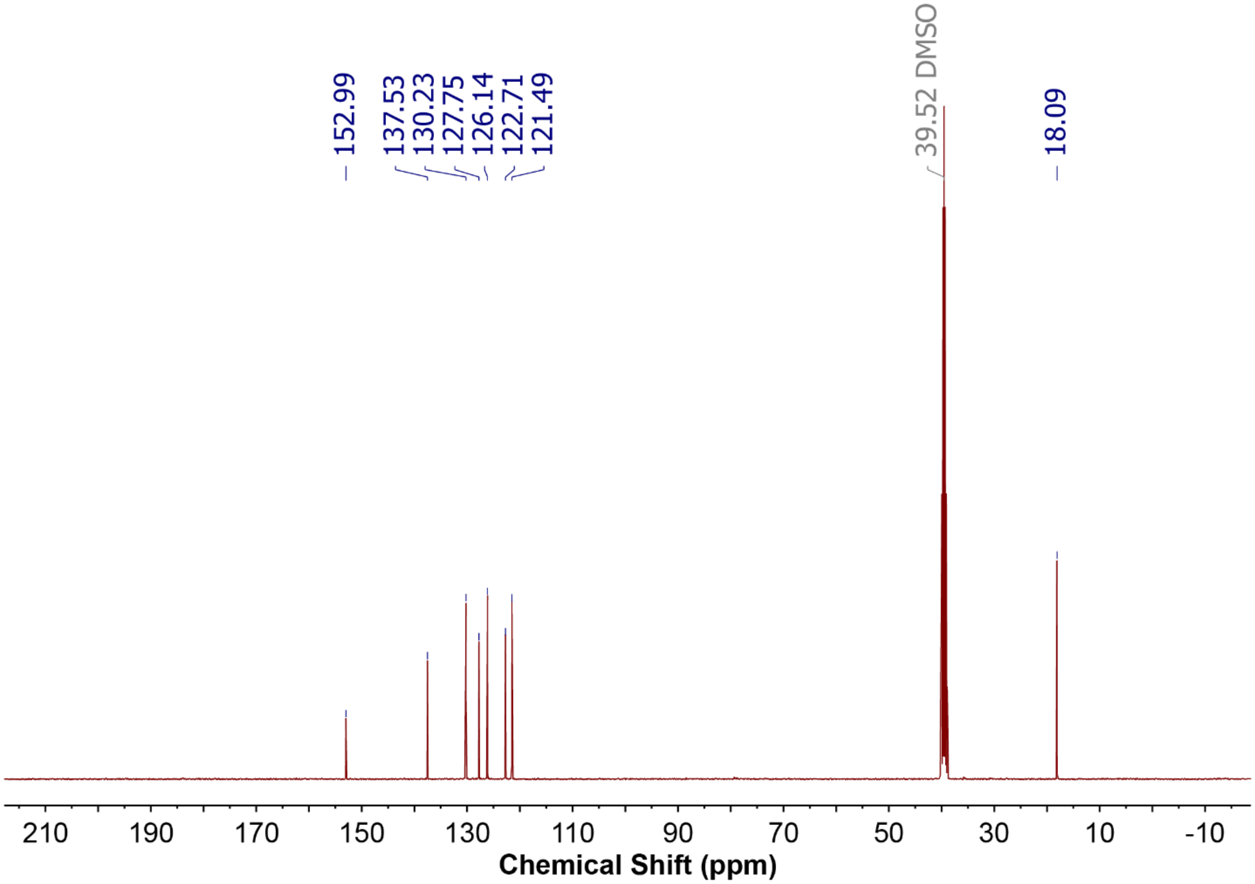

Figure S77. ^{13}C NMR (100 MHz, $CDCl_3$, 23 °C) of **L2-O**.

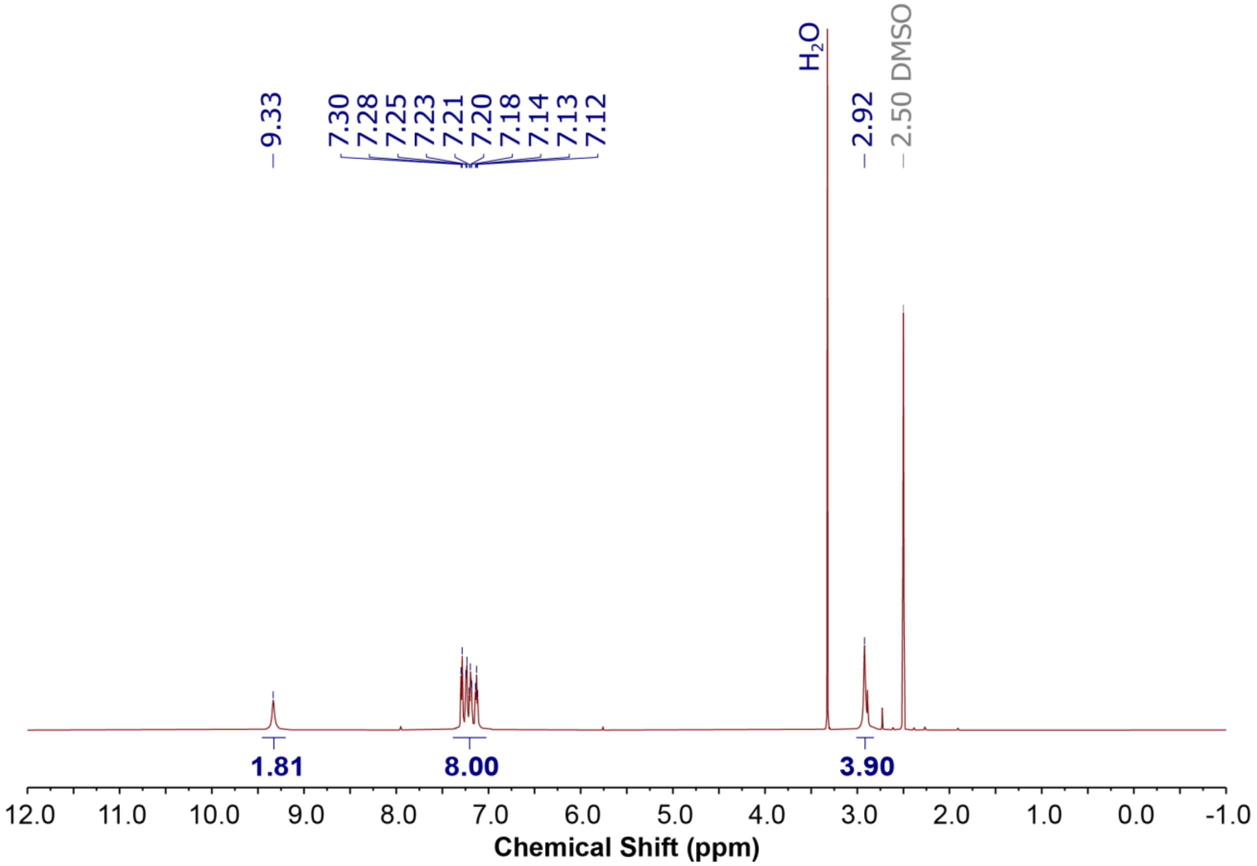

Figure S78. ^{1}H NMR (600 MHz, DMSO-d_6, 23 °C) of **polyM1-S**.

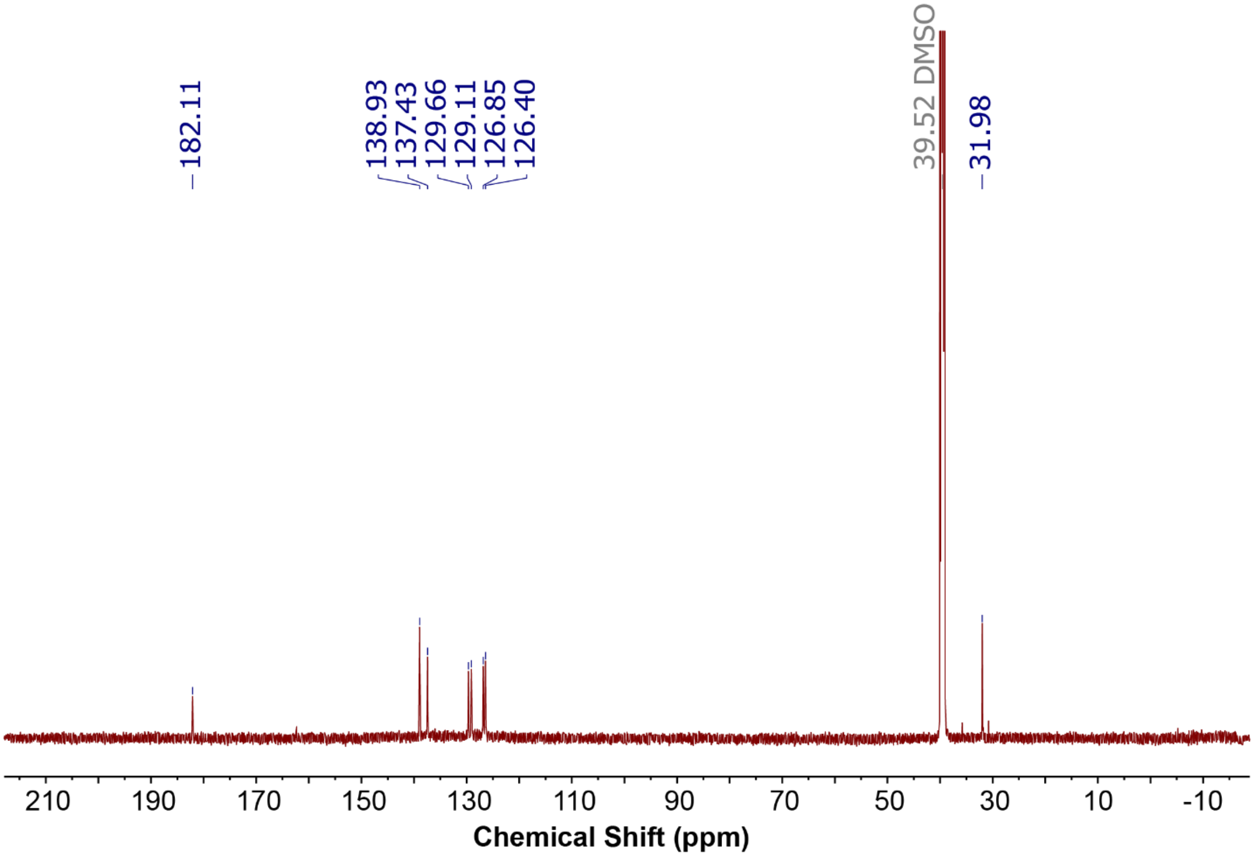

Figure S79. ^{13}C NMR (150 MHz, DMSO-d_6, 23 °C) spectrum of **polyM1-S**.

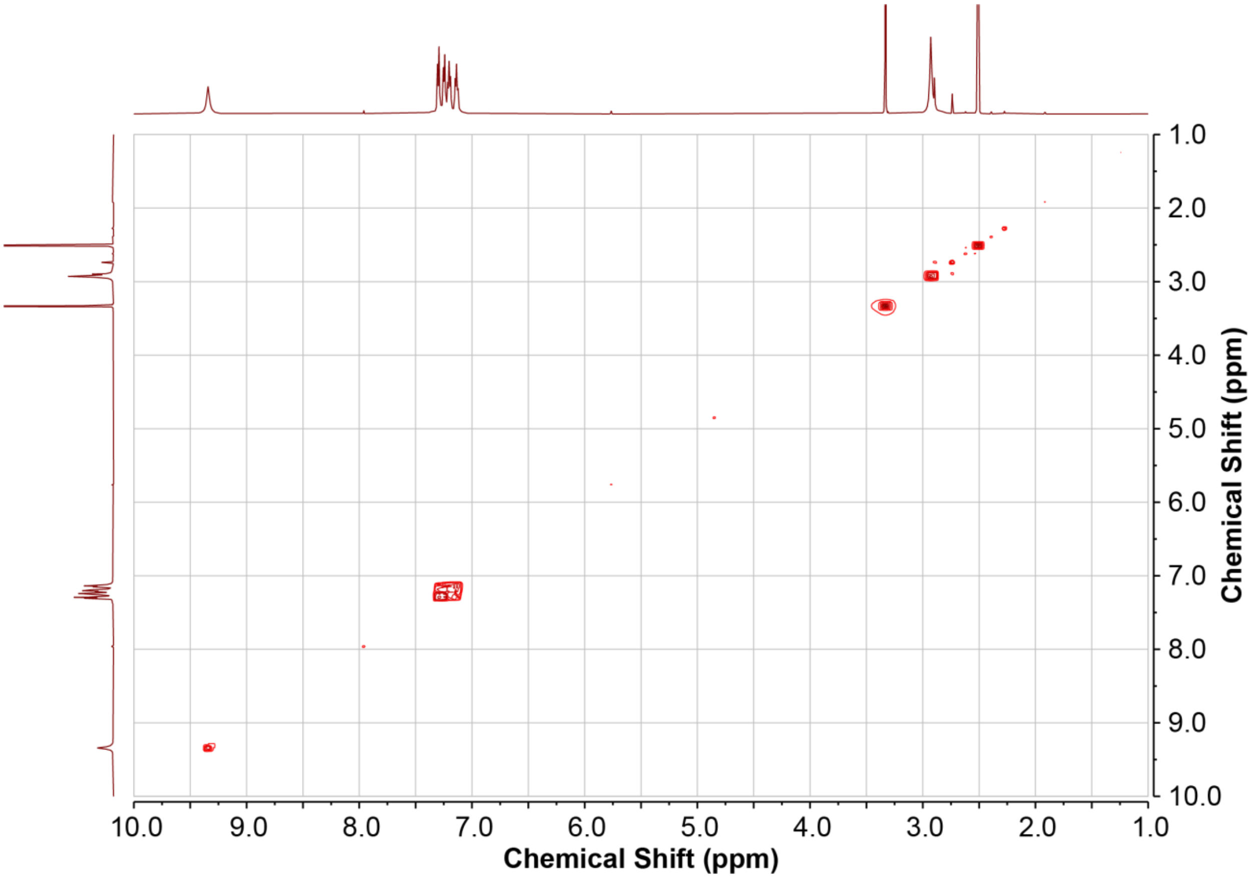

Figure S80. ^{1}H-^{1}H COSY (600 MHz, DMSO-d_6, 23 °C) spectrum of **polyM1-S**.

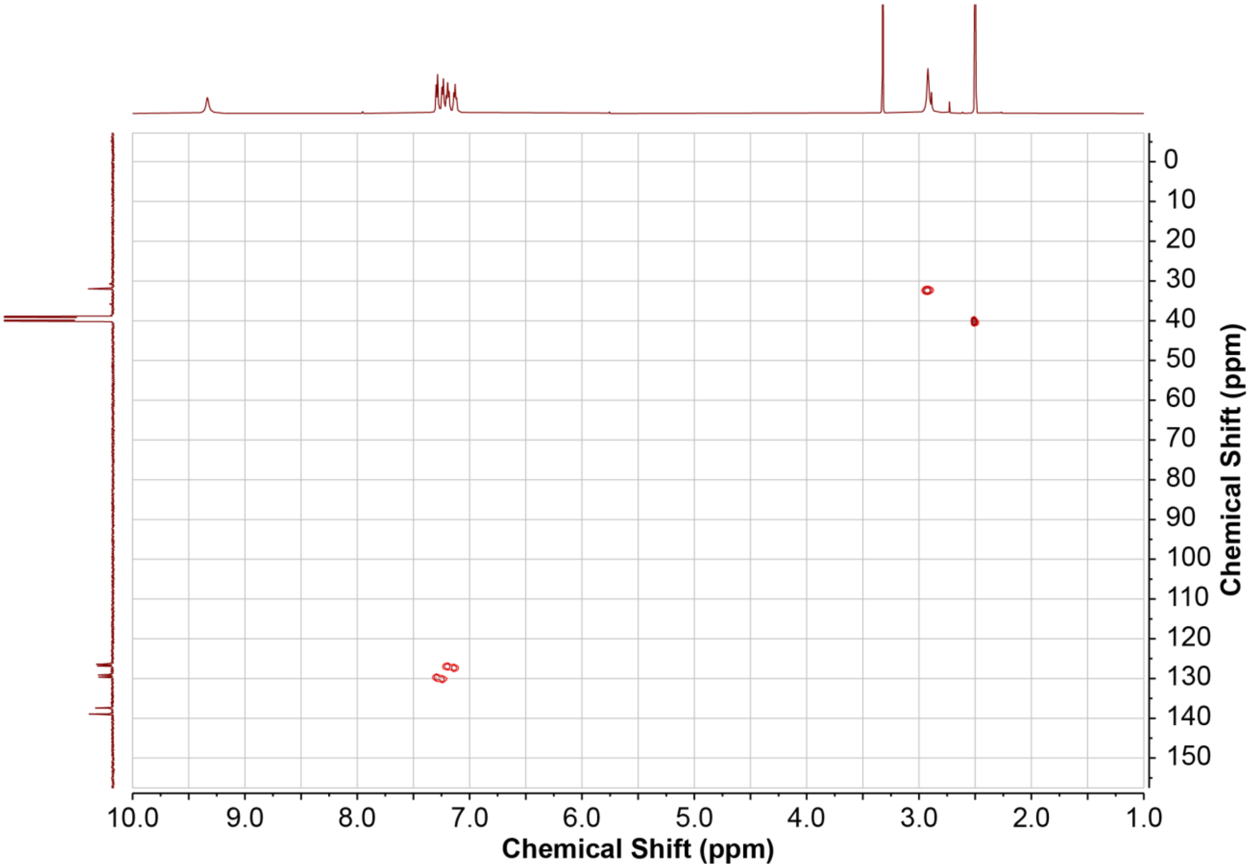

Figure S81. ^{1}H-^{13}C HSQC (^{1}H: 600 MHz, ^{13}C: 150 MHz, DMSO-d_6, 23 °C) spectrum of **polyM1-S.**

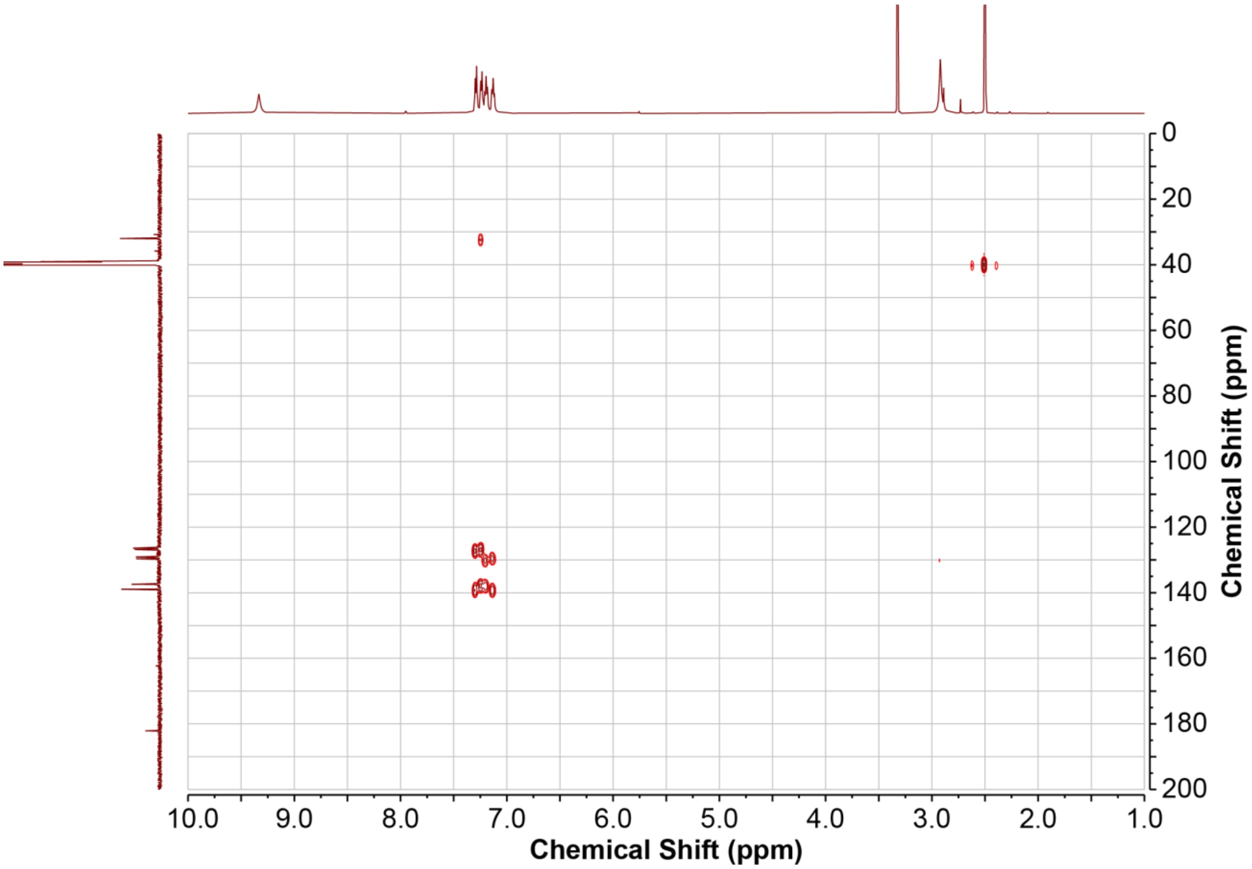

Figure S82. ^{1}H-^{13}C HMBC (^{1}H: 600 MHz, ^{13}C: 150 MHz, DMSO-d_6, 23 °C) spectrum of **polyM1-S.**

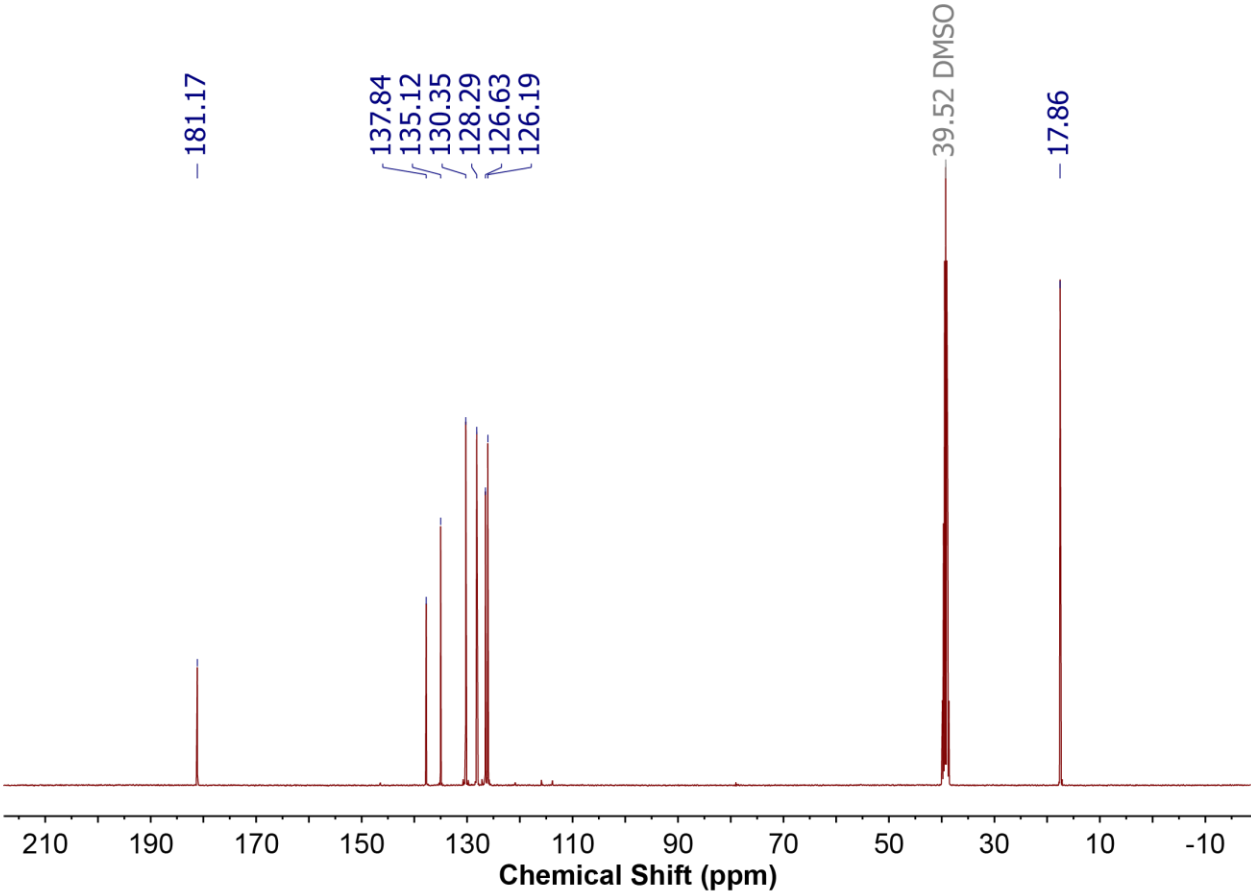

Figure S83. ^{13}C NMR (150 MHz, DMSO-d_6, 23 °C) spectrum of **L2-S**.

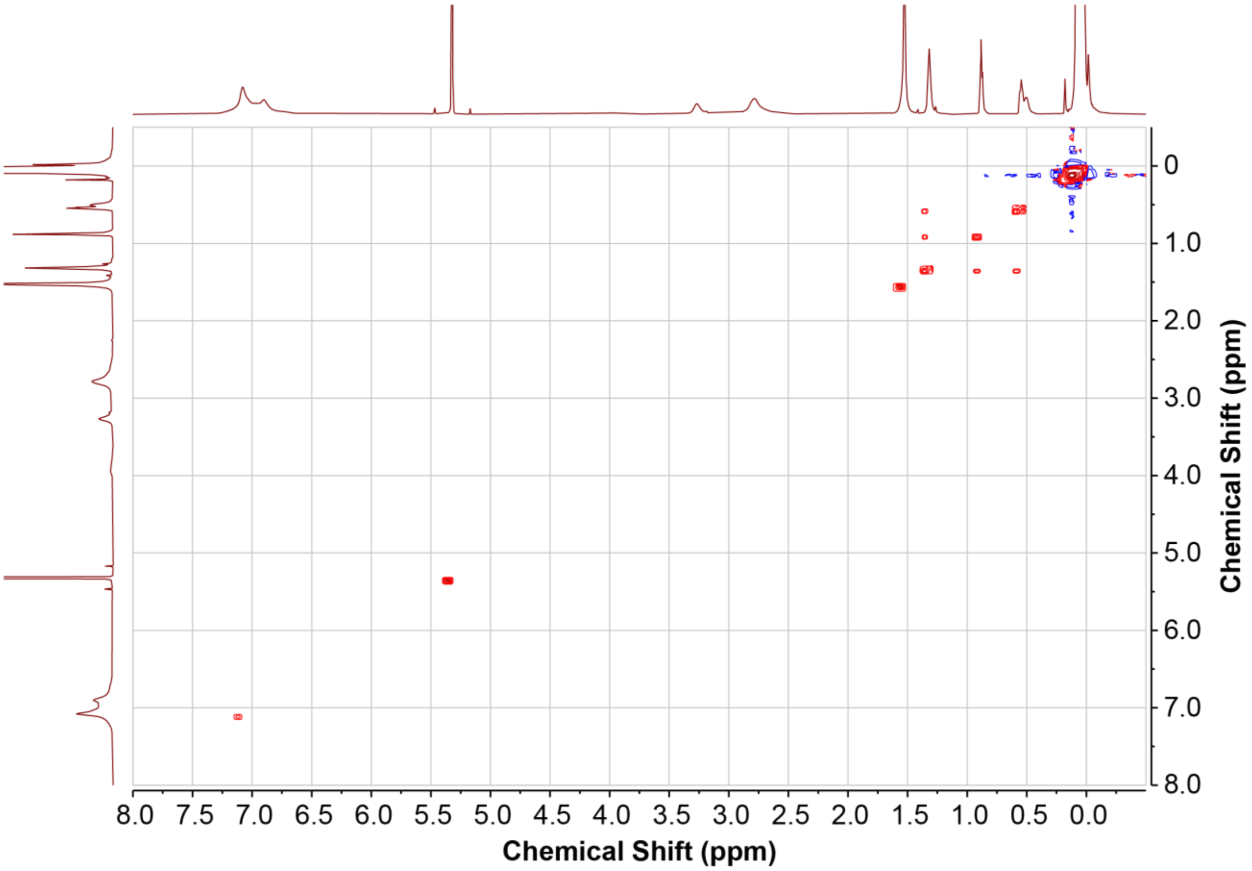

Figure S84. ${}^{1}H$-${}^{1}H$ COSY (600 MHz, $CDCl_3$, 23 °C) spectrum of **polyM1-BB**.

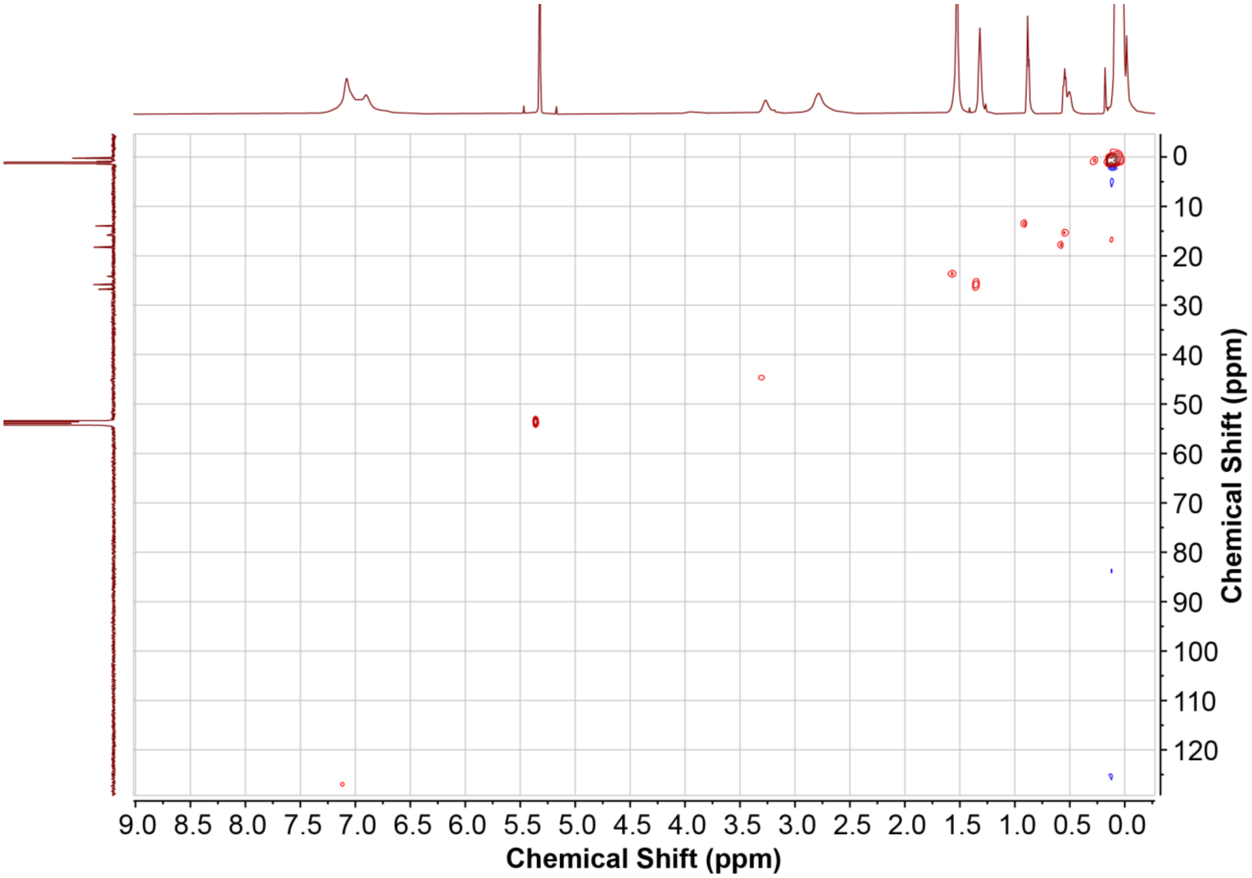

Figure S85. ^{1}H-^{13}C HSQC (^{1}H: 600 MHz, ^{13}C: 150 MHz, $CDCl_3$, 23 °C) spectrum of **polyM1-BB.**

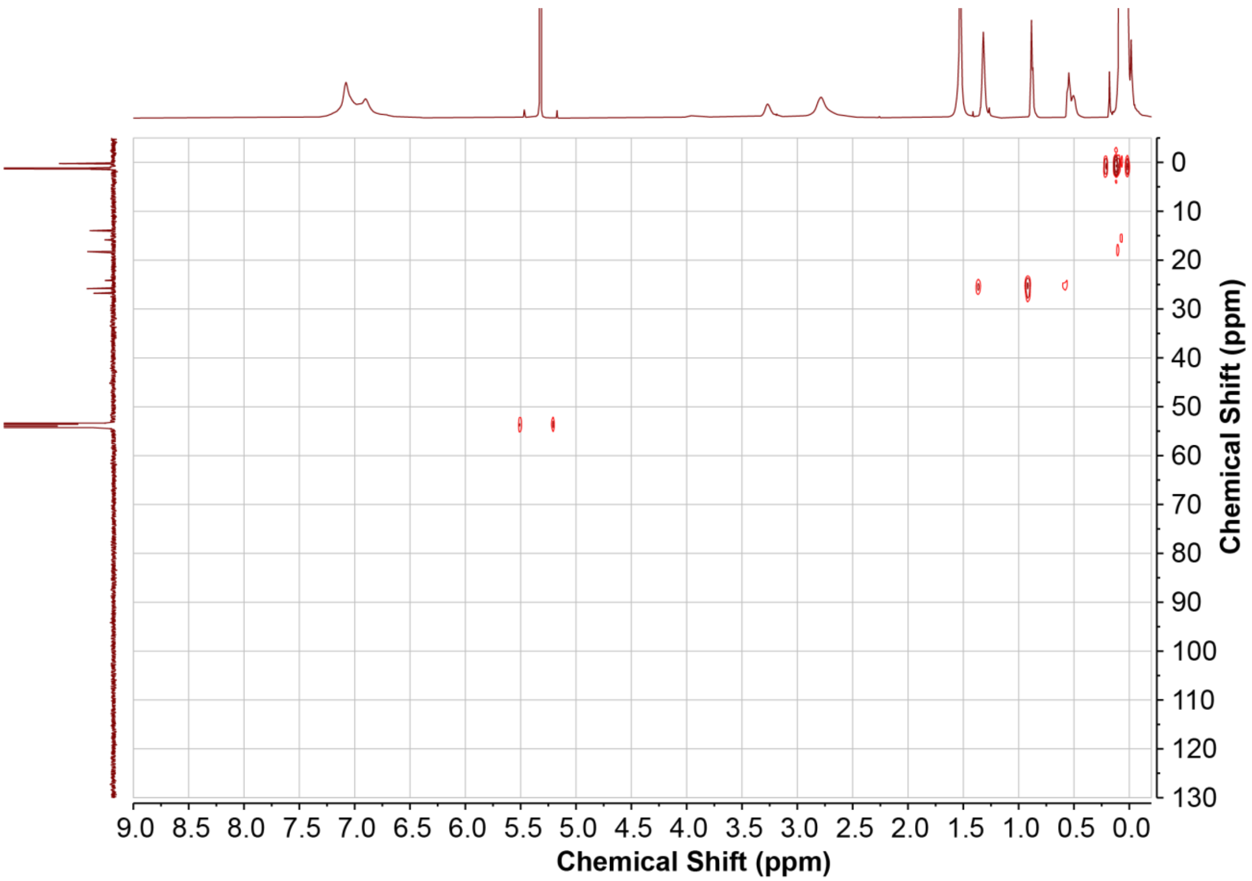

Figure S86. ^1H-^{13}C HMBC (^1H: 600 MHz, ^{13}C: 150 MHz, $CDCl_3$, 23 °C) spectrum of **polyM1-BB.**

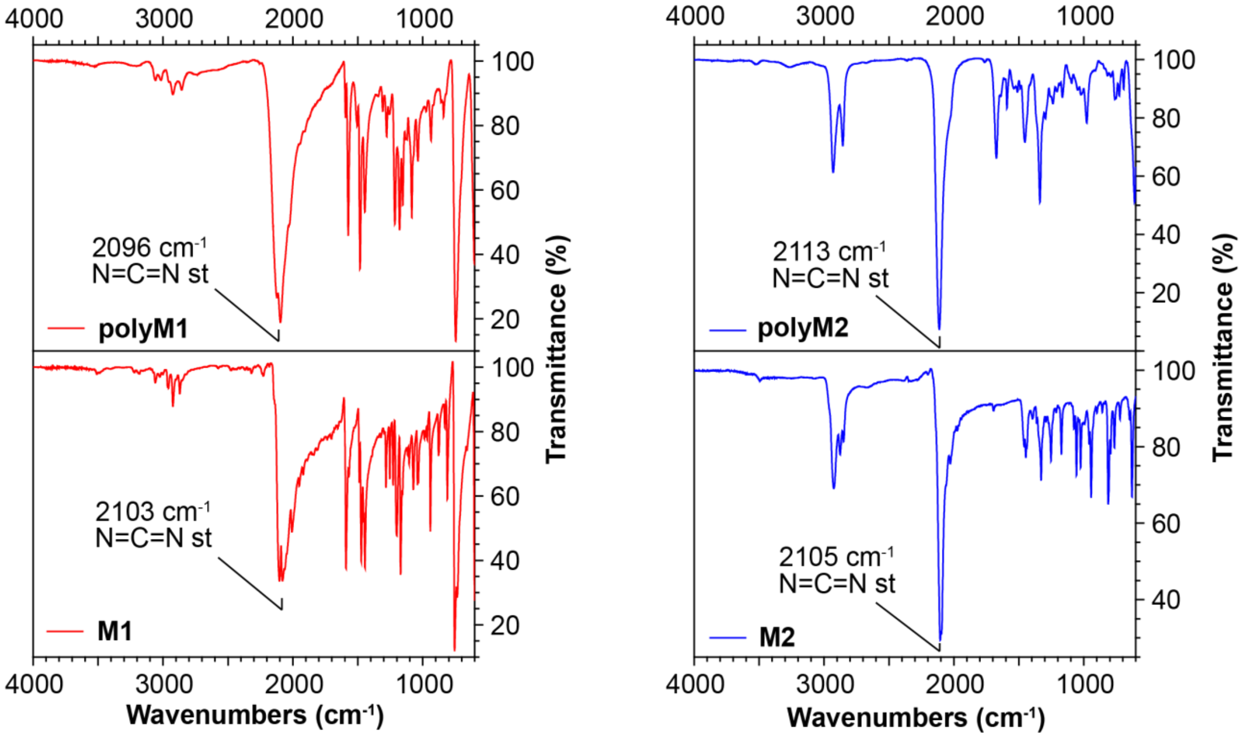

Figure S87. ATR-FTIR spectra of **polyM1** and **polyM2**, compared to monomers **M1** and **M2**, with notable features labeled with a line.

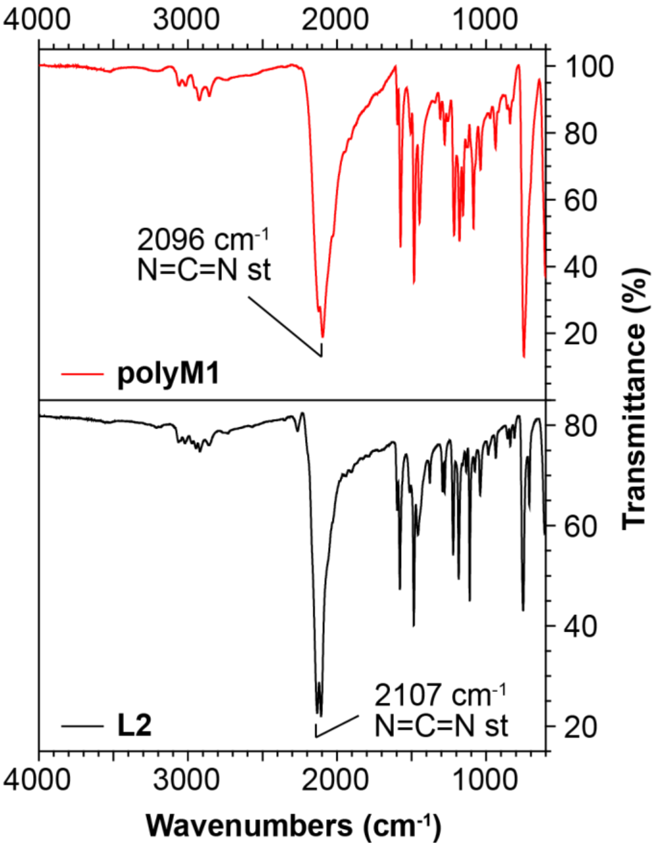

Figure S88. IR spectrum of **polyM1** and di-*o*-tolyl CDI **L2**, its small molecule model. Notable features are labeled with a line.

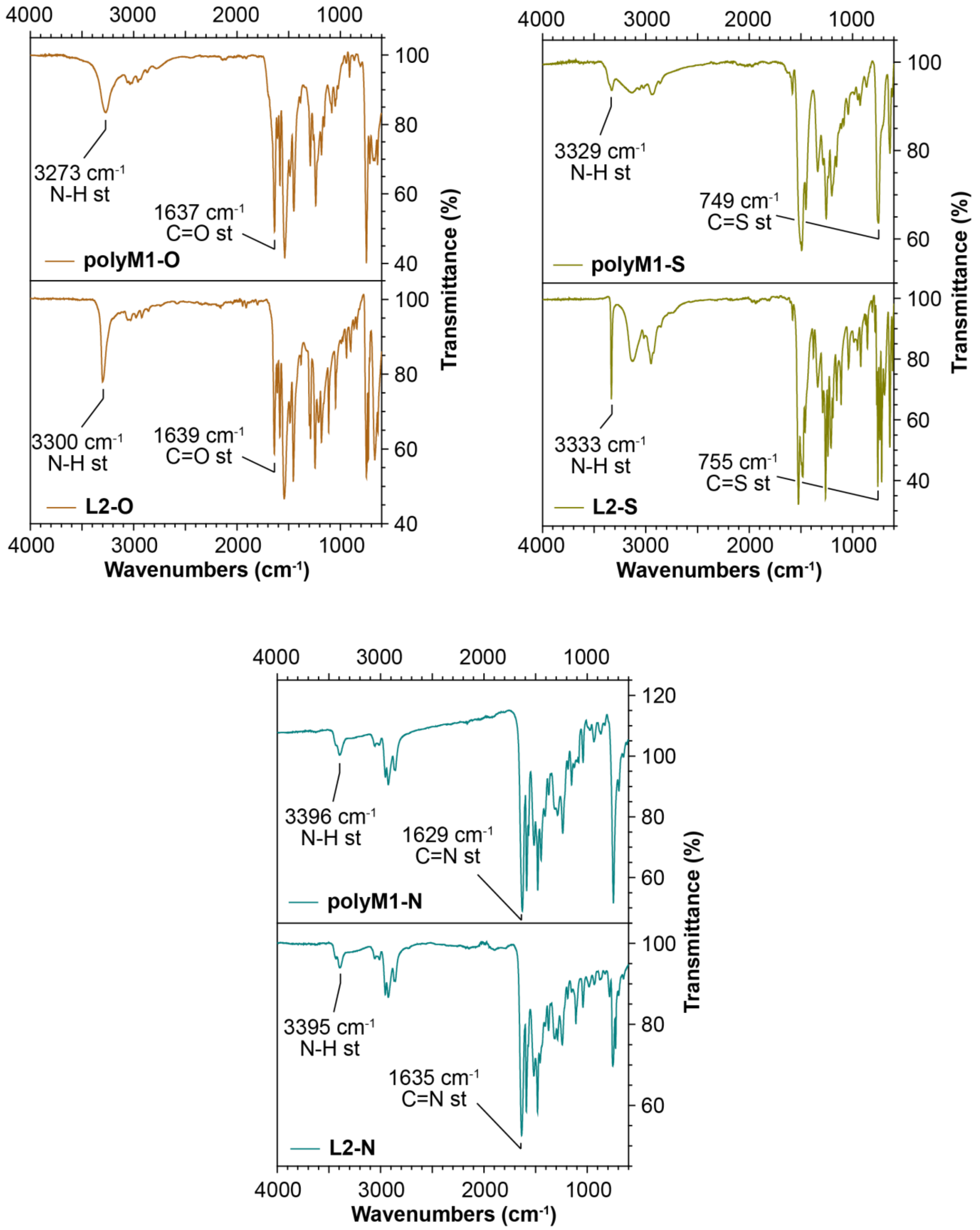

Figure S89. ATR-FTIR spectra of **polyM1** derivatives and the corresponding small molecule models of these polymers, with notable features labeled with a line.

THERMAL PROPERTY CHARACTERIZATION

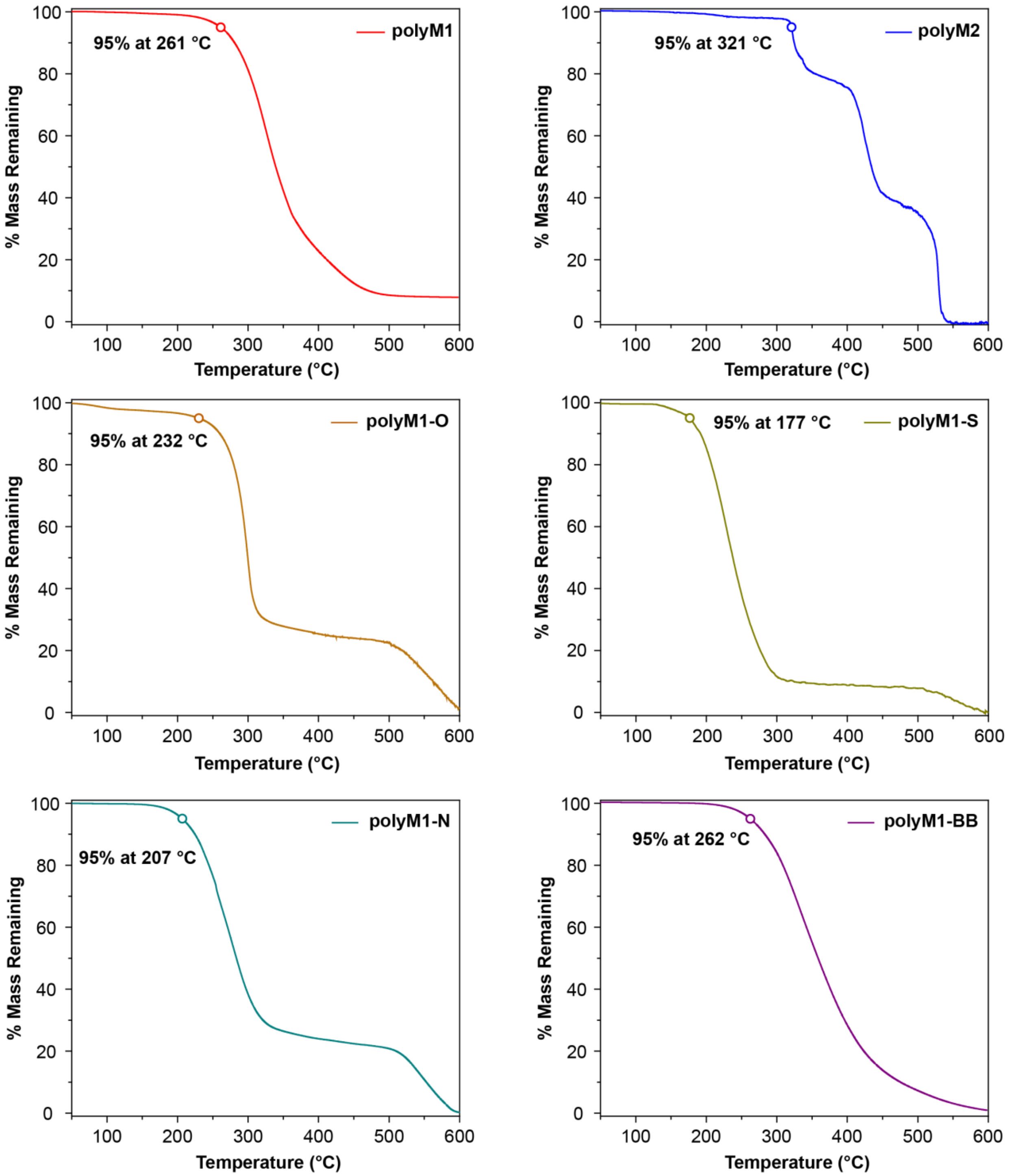

Figure S90. Individual TGA data of **polyM1**, its derivatives, and **polyM2**, with the observed 5% decomposition temperatures labeled as circles.

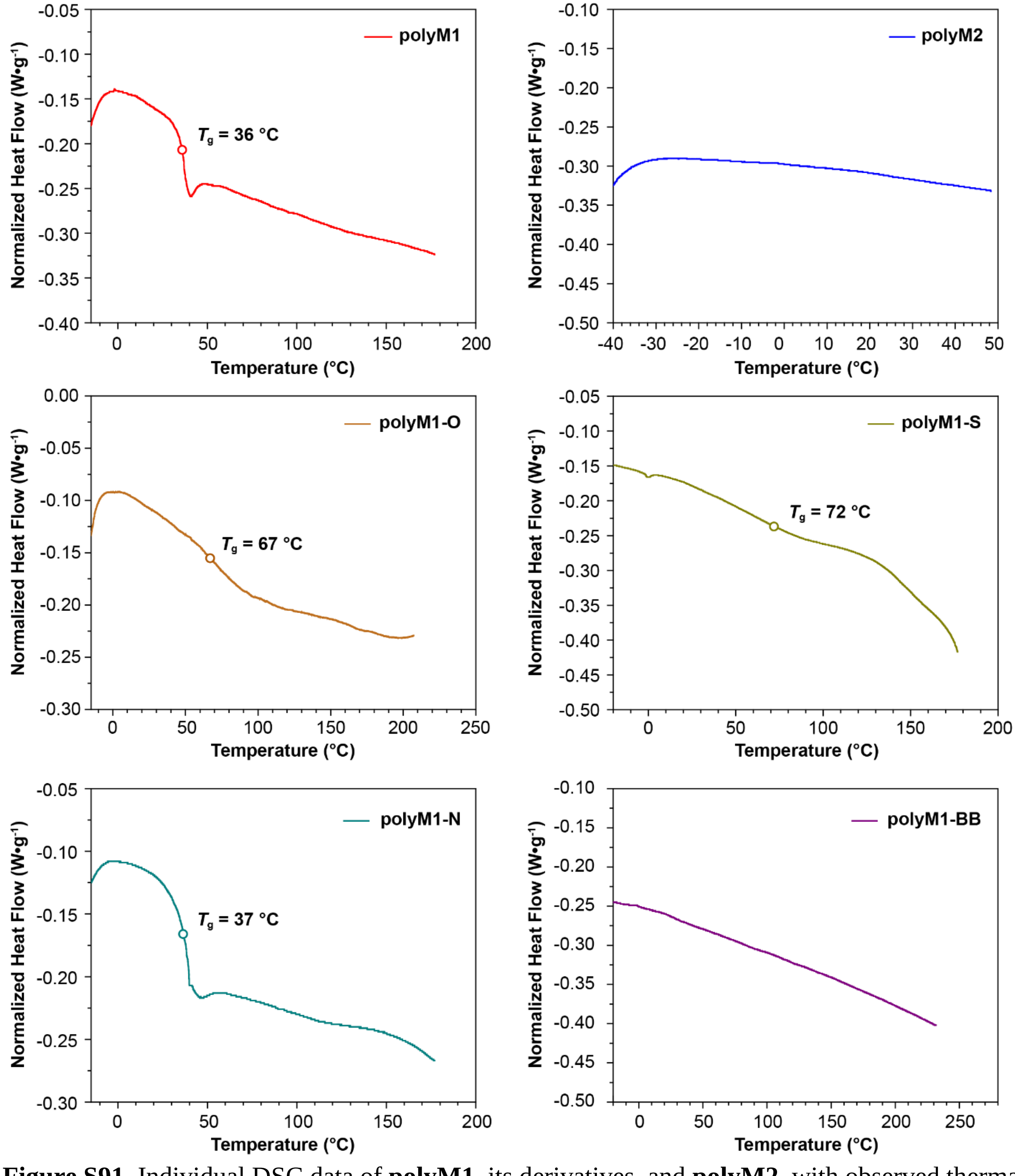

Figure S91. Individual DSC data of **polyM1**, its derivatives, and **polyM2**, with observed thermal transitions labeled as circles.

CRYSTALLOGRAPHIC DATA

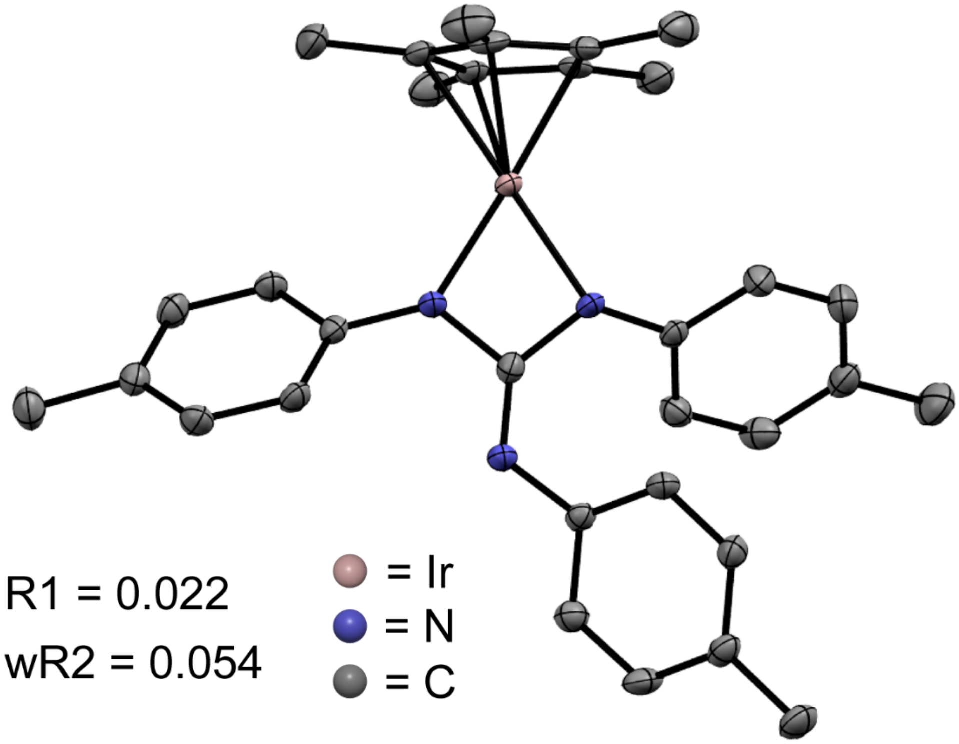

Figure S92. ORTEP plot at 50% probability of **1**, with H atoms omitted for clarity.

REFERENCES

(1) Pangborn, A. B.; Giardello, M. A.; Grubbs, R. H.; Rosen, R. K.; Timmers, F. J. Safe and Convenient Procedure for Solvent Purification. *Organometallics* **1996**, *15* (5), 1518–1520. https://doi.org/10.1021/om9503712.

(2) Neumann, W.; Fisher, P. The Preparation of Carbodiimides from Isocyantes. *Angew. Chem. Int. Ed.* **1962**. https://doi.org/10.1002/anie.196206211.

(3) Fulmer, G. R.; Miller, A. J. M.; Sherden, N. H.; Gottlieb, H. E.; Nudelman, A.; Stoltz, B. M.; Bercaw, J. E.; Goldberg, K. I. NMR Chemical Shifts of Trace Impurities: Common Laboratory Solvents, Organics, and Gases in Deuterated Solvents Relevant to the Organometallic Chemist. *Organometallics* 2010, *29* (9), 2176–2179. https://doi.org/10.1021/om100106e.

(4) Bruker Analytical X-Ray Systems, n.d.

(5) Blessing, R. H. An Empirical Correction for Absorption Anisotropy. *Acta Crystallogr A Found Crystallogr* **1995**, *51* (1), 33–38. https://doi.org/10.1107/S0108767394005726.

(6) Palatinus, L.; Chapuis, G. *SUPERFLIP* – a Computer Program for the Solution of Crystal Structures by Charge Flipping in Arbitrary Dimensions. *J. Appl. Crystallogr.* **2007**, *40* (4), 786–790. https://doi.org/10.1107/S0021889807029238.

(7) Betteridge, P. W.; Carruthers, J. R.; Cooper, R. I.; Prout, K.; Watkin, D. J. CRYSTALS Version 12: Software for Guided Crystal Structure Analysis . *J. Appl. Crystallogr.* **2003**, *36* (6), 1487–1487. https://doi.org/10.1107/s0021889803021800.

(8) Gaussian 16, Revision C.01.

(9) Hopmann, K. H. How Accurate Is DFT for Iridium-Mediated Chemistry? *Organometallics* **2016**, *35* (22), 3795–3807. https://doi.org/10.1021/acs.organomet.6b00377.

(10) Zhao, Y.; Truhlar, D. G. The M06 Suite of Density Functionals for Main Group Thermochemistry, Thermochemical Kinetics, Noncovalent Interactions, Excited States, and Transition Elements: Two New Functionals and Systematic Testing of Four M06-Class Functionals and 12 Other Functionals. *Theor. Chem. Account* **2008**, *120* (1–3), 215–241. https://doi.org/10.1007/s00214-007-0310-x.

(11) Clark, T.; Chandrasekhar, J.; Spitznagel, G. W.; Schleyer, P. V. R. Efficient Diffuse Function-Augmented Basis Sets for Anion Calculations. III. The 3-21+G Basis Set for First-Row Elements, Li-F. *J. Comput. Chem.* **1983**, *4* (3), 294–301. https://doi.org/10.1002/jcc.540040303.

(12) Hay, P. J.; Wadt, W. R. *Ab Initio* Effective Core Potentials for Molecular Calculations. Potentials for K to Au Including the Outermost Core Orbitals. *J. Chem. Phys.* **1985**, *82* (1), 299–310. https://doi.org/10.1063/1.448975.

(13) Perdew, J. P.; Burke, K.; Ernzerhof, M. Generalized Gradient Approximation Made Simple. *Phys. Rev. Lett.* **1996**, *77* (18), 3865–3868. https://doi.org/10.1103/PhysRevLett.77.3865.

(14) Perdew, J. P.; Burke, K.; Ernzerhof, M. Generalized Gradient Approximation Made Simple [Phys. Rev. Lett. 77, 3865 (1996)]. *Phys. Rev. Lett.* **1997**, *78* (7), 1396–1396. https://doi.org/10.1103/PhysRevLett.78.1396.

(15) Grimme, S. Semiempirical GGA-Type Density Functional Constructed with a Long-Range Dispersion Correction. *J. Comput. Chem.* **2006**, *27* (15), 1787–1799. https://doi.org/10.1002/jcc.20495.

(16) Krishnan, R.; Binkley, J. S.; Seeger, R.; Pople, J. A. Self-consistent Molecular Orbital Methods. XX. A Basis Set for Correlated Wave Functions. *J. Chem. Phys.* **1980**, *72* (1), 650–654. https://doi.org/10.1063/1.438955.

(17) Ehlers, A. W.; Böhme, M.; Dapprich, S.; Gobbi, A.; Höllwarth, A.; Jonas, V.; Köhler, K. F.; Stegmann, R.; Veldkamp, A.; Frenking, G. A Set of F-Polarization Functions for Pseudo-Potential Basis Sets of the Transition Metals Sc–Cu, Y–Ag and La–Au. *Chem. Phys. Lett.* **1993**, *208* (1–2), 111–114. https://doi.org/10.1016/0009-2614(93)80086-5.

(18) Cossi, M.; Rega, N.; Scalmani, G.; Barone, V. Energies, Structures, and Electronic Properties of Molecules in Solution with the C-PCM Solvation Model. *J. Comput. Chem.* **2003**, *24* (6), 669–681. https://doi.org/10.1002/jcc.10189.

(19) Barone, V.; Cossi, M. Quantum Calculation of Molecular Energies and Energy Gradients in Solution by a Conductor Solvent Model. *J. Phys. Chem. A* **1998**, *102* (11), 1995–2001. https://doi.org/10.1021/jp9716997.

(20) Maier, M. S.; Hü, K.; Reynders, M.; Matsuura, B. S.; Leippe, P.; Ko, T.; Schä, L.; Trauner, D. Oxidative Approach Enables Efficient Access to Cyclic Azobenzenes. *J. Am. Chem. Soc.* **2019**, *141* (43), 17295–17304. https://doi.org/10.1021/jacs.9b08794.

(21) Wiley, R. E.; McLaughlin, M. F.; Johnson, J. S. Dearomatization of Cyclic Diphenylhydrazines: Harnessing the *o* -Semidine Rearrangement for the Synthesis of Spirocyclic Tetrahydroquinolines. *Org. Lett.* **2022**, *24* (43), 8014–8018. https://doi.org/10.1021/acs.orglett.2c03220.

(22) Molina, P.; Alajarin, M.; Sánchez-Andrada, P.; Elguero, J.; Luisa Jimeno, M.; Angew Chem Int Ed, P. A New and Efficient Preparation of Cyclic Carbodiimides from Bis(Iminophosphoranes) and the System Boc_2O/DMAP. *J. Org. Chem* **1994**, *59* (1), 7306–7315. https://doi.org/10.1021/jo00103a022.

(23) Sheu, J.; Smith, M. B.; Oeschger, T. R.; Satchell, J. N-Sulfonyl Lactams via Sulfonation of Lactim Ethers. *Org. Prep. Proced. Int.* **1992**, *24* (2), 147–157. https://doi.org/10.1080/00304949209355688.

(24) Ulrich, H.; Tucker, B.; Richter, R. Macrocyclic Ureas as Masked Isocyanates. *J. Org. Chem.* **1978**, *43* (8), 1544–1546. https://doi.org/10.1021/jo00402a013.

(25) Behringer, H.; Meier, H. Über Höhere *N,N′*-Alkylen-Harnstoffe Und -Thioharnstoffe. I. Lactamoxime und deren Lossen-Umlagerung zu *N,N′*-Alkylen-harnstoffen. *Justus Liebigs Ann. Chem.* **1957**, *607* (1), 67–73. https://doi.org/10.1002/jlac.19576070109.

(26) Richter, R.; Tucker, B.; Ulrich, H. Synthesis and Reactions of Cyclic Carbodiimides. *J. Org. Chem.* **1983**, *48* (10), 1694–1700. https://doi.org/10.1021/jo00158a022.

(27) Damrauer, R.; Soucy, D.; Winkler, P.; Eby, S. Synthesis and Nuclear Magnetic Resonance Study of 1,3-Diazacyclonona-1,2-Diene: An Unusual Carbodiimide. *J. Org. Chem.* **1980**, *45* (7), 1315–1317. https://doi.org/10.1021/jo01295a032.

(28) Narute, S.; Parnes, R.; Toste, F. D.; Pappo, D. Enantioselective Oxidative Homocoupling and Cross-Coupling of 2-Naphthols Catalyzed by Chiral Iron Phosphate Complexes. *J. Am. Chem. Soc.* **2016**, *138* (50), 16553–16560. https://doi.org/10.1021/jacs.6b11198.

(29) Zhou, C.; Chen, X.; Lu, P.; Wang, Y. Synthesis of 2,3-Diiodoindenes and Their Applications in Construction of 13H-Indeno[1,2-l]Phenanthrenes. *Tetrahedron* **2012**, *68* (13), 2844–2850. https://doi.org/10.1016/j.tet.2012.01.093.

(30) Revesz, L.; Blum, E.; Di Padova, F. E.; Buhl, T.; Feifel, R.; Gram, H.; Hiestand, P.; Manning, U.; Neumann, U.; Rucklin, G. Pyrazoloheteroaryls: Novel P38α MAP Kinase Inhibiting Scaffolds with Oral Activity. *Bioorg.Med. Chem. Lett.* **2006**, *16* (2), 262–266. https://doi.org/10.1016/j.bmcl.2005.10.015.

(31) Fell, J. B.; Coppola, G. M. A Mild and Efficient Preparation of Carbodiimides. *Synth. Commun.* **1995**, *25* (1), 43–47. https://doi.org/10.1080/00397919508010787.

(32) Molina, P.; Aller, E.; Lorenzo, A. One-Flask Conversion of N-Aryliminophosphoranesinto N^1,N^2,N^3 -Triarylguanidines Promoted by TBAF. *Synlett* 2003, *5*, 714–716. https://doi.org/10.1055/s-2003-38351.

(33) Li, Q.; Wang, S.; Zhou, S.; Yang, G.; Zhu, X.; Liu, Y. Highly Atom Efficient Guanylation of Both Aromatic and Secondary Amines Catalyzed by Simple Lanthanide Amides. *J. Org. Chem.* **2007**, *72* (18), 6763–6767. https://doi.org/10.1021/jo0709089.

(34) Holland, A. W.; Bergman, R. G. Heterocumulene Metathesis by Iridium Guanidinate and Ureylene Complexes: Catalysis Involving Reversible Insertion to Form Six-Membered Metallacycles. *J. Am. Chem. Soc.* **2002**, *124* (31), 9010–9011. https://doi.org/10.1021/ja026178y.

(35) Lyman, D. J.; Sadri, N. Polycarbodiimides and Their Derivatives. *Makromol. Chem.* **1963**, *67* (1), 1–9. https://doi.org/10.1002/macp.1963.020670101.

(36) Molina, P.; Alajarín, M.; Sánchez-Andrada, P.; Sanz-Aparicio, J.; Martínez-Ripoll, M. A Generalized and Efficient Preparation of a Novel Class of Macrocyclic Bis(Guanidines) from Cyclic Bis(Carbodiimides). *J. Org. Chem.* **1998**, *63* (9), 2922–2927. https://doi.org/10.1021/jo972107j.

(37) Wang, F.; Zhao, P.; Xi, C. Copper-Catalyzed One-Pot Synthesis of 2-Thioxo-2,3-Dihydroquinazolin-4(1H)-Ones from Ortho-Bromobenzamides and Isothiocyanates. *Tetrahedron Lett.* **2011**, *52* (2), 231–235. https://doi.org/10.1016/j.tetlet.2010.11.010.

(38) Guan, Z.-H.; Lei, H.; Chen, M.; Ren, Z.-H.; Bai, Y.; Wang, Y.-Y. Palladium-Catalyzed Carbonylation of Amines: Switchable Approaches to Carbamates and *N*,*N*′-Disubstituted Ureas. *Adv. Synth. Catal.* **2012**, *354* (2–3), 489–496. https://doi.org/10.1002/adsc.201100545.
