# Supplementary material for: Carbodiimide Ring-Opening Metathesis Polymerization
Source: ACS Cent Sci. 2023 May 11;9(6):1104–10. doi: 10.1021/acscentsci.3c00032 (PMC10311665; doi:10.1021/acscentsci.3c00032)
Supplement: Supplementary file 2 — oc3c00032_si_002.pdf [file oc3c00032_si_002.pdf]

Supplementary Information - Computational

Carbodiimide Ring-opening Metathesis Polymerization

J. Drake Johnson,[1] Samuel W. Kaplan,[1] Jozsef Toth,[1] Zian Wang,[1] Mitchell Maw,[1] Sergei S. Sheiko,[1] Aleksandr V. Zhukhovitskiy[1]*

Affiliations:

[1] Department of Chemistry, University of North Carolina at Chapel Hill; Chapel Hill, NC 27599, USA.

*Corresponding author. Email: alexzhuk@email.unc.edu

Ring Strain Calculations

M1

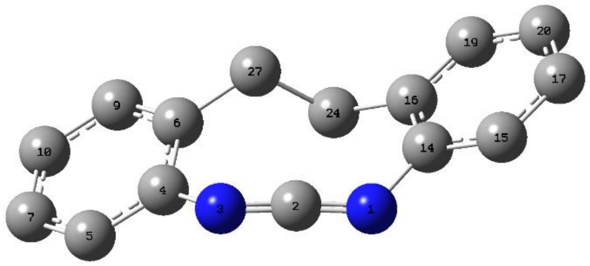

Zero-point correction= 0.225617 (Hartree/Particle)

Thermal correction to Energy= 0.238595

Thermal correction to Enthalpy= 0.239539

Thermal correction to Gibbs Free Energy= 0.186242

Sum of electronic and zero-point Energies= -687.406835

Sum of electronic and thermal Energies= -687.393857

Sum of electronic and thermal Enthalpies= -687.392913

Sum of electronic and thermal Free Energies= -687.446210

Center Number	Atomic Number	Atomic Type	Coordinates (Angstroms) X	Y	Z
1	7	0	-4.870194	3.888423	-0.258814
2	6	0	-5.611868	4.017814	-1.227336
3	7	0	-6.392861	4.014177	-2.173357
4	6	0	-6.345722	4.825289	-3.327555
5	6	0	-6.756341	4.284124	-4.554005
6	6	0	-5.964878	6.190711	-3.227418
7	6	0	-6.757055	5.077698	-5.704956

8	1	0	-7.066790	3.238024	-4.587096
9	6	0	-5.989897	6.963054	-4.396268
10	6	0	-6.371394	6.420456	-5.629789
11	1	0	-7.067807	4.645545	-6.658844
12	1	0	-5.707414	8.017776	-4.328471
13	1	0	-6.376104	7.046946	-6.524365
14	6	0	-4.375644	4.905584	0.585418
15	6	0	-4.205471	4.631562	1.949852
16	6	0	-3.981746	6.158071	0.041710
17	6	0	-3.679902	5.608510	2.800579
18	1	0	-4.495541	3.648892	2.326720
19	6	0	-3.445309	7.110763	0.918039
20	6	0	-3.299772	6.853150	2.286967
21	1	0	-3.560776	5.391046	3.864414
22	1	0	-3.130268	8.074894	0.508119
23	1	0	-2.882535	7.617910	2.945628
24	6	0	-4.115698	6.439950	-1.438341
25	1	0	-3.441915	7.266919	-1.710807
26	1	0	-3.763541	5.560237	-2.005538
27	6	0	-5.569923	6.792412	-1.897026
28	1	0	-6.282165	6.454863	-1.123510
29	1	0	-5.675236	7.887018	-1.950380

Diphenyl Carbodiimide

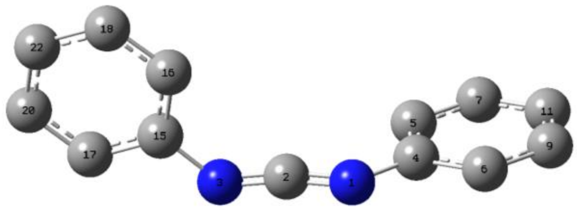

Zero-point correction= 0.189802 (Hartree/Particle)

Thermal correction to Energy= 0.202166

Thermal correction to Enthalpy= 0.203110

Thermal correction to Gibbs Free Energy= 0.147942

Sum of electronic and zero-point Energies= -610.096102

Sum of electronic and thermal Energies= -610.083739

Sum of electronic and thermal Enthalpies= -610.082795

Sum of electronic and thermal Free Energies= -610.137963

Center Number	Atomic Number	Atomic Type	Coordinates (Angstroms) X	Y	Z
1	7	0	1.159587	-0.173576	0.517475
2	6	0	0.001894	-0.109350	0.109848
3	7	0	-1.155471	-0.250588	-0.278744
4	6	0	2.333149	0.448566	0.056891
5	6	0	2.353305	1.258773	-1.095362
6	6	0	3.518113	0.235954	0.781193
7	6	0	3.549656	1.846809	-1.510409
8	1	0	1.427597	1.416696	-1.655345
9	6	0	4.710138	0.830299	0.357393
10	1	0	3.483505	-0.397237	1.669845
11	6	0	4.732281	1.636705	-0.787399
12	1	0	3.558726	2.473812	-2.405268
13	1	0	5.627856	0.660760	0.925375
14	1	0	5.665358	2.099263	-1.116193
15	6	0	-2.330655	0.444081	0.057086
16	6	0	-2.354625	1.450926	1.042064
17	6	0	-3.513319	0.101615	-0.619648
18	6	0	-3.552463	2.103810	1.339181

19	1	0	-1.430730	1.709132	1.566653
20	6	0	-4.706810	0.762389	-0.315150
21	1	0	-3.475767	-0.683173	-1.377623
22	6	0	-4.732741	1.764271	0.663050
23	1	0	-3.564525	2.883593	2.104544
24	1	0	-5.622699	0.491229	-0.845407
25	1	0	-5.666969	2.278309	0.899016

Open Bisdiphenyl Carbodiimide

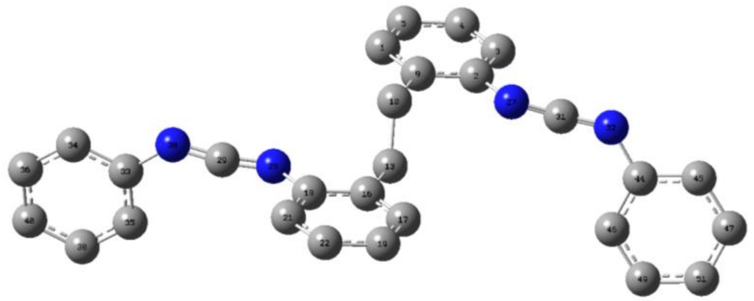

Zero-point correction= 0.415769 (Hartree/Particle)

Thermal correction to Energy= 0.443665

Thermal correction to Enthalpy= 0.444609

Thermal correction to Gibbs Free Energy= 0.347687

Sum of electronic and zero-point Energies= -1297.519740

Sum of electronic and thermal Energies= -1297.491844

Sum of electronic and thermal Enthalpies= -1297.490900

Sum of electronic and thermal Free Energies= -1297.587822

Center Number	Atomic Number	Atomic Type	Coordinates (Angstroms) X	Y	Z
1	6	0	0.983427	2.667528	0.808940
2	6	0	2.646705	0.926750	0.628689

3	6	0	3.654747	1.904831	0.544142
4	6	0	3.321311	3.259252	0.590085
5	6	0	1.980504	3.644683	0.721391
6	1	0	4.696393	1.586565	0.447542
7	1	0	4.109118	4.013340	0.524964
8	1	0	1.714071	4.703322	0.759118
9	6	0	1.291142	1.299383	0.770761
10	6	0	0.207613	0.255319	0.823708
11	1	0	-0.709792	0.690524	1.250306
12	1	0	0.522799	-0.583400	1.465850
13	6	0	-0.115144	-0.301422	-0.586231
14	1	0	0.804759	-0.728603	-1.016224
15	1	0	-0.437693	0.535724	-1.226567
16	6	0	-1.186717	-1.357844	-0.534093
17	6	0	-0.859316	-2.721821	-0.562943
18	6	0	-2.548255	-1.003091	-0.404271
19	6	0	-1.842097	-3.713011	-0.474596
20	1	0	0.194075	-3.000690	-0.658475
21	6	0	-3.541750	-1.996285	-0.318347
22	6	0	-3.188982	-3.345961	-0.352769
23	1	0	-1.559906	-4.767841	-0.502513
24	1	0	-4.588272	-1.693577	-0.223048
25	1	0	-3.966134	-4.110772	-0.284884
26	1	0	-0.065001	2.960572	0.914563
27	7	0	2.949051	-0.447474	0.596112
28	7	0	-2.867061	0.367651	-0.361490
29	6	0	-3.944460	0.950144	-0.269335
30	7	0	-4.925908	1.651305	-0.030310
31	6	0	3.974519	-1.057958	0.310602
32	7	0	4.964266	-1.777829	0.184666

33	6	0	-5.848430	2.264426	-0.896194
34	6	0	-6.921776	2.966913	-0.323102
35	6	0	-5.723516	2.200206	-2.297952
36	6	0	-7.860683	3.596949	-1.144862
37	1	0	-7.001201	3.007335	0.764978
38	6	0	-6.666537	2.833814	-3.109516
39	1	0	-4.884120	1.653607	-2.736368
40	6	0	-7.738550	3.534017	-2.538582
41	1	0	-8.692829	4.141304	-0.692330
42	1	0	-6.563714	2.780349	-4.196062
43	1	0	-8.473705	4.027516	-3.177870
44	6	0	5.433285	-2.487767	-0.934964
45	6	0	6.657166	-3.167891	-0.818610
46	6	0	4.714906	-2.544374	-2.145682
47	6	0	7.157185	-3.893996	-1.903283
48	1	0	7.198534	-3.115429	0.127973
49	6	0	5.222636	-3.273676	-3.222576
50	1	0	3.762349	-2.013605	-2.228641
51	6	0	6.444776	-3.950902	-3.107724
52	1	0	8.109836	-4.419847	-1.805620
53	1	0	4.660292	-3.312963	-4.158667
54	1	0	6.838060	-4.519636	-3.953030

M2

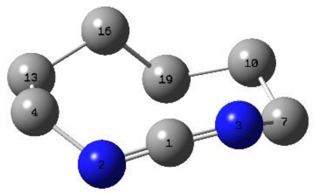

Zero-point correction= 0.180335 (Hartree/Particle)

Thermal correction to Energy= 0.188927

Thermal correction to Enthalpy= 0.189872

Thermal correction to Gibbs Free Energy= 0.147501

Sum of electronic and zero-point Energies= -382.881262

Sum of electronic and thermal Energies= -382.872670

Sum of electronic and thermal Enthalpies= -382.871725

Sum of electronic and thermal Free Energies= -382.914096

Center Number	Atomic Number	Atomic Type	Coordinates (Angstroms) X	Y	Z
1	6	0	0.747920	0.569485	0.805499
2	7	0	1.002091	1.517941	1.546425
3	7	0	0.612155	-0.399084	0.059569
4	6	0	0.314129	2.813635	1.457970
5	1	0	0.522944	3.266116	0.470507
6	1	0	0.750338	3.472879	2.223172
7	6	0	-0.455230	-1.396110	0.222629
8	1	0	-0.326798	-2.150707	-0.567612
9	1	0	-0.325880	-1.907026	1.195063
10	6	0	-1.872011	-0.799456	0.154076
11	1	0	-2.574880	-1.635582	0.310246
12	1	0	-2.060694	-0.414283	-0.863105
13	6	0	-1.209224	2.717837	1.653592
14	1	0	-1.428747	2.402850	2.688606
15	1	0	-1.606857	3.741269	1.544195
16	6	0	-1.903333	1.761046	0.657484
17	1	0	-2.879030	2.181704	0.364997
18	1	0	-1.308640	1.722611	-0.272682
19	6	0	-2.125189	0.318141	1.191627

20	1	0	-1.474714	0.145028	2.067555
21	1	0	-3.156599	0.238480	1.571503

Dimethyl Carbodiimide

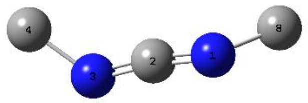

Zero-point correction= 0.087115 (Hartree/Particle)

Thermal correction to Energy= 0.093822

Thermal correction to Enthalpy= 0.094766

Thermal correction to Gibbs Free Energy= 0.056573

Sum of electronic and zero-point Energies= -227.092003

Sum of electronic and thermal Energies= -227.085295

Sum of electronic and thermal Enthalpies= -227.084351

Sum of electronic and thermal Free Energies= -227.122545

Center Number	Atomic Number	Atomic Type	Coordinates (Angstroms) X	Y	Z
1	7	0	1.163155	-0.396550	0.383821
2	6	0	-0.000113	-0.301098	-0.000428
3	7	0	-1.163428	-0.395510	-0.384799
4	6	0	-2.310694	0.350262	0.115429
5	1	0	-2.774173	0.904617	-0.716198
6	1	0	-3.060984	-0.360738	0.497012
7	1	0	-2.050721	1.060270	0.919480
8	6	0	2.310733	0.349492	-0.115287
9	1	0	3.060658	-0.361241	-0.498087
10	1	0	2.051030	1.060925	-0.918166
11	1	0	2.774540	0.902274	0.717203

Open Bisdimethyl Carbodiimide

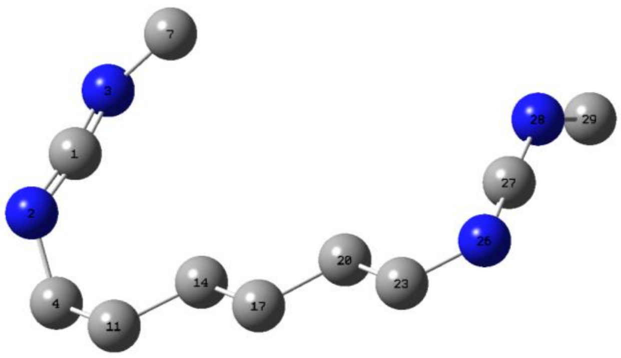

Zero-point correction= 0.267157 (Hartree/Particle)

Thermal correction to Energy= 0.285244

Thermal correction to Enthalpy= 0.286188

Thermal correction to Gibbs Free Energy= 0.215656

Sum of electronic and zero-point Energies= -609.988094

Sum of electronic and thermal Energies= -609.970007

Sum of electronic and thermal Enthalpies= -609.969062

Sum of electronic and thermal Free Energies= -610.039594

Center Number	Atomic Number	Atomic Type	Coordinates (Angstroms) X	Y	Z
1	6	0	2.948807	1.498758	-2.878326
2	7	0	1.965715	1.687178	-2.162311
3	7	0	3.896889	1.486542	-3.659320
4	6	0	0.879530	0.729513	-1.943472
5	1	0	-0.005231	1.303909	-1.629922
6	1	0	0.628258	0.205357	-2.885039
7	6	0	5.229406	0.958833	-3.399418
8	1	0	5.539944	0.330174	-4.248041

9	1	0	5.944767	1.794344	-3.324754
10	1	0	5.284161	0.361064	-2.472330
11	6	0	1.232558	-0.301423	-0.861607
12	1	0	1.489042	0.231705	0.070654
13	1	0	0.326283	-0.898493	-0.654511
14	6	0	2.379713	-1.233270	-1.257383
15	1	0	3.284051	-0.635586	-1.473558
16	1	0	2.120535	-1.757857	-2.196228
17	6	0	2.720320	-2.261596	-0.174348
18	1	0	1.829755	-2.881608	0.038378
19	1	0	2.963246	-1.732338	0.765591
20	6	0	3.892011	-3.164784	-0.568417
21	1	0	4.785357	-2.547904	-0.775310
22	1	0	3.652869	-3.700743	-1.505093
23	6	0	4.232903	-4.186390	0.518587
24	1	0	4.466776	-3.664250	1.464178
25	1	0	3.357339	-4.825142	0.728164
26	7	0	5.357716	-5.067113	0.214879
27	6	0	6.129196	-4.981634	-0.738555
28	7	0	7.037920	-4.930284	-1.565323
29	6	0	7.067787	-5.597409	-2.860308
30	1	0	7.183469	-4.843575	-3.655604
31	1	0	7.947458	-6.259524	-2.903055
32	1	0	6.161847	-6.194981	-3.062196

Complete Catalytic Cycle for M1 (Initiation and Propagation)

M1

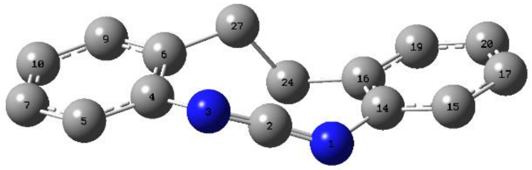

Zero-point correction= 0.225442 (Hartree/Particle)

Thermal correction to Energy= 0.238426

Thermal correction to Enthalpy= 0.239370

Thermal correction to Gibbs Free Energy= 0.186059

Sum of electronic and zero-point Energies= -687.412396

Sum of electronic and thermal Energies= -687.399412

Sum of electronic and thermal Enthalpies= -687.398468

Sum of electronic and thermal Free Energies= -687.451778

Center Number	Atomic Number	Atomic Type	Coordinates (Angstroms) X	Y	Z
1	7	0	-4.855624	3.882917	-0.270084
2	6	0	-5.611509	4.019232	-1.227579
3	7	0	-6.406898	4.020958	-2.162416
4	6	0	-6.352637	4.828932	-3.323060
5	6	0	-6.763494	4.283482	-4.547812
6	6	0	-5.965878	6.192728	-3.224534
7	6	0	-6.758510	5.073787	-5.702359
8	1	0	-7.077420	3.238208	-4.581376
9	6	0	-5.984853	6.961788	-4.396824

10	6	0	-6.366626	6.415801	-5.629801
11	1	0	-7.068742	4.639536	-6.655361
12	1	0	-5.696679	8.014857	-4.332009
13	1	0	-6.366335	7.039394	-6.526399
14	6	0	-4.368508	4.903669	0.580537
15	6	0	-4.200348	4.625573	1.944670
16	6	0	-3.980419	6.158599	0.038477
17	6	0	-3.680571	5.603785	2.799321
18	1	0	-4.487173	3.641893	2.322040
19	6	0	-3.449957	7.112636	0.918523
20	6	0	-3.305488	6.851821	2.287895
21	1	0	-3.562890	5.384513	3.862845
22	1	0	-3.140142	8.079303	0.511411
23	1	0	-2.893213	7.617310	2.948822
24	6	0	-4.113969	6.444973	-1.440678
25	1	0	-3.444172	7.275573	-1.709178
26	1	0	-3.759684	5.567555	-2.010092
27	6	0	-5.569489	6.797835	-1.896169
28	1	0	-6.281799	6.463248	-1.121396
29	1	0	-5.670740	7.891915	-1.953845

1

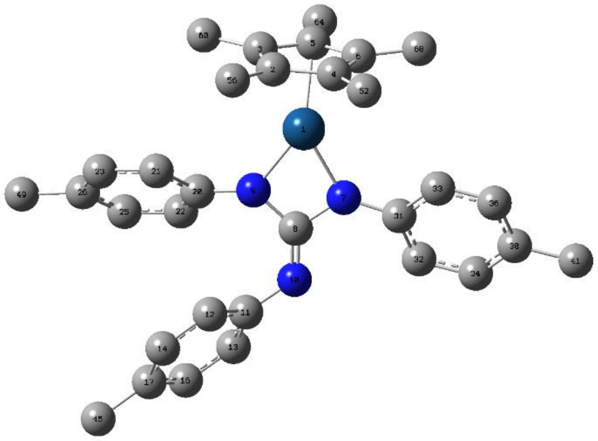

Zero-point correction= 0.578953 (Hartree/Particle)

Thermal correction to Energy= 0.617682

Thermal correction to Enthalpy= 0.618627

Thermal correction to Gibbs Free Energy= 0.504112

Sum of electronic and zero-point Energies= -1508.196942

Sum of electronic and thermal Energies= -1508.158212

Sum of electronic and thermal Enthalpies= -1508.157268

Sum of electronic and thermal Free Energies= -1508.271783

Center Number	Atomic Number	Atomic Type	Coordinates (Angstroms) X	Y	Z
1	77	0	-0.124976	-0.762464	-1.574821
2	6	0	0.167876	-0.673154	-3.725746
3	6	0	0.038651	0.663456	-3.217655
4	6	0	-1.076564	-1.394364	-3.432744
5	6	0	-1.263562	0.757068	-2.571136
6	6	0	-1.971150	-0.496827	-2.757600
7	7	0	-0.404453	-1.624805	0.226901
8	6	0	0.885405	-1.387795	0.719461

9	7	0	1.461886	-0.694269	-0.356036
10	7	0	1.351571	-1.793213	1.855770
11	6	0	2.720173	-1.808983	2.143664
12	6	0	3.685201	-2.294878	1.231358
13	6	0	3.160331	-1.397234	3.417845
14	6	0	5.036673	-2.326540	1.575156
15	1	0	3.356549	-2.627244	0.243911
16	6	0	4.519538	-1.419124	3.744470
17	6	0	5.485106	-1.880179	2.832910
18	1	0	5.766097	-2.696859	0.847902
19	1	0	4.841350	-1.071659	4.731099
20	6	0	2.613221	0.098798	-0.330707
21	6	0	3.428280	0.151213	-1.475443
22	6	0	2.971624	0.876686	0.789675
23	6	0	4.583960	0.936560	-1.491107
24	1	0	3.143128	-0.451862	-2.340542
25	6	0	4.134440	1.648345	0.765606
26	6	0	4.967213	1.691621	-0.368678
27	1	0	5.208095	0.959127	-2.389674
28	1	0	4.401637	2.238879	1.647354
29	1	0	2.420664	-1.033374	4.135752
30	1	0	2.338196	0.857988	1.678277
31	6	0	-1.417355	-2.380505	0.815635
32	6	0	-1.545133	-2.579229	2.210207
33	6	0	-2.393203	-2.953303	-0.027344
34	6	0	-2.617080	-3.320583	2.716496
35	1	0	-0.794625	-2.157185	2.877224
36	6	0	-3.461429	-3.682416	0.495088
37	1	0	-2.274961	-2.819541	-1.103646
38	6	0	-3.599731	-3.881236	1.881033

39	1	0	-2.694714	-3.465660	3.798799
40	1	0	-4.198820	-4.118215	-0.186624
41	6	0	-4.770670	-4.645173	2.449447
42	1	0	-5.628762	-3.976914	2.644116
43	1	0	-5.116169	-5.424033	1.751163
44	1	0	-4.507904	-5.126298	3.404675
45	6	0	6.949092	-1.926983	3.196846
46	1	0	7.580272	-1.595075	2.356443
47	1	0	7.163170	-1.286060	4.065966
48	1	0	7.265412	-2.953485	3.453851
49	6	0	6.239933	2.502563	-0.366640
50	1	0	7.056067	1.959127	0.141925
51	1	0	6.575978	2.720497	-1.392136
52	6	0	-1.366115	-2.794691	-3.864854
53	1	0	-2.167678	-3.245233	-3.262354
54	1	0	-1.685729	-2.804569	-4.921806
55	1	0	-0.468423	-3.424432	-3.776855
56	6	0	1.316927	-1.231271	-4.500978
57	1	0	1.514376	-2.275305	-4.215250
58	1	0	1.090520	-1.211354	-5.581415
59	1	0	2.230120	-0.642650	-4.334808
60	6	0	1.028561	1.776685	-3.337835
61	1	0	2.053755	1.391636	-3.423303
62	1	0	0.807925	2.387999	-4.229877
63	1	0	0.987933	2.432234	-2.455217
64	6	0	-1.831475	1.978417	-1.927386
65	1	0	-1.038065	2.593584	-1.479257
66	1	0	-2.353670	2.589950	-2.684723
67	1	0	-2.556322	1.707489	-1.146490
68	6	0	-3.393929	-0.741043	-2.364987

69	1	0	-3.578185	-0.441125	-1.322400
70	1	0	-4.064961	-0.154234	-3.015387
71	1	0	-3.663771	-1.800765	-2.466724
72	1	0	6.105457	3.457463	0.166432

Structure 2*

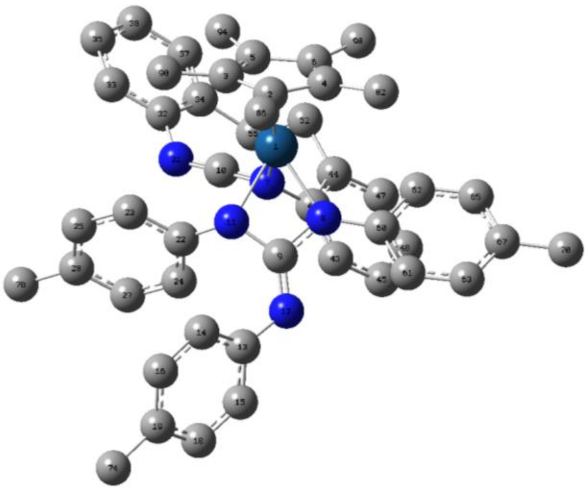

Zero-point correction= 0.806307 (Hartree/Particle)

Thermal correction to Energy= 0.859346

Thermal correction to Enthalpy= 0.860290

Thermal correction to Gibbs Free Energy= 0.715328

Sum of electronic and zero-point Energies= -2195.622131

Sum of electronic and thermal Energies= -2195.569092

Sum of electronic and thermal Enthalpies= -2195.568148

Sum of electronic and thermal Free Energies= -2195.713109

Center Atomic Atomic Coordinates (Angstroms)

Number	Number	Type	X	Y	Z
1	77	0	-0.266966	-0.656938	-1.174046
2	6	0	-0.155077	-0.981196	-3.270871
3	6	0	-1.243258	-0.034312	-3.035588
4	6	0	-0.530556	-2.235732	-2.632446
5	6	0	-2.218446	-0.675977	-2.204138
6	6	0	-1.784679	-2.048256	-1.944727
7	7	0	-0.920689	-0.074804	0.870531
8	7	0	1.431662	-1.287455	-0.196057
9	6	0	2.064194	-0.072591	-0.063531
10	6	0	-1.676652	0.921824	0.905660
11	7	0	1.240202	0.804947	-0.777475
12	7	0	3.149054	0.134579	0.646025
13	6	0	3.869504	1.322135	0.584247
14	6	0	4.049478	2.086919	-0.597999
15	6	0	4.528402	1.776198	1.750816
16	6	0	4.833444	3.241230	-0.595772
17	1	0	3.547426	1.765634	-1.513355
18	6	0	5.303607	2.939886	1.741566
19	6	0	5.475176	3.699062	0.571319
20	1	0	4.947342	3.810720	-1.524488
21	1	0	5.788312	3.269933	2.666409
22	6	0	0.958307	2.101050	-0.317116
23	6	0	0.483022	3.056322	-1.237530
24	6	0	1.079860	2.492764	1.035350
25	6	0	0.139429	4.345836	-0.825656
26	1	0	0.392768	2.766646	-2.284678
27	6	0	0.747660	3.789002	1.434117
28	6	0	0.269619	4.742628	0.517624

29	1	0	-0.229359	5.066110	-1.562942
30	1	0	0.851399	4.067112	2.487812
31	7	0	-2.379456	1.912876	0.918214
32	6	0	-3.796593	1.975965	0.903909
33	6	0	-4.404130	2.933704	0.081729
34	6	0	-4.560415	1.117613	1.733958
35	6	0	-5.799217	3.012453	0.025415
36	1	0	-3.772848	3.593265	-0.517364
37	6	0	-5.956847	1.232874	1.664868
38	6	0	-6.577799	2.156229	0.813707
39	1	0	-6.273774	3.747624	-0.627712
40	1	0	-6.563413	0.584355	2.302907
41	1	0	-7.667605	2.216971	0.779391
42	6	0	-0.802010	-0.894287	2.061114
43	6	0	0.454217	-1.093231	2.640677
44	6	0	-1.966260	-1.506833	2.579092
45	6	0	0.572655	-1.956875	3.734965
46	1	0	1.332189	-0.591415	2.232638
47	6	0	-1.812436	-2.377563	3.668738
48	6	0	-0.555643	-2.613261	4.239469
49	1	0	1.555468	-2.123106	4.181281
50	1	0	-2.701191	-2.868229	4.075054
51	1	0	-0.461223	-3.300582	5.083183
52	6	0	-3.335989	-1.161494	2.046796
53	1	0	-3.286505	-1.043144	0.954246
54	1	0	-4.033117	-1.988841	2.244714
55	6	0	-3.895905	0.156172	2.691680
56	1	0	-4.612790	-0.111930	3.481604
57	1	0	-3.064162	0.680295	3.192631
58	1	0	4.404738	1.199464	2.671921

59	1	0	1.440946	1.768500	1.767690
60	6	0	1.851893	-2.541395	0.227361
61	6	0	3.206369	-2.886392	0.440282
62	6	0	0.872881	-3.535666	0.453887
63	6	0	3.545242	-4.178232	0.859199
64	1	0	3.974737	-2.130318	0.281478
65	6	0	1.228789	-4.818224	0.865911
66	1	0	-0.175091	-3.261866	0.308392
67	6	0	2.575309	-5.170577	1.081337
68	1	0	4.601025	-4.424884	1.012639
69	1	0	0.444732	-5.563987	1.033218
70	6	0	2.955329	-6.550728	1.560704
71	1	0	2.784699	-6.661311	2.646765
72	1	0	2.357599	-7.329462	1.059295
73	1	0	4.020278	-6.757637	1.371518
74	6	0	6.333816	4.940992	0.555538
75	1	0	5.873226	5.740253	-0.048255
76	1	0	6.492156	5.327041	1.574819
77	1	0	7.329155	4.740019	0.119727
78	6	0	-0.058753	6.149438	0.954265
79	1	0	-0.839870	6.593781	0.317433
80	1	0	-0.406247	6.171799	1.999280
81	1	0	0.828520	6.804626	0.890711
82	6	0	0.261079	-3.498957	-2.729478
83	1	0	-0.031611	-4.221008	-1.957285
84	1	0	0.096494	-3.953032	-3.721962
85	1	0	1.336304	-3.296990	-2.616418
86	6	0	1.024181	-0.765129	-4.163629
87	1	0	1.855833	-1.423932	-3.875244
88	1	0	0.758167	-0.983699	-5.212543

89	1	0	1.371811	0.276484	-4.105367
90	6	0	-1.371909	1.330197	-3.629607
91	1	0	-0.401274	1.696879	-3.991792
92	1	0	-2.063218	1.296343	-4.489747
93	1	0	-1.768860	2.051084	-2.899586
94	6	0	-3.526316	-0.072929	-1.807791
95	1	0	-3.438424	1.011609	-1.655758
96	1	0	-4.267050	-0.240970	-2.609503
97	1	0	-3.931054	-0.521643	-0.890270
98	6	0	-2.582297	-3.104423	-1.248298
99	1	0	-3.159884	-2.684612	-0.412246
100	1	0	-3.295779	-3.572504	-1.949220
101	1	0	-1.931238	-3.896393	-0.850759

TS1*

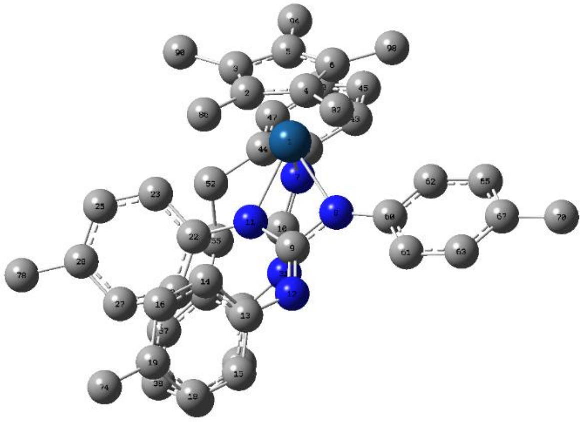

Zero-point correction= 0.805839 (Hartree/Particle)

Thermal correction to Energy= 0.857936

Thermal correction to Enthalpy= 0.858880

Thermal correction to Gibbs Free Energy= 0.716483

Sum of electronic and zero-point Energies= -2195.612632

Sum of electronic and thermal Energies= -2195.560535

Sum of electronic and thermal Enthalpies= -2195.559590

Sum of electronic and thermal Free Energies= -2195.701987

Center Number	Atomic Number	Atomic Type	Coordinates (Angstroms) X	Y	Z
1	77	0	-1.379582	-0.286557	-0.790618
2	6	0	-1.296376	-1.331513	-2.691122
3	6	0	-1.582266	0.079263	-2.949038
4	6	0	-2.366004	-1.869405	-1.891474
5	6	0	-2.828925	0.392144	-2.297561
6	6	0	-3.325373	-0.793052	-1.638370
7	7	0	-1.087275	1.365748	0.431604
8	7	0	-1.017934	-1.374267	0.993625
9	6	0	0.344283	-1.438316	0.893280
10	6	0	0.020677	1.260951	1.099555
11	7	0	0.609153	-0.327138	0.015836
12	7	0	1.136369	-2.268582	1.505691
13	6	0	2.354570	-2.707362	1.010424
14	6	0	2.560594	-2.980301	-0.365183
15	6	0	3.421026	-2.979822	1.897504
16	6	0	3.784189	-3.466841	-0.825407
17	1	0	1.744144	-2.785421	-1.065493
18	6	0	4.644668	-3.458222	1.420754
19	6	0	4.856361	-3.710793	0.053131
20	1	0	3.916504	-3.656467	-1.895647
21	1	0	5.460389	-3.640738	2.127764
22	6	0	1.843231	0.081298	-0.550039
23	6	0	1.816636	0.710880	-1.802309
24	6	0	3.060321	0.050039	0.157276

25	6	0	2.975424	1.270714	-2.351734
26	1	0	0.860771	0.779714	-2.315206
27	6	0	4.208855	0.611230	-0.396078
28	6	0	4.195922	1.229277	-1.661417
29	1	0	2.924636	1.758877	-3.329713
30	1	0	5.136177	0.590018	0.183355
31	7	0	0.667131	1.674497	2.073995
32	6	0	1.969921	2.183128	2.191257
33	6	0	2.930612	1.482555	2.938174
34	6	0	2.274043	3.447420	1.625526
35	6	0	4.236474	1.969949	3.034502
36	1	0	2.646459	0.534115	3.399624
37	6	0	3.596399	3.905342	1.727458
38	6	0	4.580375	3.173423	2.405695
39	1	0	4.985438	1.403992	3.593266
40	1	0	3.846075	4.873142	1.282319
41	1	0	5.601551	3.556346	2.466464
42	6	0	-1.762845	2.621396	0.397061
43	6	0	-3.137220	2.636918	0.671395
44	6	0	-1.078233	3.796276	0.008382
45	6	0	-3.861308	3.826718	0.569102
46	1	0	-3.620479	1.698393	0.952838
47	6	0	-1.835362	4.976252	-0.102428
48	6	0	-3.207579	5.001027	0.171861
49	1	0	-4.931656	3.834504	0.786778
50	1	0	-1.326927	5.893435	-0.413618
51	1	0	-3.765764	5.934841	0.073321
52	6	0	0.417327	3.838483	-0.218089
53	1	0	0.796622	2.856282	-0.541401
54	1	0	0.626171	4.537280	-1.043024

55	6	0	1.184952	4.331997	1.059035
56	1	0	1.622180	5.320441	0.850824
57	1	0	0.441121	4.485492	1.860661
58	1	0	3.277815	-2.781958	2.963272
59	1	0	3.102580	-0.391645	1.148826
60	6	0	-1.867071	-2.177817	1.744966
61	6	0	-1.510821	-3.410887	2.341294
62	6	0	-3.198615	-1.727468	1.900664
63	6	0	-2.466350	-4.147085	3.050460
64	1	0	-0.488240	-3.771047	2.237237
65	6	0	-4.138133	-2.477795	2.605662
66	1	0	-3.467246	-0.774189	1.439932
67	6	0	-3.792907	-3.707417	3.199227
68	1	0	-2.172046	-5.102606	3.496735
69	1	0	-5.162321	-2.103235	2.701076
70	6	0	-4.802072	-4.504338	3.989137
71	1	0	-4.512068	-5.564898	4.049876
72	1	0	-4.889846	-4.126368	5.023553
73	1	0	-5.804519	-4.443349	3.535745
74	6	0	6.169795	-4.259995	-0.449291
75	1	0	6.402122	-3.880583	-1.457353
76	1	0	6.998460	-3.987287	0.223115
77	1	0	6.148331	-5.362898	-0.513271
78	6	0	5.451007	1.836280	-2.236715
79	1	0	5.900090	2.556763	-1.532325
80	1	0	6.213140	1.063656	-2.436481
81	1	0	5.242883	2.361022	-3.181462
82	6	0	-2.508176	-3.279448	-1.416005
83	1	0	-2.982667	-3.307079	-0.423837
84	1	0	-3.132038	-3.857923	-2.118715

85	1	0	-1.526686	-3.768982	-1.338331
86	6	0	-0.115445	-2.081782	-3.220287
87	1	0	0.070927	-2.989165	-2.628252
88	1	0	-0.298023	-2.386277	-4.265198
89	1	0	0.790297	-1.457088	-3.196565
90	6	0	-0.894442	0.973501	-3.933947
91	1	0	0.089210	0.578327	-4.225179
92	1	0	-1.506616	1.046765	-4.850010
93	1	0	-0.763573	1.991484	-3.536483
94	6	0	-3.520533	1.711993	-2.392383
95	1	0	-2.808052	2.543253	-2.281611
96	1	0	-4.000994	1.798216	-3.382749
97	1	0	-4.293762	1.818847	-1.621278
98	6	0	-4.665057	-0.945306	-0.994638
99	1	0	-4.978274	-0.013731	-0.499686
100	1	0	-5.421572	-1.194039	-1.759667
101	1	0	-4.656972	-1.749796	-0.246214

Structure 2

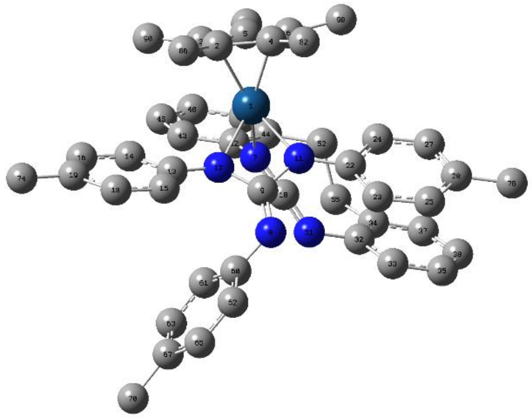

Zero-point correction= 0.806429 (Hartree/Particle)

Thermal correction to Energy= 0.859112

Thermal correction to Enthalpy= 0.860056

Thermal correction to Gibbs Free Energy= 0.717182

Sum of electronic and zero-point Energies= -2195.631131

Sum of electronic and thermal Energies= -2195.578449

Sum of electronic and thermal Enthalpies= -2195.577504

Sum of electronic and thermal Free Energies= -2195.720378

Center Number	Atomic Number	Atomic Type	Coordinates (Angstroms) X	Y	Z
1	77	0	-1.647985	3.281403	-0.192907
2	6	0	-1.353570	4.888314	1.189602
3	6	0	-0.928103	5.355001	-0.125307
4	6	0	-0.526871	3.752520	1.553096
5	6	0	0.173299	4.513444	-0.542570
6	6	0	0.434533	3.531764	0.477426
7	7	0	-1.926211	2.484324	-2.219944
8	7	0	-4.742338	0.898244	0.543709
9	6	0	-3.747342	1.723275	0.310018
10	6	0	-2.293726	1.296182	-2.403482
11	7	0	-2.402626	1.406849	0.376534
12	7	0	-3.723004	3.054001	-0.087431
13	6	0	-4.694512	4.013573	0.159623
14	6	0	-4.670399	5.205591	-0.595819
15	6	0	-5.678957	3.892390	1.167831
16	6	0	-5.592188	6.228011	-0.365021
17	1	0	-3.891612	5.315574	-1.352507
18	6	0	-6.602386	4.918778	1.380249
19	6	0	-6.586112	6.104723	0.623244
20	1	0	-5.541513	7.142672	-0.964372
21	1	0	-7.354199	4.801040	2.167385
22	6	0	-1.805247	0.165714	0.526154
23	6	0	-2.416932	-0.936713	1.170817

24	6	0	-0.496531	-0.012312	0.018113
25	6	0	-1.735933	-2.154685	1.273874
26	1	0	-3.425893	-0.819361	1.564572
27	6	0	0.165021	-1.232909	0.132512
28	6	0	-0.442422	-2.337692	0.757225
29	1	0	-2.231619	-2.996562	1.768791
30	1	0	1.169746	-1.338135	-0.289452
31	7	0	-2.709466	0.171866	-2.581106
32	6	0	-1.974797	-1.026806	-2.786195
33	6	0	-2.348681	-2.165678	-2.067235
34	6	0	-0.923660	-1.053971	-3.735265
35	6	0	-1.633910	-3.353482	-2.242180
36	1	0	-3.160271	-2.093509	-1.342752
37	6	0	-0.237268	-2.265890	-3.898647
38	6	0	-0.575014	-3.405533	-3.156064
39	1	0	-1.904752	-4.235435	-1.658373
40	1	0	0.573333	-2.309851	-4.631398
41	1	0	-0.017278	-4.333689	-3.299319
42	6	0	-1.410851	3.202052	-3.363277
43	6	0	-1.946692	4.461242	-3.644479
44	6	0	-0.336234	2.664971	-4.104157
45	6	0	-1.416150	5.220784	-4.691075
46	1	0	-2.766698	4.830874	-3.028044
47	6	0	0.184317	3.454254	-5.143351
48	6	0	-0.342032	4.717048	-5.437933
49	1	0	-1.833444	6.205013	-4.913747
50	1	0	1.026023	3.064938	-5.722379
51	1	0	0.087559	5.309522	-6.248556
52	6	0	0.211251	1.285591	-3.820424
53	1	0	1.265756	1.242710	-4.130812

54	1	0	0.195542	1.091829	-2.734898
55	6	0	-0.591105	0.161956	-4.568023
56	1	0	-1.531954	0.596618	-4.946340
57	1	0	-0.019103	-0.156533	-5.451864
58	1	0	-5.709508	2.989969	1.779652
59	1	0	-0.027210	0.834821	-0.486815
60	6	0	-6.007795	1.102957	0.005407
61	6	0	-6.237356	1.666872	-1.276900
62	6	0	-7.142631	0.664641	0.727501
63	6	0	-7.529471	1.791001	-1.789087
64	1	0	-5.380419	2.019005	-1.856421
65	6	0	-8.432711	0.802829	0.206501
66	1	0	-6.989320	0.224775	1.717139
67	6	0	-8.657959	1.367282	-1.061098
68	1	0	-7.670721	2.234269	-2.780742
69	1	0	-9.289848	0.465303	0.798688
70	6	0	-10.049723	1.487988	-1.634196
71	1	0	-10.171900	2.427987	-2.196902
72	1	0	-10.273736	0.661388	-2.332625
73	1	0	-10.810852	1.460998	-0.838442
74	6	0	-7.611620	7.189637	0.844831
75	1	0	-8.514165	7.020451	0.230263
76	1	0	-7.936344	7.223461	1.897028
77	1	0	-7.211673	8.179248	0.572634
78	6	0	0.254461	-3.673377	0.822838
79	1	0	1.305353	-3.568319	1.139842
80	1	0	-0.251792	-4.353052	1.525954
81	1	0	0.261171	-4.160306	-0.169353
82	6	0	-0.586057	2.973962	2.827997
83	1	0	-0.386726	1.909089	2.635375

84	1	0	0.169995	3.348583	3.539047
85	1	0	-1.576874	3.061810	3.295648
86	6	0	-2.407436	5.518084	2.040112
87	1	0	-2.857460	4.781025	2.720405
88	1	0	-1.960395	6.325099	2.646073
89	1	0	-3.209062	5.945468	1.421012
90	6	0	-1.404460	6.591838	-0.816088
91	1	0	-2.452580	6.805463	-0.560472
92	1	0	-0.795423	7.460063	-0.508131
93	1	0	-1.322130	6.491439	-1.908507
94	6	0	0.978376	4.685082	-1.787442
95	1	0	0.392149	5.151432	-2.590532
96	1	0	1.843297	5.335800	-1.568341
97	1	0	1.361930	3.721137	-2.150314
98	6	0	1.589688	2.584828	0.519137
99	1	0	1.894030	2.278326	-0.492902
100	1	0	2.455682	3.073073	1.000054
101	1	0	1.343096	1.680548	1.093856

TS1

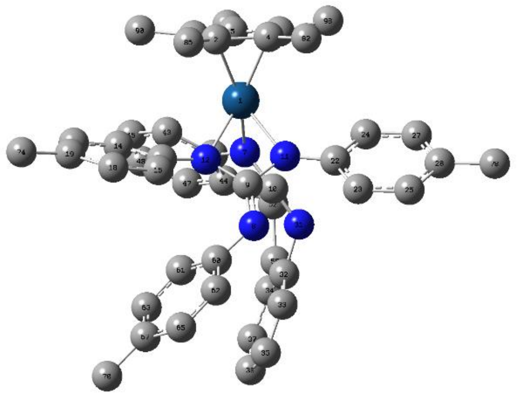

Zero-point correction= 0.805608 (Hartree/Particle)

Thermal correction to Energy= 0.857956

Thermal correction to Enthalpy= 0.858900

Thermal correction to Gibbs Free Energy= 0.715346

Sum of electronic and zero-point Energies= -2195.612522

Sum of electronic and thermal Energies= -2195.560174

Sum of electronic and thermal Enthalpies= -2195.559230

Sum of electronic and thermal Free Energies= -2195.702783

Center Number	Atomic Number	Atomic Type	Coordinates (Angstroms) X	Y	Z
1	77	0	1.574567	2.356883	-1.275110
2	6	0	1.956793	4.330737	-0.545039
3	6	0	2.624471	4.197868	-1.839067
4	6	0	2.535336	3.353440	0.355220
5	6	0	3.592862	3.133703	-1.717901
6	6	0	3.559304	2.612071	-0.370719
7	7	0	1.505683	0.678919	-2.667587
8	7	0	-2.070778	0.965931	-0.503695
9	6	0	-0.890595	1.460648	-0.789117
10	6	0	0.878462	-0.340591	-2.254942
11	7	0	0.312890	1.015498	-0.256713
12	7	0	-0.485702	2.444783	-1.680564
13	6	0	-1.245575	3.553945	-2.042470
14	6	0	-0.924974	4.244504	-3.229169
15	6	0	-2.294708	4.063820	-1.243487
16	6	0	-1.618649	5.399132	-3.600533
17	1	0	-0.121497	3.854624	-3.856230
18	6	0	-2.989006	5.210435	-1.633134
19	6	0	-2.669506	5.904989	-2.814968
20	1	0	-1.343793	5.916573	-4.525092
21	1	0	-3.795926	5.586566	-0.996084

22	6	0	0.538426	0.117829	0.777784
23	6	0	-0.447082	-0.305070	1.698870
24	6	0	1.847295	-0.398022	0.925101
25	6	0	-0.107327	-1.188696	2.730973
26	1	0	-1.466138	0.061755	1.583000
27	6	0	2.166950	-1.273353	1.960998
28	6	0	1.196032	-1.685572	2.895286
29	1	0	-0.885646	-1.501918	3.434628
30	1	0	3.190010	-1.654510	2.043850
31	7	0	0.404689	-1.436866	-2.064084
32	6	0	-0.679072	-2.040986	-2.761882
33	6	0	-1.971203	-1.999795	-2.228926
34	6	0	-0.381544	-2.633890	-4.007154
35	6	0	-3.026695	-2.552861	-2.959505
36	1	0	-2.132355	-1.485277	-1.278787
37	6	0	-1.474333	-3.162486	-4.719293
38	6	0	-2.777812	-3.130748	-4.210594
39	1	0	-4.042251	-2.506038	-2.560716
40	1	0	-1.284077	-3.616102	-5.696451
41	1	0	-3.598340	-3.552207	-4.795483
42	6	0	1.857848	0.690377	-4.073059
43	6	0	1.862117	1.955010	-4.681263
44	6	0	2.109258	-0.475411	-4.837341
45	6	0	2.092242	2.094834	-6.049406
46	1	0	1.667641	2.820493	-4.048506
47	6	0	2.328168	-0.300471	-6.217509
48	6	0	2.319042	0.954572	-6.830086
49	1	0	2.089429	3.090267	-6.499233
50	1	0	2.534006	-1.192686	-6.816468
51	1	0	2.501593	1.042078	-7.903169

52	6	0	2.215859	-1.887373	-4.291792
53	1	0	3.067482	-2.355420	-4.813284
54	1	0	2.484670	-1.894579	-3.228908
55	6	0	1.016444	-2.839725	-4.578535
56	1	0	0.907246	-2.909213	-5.674037
57	1	0	1.348596	-3.842292	-4.251442
58	1	0	-2.554471	3.550117	-0.316317
59	1	0	2.598640	-0.097479	0.191941
60	6	0	-3.167417	1.132035	-1.346578
61	6	0	-3.079387	1.158150	-2.761849
62	6	0	-4.461345	1.168954	-0.779673
63	6	0	-4.225982	1.234777	-3.552544
64	1	0	-2.093179	1.113504	-3.228934
65	6	0	-5.603340	1.253842	-1.582637
66	1	0	-4.551740	1.129545	0.309617
67	6	0	-5.513308	1.293101	-2.984772
68	1	0	-4.123082	1.244999	-4.642829
69	1	0	-6.591013	1.285479	-1.110980
70	6	0	-6.744252	1.413759	-3.850641
71	1	0	-6.666190	0.781661	-4.750409
72	1	0	-7.649817	1.117768	-3.298147
73	1	0	-6.892932	2.452351	-4.197265
74	6	0	-3.448370	7.126533	-3.237781
75	1	0	-4.354282	6.846667	-3.804874
76	1	0	-3.779744	7.711280	-2.364795
77	1	0	-2.844472	7.780741	-3.885971
78	6	0	1.550878	-2.614464	4.030879
79	1	0	2.121908	-2.089508	4.817391
80	1	0	0.646145	-3.031415	4.499671
81	1	0	2.177242	-3.452906	3.683766

82	6	0	2.182556	3.150657	1.793776
83	1	0	2.223052	2.081983	2.053207
84	1	0	2.893541	3.696196	2.437534
85	1	0	1.168972	3.520086	2.004651
86	6	0	0.927901	5.361565	-0.212046
87	1	0	0.319566	5.049114	0.648586
88	1	0	1.420739	6.317023	0.037967
89	1	0	0.253871	5.527823	-1.065776
90	6	0	2.445348	5.125899	-2.996779
91	1	0	1.388976	5.411370	-3.114719
92	1	0	3.029596	6.047835	-2.830659
93	1	0	2.796625	4.674445	-3.936273
94	6	0	4.531334	2.664603	-2.782220
95	1	0	4.169716	2.928433	-3.784764
96	1	0	5.520235	3.131955	-2.633816
97	1	0	4.661121	1.572959	-2.736793
98	6	0	4.531011	1.638517	0.214480
99	1	0	4.796604	0.853121	-0.509332
100	1	0	5.460442	2.161822	0.500940
101	1	0	4.120532	1.159797	1.114755

Structure 3*

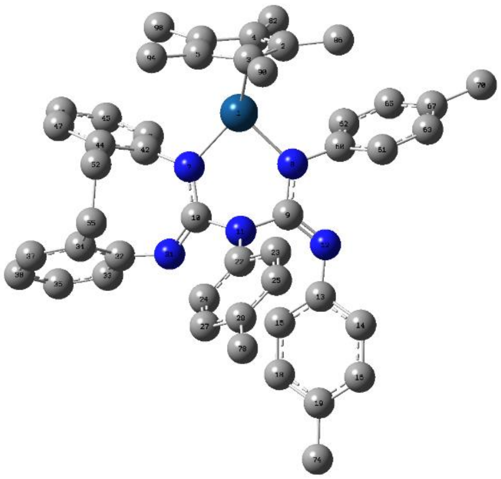

Zero-point correction= 0.808571 (Hartree/Particle)

Thermal correction to Energy= 0.860230

Thermal correction to Enthalpy= 0.861175

Thermal correction to Gibbs Free Energy= 0.720262

Sum of electronic and zero-point Energies= -2195.646531

Sum of electronic and thermal Energies= -2195.594872

Sum of electronic and thermal Enthalpies= -2195.593928

Sum of electronic and thermal Free Energies= -2195.734840

Center Number	Atomic Number	Atomic Type	Coordinates (Angstroms) X	Y	Z
1	77	0	1.486780	2.593655	-1.156024
2	6	0	2.797169	3.924166	0.036108
3	6	0	2.378257	4.543819	-1.202154
4	6	0	3.490902	2.710989	-0.311919
5	6	0	2.927546	3.772803	-2.316309
6	6	0	3.601096	2.635032	-1.771541
7	7	0	0.527533	1.359114	-2.451451

8	7	0	-0.017397	2.226261	0.130235
9	6	0	-1.313921	1.916899	-0.205292
10	6	0	-0.813683	1.080506	-2.474733
11	7	0	-1.588734	1.898388	-1.599775
12	7	0	-2.225712	1.726051	0.708947
13	6	0	-3.506678	1.252289	0.416572
14	6	0	-4.582413	1.751904	1.183882
15	6	0	-3.789065	0.239831	-0.533631
16	6	0	-5.885035	1.284312	0.991601
17	1	0	-4.372338	2.519659	1.933332
18	6	0	-5.093251	-0.229770	-0.702052
19	6	0	-6.169460	0.285425	0.044022
20	1	0	-6.699998	1.695911	1.595369
21	1	0	-5.284064	-1.018097	-1.437654
22	6	0	-2.648368	2.713537	-2.083238
23	6	0	-2.875400	3.949796	-1.442932
24	6	0	-3.460021	2.365992	-3.176984
25	6	0	-3.893154	4.799260	-1.876446
26	1	0	-2.244929	4.237908	-0.598424
27	6	0	-4.459310	3.243785	-3.615614
28	6	0	-4.707544	4.466828	-2.974928
29	1	0	-4.048273	5.751548	-1.360321
30	1	0	-5.076814	2.954558	-4.471419
31	7	0	-1.473975	0.171763	-3.130840
32	6	0	-1.143122	-0.527895	-4.287540
33	6	0	-1.233715	-1.938940	-4.269492
34	6	0	-0.917059	0.127272	-5.532021
35	6	0	-1.075915	-2.688627	-5.436935
36	1	0	-1.429186	-2.431256	-3.313336
37	6	0	-0.784394	-0.647144	-6.692991

38	6	0	-0.858213	-2.045188	-6.663941
39	1	0	-1.136939	-3.779209	-5.389877
40	1	0	-0.610544	-0.129655	-7.642015
41	1	0	-0.750564	-2.623728	-7.584322
42	6	0	1.385560	0.590180	-3.300702
43	6	0	2.059949	-0.517837	-2.769536
44	6	0	1.570314	0.973636	-4.643606
45	6	0	2.926775	-1.265116	-3.571045
46	1	0	1.893605	-0.772896	-1.720071
47	6	0	2.465348	0.225686	-5.426014
48	6	0	3.140176	-0.883794	-4.902660
49	1	0	3.443781	-2.133233	-3.155404
50	1	0	2.613778	0.513379	-6.470718
51	1	0	3.826140	-1.452918	-5.534442
52	6	0	0.738034	2.073936	-5.247767
53	1	0	0.687201	2.923894	-4.552910
54	1	0	1.227501	2.418935	-6.171410
55	6	0	-0.734470	1.620094	-5.580250
56	1	0	-1.420963	2.099685	-4.865305
57	1	0	-0.996398	1.989786	-6.583460
58	1	0	-2.981972	-0.164974	-1.149201
59	1	0	-3.310280	1.405046	-3.665890
60	6	0	0.279629	2.282647	1.529478
61	6	0	-0.249088	3.297669	2.342384
62	6	0	1.158004	1.346944	2.086292
63	6	0	0.116515	3.379364	3.686785
64	1	0	-0.938330	4.022391	1.904808
65	6	0	1.522707	1.436112	3.435594
66	1	0	1.567982	0.567056	1.440480
67	6	0	1.010659	2.453097	4.258370

68	1	0	-0.288357	4.184146	4.307508
69	1	0	2.220062	0.705814	3.856642
70	6	0	1.386890	2.541455	5.716821
71	1	0	1.559229	3.585862	6.022165
72	1	0	0.579181	2.144969	6.356538
73	1	0	2.296815	1.960087	5.929504
74	6	0	-7.579696	-0.202082	-0.182612
75	1	0	-8.048402	0.317941	-1.037301
76	1	0	-7.599440	-1.280218	-0.408821
77	1	0	-8.211871	-0.019876	0.700469
78	6	0	-5.820559	5.381255	-3.424342
79	1	0	-6.145470	5.139086	-4.447801
80	1	0	-6.701190	5.289485	-2.764351
81	1	0	-5.506079	6.437210	-3.399513
82	6	0	4.148080	1.763286	0.633548
83	1	0	4.057969	0.726688	0.276005
84	1	0	5.223781	2.005518	0.699275
85	1	0	3.711101	1.834853	1.637857
86	6	0	2.604772	4.478748	1.407885
87	1	0	2.556655	3.682636	2.163707
88	1	0	3.449226	5.146344	1.653420
89	1	0	1.676776	5.063102	1.473727
90	6	0	1.623684	5.825969	-1.330921
91	1	0	0.966588	5.984384	-0.464081
92	1	0	2.330104	6.673003	-1.389397
93	1	0	1.010898	5.828009	-2.244028
94	6	0	2.889438	4.170761	-3.753572
95	1	0	1.959080	4.702593	-3.999668
96	1	0	3.733673	4.849761	-3.967021
97	1	0	2.980351	3.293581	-4.408792

98	6	0	4.415348	1.639488	-2.524969
99	1	0	4.006016	1.462595	-3.529384
100	1	0	5.448493	2.016489	-2.627990
101	1	0	4.452075	0.676791	-1.998087

Structure 3

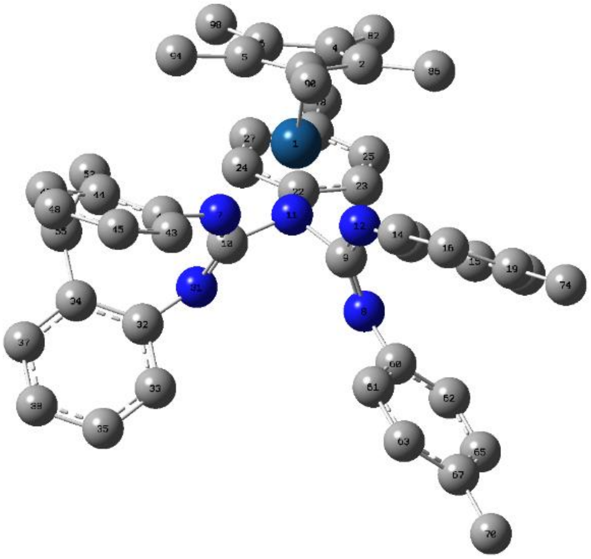

Zero-point correction= 0.807824 (Hartree/Particle)

Thermal correction to Energy= 0.859938

Thermal correction to Enthalpy= 0.860882

Thermal correction to Gibbs Free Energy= 0.718456

Sum of electronic and zero-point Energies= -2195.651440

Sum of electronic and thermal Energies= -2195.599326

Sum of electronic and thermal Enthalpies= -2195.598381

Sum of electronic and thermal Free Energies= -2195.740808

Center Number	Atomic Number	Atomic Type	Coordinates (Angstroms) X	Y	Z
1	77	0	1.432676	2.361467	-1.644351
2	6	0	2.087784	4.225883	-0.739839
3	6	0	2.578087	4.111275	-2.113569

4	6	0	2.612123	3.113460	0.005772
5	6	0	3.463507	2.954999	-2.175413
6	6	0	3.468958	2.321524	-0.885144
7	7	0	1.033427	0.875847	-3.057272
8	7	0	-2.184823	0.764120	-0.912053
9	6	0	-1.063000	1.363574	-1.142914
10	6	0	0.437880	-0.084706	-2.323088
11	7	0	0.215750	0.592745	-1.005268
12	7	0	-0.640160	2.569370	-1.570075
13	6	0	-1.427475	3.712521	-1.754784
14	6	0	-1.131500	4.562621	-2.834292
15	6	0	-2.457703	4.072578	-0.864722
16	6	0	-1.864804	5.734864	-3.034111
17	1	0	-0.314602	4.281158	-3.502862
18	6	0	-3.185279	5.245033	-1.077794
19	6	0	-2.910843	6.095090	-2.165426
20	1	0	-1.623421	6.384404	-3.880847
21	1	0	-3.984410	5.512341	-0.379938
22	6	0	0.506150	-0.090737	0.218186
23	6	0	-0.091508	0.332227	1.414381
24	6	0	1.490024	-1.090333	0.248770
25	6	0	0.297692	-0.244776	2.626294
26	1	0	-0.853265	1.112653	1.392681
27	6	0	1.868066	-1.655345	1.470043
28	6	0	1.280974	-1.247514	2.680450
29	1	0	-0.171947	0.096317	3.553130
30	1	0	2.638157	-2.431740	1.481394
31	7	0	0.002567	-1.281016	-2.500604
32	6	0	-0.031991	-1.862147	-3.783142
33	6	0	-1.234728	-1.803436	-4.515976

34	6	0	1.071887	-2.576131	-4.305924
35	6	0	-1.346002	-2.435238	-5.758491
36	1	0	-2.076819	-1.251683	-4.089228
37	6	0	0.936365	-3.205265	-5.552870
38	6	0	-0.258032	-3.146448	-6.281740
39	1	0	-2.284778	-2.373836	-6.315067
40	1	0	1.797409	-3.745397	-5.958701
41	1	0	-0.336927	-3.648056	-7.249051
42	6	0	1.638571	0.727262	-4.320736
43	6	0	1.309949	1.662632	-5.319587
44	6	0	2.670060	-0.215855	-4.544068
45	6	0	1.962876	1.645872	-6.553594
46	1	0	0.537495	2.403271	-5.097712
47	6	0	3.308127	-0.219652	-5.794660
48	6	0	2.965515	0.694710	-6.796403
49	1	0	1.694112	2.373712	-7.322923
50	1	0	4.106080	-0.947294	-5.970683
51	1	0	3.485686	0.675085	-7.756886
52	6	0	3.081779	-1.186201	-3.467943
53	1	0	4.172296	-1.336482	-3.515169
54	1	0	2.862699	-0.737406	-2.487169
55	6	0	2.392480	-2.591980	-3.578023
56	1	0	3.070913	-3.275716	-4.111450
57	1	0	2.260298	-2.992058	-2.557813
58	1	0	-2.683204	3.426935	-0.014426
59	1	0	1.949535	-1.427793	-0.680936
60	6	0	-3.391329	1.238688	-1.439538
61	6	0	-3.506224	1.664983	-2.783684
62	6	0	-4.555520	1.226070	-0.646298
63	6	0	-4.735454	2.078711	-3.296982

64	1	0	-2.611975	1.674543	-3.412303
65	6	0	-5.779289	1.657796	-1.169039
66	1	0	-4.480336	0.889595	0.391098
67	6	0	-5.896610	2.091953	-2.500432
68	1	0	-4.797578	2.408304	-4.338925
69	1	0	-6.667267	1.656306	-0.529221
70	6	0	-7.224309	2.529186	-3.070006
71	1	0	-7.109654	3.412658	-3.718875
72	1	0	-7.678111	1.731995	-3.685214
73	1	0	-7.938186	2.776992	-2.269329
74	6	0	-3.730082	7.339524	-2.403347
75	1	0	-4.653871	7.103185	-2.960641
76	1	0	-4.033799	7.806123	-1.452950
77	1	0	-3.168897	8.080328	-2.993472
78	6	0	1.671487	-1.886518	3.989665
79	1	0	2.720217	-2.221258	3.972960
80	1	0	1.538887	-1.187296	4.829603
81	1	0	1.046280	-2.772798	4.196639
82	6	0	2.375637	2.803955	1.448043
83	1	0	2.276521	1.718768	1.603179
84	1	0	3.222487	3.164145	2.057512
85	1	0	1.456977	3.286380	1.810878
86	6	0	1.207273	5.315977	-0.220487
87	1	0	0.646746	4.981434	0.664278
88	1	0	1.815196	6.192066	0.064243
89	1	0	0.482062	5.630289	-0.985712
90	6	0	2.376628	5.108346	-3.209122
91	1	0	1.430704	5.652290	-3.075552
92	1	0	3.198947	5.845438	-3.211161
93	1	0	2.360747	4.612011	-4.191405

94	6	0	4.230770	2.527977	-3.382686
95	1	0	3.638286	2.671990	-4.298512
96	1	0	5.153045	3.128562	-3.466170
97	1	0	4.508721	1.466909	-3.324511
98	6	0	4.250160	1.115485	-0.474543
99	1	0	4.543529	0.518582	-1.350032
100	1	0	5.166692	1.416146	0.062053
101	1	0	3.656156	0.475506	0.196813

TS2

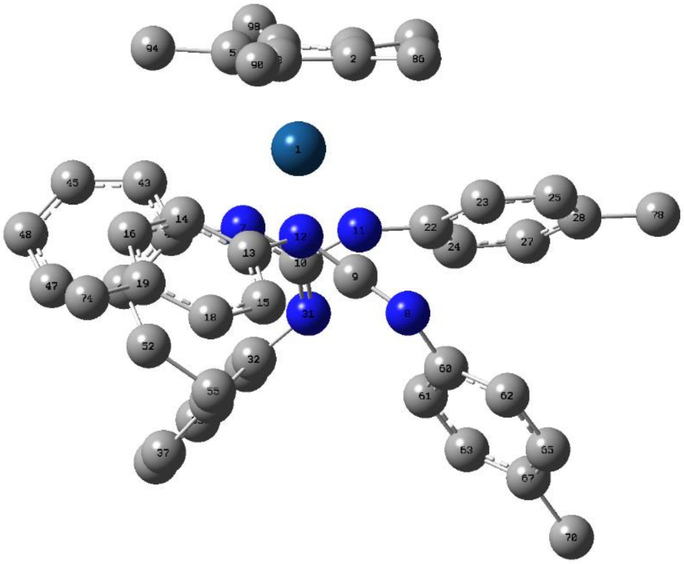

Zero-point correction= 0.805936 (Hartree/Particle)

Thermal correction to Energy= 0.858165

Thermal correction to Enthalpy= 0.859109

Thermal correction to Gibbs Free Energy= 0.716335

Sum of electronic and zero-point Energies= -2195.629084

Sum of electronic and thermal Energies= -2195.576855

Sum of electronic and thermal Enthalpies= -2195.575911

Sum of electronic and thermal Free Energies= -2195.718685

Center	Atomic	Atomic	Coordinates (Angstroms)		
Number	Number	Type	X	Y	Z
1	77	0	2.209303	2.655380	-1.538383
2	6	0	3.402529	3.332690	0.159982
3	6	0	3.441762	4.348590	-0.881333
4	6	0	3.969803	2.122965	-0.402214
5	6	0	4.003857	3.770750	-2.074323

6	6	0	4.335605	2.375499	-1.778290
7	7	0	1.264854	1.876056	-3.252863
8	7	0	-1.375095	2.411990	0.099938
9	6	0	-0.512239	2.728982	-0.712810
10	6	0	0.651239	0.807707	-2.670636
11	7	0	0.889224	1.016499	-1.290739
12	7	0	0.268300	3.534385	-1.329888
13	6	0	-0.174156	4.785096	-1.843802
14	6	0	0.612881	5.422875	-2.815371
15	6	0	-1.373526	5.383244	-1.421965
16	6	0	0.207698	6.645611	-3.351383
17	1	0	1.522929	4.926225	-3.157139
18	6	0	-1.767377	6.606552	-1.972109
19	6	0	-0.986962	7.263868	-2.939748
20	1	0	0.825694	7.125495	-4.115652
21	1	0	-2.705406	7.061820	-1.641984
22	6	0	0.886150	0.049380	-0.279718
23	6	0	0.944446	0.506004	1.055364
24	6	0	0.847142	-1.343509	-0.503250
25	6	0	0.942967	-0.389085	2.123914
26	1	0	0.992520	1.582226	1.231684
27	6	0	0.846801	-2.228469	0.580736
28	6	0	0.885585	-1.779602	1.911484
29	1	0	0.987610	-0.002834	3.147146
30	1	0	0.816727	-3.305236	0.384572
31	7	0	-0.021354	-0.208750	-3.137105
32	6	0	-0.566816	-0.298176	-4.416380
33	6	0	-0.349912	-1.488426	-5.153799
34	6	0	-1.464270	0.670437	-4.952849
35	6	0	-0.969192	-1.710865	-6.384980

36	1	0	0.324568	-2.236366	-4.727911
37	6	0	-2.085457	0.419528	-6.185875
38	6	0	-1.850290	-0.754229	-6.911518
39	1	0	-0.770785	-2.637100	-6.931411
40	1	0	-2.771388	1.175846	-6.582595
41	1	0	-2.350188	-0.923285	-7.868233
42	6	0	1.455308	2.096744	-4.632664
43	6	0	2.677296	1.695048	-5.204521
44	6	0	0.488280	2.750488	-5.435848
45	6	0	2.974837	1.950174	-6.545808
46	1	0	3.381652	1.161481	-4.566221
47	6	0	0.806014	2.991202	-6.784245
48	6	0	2.033527	2.614575	-7.341562
49	1	0	3.930007	1.625616	-6.966435
50	1	0	0.056752	3.484100	-7.411204
51	1	0	2.245113	2.825227	-8.392650
52	6	0	-0.877426	3.143066	-4.921856
53	1	0	-0.791806	3.967915	-4.198266
54	1	0	-1.451924	3.529039	-5.779896
55	6	0	-1.692911	1.991192	-4.263055
56	1	0	-1.432395	1.916661	-3.198462
57	1	0	-2.760512	2.262171	-4.316019
58	1	0	-1.996043	4.887272	-0.673736
59	1	0	0.796905	-1.711476	-1.527311
60	6	0	-2.261303	1.332191	0.216048
61	6	0	-2.347247	0.312270	-0.751173
62	6	0	-3.074235	1.280108	1.360951
63	6	0	-3.238810	-0.742169	-0.556026
64	1	0	-1.692985	0.323867	-1.626638
65	6	0	-3.966363	0.218725	1.534519

66	1	0	-2.990583	2.072037	2.108475
67	6	0	-4.066320	-0.809739	0.581581
68	1	0	-3.289647	-1.538863	-1.304113
69	1	0	-4.593263	0.182493	2.429703
70	6	0	-5.041089	-1.946108	0.759565
71	1	0	-5.933427	-1.800407	0.125953
72	1	0	-4.589251	-2.907076	0.466564
73	1	0	-5.380633	-2.021027	1.803566
74	6	0	-1.405359	8.599192	-3.501946
75	1	0	-1.061940	8.719249	-4.541124
76	1	0	-2.499474	8.717384	-3.477280
77	1	0	-0.970507	9.426962	-2.914430
78	6	0	0.838791	-2.746137	3.069481
79	1	0	1.206965	-3.740908	2.773363
80	1	0	1.446492	-2.387811	3.915869
81	1	0	-0.193660	-2.874317	3.441382
82	6	0	4.205233	0.835383	0.317195
83	1	0	4.098137	-0.019280	-0.366799
84	1	0	5.230927	0.826061	0.725332
85	1	0	3.495603	0.701669	1.144564
86	6	0	2.993820	3.555787	1.580935
87	1	0	2.732365	2.605469	2.068284
88	1	0	3.819289	4.014326	2.153526
89	1	0	2.125449	4.229210	1.640982
90	6	0	3.012249	5.765992	-0.682159
91	1	0	2.061850	5.816562	-0.130161
92	1	0	3.778595	6.297512	-0.091844
93	1	0	2.882610	6.291419	-1.636855
94	6	0	4.281355	4.460331	-3.372321
95	1	0	3.711131	5.396983	-3.455763

96	1	0	5.353988	4.706956	-3.454645
97	1	0	4.011803	3.813034	-4.222277
98	6	0	5.133376	1.451501	-2.642276
99	1	0	5.151553	1.799953	-3.683697
100	1	0	6.176392	1.416827	-2.281766
101	1	0	4.733940	0.426616	-2.615616

Structure 4

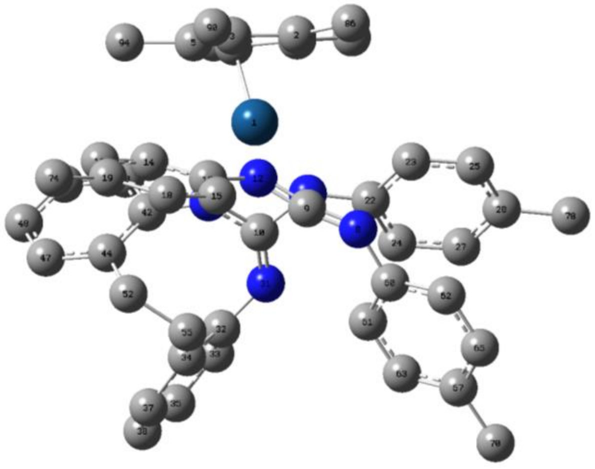

Zero-point correction= 0.806509 (Hartree/Particle)

Thermal correction to Energy= 0.859433

Thermal correction to Enthalpy= 0.860377

Thermal correction to Gibbs Free Energy= 0.716781

Sum of electronic and zero-point Energies= -2195.631862

Sum of electronic and thermal Energies= -2195.578937

Sum of electronic and thermal Enthalpies= -2195.577993

Sum of electronic and thermal Free Energies= -2195.721589

Center Number	Atomic Number	Atomic Type	Coordinates (Angstroms) X	Y	Z
1	77	0	2.188212	2.599747	-1.526360
2	6	0	3.354547	3.374374	0.165319
3	6	0	3.306587	4.390258	-0.881217
4	6	0	3.983452	2.205601	-0.397691
5	6	0	3.892400	3.854652	-2.076224
6	6	0	4.299232	2.475389	-1.792851
7	7	0	1.175662	1.931269	-3.243529
8	7	0	-1.247259	2.361786	0.398415
9	6	0	-0.604937	2.925694	-0.463280
10	6	0	0.731482	0.735541	-2.712907
11	7	0	1.115022	0.822409	-1.381695
12	7	0	0.185830	3.561933	-1.204023
13	6	0	-0.157795	4.812354	-1.808554
14	6	0	0.561681	5.245458	-2.929598
15	6	0	-1.173341	5.622784	-1.277041
16	6	0	0.263275	6.479149	-3.512374
17	1	0	1.336305	4.600515	-3.345285
18	6	0	-1.461724	6.853384	-1.872535
19	6	0	-0.751129	7.305810	-2.999150
20	1	0	0.829376	6.804097	-4.389910
21	1	0	-2.249781	7.480911	-1.447253
22	6	0	0.926122	-0.079417	-0.346343

23	6	0	1.142383	0.375144	0.975998
24	6	0	0.521611	-1.426776	-0.514950
25	6	0	0.945168	-0.459161	2.074047
26	1	0	1.444874	1.415056	1.112251
27	6	0	0.325591	-2.248726	0.599927
28	6	0	0.521136	-1.792658	1.914118
29	1	0	1.113335	-0.065432	3.082025
30	1	0	0.004758	-3.283929	0.440966
31	7	0	0.117729	-0.290922	-3.243084
32	6	0	-0.491674	-0.340933	-4.492569
33	6	0	-0.262125	-1.500651	-5.278743
34	6	0	-1.446711	0.605007	-4.969262
35	6	0	-0.920280	-1.714912	-6.489889
36	1	0	0.457690	-2.232606	-4.901930
37	6	0	-2.107364	0.361221	-6.183990
38	6	0	-1.859563	-0.780828	-6.953526
39	1	0	-0.707311	-2.617531	-7.069531
40	1	0	-2.837069	1.101383	-6.530469
41	1	0	-2.392506	-0.943001	-7.893457
42	6	0	1.399605	2.167907	-4.617026
43	6	0	2.645185	1.819745	-5.172134
44	6	0	0.417991	2.781373	-5.434339
45	6	0	2.954626	2.097583	-6.506260
46	1	0	3.361235	1.314942	-4.523020
47	6	0	0.747593	3.049033	-6.774988
48	6	0	2.000051	2.730314	-7.312660
49	1	0	3.929592	1.817232	-6.913392
50	1	0	-0.012137	3.512095	-7.412158
51	1	0	2.221213	2.959239	-8.358000
52	6	0	-0.976979	3.092342	-4.940568

53	1	0	-0.958211	3.943104	-4.241761
54	1	0	-1.573078	3.407896	-5.813072
55	6	0	-1.704734	1.904520	-4.250307
56	1	0	-1.381654	1.841273	-3.203828
57	1	0	-2.785562	2.125367	-4.248832
58	1	0	-1.726088	5.293911	-0.392872
59	1	0	0.343370	-1.794479	-1.524409
60	6	0	-2.126594	1.266678	0.434187
61	6	0	-2.262257	0.385771	-0.654437
62	6	0	-2.856916	1.049269	1.612431
63	6	0	-3.138312	-0.693312	-0.554502
64	1	0	-1.657902	0.524464	-1.553573
65	6	0	-3.730814	-0.038298	1.693364
66	1	0	-2.731980	1.735727	2.452353
67	6	0	-3.885196	-0.928109	0.615937
68	1	0	-3.233435	-1.379938	-1.400376
69	1	0	-4.301666	-0.203847	2.610896
70	6	0	-4.797628	-2.123668	0.717564
71	1	0	-5.382433	-2.258266	-0.206198
72	1	0	-4.212046	-3.047326	0.868232
73	1	0	-5.493591	-2.025025	1.563946
74	6	0	-1.087894	8.623115	-3.651139
75	1	0	-1.894690	8.496462	-4.394210
76	1	0	-1.437822	9.357827	-2.909660
77	1	0	-0.216872	9.041522	-4.178007
78	6	0	0.250801	-2.678182	3.106262
79	1	0	0.306253	-3.743547	2.831886
80	1	0	0.972067	-2.492421	3.918835
81	1	0	-0.758033	-2.497354	3.520452
82	6	0	4.337229	0.939741	0.312571

83	1	0	4.194657	0.071184	-0.346971
84	1	0	5.399451	0.974651	0.611393
85	1	0	3.722209	0.792357	1.209328
86	6	0	2.948703	3.593145	1.588450
87	1	0	2.862857	2.640775	2.130632
88	1	0	3.701776	4.210565	2.109027
89	1	0	1.983133	4.118151	1.649201
90	6	0	2.799997	5.778670	-0.665180
91	1	0	1.862619	5.772683	-0.088983
92	1	0	3.548758	6.350028	-0.089240
93	1	0	2.616056	6.299278	-1.613517
94	6	0	4.110704	4.567389	-3.372403
95	1	0	3.487946	5.471076	-3.437137
96	1	0	5.167260	4.871836	-3.466880
97	1	0	3.865068	3.913976	-4.224645
98	6	0	5.159802	1.610041	-2.657647
99	1	0	5.159080	1.962421	-3.697964
100	1	0	6.200971	1.645436	-2.291374
101	1	0	4.830093	0.560727	-2.634855

Structure 5

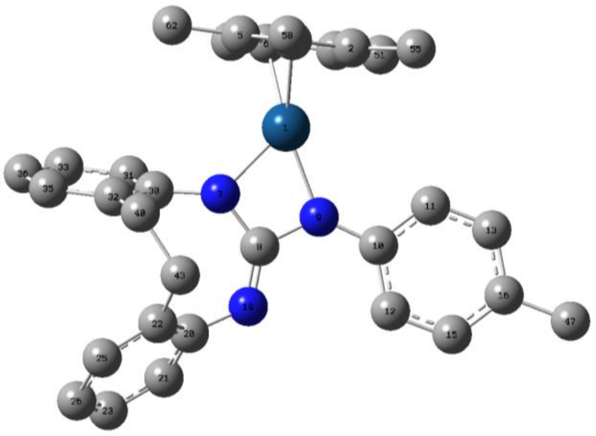

Zero-point correction= 0.561713 (Hartree/Particle)

Thermal correction to Energy= 0.597480

Thermal correction to Enthalpy= 0.598425

Thermal correction to Gibbs Free Energy= 0.492714

Sum of electronic and zero-point Energies= -1507.018869

Sum of electronic and thermal Energies= -1506.983101

Sum of electronic and thermal Enthalpies= -1506.982157

Sum of electronic and thermal Free Energies= -1507.087868

Center Number	Atomic Number	Atomic Type	Coordinates (Angstroms) X	Y	Z
1	77	0	2.527363	2.119433	-1.691070
2	6	0	3.372243	3.274202	-0.015785
3	6	0	3.034123	4.137546	-1.146827
4	6	0	4.334226	2.315793	-0.475387
5	6	0	3.842764	3.754991	-2.283839
6	6	0	4.612426	2.601405	-1.888692
7	7	0	1.422569	1.609008	-3.268341
8	6	0	0.696538	0.587878	-2.656785
9	7	0	1.166096	0.674485	-1.333314
10	6	0	0.739320	-0.037070	-0.215187
11	6	0	1.471441	0.129631	0.982670
12	6	0	-0.375529	-0.908754	-0.189957
13	6	0	1.111165	-0.538893	2.151333
14	1	0	2.334555	0.795953	0.959734
15	6	0	-0.720990	-1.571471	0.992715
16	6	0	0.002307	-1.405492	2.185955
17	1	0	1.705522	-0.389086	3.058534
18	1	0	-1.585465	-2.243339	0.985805

19	7	0	-0.155690	-0.265052	-3.116067
20	6	0	-0.699763	-0.204169	-4.406530
21	6	0	-0.687963	-1.392473	-5.173388
22	6	0	-1.384547	0.933819	-4.916911
23	6	0	-1.298344	-1.455135	-6.426497
24	1	0	-0.178593	-2.265485	-4.757335
25	6	0	-2.010879	0.837690	-6.170203
26	6	0	-1.972562	-0.333994	-6.932820
27	1	0	-1.259411	-2.383121	-7.002789
28	1	0	-2.536438	1.718394	-6.553420
29	1	0	-2.468212	-0.375698	-7.905508
30	6	0	1.674604	1.801245	-4.644126
31	6	0	2.821100	1.226768	-5.219010
32	6	0	0.847585	2.656123	-5.402809
33	6	0	3.150433	1.478878	-6.554374
34	1	0	3.447080	0.589589	-4.588997
35	6	0	1.190329	2.892508	-6.743368
36	6	0	2.330113	2.318093	-7.320556
37	1	0	4.041741	1.024291	-6.993798
38	1	0	0.546694	3.544914	-7.340876
39	1	0	2.576116	2.524807	-8.364848
40	6	0	-0.382690	3.280009	-4.791667
41	1	0	-0.082750	3.979583	-3.991450
42	1	0	-0.887721	3.871976	-5.571399
43	6	0	-1.398966	2.255038	-4.187994
44	1	0	-1.180025	2.104294	-3.122176
45	1	0	-2.405852	2.698770	-4.253883
46	1	0	-0.949325	-1.061657	-1.102800
47	6	0	-0.405766	-2.105120	3.459561
48	1	0	-1.015420	-2.996885	3.245782

49	1	0	0.474164	-2.418824	4.044263
50	1	0	-1.006656	-1.442298	4.107924
51	6	0	5.027855	1.266505	0.331810
52	1	0	5.156824	0.343381	-0.252805
53	1	0	6.029334	1.621308	0.632095
54	1	0	4.466383	1.021696	1.244301
55	6	0	2.844218	3.444990	1.372654
56	1	0	3.041937	2.559372	1.992043
57	1	0	3.327623	4.312219	1.855208
58	6	0	2.074736	5.280563	-1.092687
59	1	0	1.249543	5.064101	-0.398751
60	1	0	2.592723	6.188602	-0.736349
61	1	0	1.655157	5.492529	-2.086500
62	6	0	3.838138	4.393814	-3.637155
63	1	0	2.880149	4.899886	-3.826562
64	1	0	4.645582	5.141282	-3.716873
65	1	0	3.980488	3.638922	-4.425234
66	6	0	5.593492	1.865191	-2.740854
67	1	0	5.332009	1.955752	-3.804881
68	1	0	6.606257	2.281022	-2.596314
69	1	0	5.621151	0.798568	-2.473298
70	1	0	1.758350	3.623686	1.364557

L1

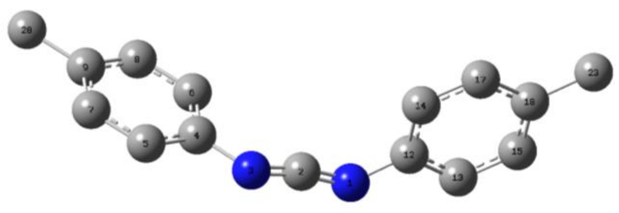

Zero-point correction= 0.242126 (Hartree/Particle)

Thermal correction to Energy= 0.258427

Thermal correction to Enthalpy= 0.259371

Thermal correction to Gibbs Free Energy= 0.193137

Sum of electronic and zero-point Energies= -688.596271

Sum of electronic and thermal Energies= -688.579969

Sum of electronic and thermal Enthalpies= -688.579025

Sum of electronic and thermal Free Energies= -688.645259

Center Number	Atomic Number	Atomic Type	Coordinates (Angstroms) X	Y	Z
1	7	0	-6.399445	1.442492	-4.438372
2	6	0	-6.655128	0.364369	-3.903742
3	7	0	-7.104145	-0.694419	-3.466790
4	6	0	-7.043872	-1.243954	-2.170555
5	6	0	-7.657245	-2.487166	-1.949911
6	6	0	-6.400725	-0.590392	-1.100507
7	6	0	-7.626880	-3.065409	-0.676873
8	6	0	-6.379462	-1.180266	0.163293
9	6	0	-6.988816	-2.428034	0.400210
10	1	0	-8.111124	-4.032344	-0.514762
11	1	0	-5.880121	-0.662301	0.987198
12	6	0	-5.243124	1.869858	-5.121245
13	6	0	-5.226317	3.174086	-5.641057
14	6	0	-4.120335	1.038517	-5.302964
15	6	0	-4.102017	3.636955	-6.331365
16	1	0	-6.101723	3.812066	-5.502005
17	6	0	-3.006160	1.515393	-5.994172

18	6	0	-2.972827	2.821880	-6.518731
19	1	0	-4.101351	4.652779	-6.736192
20	1	0	-2.141337	0.860248	-6.132772
21	1	0	-5.923631	0.378742	-1.269889
22	1	0	-4.132288	0.021384	-4.901980
23	6	0	-1.750268	3.334836	-7.237764
24	1	0	-0.983869	3.672336	-6.518130
25	1	0	-1.291502	2.547578	-7.856414
26	1	0	-1.998053	4.189980	-7.884587
27	1	0	-8.157752	-2.987296	-2.781826
28	6	0	-6.937295	-3.064128	1.766837
29	1	0	-7.657859	-3.891678	1.847994
30	1	0	-5.932320	-3.471130	1.974109
31	1	0	-7.158248	-2.328977	2.557236

Structure 6

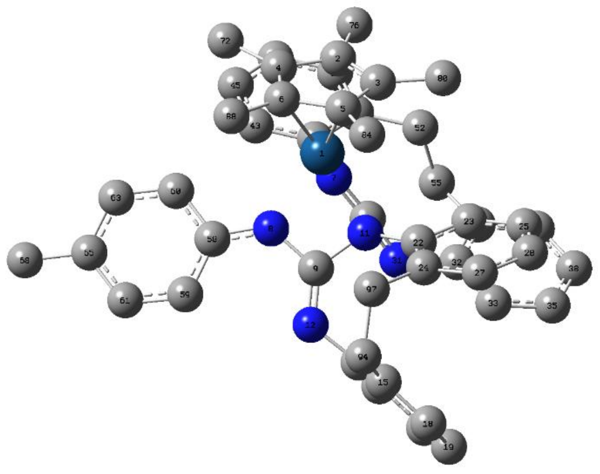

Zero-point correction= 0.789667 (Hartree/Particle)

Thermal correction to Energy= 0.839317

Thermal correction to Enthalpy= 0.840261

Thermal correction to Gibbs Free Energy= 0.707372

Sum of electronic and zero-point Energies= -2194.452174

Sum of electronic and thermal Energies= -2194.402524

Sum of electronic and thermal Enthalpies= -2194.401580

Sum of electronic and thermal Free Energies= -2194.534469

Center Number	Atomic Number	Atomic Type	Coordinates (Angstroms) X	Y	Z
1	77	0	-3.239672	1.989615	-0.522930
2	6	0	-1.317894	3.054302	-0.254969
3	6	0	-1.305457	2.317702	-1.491623
4	6	0	-1.507830	2.135589	0.849302
5	6	0	-1.521444	0.910515	-1.163772
6	6	0	-1.646016	0.805694	0.281046
7	7	0	-4.576643	3.761594	-0.377694
8	7	0	-4.939849	0.952408	0.125135
9	6	0	-5.559742	0.832669	-1.107909
10	6	0	-5.446168	3.956763	-1.264929
11	7	0	-4.670413	1.484446	-1.960631
12	7	0	-6.729461	0.305403	-1.334006
13	6	0	-7.356593	0.358862	-2.582167
14	6	0	-8.274352	1.395777	-2.861344
15	6	0	-7.190216	-0.681016	-3.534173
16	6	0	-9.003935	1.416070	-4.055440
17	1	0	-8.395852	2.190068	-2.120590
18	6	0	-7.926086	-0.638634	-4.727279
19	6	0	-8.833264	0.394720	-4.999360
20	1	0	-9.704240	2.233836	-4.247638
21	1	0	-7.775793	-1.438668	-5.459499
22	6	0	-4.442706	1.245432	-3.319397

23	6	0	-4.105302	2.357802	-4.122585
24	6	0	-4.421124	-0.050458	-3.903928
25	6	0	-3.801079	2.216574	-5.476453
26	1	0	-4.087403	3.342912	-3.651773
27	6	0	-4.126428	-0.168182	-5.272014
28	6	0	-3.820772	0.943448	-6.065178
29	1	0	-3.554635	3.099449	-6.071853
30	1	0	-4.118560	-1.169826	-5.713794
31	7	0	-6.344282	4.142312	-2.057407
32	6	0	-6.353919	4.905114	-3.254160
33	6	0	-6.834461	4.311072	-4.426827
34	6	0	-5.921452	6.252869	-3.222436
35	6	0	-6.853117	5.053086	-5.612332
36	1	0	-7.168523	3.272492	-4.396208
37	6	0	-5.967116	6.973188	-4.425079
38	6	0	-6.418394	6.384333	-5.613748
39	1	0	-7.212192	4.587332	-6.532601
40	1	0	-5.643701	8.017719	-4.418858
41	1	0	-6.438074	6.968299	-6.536520
42	6	0	-4.265448	4.851201	0.515009
43	6	0	-4.191960	4.568245	1.882442
44	6	0	-3.946139	6.128221	0.001350
45	6	0	-3.802275	5.569914	2.776373
46	1	0	-4.435414	3.559894	2.222122
47	6	0	-3.552332	7.112602	0.922793
48	6	0	-3.477256	6.845237	2.294317
49	1	0	-3.745584	5.350270	3.844730
50	1	0	-3.286663	8.103399	0.544392
51	1	0	-3.160539	7.629560	2.985190
52	6	0	-4.022988	6.439325	-1.474736

53	1	0	-3.306541	7.239988	-1.712017
54	1	0	-3.704332	5.557620	-2.056709
55	6	0	-5.456162	6.883330	-1.932030
56	1	0	-6.174528	6.628866	-1.134513
57	1	0	-5.477565	7.977976	-2.036344
58	6	0	-5.422648	0.613948	1.374764
59	6	0	-6.613269	-0.124867	1.608143
60	6	0	-4.670590	1.001384	2.511792
61	6	0	-7.008844	-0.431839	2.914172
62	1	0	-7.205905	-0.443241	0.751499
63	6	0	-5.080790	0.680966	3.805802
64	1	0	-3.748537	1.557355	2.341081
65	6	0	-6.265207	-0.041270	4.041623
66	1	0	-7.931817	-1.003087	3.061504
67	1	0	-4.465431	0.998748	4.654248
68	6	0	-6.728843	-0.358092	5.443044
69	1	0	-7.373127	-1.251532	5.456811
70	1	0	-5.875449	-0.537741	6.116932
71	1	0	-7.314057	0.474251	5.874656
72	6	0	-1.363767	2.483701	2.296744
73	1	0	-1.744628	1.679920	2.943420
74	1	0	-0.296912	2.629643	2.542169
75	1	0	-1.895423	3.415494	2.541961
76	6	0	-1.075551	4.520300	-0.109163
77	1	0	-1.563898	4.919477	0.790516
78	1	0	0.010197	4.700926	-0.020490
79	1	0	-1.441726	5.077368	-0.983280
80	6	0	-1.021012	2.860821	-2.853313
81	1	0	-1.271034	3.930493	-2.913192
82	1	0	0.050894	2.745828	-3.092709

83	1	0	-1.604840	2.329813	-3.619773
84	6	0	-1.483705	-0.217375	-2.141903
85	1	0	-1.962455	0.070158	-3.089998
86	1	0	-0.436218	-0.496178	-2.350769
87	1	0	-2.005304	-1.101302	-1.748716
88	6	0	-1.809051	-0.465207	1.050928
89	1	0	-2.309432	-1.229288	0.439146
90	1	0	-0.819585	-0.852844	1.348919
91	1	0	-2.410275	-0.302362	1.956728
92	1	0	-9.398600	0.400716	-5.934364
93	1	0	-3.587453	0.817422	-7.125034
94	6	0	-6.193984	-1.786313	-3.284203
95	1	0	-6.478482	-2.356355	-2.382251
96	1	0	-6.229834	-2.482211	-4.138092
97	6	0	-4.723388	-1.289538	-3.101043
98	1	0	-4.045435	-2.098342	-3.419958
99	1	0	-4.521102	-1.099488	-2.037819

Structure 7

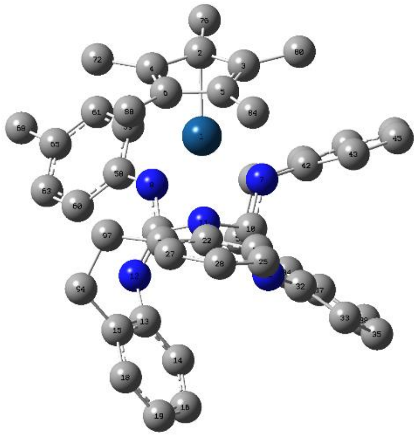

Zero-point correction= 0.791302 (Hartree/Particle)

Thermal correction to Energy= 0.840193

Thermal correction to Enthalpy= 0.841138

Thermal correction to Gibbs Free Energy= 0.708520

Sum of electronic and zero-point Energies= -2194.471735

Sum of electronic and thermal Energies= -2194.422844

Sum of electronic and thermal Enthalpies= -2194.421899

Sum of electronic and thermal Free Energies= -2194.554516

Center Number	Atomic Number	Atomic Type	Coordinates (Angstroms) X	Y	Z
1	77	0	-3.823106	2.541004	-0.711610
2	6	0	-2.013849	2.554587	0.524156
3	6	0	-1.808440	1.768926	-0.674370
4	6	0	-3.113439	1.978453	1.271194
5	6	0	-2.750726	0.657609	-0.654263
6	6	0	-3.551527	0.799513	0.541794
7	7	0	-4.399520	2.679214	-2.714259
8	7	0	-4.708994	4.438651	-0.527008
9	6	0	-6.019538	4.197654	-0.740912
10	6	0	-5.726895	2.828371	-2.648933
11	7	0	-6.009513	2.752540	-1.175172
12	7	0	-7.033189	4.980423	-0.713545
13	6	0	-8.360155	4.540719	-0.854286
14	6	0	-9.035139	4.664364	-2.081317
15	6	0	-9.047193	4.075938	0.292130
16	6	0	-10.385223	4.310351	-2.181753
17	1	0	-8.477929	5.014556	-2.951998
18	6	0	-10.398038	3.718133	0.165108
19	6	0	-11.073072	3.833860	-1.057345
20	1	0	-10.896960	4.401321	-3.143396
21	1	0	-10.925172	3.342736	1.047764
22	6	0	-6.917781	1.751472	-0.682593

23	6	0	-7.211292	0.656094	-1.516216
24	6	0	-7.384052	1.766798	0.650882
25	6	0	-7.982127	-0.409547	-1.048687
26	1	0	-6.836128	0.648353	-2.539540
27	6	0	-8.170748	0.688630	1.087652
28	6	0	-8.473918	-0.396455	0.262026
29	1	0	-8.197668	-1.247624	-1.715161
30	1	0	-8.537934	0.710261	2.117820
31	7	0	-6.700284	3.024070	-3.471148
32	6	0	-6.511406	3.484728	-4.779792
33	6	0	-7.156422	2.791899	-5.829381
34	6	0	-5.819332	4.694592	-5.077325
35	6	0	-7.089013	3.245783	-7.147941
36	1	0	-7.706426	1.880384	-5.580947
37	6	0	-5.778752	5.135647	-6.409261
38	6	0	-6.395271	4.427380	-7.446972
39	1	0	-7.585749	2.681406	-7.941564
40	1	0	-5.246304	6.066962	-6.628329
41	1	0	-6.344845	4.797884	-8.473512
42	6	0	-3.637859	2.663832	-3.907665
43	6	0	-3.331047	1.426892	-4.497465
44	6	0	-3.147307	3.864756	-4.461916
45	6	0	-2.510135	1.363949	-5.628580
46	1	0	-3.755545	0.522116	-4.058535
47	6	0	-2.323379	3.779376	-5.594998
48	6	0	-1.992533	2.546536	-6.171968
49	1	0	-2.278141	0.397253	-6.081736
50	1	0	-1.946804	4.706780	-6.036338
51	1	0	-1.345033	2.511676	-7.051321
52	6	0	-3.567033	5.200186	-3.907186

53	1	0	-3.283559	5.275003	-2.846161
54	1	0	-3.019009	5.983615	-4.453412
55	6	0	-5.108647	5.486174	-4.005740
56	1	0	-5.577688	5.298574	-3.030712
57	1	0	-5.233115	6.560839	-4.213923
58	6	0	-4.166654	5.671136	-0.149074
59	6	0	-2.808647	5.904413	-0.443556
60	6	0	-4.885867	6.683330	0.526909
61	6	0	-2.186603	7.100733	-0.085568
62	1	0	-2.254283	5.113834	-0.953022
63	6	0	-4.246936	7.875447	0.880975
64	1	0	-5.940110	6.529904	0.756042
65	6	0	-2.892900	8.114820	0.585571
66	1	0	-1.131848	7.252138	-0.334934
67	1	0	-4.820663	8.647599	1.403235
68	6	0	-2.217314	9.399656	0.997806
69	1	0	-1.416761	9.673773	0.292738
70	1	0	-2.937699	10.230937	1.047688
71	1	0	-1.755535	9.304845	1.996881
72	6	0	-3.599009	2.440770	2.607499
73	1	0	-4.625162	2.092832	2.791872
74	1	0	-2.953584	2.050587	3.413727
75	1	0	-3.592621	3.539622	2.666326
76	6	0	-1.149230	3.669447	1.006636
77	1	0	-1.736023	4.454864	1.503256
78	1	0	-0.433667	3.256305	1.739790
79	1	0	-0.571973	4.126703	0.192339
80	6	0	-0.773493	1.998174	-1.728700
81	1	0	-0.446688	3.048055	-1.739016
82	1	0	0.109036	1.361440	-1.545515

83	1	0	-1.177554	1.753392	-2.723578
84	6	0	-2.805237	-0.482018	-1.620005
85	1	0	-2.351236	-0.200435	-2.579840
86	1	0	-2.250640	-1.349146	-1.220962
87	1	0	-3.844297	-0.796394	-1.801828
88	6	0	-4.638748	-0.129936	0.963826
89	1	0	-5.159671	-0.552983	0.092235
90	1	0	-4.208087	-0.958727	1.551957
91	1	0	-5.383316	0.385068	1.585112
92	1	0	-12.127624	3.557165	-1.129329
93	1	0	-9.079258	-1.223810	0.638536
94	6	0	-8.326063	3.930556	1.611778
95	1	0	-7.922892	4.905877	1.936547
96	1	0	-9.060001	3.606285	2.366498
97	6	0	-7.138998	2.908429	1.603561
98	1	0	-7.035745	2.504256	2.623480
99	1	0	-6.192065	3.416750	1.375688

Structure 7*

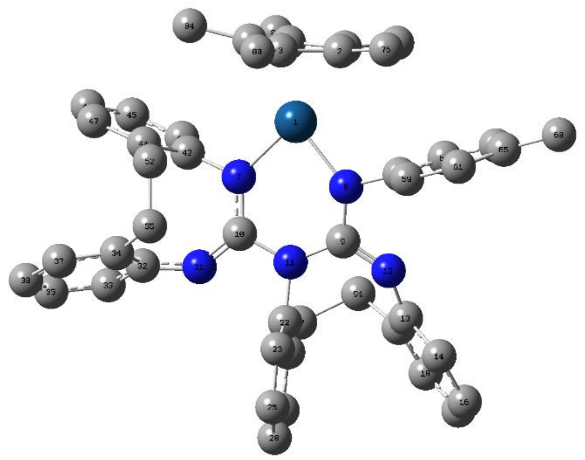

Zero-point correction= 0.791215 (Hartree/Particle)

Thermal correction to Energy= 0.839621

Thermal correction to Enthalpy= 0.840565

Thermal correction to Gibbs Free Energy= 0.711111

Sum of electronic and zero-point Energies= -2194.467193

Sum of electronic and thermal Energies= -2194.418787

Sum of electronic and thermal Enthalpies= -2194.417843

Sum of electronic and thermal Free Energies= -2194.547296

Center Number	Atomic Number	Atomic Type	Coordinates (Angstroms) X	Y	Z
1	77	0	-3.993923	3.031852	-0.131087
2	6	0	-2.468487	4.014987	1.153884
3	6	0	-2.262656	4.343958	-0.247957
4	6	0	-2.382055	2.590395	1.273655
5	6	0	-1.917332	3.133493	-0.963938
6	6	0	-2.061387	2.040152	-0.047397
7	7	0	-5.104409	2.366696	-1.668841
8	7	0	-5.701963	3.278650	0.856369
9	6	0	-6.981737	2.803235	0.602977
10	6	0	-6.459934	2.238981	-1.833034
11	7	0	-7.302976	2.444590	-0.714971
12	7	0	-7.832836	2.822239	1.588286
13	6	0	-9.006939	2.138162	1.843164
14	6	0	-10.033284	2.849154	2.517239
15	6	0	-9.186784	0.736142	1.640131
16	6	0	-11.190379	2.215555	2.969699
17	1	0	-9.886784	3.920217	2.679543
18	6	0	-10.345431	0.117415	2.132693
19	6	0	-11.350898	0.832853	2.792712
20	1	0	-11.963029	2.800096	3.476587

21	1	0	-10.461132	-0.959508	1.969966
22	6	0	-8.716297	2.432341	-1.043355
23	6	0	-9.346900	3.651160	-1.300711
24	6	0	-9.415645	1.216181	-1.073001
25	6	0	-10.710698	3.673456	-1.614240
26	1	0	-8.761053	4.572418	-1.255620
27	6	0	-10.779188	1.257344	-1.396539
28	6	0	-11.425700	2.470250	-1.669378
29	1	0	-11.209831	4.623911	-1.815975
30	1	0	-11.341946	0.319937	-1.417967
31	7	0	-7.108076	1.861340	-2.900766
32	6	0	-6.897236	2.048174	-4.248879
33	6	0	-7.230972	0.989420	-5.131359
34	6	0	-6.534437	3.307646	-4.820059
35	6	0	-7.204082	1.156723	-6.516843
36	1	0	-7.518317	0.030134	-4.692660
37	6	0	-6.536705	3.455019	-6.213028
38	6	0	-6.866615	2.399441	-7.073361
39	1	0	-7.460868	0.314767	-7.165821
40	1	0	-6.256028	4.428944	-6.628366
41	1	0	-6.860246	2.543699	-8.156207
42	6	0	-4.348563	1.932603	-2.810515
43	6	0	-3.965501	0.589075	-2.904637
44	6	0	-4.013572	2.852214	-3.819314
45	6	0	-3.243442	0.141914	-4.016151
46	1	0	-4.246527	-0.093321	-2.099315
47	6	0	-3.301826	2.383656	-4.934143
48	6	0	-2.913592	1.044004	-5.038173
49	1	0	-2.946515	-0.907108	-4.087732
50	1	0	-3.052815	3.090466	-5.730485

51	1	0	-2.357849	0.701998	-5.914359
52	6	0	-4.474968	4.283147	-3.725897
53	1	0	-4.212034	4.692420	-2.735430
54	1	0	-3.949785	4.871080	-4.495024
55	6	0	-6.020954	4.420514	-3.944169
56	1	0	-6.531338	4.407421	-2.968861
57	1	0	-6.227302	5.397512	-4.408839
58	6	0	-5.590778	3.846058	2.174764
59	6	0	-5.746861	5.227056	2.334595
60	6	0	-5.336208	3.045158	3.294509
61	6	0	-5.637582	5.802487	3.605634
62	1	0	-5.958109	5.840261	1.455293
63	6	0	-5.217374	3.627528	4.559152
64	1	0	-5.244113	1.965867	3.161389
65	6	0	-5.363061	5.017012	4.737324
66	1	0	-5.760560	6.883300	3.721968
67	1	0	-5.016957	2.994139	5.428556
68	6	0	-5.203388	5.642101	6.101496
69	1	0	-5.702457	6.621885	6.151314
70	1	0	-5.622427	4.995198	6.888356
71	1	0	-4.137182	5.799513	6.341905
72	6	0	-2.425392	1.785333	2.529325
73	1	0	-2.994241	0.854380	2.384656
74	1	0	-1.395419	1.509095	2.816357
75	1	0	-2.877359	2.352239	3.352648
76	6	0	-2.597587	5.003023	2.262657
77	1	0	-3.124830	4.578674	3.127332
78	1	0	-1.587619	5.314391	2.583550
79	1	0	-3.143859	5.898750	1.938121
80	6	0	-2.238184	5.726383	-0.809095

81	1	0	-2.969195	6.369485	-0.298201
82	1	0	-1.235541	6.166982	-0.665721
83	1	0	-2.458263	5.721560	-1.886118
84	6	0	-1.360837	3.057436	-2.343010
85	1	0	-1.739217	3.866760	-2.981028
86	1	0	-0.262375	3.157816	-2.282073
87	1	0	-1.594130	2.098975	-2.825399
88	6	0	-1.761384	0.604145	-0.320280
89	1	0	-1.875277	0.373043	-1.387798
90	1	0	-0.721403	0.380776	-0.022950
91	1	0	-2.427270	-0.053071	0.258616
92	1	0	-12.243375	0.322234	3.161786
93	1	0	-12.490520	2.476237	-1.914340
94	6	0	-8.233207	-0.061793	0.787666
95	1	0	-7.216253	0.354018	0.844759
96	1	0	-8.181599	-1.102086	1.146948
97	6	0	-8.726153	-0.071548	-0.697446
98	1	0	-9.442585	-0.897964	-0.826941
99	1	0	-7.875799	-0.249490	-1.377204

TS4*

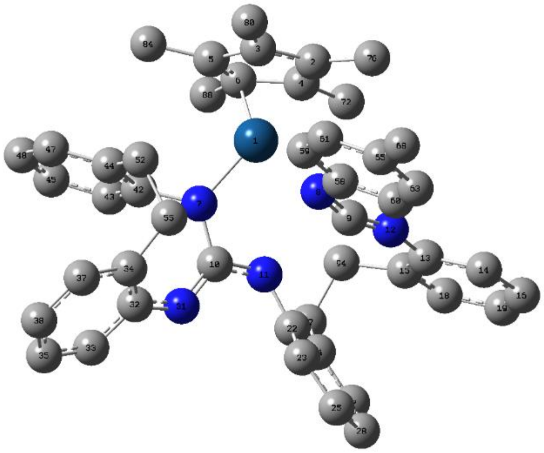

Zero-point correction= 0.787254 (Hartree/Particle)

Thermal correction to Energy= 0.836783

Thermal correction to Enthalpy= 0.837727

Thermal correction to Gibbs Free Energy= 0.703825

Sum of electronic and zero-point Energies= -2194.420804

Sum of electronic and thermal Energies= -2194.371274

Sum of electronic and thermal Enthalpies= -2194.370330

Sum of electronic and thermal Free Energies= -2194.504232

Center	Atomic	Atomic	Coordinates (Angstroms)		
Number	Number	Type	X	Y	Z
1	77	0	-4.222570	2.512536	-0.282518
2	6	0	-3.079535	3.248032	1.482540
3	6	0	-2.371734	3.543519	0.263205
4	6	0	-3.280473	1.821817	1.547102
5	6	0	-2.078999	2.276288	-0.422050
6	6	0	-2.640324	1.215870	0.376037
7	7	0	-5.054249	1.924634	-1.938936
8	7	0	-5.961089	3.639943	0.223087
9	6	0	-6.694966	2.873735	0.943375
10	6	0	-6.462032	1.723206	-2.093001
11	7	0	-7.049310	1.333352	-0.937838
12	7	0	-7.233422	2.433520	1.921417
13	6	0	-8.045226	1.652481	2.721641
14	6	0	-8.853644	2.309789	3.672082
15	6	0	-7.995802	0.239569	2.654758
16	6	0	-9.633580	1.563618	4.555586
17	1	0	-8.855613	3.401100	3.702598
18	6	0	-8.780775	-0.477721	3.569958
19	6	0	-9.592616	0.162640	4.512318

20	1	0	-10.264984	2.077136	5.283713
21	1	0	-8.755132	-1.570320	3.523934
22	6	0	-8.435171	1.336730	-0.822885
23	6	0	-9.257951	2.399787	-1.274806
24	6	0	-9.038348	0.274008	-0.093458
25	6	0	-10.624308	2.426288	-0.990692
26	1	0	-8.792309	3.207726	-1.842898
27	6	0	-10.414860	0.317586	0.175589
28	6	0	-11.214689	1.380888	-0.261508
29	1	0	-11.234187	3.265448	-1.337757
30	1	0	-10.861418	-0.504183	0.744586
31	7	0	-7.093999	1.752960	-3.252322
32	6	0	-6.731327	2.285392	-4.474599
33	6	0	-7.228359	1.588735	-5.615364
34	6	0	-6.047497	3.519674	-4.719472
35	6	0	-7.057611	2.056898	-6.915870
36	1	0	-7.765451	0.654112	-5.431206
37	6	0	-5.908959	3.981568	-6.038938
38	6	0	-6.397293	3.275228	-7.142739
39	1	0	-7.452779	1.477627	-7.755361
40	1	0	-5.386830	4.932705	-6.193267
41	1	0	-6.271737	3.667150	-8.154831
42	6	0	-4.205810	1.515374	-3.009434
43	6	0	-3.977438	0.148672	-3.229164
44	6	0	-3.558333	2.491313	-3.793867
45	6	0	-3.081092	-0.263716	-4.221082
46	1	0	-4.504842	-0.577248	-2.605402
47	6	0	-2.673756	2.057031	-4.792117
48	6	0	-2.423349	0.695586	-5.003586
49	1	0	-2.899169	-1.328775	-4.383847

50	1	0	-2.178139	2.806997	-5.415504
51	1	0	-1.724756	0.383404	-5.783422
52	6	0	-3.846552	3.958939	-3.586385
53	1	0	-3.417577	4.292589	-2.623497
54	1	0	-3.332855	4.524627	-4.380577
55	6	0	-5.365502	4.304325	-3.626931
56	1	0	-5.824319	4.103023	-2.650121
57	1	0	-5.464094	5.385460	-3.821171
58	6	0	-6.068185	5.055359	0.285872
59	6	0	-5.201965	5.829973	-0.501986
60	6	0	-7.005309	5.698411	1.114764
61	6	0	-5.261518	7.223906	-0.445482
62	1	0	-4.483488	5.328667	-1.151018
63	6	0	-7.055383	7.094035	1.153944
64	1	0	-7.688632	5.109484	1.730774
65	6	0	-6.187463	7.885444	0.380449
66	1	0	-4.574082	7.811479	-1.060837
67	1	0	-7.784563	7.581080	1.807623
68	6	0	-6.270997	9.391068	0.409751
69	1	0	-6.967101	9.762559	-0.362826
70	1	0	-6.638316	9.751197	1.383118
71	1	0	-5.289061	9.848917	0.213936
72	6	0	-3.976401	1.080730	2.641867
73	1	0	-4.389118	0.130490	2.274173
74	1	0	-3.268518	0.855850	3.458636
75	1	0	-4.802451	1.675725	3.058998
76	6	0	-3.592633	4.247553	2.470075
77	1	0	-4.521935	3.890571	2.938696
78	1	0	-2.851229	4.416804	3.269049
79	1	0	-3.804544	5.209546	1.981765

80	6	0	-1.885421	4.885798	-0.169003
81	1	0	-2.521530	5.688105	0.229642
82	1	0	-0.857366	5.040409	0.203630
83	1	0	-1.857434	4.966934	-1.266053
84	6	0	-1.219995	2.112990	-1.630632
85	1	0	-1.296288	2.987149	-2.291711
86	1	0	-0.165566	2.002937	-1.322339
87	1	0	-1.512881	1.225006	-2.208959
88	6	0	-2.544543	-0.245869	0.086584
89	1	0	-2.488458	-0.421773	-0.997642
90	1	0	-1.637205	-0.663958	0.555943
91	1	0	-3.416944	-0.783507	0.485890
92	1	0	-10.191480	-0.427123	5.209663
93	1	0	-12.284335	1.394868	-0.038311
94	6	0	-7.238227	-0.474119	1.569408
95	1	0	-6.449199	0.167229	1.151768
96	1	0	-6.773195	-1.387320	1.976885
97	6	0	-8.195681	-0.873691	0.410294
98	1	0	-8.861605	-1.679321	0.762462
99	1	0	-7.575834	-1.273128	-0.410049

Structure 8

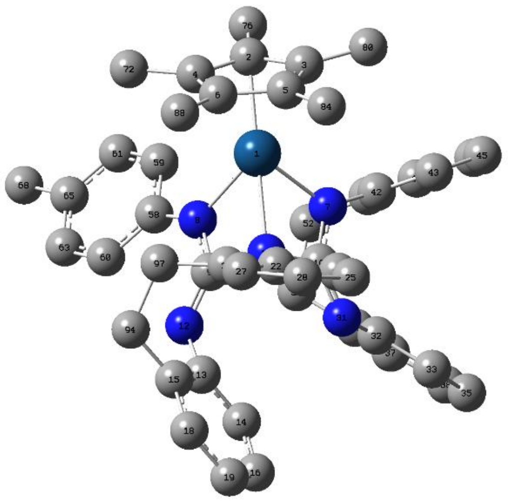

Zero-point correction= 0.791302 (Hartree/Particle)

Thermal correction to Energy= 0.840193

Thermal correction to Enthalpy= 0.841138

Thermal correction to Gibbs Free Energy= 0.708520

Sum of electronic and zero-point Energies= -2194.471735

Sum of electronic and thermal Energies= -2194.422844

Sum of electronic and thermal Enthalpies= -2194.421899

Sum of electronic and thermal Free Energies= -2194.554516

Center Number	Atomic Number	Atomic Type	Coordinates (Angstroms) X	Y	Z
1	77	0	-3.820968	2.556979	-0.668511
2	6	0	-1.758751	2.355165	-0.097419
3	6	0	-2.165809	1.145485	-0.803762
4	6	0	-2.565056	2.451027	1.116646
5	6	0	-3.254747	0.549925	-0.080431
6	6	0	-3.486814	1.351051	1.124490
7	7	0	-4.308404	2.812831	-2.682626

8	7	0	-4.826682	4.392854	-0.402627
9	6	0	-6.117497	4.076747	-0.660785
10	6	0	-5.642326	2.902392	-2.669934
11	7	0	-5.995541	2.682299	-1.224445
12	7	0	-7.185109	4.783825	-0.570742
13	6	0	-8.468322	4.284131	-0.842877
14	6	0	-9.081842	4.527837	-2.085898
15	6	0	-9.190006	3.630008	0.183929
16	6	0	-10.392763	4.103216	-2.323610
17	1	0	-8.503416	5.029789	-2.863519
18	6	0	-10.500011	3.203114	-0.080796
19	6	0	-11.108396	3.434155	-1.321109
20	1	0	-10.852788	4.290587	-3.297407
21	1	0	-11.048443	2.681683	0.709883
22	6	0	-6.851584	1.575872	-0.881361
23	6	0	-7.038775	0.567147	-1.843983
24	6	0	-7.357924	1.408102	0.428300
25	6	0	-7.729953	-0.603334	-1.524574
26	1	0	-6.640487	0.705498	-2.848983
27	6	0	-8.049585	0.219822	0.717908
28	6	0	-8.239723	-0.785637	-0.234241
29	1	0	-7.862596	-1.372283	-2.288830
30	1	0	-8.444034	0.094250	1.730485
31	7	0	-6.580851	3.157805	-3.515011
32	6	0	-6.351950	3.771688	-4.752918
33	6	0	-6.980662	3.213723	-5.889559
34	6	0	-5.644621	5.001268	-4.893369
35	6	0	-6.885823	3.818381	-7.144045
36	1	0	-7.542816	2.285335	-5.758772
37	6	0	-5.578557	5.596248	-6.163563

38	6	0	-6.180330	5.021836	-7.288605
39	1	0	-7.371781	3.355971	-8.007225
40	1	0	-5.034989	6.541665	-6.261520
41	1	0	-6.108815	5.511172	-8.262798
42	6	0	-3.528585	2.847007	-3.864199
43	6	0	-3.273623	1.647035	-4.545311
44	6	0	-3.001708	4.068045	-4.333669
45	6	0	-2.466655	1.640315	-5.687672
46	1	0	-3.715807	0.725414	-4.160531
47	6	0	-2.187535	4.039302	-5.476282
48	6	0	-1.909718	2.840783	-6.146637
49	1	0	-2.272160	0.702838	-6.213936
50	1	0	-1.783937	4.982984	-5.854730
51	1	0	-1.273297	2.847474	-7.034784
52	6	0	-3.394811	5.369908	-3.686489
53	1	0	-3.081654	5.382936	-2.630168
54	1	0	-2.859824	6.183727	-4.200716
55	6	0	-4.936540	5.659601	-3.732063
56	1	0	-5.394438	5.350089	-2.783598
57	1	0	-5.066807	6.751252	-3.805635
58	6	0	-4.349813	5.649890	-0.022090
59	6	0	-2.962265	5.885199	-0.143081
60	6	0	-5.159442	6.698993	0.468373
61	6	0	-2.401823	7.109276	0.214980
62	1	0	-2.343472	5.078361	-0.539513
63	6	0	-4.579233	7.923439	0.822329
64	1	0	-6.234050	6.546065	0.555837
65	6	0	-3.199733	8.158677	0.710674
66	1	0	-1.323469	7.258545	0.101349
67	1	0	-5.224859	8.723568	1.197868

68	6	0	-2.587950	9.476873	1.116913
69	1	0	-1.875034	9.838540	0.357752
70	1	0	-3.361761	10.246619	1.259157
71	1	0	-2.029889	9.383355	2.065200
72	6	0	-2.425416	3.479147	2.191610
73	1	0	-3.391658	3.666201	2.682268
74	1	0	-1.712092	3.125435	2.956134
75	1	0	-2.057586	4.431584	1.786623
76	6	0	-0.562443	3.182159	-0.448775
77	1	0	-0.558048	4.135782	0.097557
78	1	0	0.360117	2.637543	-0.181389
79	1	0	-0.529561	3.393185	-1.528530
80	6	0	-1.504289	0.621763	-2.033608
81	1	0	-1.266687	1.435396	-2.734165
82	1	0	-0.561630	0.119252	-1.755322
83	1	0	-2.142614	-0.103492	-2.555493
84	6	0	-4.002843	-0.696824	-0.426587
85	1	0	-3.883078	-0.947893	-1.490083
86	1	0	-3.632335	-1.544398	0.175453
87	1	0	-5.078056	-0.573417	-0.222449
88	6	0	-4.437668	0.990634	2.215524
89	1	0	-5.351448	0.533849	1.807811
90	1	0	-3.962441	0.255418	2.888519
91	1	0	-4.717943	1.870337	2.810614
92	1	0	-12.131678	3.096812	-1.502331
93	1	0	-8.776998	-1.699265	0.028831
94	6	0	-8.547848	3.375399	1.526374
95	1	0	-8.263816	4.335384	1.992301
96	1	0	-9.296758	2.900167	2.179703
97	6	0	-7.274795	2.467640	1.496982

98	1	0	-7.190655	1.973785	2.477182
99	1	0	-6.365866	3.073563	1.378043

Structure 8*

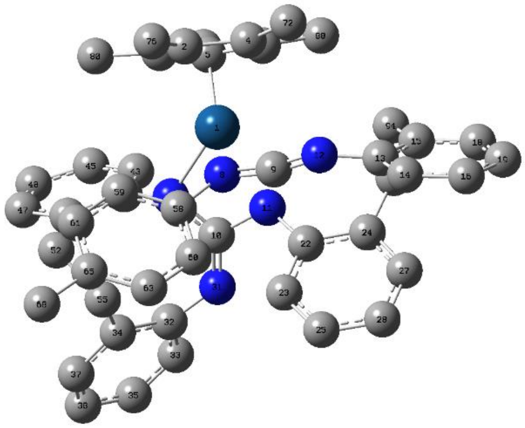

Zero-point correction= 0.790118 (Hartree/Particle)

Thermal correction to Energy= 0.838715

Thermal correction to Enthalpy= 0.839659

Thermal correction to Gibbs Free Energy= 0.710841

Sum of electronic and zero-point Energies= -2194.433057

Sum of electronic and thermal Energies= -2194.384460

Sum of electronic and thermal Enthalpies= -2194.383516

Sum of electronic and thermal Free Energies= -2194.512334

Center Number	Atomic Number	Atomic Type	Coordinates (Angstroms) X	Y	Z
1	77	0	-4.542848	2.492656	-0.435551
2	6	0	-2.986166	3.161053	0.941334
3	6	0	-2.386236	2.666547	-0.266552
4	6	0	-3.697382	2.068880	1.597444
5	6	0	-2.788479	1.270859	-0.422646
6	6	0	-3.577526	0.914535	0.753221
7	7	0	-4.877788	3.027850	-2.401463

8	7	0	-5.990828	4.073327	0.282069
9	6	0	-6.721088	3.584819	1.187831
10	6	0	-6.084174	2.402161	-2.604490
11	7	0	-6.428805	1.804659	-1.388648
12	7	0	-7.231581	3.159814	2.186057
13	6	0	-8.128580	2.319425	2.837763
14	6	0	-9.097984	2.901442	3.673996
15	6	0	-8.002548	0.915235	2.720250
16	6	0	-9.980070	2.084401	4.382861
17	1	0	-9.151096	3.989158	3.747851
18	6	0	-8.893252	0.125396	3.462747
19	6	0	-9.875570	0.690044	4.282960
20	1	0	-10.741673	2.538184	5.020369
21	1	0	-8.810525	-0.962056	3.378786
22	6	0	-7.774697	1.950338	-0.987638
23	6	0	-8.533422	3.080390	-1.392969
24	6	0	-8.416866	0.987781	-0.168802
25	6	0	-9.872473	3.237438	-1.041210
26	1	0	-8.044963	3.843987	-2.000108
27	6	0	-9.772629	1.157318	0.167720
28	6	0	-10.512186	2.261352	-0.261458
29	1	0	-10.416128	4.126253	-1.373645
30	1	0	-10.244474	0.396686	0.796501
31	7	0	-6.806404	2.278052	-3.687901
32	6	0	-6.669577	2.973527	-4.883359
33	6	0	-6.973242	2.230322	-6.058981
34	6	0	-6.388391	4.368051	-5.045814
35	6	0	-6.975998	2.801415	-7.329606
36	1	0	-7.209323	1.170411	-5.929537
37	6	0	-6.412915	4.921123	-6.338405

38	6	0	-6.696789	4.168489	-7.482000
39	1	0	-7.207274	2.182663	-8.201319
40	1	0	-6.196649	5.990975	-6.436703
41	1	0	-6.709141	4.638456	-8.468312
42	6	0	-3.937272	3.060970	-3.469761
43	6	0	-3.335722	1.861207	-3.882761
44	6	0	-3.643671	4.261784	-4.147030
45	6	0	-2.371376	1.846170	-4.893771
46	1	0	-3.645905	0.940923	-3.386058
47	6	0	-2.666395	4.233229	-5.157105
48	6	0	-2.013427	3.049009	-5.517841
49	1	0	-1.906362	0.903915	-5.194949
50	1	0	-2.440217	5.162541	-5.688826
51	1	0	-1.256223	3.059188	-6.305646
52	6	0	-4.443560	5.515778	-3.896670
53	1	0	-4.161907	5.975841	-2.939336
54	1	0	-4.189924	6.241712	-4.687319
55	6	0	-5.984937	5.285192	-3.912118
56	1	0	-6.307924	4.894897	-2.940143
57	1	0	-6.466591	6.268597	-4.045966
58	6	0	-6.182217	5.400734	-0.195049
59	6	0	-5.067067	6.099065	-0.665590
60	6	0	-7.443048	6.021211	-0.171372
61	6	0	-5.208522	7.423272	-1.088861
62	1	0	-4.106915	5.583136	-0.704987
63	6	0	-7.569050	7.341298	-0.608456
64	1	0	-8.316942	5.456224	0.161945
65	6	0	-6.456138	8.068735	-1.069607
66	1	0	-4.332850	7.963152	-1.459927
67	1	0	-8.554688	7.815043	-0.601035

68	6	0	-6.598833	9.501846	-1.517272
69	1	0	-5.777973	9.792329	-2.190276
70	1	0	-7.554639	9.662305	-2.040040
71	1	0	-6.579629	10.188062	-0.652482
72	6	0	-4.251360	2.140204	2.983904
73	1	0	-4.939676	1.312259	3.199378
74	1	0	-3.421232	2.088194	3.710580
75	1	0	-4.784820	3.085898	3.157042
76	6	0	-2.831335	4.528835	1.524037
77	1	0	-3.792999	4.911294	1.897127
78	1	0	-2.127848	4.488884	2.373492
79	1	0	-2.434582	5.238439	0.785165
80	6	0	-1.491573	3.421795	-1.194608
81	1	0	-1.703963	4.500452	-1.162838
82	1	0	-0.437865	3.269691	-0.903061
83	1	0	-1.619409	3.075432	-2.230440
84	6	0	-2.178956	0.267652	-1.347070
85	1	0	-1.726174	0.749195	-2.222886
86	1	0	-1.385696	-0.279037	-0.805665
87	1	0	-2.920211	-0.469604	-1.689745
88	6	0	-3.994345	-0.489280	1.052964
89	1	0	-4.727909	-0.860107	0.321091
90	1	0	-3.108192	-1.143559	0.996433
91	1	0	-4.424027	-0.575493	2.058001
92	1	0	-10.554609	0.045843	4.845523
93	1	0	-11.563800	2.365779	0.015563
94	6	0	-7.012766	0.275287	1.782710
95	1	0	-6.198404	0.973140	1.544814
96	1	0	-6.571167	-0.607741	2.270648
97	6	0	-7.657195	-0.157277	0.438804

98	1	0	-8.338763	-1.004230	0.621028
99	1	0	-6.854754	-0.487630	-0.240852

Structure 9

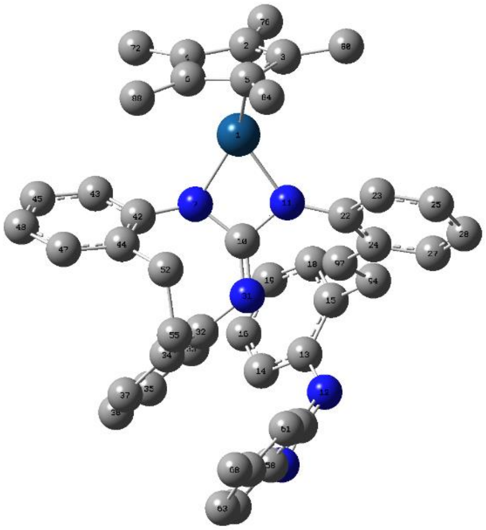

Zero-point correction= 0.787437 (Hartree/Particle)

Thermal correction to Energy= 0.838452

Thermal correction to Enthalpy= 0.839396

Thermal correction to Gibbs Free Energy= 0.699423

Sum of electronic and zero-point Energies= -2194.455580

Sum of electronic and thermal Energies= -2194.404566

Sum of electronic and thermal Enthalpies= -2194.403621

Sum of electronic and thermal Free Energies= -2194.543595

Center Number	Atomic Number	Atomic Type	Coordinates (Angstroms) X	Y	Z
1	77	0	-5.184609	2.896389	-0.174450
2	6	0	-5.128178	3.218088	1.960176
3	6	0	-4.273207	4.209583	1.344720
4	6	0	-4.601718	1.886990	1.669967
5	6	0	-3.271553	3.493254	0.591677
6	6	0	-3.463927	2.052357	0.810011

7	7	0	-6.056988	1.639200	-1.501556
8	7	0	-9.599410	2.771281	-7.012552
9	6	0	-10.102032	3.178073	-5.966765
10	6	0	-6.381102	2.653358	-2.417161
11	7	0	-6.136484	3.807006	-1.666037
12	7	0	-10.700683	3.708382	-5.035213
13	6	0	-11.330853	3.189201	-3.889509
14	6	0	-12.102785	2.014943	-3.959216
15	6	0	-11.210836	3.905422	-2.675432
16	6	0	-12.765859	1.545959	-2.821672
17	1	0	-12.178605	1.485550	-4.912271
18	6	0	-11.885521	3.410957	-1.549573
19	6	0	-12.663055	2.247621	-1.612914
20	1	0	-13.364179	0.633986	-2.882339
21	1	0	-11.787910	3.953599	-0.604883
22	6	0	-6.410008	5.130664	-2.044091
23	6	0	-5.371753	6.073123	-1.933955
24	6	0	-7.710326	5.550804	-2.440666
25	6	0	-5.586003	7.420441	-2.235412
26	1	0	-4.392905	5.710542	-1.610035
27	6	0	-7.898619	6.908353	-2.745282
28	6	0	-6.855761	7.840783	-2.651172
29	1	0	-4.764532	8.136312	-2.150534
30	1	0	-8.892844	7.249787	-3.043939
31	7	0	-6.753203	2.636749	-3.647024
32	6	0	-7.063236	1.442700	-4.316059
33	6	0	-8.395098	0.983443	-4.317937
34	6	0	-6.082857	0.763742	-5.078336
35	6	0	-8.763784	-0.130856	-5.077635
36	1	0	-9.137105	1.519596	-3.719403

37	6	0	-6.477304	-0.348994	-5.833038
38	6	0	-7.804294	-0.798387	-5.848668
39	1	0	-9.803395	-0.469652	-5.068905
40	1	0	-5.717073	-0.873985	-6.419786
41	1	0	-8.083616	-1.664601	-6.453213
42	6	0	-5.574298	0.343454	-1.793476
43	6	0	-6.061062	-0.710859	-0.995895
44	6	0	-4.540015	0.089397	-2.728867
45	6	0	-5.554416	-2.006563	-1.123030
46	1	0	-6.843917	-0.482102	-0.269138
47	6	0	-4.053692	-1.222275	-2.849278
48	6	0	-4.543911	-2.267961	-2.059190
49	1	0	-5.947686	-2.809408	-0.494477
50	1	0	-3.257080	-1.415494	-3.574637
51	1	0	-4.137501	-3.276252	-2.168281
52	6	0	-3.988897	1.179645	-3.609030
53	1	0	-4.122990	2.154583	-3.116640
54	1	0	-2.903971	1.029788	-3.734616
55	6	0	-4.638653	1.197484	-5.033310
56	1	0	-4.544967	2.217795	-5.441660
57	1	0	-4.062105	0.528618	-5.692776
58	6	0	-8.240505	2.724332	-7.384294
59	6	0	-7.279463	3.554419	-6.777292
60	6	0	-7.853162	1.855720	-8.414504
61	6	0	-5.953357	3.505742	-7.201634
62	1	0	-7.576101	4.219410	-5.962950
63	6	0	-6.513971	1.802220	-8.812540
64	1	0	-8.604712	1.217492	-8.883862
65	6	0	-5.541485	2.619885	-8.214746
66	1	0	-5.215758	4.156741	-6.723235

67	1	0	-6.215969	1.109596	-9.604781
68	6	0	-4.089448	2.527535	-8.609498
69	1	0	-3.515130	1.977496	-7.842665
70	1	0	-3.966916	1.997155	-9.565979
71	1	0	-3.633292	3.526341	-8.701454
72	6	0	-5.148899	0.591156	2.174852
73	1	0	-4.977992	-0.213499	1.444309
74	1	0	-4.661546	0.309009	3.124190
75	1	0	-6.231458	0.666262	2.355621
76	6	0	-6.310447	3.506209	2.825505
77	1	0	-7.056623	2.702117	2.751111
78	1	0	-5.992489	3.581802	3.880477
79	1	0	-6.784538	4.456459	2.541284
80	6	0	-4.405838	5.692485	1.481568
81	1	0	-5.462893	5.986229	1.562358
82	1	0	-3.882154	6.044781	2.387116
83	1	0	-3.977405	6.212408	0.613498
84	6	0	-2.161444	4.097572	-0.204254
85	1	0	-2.422551	5.109493	-0.545712
86	1	0	-1.250020	4.168620	0.414909
87	1	0	-1.929299	3.478779	-1.083510
88	6	0	-2.631782	0.955142	0.227266
89	1	0	-2.183802	1.272039	-0.726037
90	1	0	-1.817187	0.674776	0.917255
91	1	0	-3.249267	0.065433	0.030834
92	1	0	-13.182865	1.887931	-0.722143
93	1	0	-7.040256	8.890682	-2.890661
94	6	0	-10.272286	5.082225	-2.577582
95	1	0	-10.376915	5.713029	-3.476385
96	1	0	-10.520904	5.696524	-1.696749

97	6	0	-8.833529	4.545195	-2.484930
98	1	0	-8.658077	3.875537	-3.341908
99	1	0	-8.748267	3.890658	-1.597493

Structure 1'

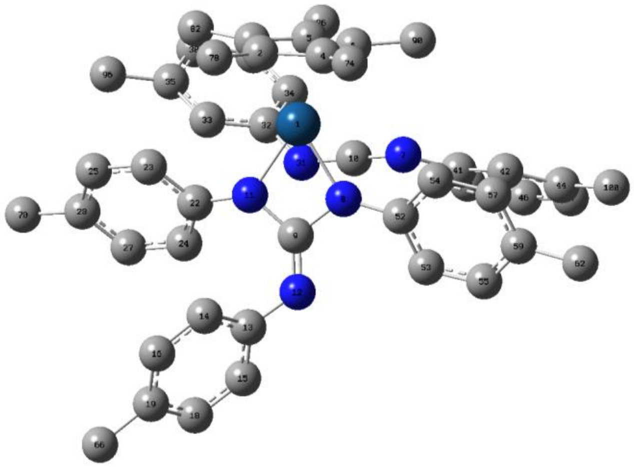

Zero-point correction= 0.823830 (Hartree/Particle)
Thermal correction to Energy= 0.879814
Thermal correction to Enthalpy= 0.880758
Thermal correction to Gibbs Free Energy= 0.727828
Sum of electronic and zero-point Energies= -2196.818886
Sum of electronic and thermal Energies= -2196.762902
Sum of electronic and thermal Enthalpies= -2196.761958
Sum of electronic and thermal Free Energies= -2196.914887

Center Number	Atomic Number	Atomic Type	Coordinates (Angstroms) X	Y	Z
1	77	0	-0.108026	-0.609362	-1.465767
2	6	0	0.226374	-0.753344	-3.603579
3	6	0	0.125539	0.638401	-3.264438
4	6	0	-1.045906	-1.401380	-3.259668

5	6	0	-1.180478	0.841899	-2.664948
6	6	0	-1.918771	-0.406814	-2.699589
7	7	0	-2.413723	0.965941	1.093765
8	7	0	-0.445188	-1.405891	0.348743
9	6	0	0.845722	-1.219362	0.856841
10	6	0	-1.404402	1.660802	1.056567
11	7	0	1.458664	-0.531385	-0.198533
12	7	0	1.281465	-1.684045	1.983686
13	6	0	2.639507	-1.819200	2.278210
14	6	0	3.583427	-2.298125	1.340086
15	6	0	3.088307	-1.544201	3.587069
16	6	0	4.922152	-2.462009	1.697472
17	1	0	3.249422	-2.522576	0.324358
18	6	0	4.435154	-1.696915	3.927546
19	6	0	5.379706	-2.157796	2.993326
20	1	0	5.635271	-2.826600	0.951340
21	1	0	4.763747	-1.455477	4.943208
22	6	0	2.611099	0.246458	-0.139906
23	6	0	3.333114	0.470978	-1.327494
24	6	0	3.057052	0.876913	1.042908
25	6	0	4.458057	1.297981	-1.337159
26	1	0	2.986385	-0.020944	-2.238059
27	6	0	4.192565	1.688596	1.022255
28	6	0	4.913875	1.924313	-0.162862
29	1	0	4.999810	1.457997	-2.274568
30	1	0	4.519280	2.167760	1.950220
31	7	0	-0.294384	2.174671	0.920165
32	6	0	0.027017	3.411011	0.324387
33	6	0	1.272893	3.536122	-0.311859
34	6	0	-0.866308	4.499117	0.339790

35	6	0	1.628938	4.724614	-0.967543
36	1	0	1.959052	2.688269	-0.303195
37	6	0	-0.506572	5.689185	-0.299428
38	6	0	0.727266	5.803783	-0.953435
39	1	0	-1.197731	6.535665	-0.287665
40	1	0	0.996914	6.738668	-1.452187
41	6	0	-3.505553	0.927767	1.975436
42	6	0	-4.373069	-0.172944	1.882009
43	6	0	-3.757115	1.951427	2.908448
44	6	0	-5.497688	-0.268979	2.713111
45	1	0	-4.141271	-0.968182	1.169035
46	6	0	-4.880050	1.857971	3.735663
47	6	0	-5.745964	0.761007	3.639146
48	1	0	-5.084128	2.651837	4.458475
49	1	0	-6.624594	0.701166	4.287417
50	1	0	2.364706	-1.184326	4.323221
51	1	0	2.498025	0.740468	1.968246
52	6	0	-1.469165	-2.151247	0.930329
53	6	0	-1.644198	-2.279840	2.326862
54	6	0	-2.407049	-2.778897	0.086095
55	6	0	-2.704415	-3.035756	2.834374
56	1	0	-0.942099	-1.787103	2.997566
57	6	0	-3.467015	-3.522323	0.607734
58	1	0	-2.267787	-2.674836	-0.989680
59	6	0	-3.631473	-3.679702	1.996067
60	1	0	-2.822507	-3.121195	3.919203
61	1	0	-4.175336	-4.000786	-0.076159
62	6	0	-4.735183	-4.539239	2.560495
63	1	0	-5.652146	-4.466060	1.954575
64	1	0	-4.438961	-5.603333	2.580304

65	1	0	-4.979442	-4.246184	3.593413
66	6	0	6.826766	-2.354111	3.374324
67	1	0	7.497282	-2.109471	2.535000
68	1	0	7.101001	-1.723543	4.234353
69	1	0	7.027627	-3.403091	3.656463
70	6	0	6.108522	2.846007	-0.180585
71	1	0	5.801387	3.894591	-0.348153
72	1	0	6.651631	2.814815	0.776961
73	1	0	6.808120	2.577255	-0.987525
74	6	0	-1.368818	-2.828505	-3.563503
75	1	0	-2.283704	-3.156899	-3.050958
76	1	0	-1.525099	-2.950740	-4.649607
77	1	0	-0.542777	-3.491687	-3.265055
78	6	0	1.368238	-1.433955	-4.286136
79	1	0	1.515219	-2.448377	-3.886258
80	1	0	1.165818	-1.520532	-5.368204
81	1	0	2.303182	-0.869641	-4.159536
82	6	0	1.118473	1.723481	-3.533194
83	1	0	2.109838	1.314779	-3.768635
84	1	0	0.783649	2.328680	-4.393658
85	1	0	1.218882	2.391487	-2.664443
86	6	0	-1.744472	2.153979	-2.239985
87	1	0	-0.952487	2.860171	-1.958038
88	1	0	-2.310124	2.590394	-3.083133
89	1	0	-2.437007	2.030207	-1.395461
90	6	0	-3.347874	-0.547921	-2.277991
91	1	0	-3.497096	-0.129479	-1.270057
92	1	0	-4.000507	-0.000959	-2.979979
93	1	0	-3.665769	-1.598988	-2.265050
94	1	0	-1.829655	4.400205	0.846254

95	1	0	-3.079620	2.806766	2.970930
96	6	0	2.945756	4.815928	-1.698428
97	1	0	3.650326	4.052436	-1.335844
98	1	0	2.800718	4.646541	-2.780298
99	1	0	3.399822	5.812558	-1.582119
100	6	0	-6.407515	-1.469193	2.630679
101	1	0	-6.234182	-2.035421	1.704022
102	1	0	-6.223464	-2.156060	3.474091
103	1	0	-7.466935	-1.169973	2.675569

Structure 2*’

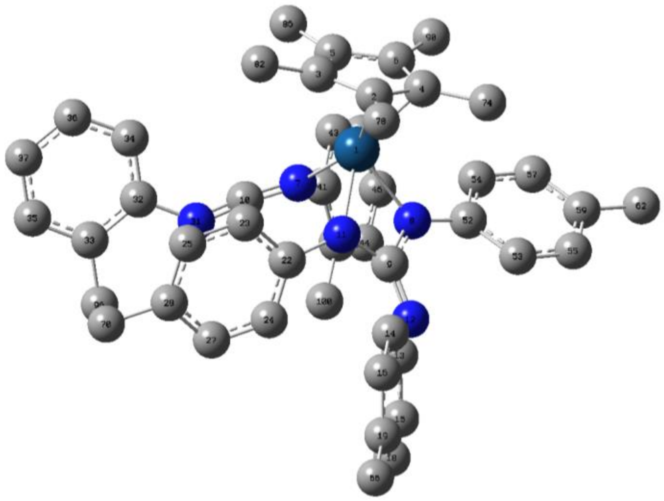

Zero-point correction= 0.824589 (Hartree/Particle)

Thermal correction to Energy= 0.879843

Thermal correction to Enthalpy= 0.880787

Thermal correction to Gibbs Free Energy= 0.732309

Sum of electronic and zero-point Energies= -2196.810547

Sum of electronic and thermal Energies= -2196.755293

Sum of electronic and thermal Enthalpies= -2196.754349

Sum of electronic and thermal Free Energies= -2196.902827

Center Atomic Atomic Coordinates (Angstroms)

Number	Number	Type	X	Y	Z
1	77	0	-0.391168	-0.929554	-0.977568
2	6	0	-0.292154	-0.995027	-3.117657
3	6	0	-1.649215	-0.608536	-2.760783
4	6	0	-0.037913	-2.312478	-2.561341
5	6	0	-2.212759	-1.688422	-1.985340
6	6	0	-1.246785	-2.753525	-1.869457
7	7	0	-1.337355	-0.348636	0.923385
8	7	0	1.258442	-1.323705	0.239210
9	6	0	1.856012	-0.085350	0.147384
10	6	0	-1.798932	0.804846	1.099006
11	7	0	0.937225	0.655123	-0.590365
12	7	0	3.008148	0.237264	0.689556
13	6	0	3.761179	1.325908	0.274083
14	6	0	3.765404	1.837180	-1.050851
15	6	0	4.638745	1.938407	1.201580
16	6	0	4.599138	2.897003	-1.411006
17	1	0	3.092176	1.393122	-1.787907
18	6	0	5.459815	3.006651	0.829648
19	6	0	5.462692	3.510032	-0.483269
20	1	0	4.575989	3.267478	-2.441584
21	1	0	6.117063	3.463797	1.576856
22	6	0	0.623241	1.997775	-0.406500
23	6	0	-0.303603	2.583491	-1.298677
24	6	0	1.116028	2.802386	0.646667
25	6	0	-0.741128	3.895106	-1.135756
26	1	0	-0.698042	1.959163	-2.099258
27	6	0	0.676918	4.122480	0.788614
28	6	0	-0.263761	4.697579	-0.082391

29	1	0	-1.479703	4.304918	-1.832228
30	1	0	1.067221	4.722000	1.617818
31	7	0	-2.117741	1.955590	1.296586
32	6	0	-3.195729	2.781598	0.953395
33	6	0	-3.333694	4.007549	1.640978
34	6	0	-4.081803	2.415872	-0.075552
35	6	0	-4.383662	4.853709	1.250830
36	6	0	-5.113798	3.278964	-0.445478
37	6	0	-5.263874	4.504413	0.219221
38	1	0	-4.504266	5.807711	1.771030
39	1	0	-5.795554	2.996995	-1.250652
40	1	0	-6.066822	5.187663	-0.065892
41	6	0	-1.393842	-1.329923	1.986940
42	6	0	-0.549979	-1.199032	3.105932
43	6	0	-2.251371	-2.421806	1.828464
44	6	0	-0.584831	-2.239148	4.051540
45	6	0	-2.269631	-3.438313	2.787720
46	6	0	-1.422276	-3.349646	3.899380
47	1	0	0.069184	-2.165976	4.924789
48	1	0	-2.937877	-4.293100	2.662643
49	1	0	-1.417194	-4.141642	4.651574
50	1	0	4.651219	1.560483	2.227941
51	1	0	1.819255	2.386165	1.366591
52	6	0	1.857514	-2.531887	0.582360
53	6	0	3.254356	-2.760835	0.577722
54	6	0	1.013258	-3.614778	0.914710
55	6	0	3.761226	-4.026456	0.889911
56	1	0	3.924121	-1.938385	0.328617
57	6	0	1.536516	-4.871641	1.218581
58	1	0	-0.062793	-3.438845	0.928394

59	6	0	2.924016	-5.108238	1.215813
60	1	0	4.845076	-4.181823	0.869881
61	1	0	0.852302	-5.689309	1.468446
62	6	0	3.490797	-6.459173	1.579199
63	1	0	3.623569	-6.559647	2.671626
64	1	0	2.822317	-7.273716	1.257108
65	1	0	4.477225	-6.615523	1.114548
66	6	0	6.374646	4.642286	-0.890791
67	1	0	5.893511	5.295804	-1.636584
68	1	0	6.656534	5.258539	-0.022306
69	1	0	7.309929	4.266630	-1.344183
70	6	0	-0.782559	6.097831	0.125335
71	1	0	-1.823160	6.078841	0.497757
72	1	0	-0.172333	6.643937	0.861285
73	1	0	-0.786225	6.672313	-0.815901
74	6	0	1.202374	-3.129001	-2.731229
75	1	0	1.423911	-3.689448	-1.810341
76	1	0	1.069979	-3.847977	-3.557876
77	1	0	2.065804	-2.489115	-2.962359
78	6	0	0.652835	-0.195698	-3.955826
79	1	0	1.695574	-0.437610	-3.705992
80	1	0	0.491008	-0.415662	-5.025006
81	1	0	0.503325	0.881674	-3.795113
82	6	0	-2.417961	0.562194	-3.290322
83	1	0	-1.755285	1.280236	-3.794417
84	1	0	-3.157904	0.215490	-4.033285
85	1	0	-2.961402	1.096247	-2.497457
86	6	0	-3.618815	-1.703408	-1.473497
87	1	0	-3.806934	-0.869311	-0.779471
88	1	0	-4.320034	-1.597954	-2.318225

89	1	0	-3.848470	-2.646822	-0.958995
90	6	0	-1.481418	-4.118968	-1.310107
91	1	0	-2.152541	-4.091664	-0.439304
92	1	0	-1.940193	-4.768344	-2.076417
93	1	0	-0.534133	-4.578134	-0.993807
94	1	0	-2.889698	-2.464036	0.946631
95	1	0	-3.940259	1.458296	-0.582309
96	6	0	-2.389252	4.377148	2.750219
97	1	0	-2.486238	3.680178	3.599490
98	1	0	-1.346382	4.315754	2.398717
99	1	0	-2.591065	5.396218	3.110094
100	6	0	0.364303	-0.016672	3.281642
101	1	0	1.159983	-0.019655	2.519527
102	1	0	-0.175301	0.939277	3.179670
103	1	0	0.832721	-0.042824	4.276345

Structure 2'

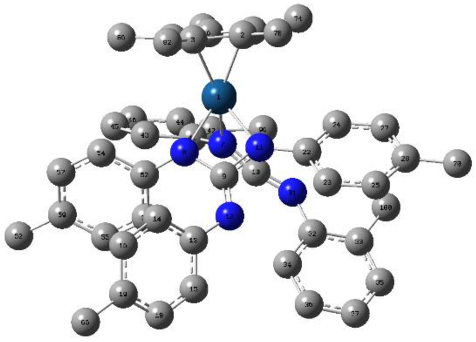

Zero-point correction= 0.823543 (Hartree/Particle)

Thermal correction to Energy= 0.879222

Thermal correction to Enthalpy= 0.880166

Thermal correction to Gibbs Free Energy= 0.728663

Sum of electronic and zero-point Energies= -2196.814904

Sum of electronic and thermal Energies= -2196.759226

Sum of electronic and thermal Enthalpies= -2196.758282

Sum of electronic and thermal Free Energies= -2196.909785

Center Number	Atomic Number	Atomic Type	Coordinates (Angstroms) X	Y	Z
1	77	0	2.137602	2.475585	-1.716623
2	6	0	2.661892	2.166736	0.334927
3	6	0	3.145065	3.461119	-0.115446
4	6	0	3.327161	1.133373	-0.444072
5	6	0	4.122401	3.227185	-1.177784
6	6	0	4.214626	1.804026	-1.372206
7	7	0	1.955700	1.653711	-3.751217
8	7	0	0.938220	4.081016	-2.437734
9	6	0	-0.285798	3.437042	-2.271456
10	6	0	0.921959	0.965828	-3.978872
11	7	0	0.060271	2.237475	-1.682551
12	7	0	-1.503148	3.803080	-2.595594
13	6	0	-1.823440	5.095397	-2.993051
14	6	0	-1.256864	6.269939	-2.431459
15	6	0	-2.841062	5.272145	-3.960187
16	6	0	-1.678301	7.536869	-2.836566
17	1	0	-0.471899	6.166036	-1.678859
18	6	0	-3.247282	6.547596	-4.364873
19	6	0	-2.673722	7.708468	-3.818456
20	1	0	-1.221074	8.422165	-2.381500
21	1	0	-4.032091	6.646951	-5.122114
22	6	0	-0.764496	1.241262	-1.181496
23	6	0	-2.151742	1.396403	-0.939715
24	6	0	-0.189261	-0.021233	-0.908544

25	6	0	-2.908393	0.318971	-0.469063
26	1	0	-2.616228	2.358222	-1.151972
27	6	0	-0.960335	-1.083505	-0.436819
28	6	0	-2.342481	-0.942873	-0.214528
29	1	0	-3.980158	0.463772	-0.296313
30	1	0	-0.480855	-2.049359	-0.245479
31	7	0	0.013723	0.173822	-4.042592
32	6	0	-1.353117	0.125527	-4.332696
33	6	0	-1.981759	-1.139522	-4.277511
34	6	0	-2.076711	1.295431	-4.618499
35	6	0	-3.359475	-1.189911	-4.535984
36	6	0	-3.446804	1.213001	-4.869009
37	6	0	-4.089659	-0.031825	-4.832518
38	1	0	-3.864247	-2.158781	-4.495537
39	1	0	-4.013085	2.123155	-5.079954
40	1	0	-5.162469	-0.100687	-5.026646
41	6	0	3.056259	1.711768	-4.677060
42	6	0	3.599187	0.528899	-5.225137
43	6	0	3.638242	2.961800	-4.922925
44	6	0	4.722623	0.659229	-6.060557
45	6	0	4.756569	3.059822	-5.754046
46	6	0	5.300093	1.903850	-6.330540
47	1	0	5.160743	-0.248821	-6.483916
48	1	0	5.202493	4.038908	-5.943690
49	1	0	6.178495	1.968831	-6.976562
50	1	0	-3.304482	4.379850	-4.391431
51	1	0	0.875188	-0.147944	-1.112723
52	6	0	1.221151	4.876301	-3.555144
53	6	0	0.634882	4.662482	-4.822429
54	6	0	2.185420	5.895408	-3.439684

55	6	0	1.022172	5.424661	-5.924236
56	1	0	-0.121760	3.883221	-4.929968
57	6	0	2.580036	6.643805	-4.552915
58	1	0	2.626030	6.083469	-2.458963
59	6	0	2.009156	6.423781	-5.817953
60	1	0	0.559546	5.232313	-6.897407
61	1	0	3.341561	7.421404	-4.438338
62	6	0	2.419592	7.239316	-7.019528
63	1	0	2.644294	6.592513	-7.883825
64	1	0	1.613070	7.925730	-7.331319
65	1	0	3.311651	7.846203	-6.801516
66	6	0	-3.089818	9.085773	-4.276687
67	1	0	-2.336198	9.532090	-4.950347
68	1	0	-4.044039	9.050169	-4.825385
69	1	0	-3.208160	9.775627	-3.424664
70	6	0	-3.188981	-2.108975	0.234419
71	1	0	-3.585560	-2.672095	-0.630282
72	1	0	-2.605846	-2.816957	0.844825
73	1	0	-4.054300	-1.771391	0.826951
74	6	0	3.278681	-0.340701	-0.196228
75	1	0	3.321880	-0.908874	-1.138003
76	1	0	4.140662	-0.648720	0.421808
77	1	0	2.362412	-0.624527	0.340703
78	6	0	1.685826	1.924266	1.441238
79	1	0	1.044762	1.060960	1.209063
80	1	0	2.225257	1.720602	2.382198
81	1	0	1.040876	2.801020	1.594903
82	6	0	2.789304	4.791141	0.464656
83	1	0	1.795363	4.762707	0.933201
84	1	0	3.527720	5.081589	1.231736

85	1	0	2.777913	5.565899	-0.315091
86	6	0	4.971907	4.255323	-1.852247
87	1	0	4.483302	5.238836	-1.853176
88	1	0	5.937222	4.355386	-1.325635
89	1	0	5.177653	3.972730	-2.895283
90	6	0	5.137582	1.112881	-2.320714
91	1	0	5.369678	1.742564	-3.190556
92	1	0	6.081450	0.876978	-1.798640
93	1	0	4.703992	0.168773	-2.679793
94	1	0	-1.573480	2.262755	-4.581987
95	1	0	3.209182	3.845602	-4.452863
96	6	0	3.055494	-0.835168	-4.886880
97	1	0	2.857538	-0.925345	-3.805434
98	1	0	2.101233	-1.042223	-5.399825
99	1	0	3.774474	-1.614288	-5.178021
100	6	0	-1.199236	-2.373077	-3.918107
101	1	0	-1.839879	-3.265785	-3.960561
102	1	0	-0.343437	-2.517675	-4.597602
103	1	0	-0.792065	-2.275475	-2.897480

TS1’

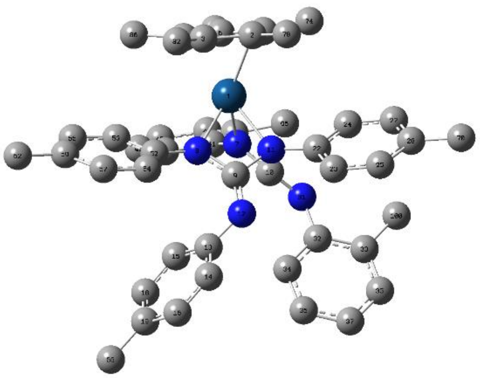

Zero-point correction= 0.824193 (Hartree/Particle)

Thermal correction to Energy= 0.878596

Thermal correction to Enthalpy= 0.879540

Thermal correction to Gibbs Free Energy= 0.731991

Sum of electronic and zero-point Energies= -2196.812367

Sum of electronic and thermal Energies= -2196.757965

Sum of electronic and thermal Enthalpies= -2196.757021

Sum of electronic and thermal Free Energies= -2196.904570

Center	Atomic	Atomic	Coordinates (Angstroms)		
Number	Number	Type	X	Y	Z
1	77	0	2.109364	2.147712	-1.841575
2	6	0	2.965588	2.087162	0.128399
3	6	0	3.580254	3.152767	-0.631706
4	6	0	3.256581	0.818450	-0.535964
5	6	0	4.253828	2.548443	-1.780659
6	6	0	4.043583	1.122188	-1.709676
7	7	0	1.235979	1.100901	-3.470684
8	7	0	1.018545	3.767974	-2.600633
9	6	0	-0.230770	3.422466	-2.150264
10	6	0	0.015147	0.784189	-3.251136
11	7	0	-0.002303	2.167340	-1.537120
12	7	0	-1.408755	3.977658	-2.250704
13	6	0	-1.738192	4.875524	-3.257479
14	6	0	-2.734123	5.848325	-3.010217
15	6	0	-1.208132	4.809395	-4.571930
16	6	0	-3.155776	6.723688	-4.014800
17	1	0	-3.169561	5.902107	-2.008558
18	6	0	-1.648677	5.680190	-5.570242
19	6	0	-2.622925	6.663207	-5.314981
20	1	0	-3.924535	7.469770	-3.788706

21	1	0	-1.223498	5.598722	-6.576051
22	6	0	-0.763090	1.578948	-0.522181
23	6	0	-1.807076	2.225043	0.173493
24	6	0	-0.438794	0.253641	-0.159966
25	6	0	-2.489000	1.551054	1.192420
26	1	0	-2.076783	3.243254	-0.104657
27	6	0	-1.124587	-0.400903	0.862720
28	6	0	-2.171786	0.231737	1.558783
29	1	0	-3.293662	2.069842	1.723248
30	1	0	-0.845894	-1.426474	1.124976
31	7	0	-0.988957	0.078312	-3.316457
32	6	0	-2.353779	0.389065	-3.393034
33	6	0	-3.275962	-0.557987	-2.889522
34	6	0	-2.794284	1.596472	-3.964729
35	6	0	-4.641740	-0.246462	-2.968496
36	6	0	-4.159182	1.879885	-4.032169
37	6	0	-5.087877	0.956553	-3.531604
38	1	0	-5.365048	-0.966095	-2.574820
39	1	0	-4.492146	2.825489	-4.466627
40	1	0	-6.157880	1.172109	-3.578734
41	6	0	1.924520	0.684427	-4.655706
42	6	0	2.153158	-0.685522	-4.899115
43	6	0	2.441968	1.676495	-5.498093
44	6	0	2.908853	-1.021993	-6.035893
45	6	0	3.191061	1.315425	-6.620671
46	6	0	3.424758	-0.041119	-6.889837
47	1	0	3.104695	-2.078833	-6.237649
48	1	0	3.590006	2.088208	-7.281469
49	1	0	4.014898	-0.334292	-7.761049
50	1	0	-0.438527	4.065576	-4.794476

51	1	0	0.368900	-0.243570	-0.699947
52	6	0	1.450153	5.040835	-2.963830
53	6	0	2.581442	5.160076	-3.795320
54	6	0	0.855685	6.227659	-2.477844
55	6	0	3.087535	6.413200	-4.147907
56	1	0	3.067542	4.245953	-4.138295
57	6	0	1.367930	7.473916	-2.843301
58	1	0	-0.004190	6.161786	-1.810300
59	6	0	2.486169	7.598440	-3.688763
60	1	0	3.969817	6.473854	-4.792775
61	1	0	0.891572	8.378980	-2.453433
62	6	0	3.004848	8.953415	-4.103227
63	1	0	4.074851	8.909930	-4.360073
64	1	0	2.467975	9.331355	-4.991706
65	1	0	2.868671	9.696049	-3.301000
66	6	0	-3.059497	7.629543	-6.389569
67	1	0	-2.371716	8.491630	-6.458858
68	1	0	-3.075273	7.147135	-7.380196
69	1	0	-4.065137	8.028182	-6.182488
70	6	0	-2.942760	-0.492196	2.635107
71	1	0	-3.414546	0.217148	3.332788
72	1	0	-3.747418	-1.112187	2.200769
73	1	0	-2.289144	-1.165840	3.212083
74	6	0	2.953190	-0.543521	0.000132
75	1	0	2.778260	-1.265579	-0.812212
76	1	0	3.802089	-0.912814	0.602037
77	1	0	2.063901	-0.524396	0.646187
78	6	0	2.192101	2.230029	1.399959
79	1	0	1.347295	1.524517	1.419862
80	1	0	2.843876	2.019577	2.265253

81	1	0	1.794133	3.249321	1.505495
82	6	0	3.592636	4.608810	-0.294105
83	1	0	2.749831	4.867611	0.362745
84	1	0	4.530815	4.872103	0.224176
85	1	0	3.515855	5.218328	-1.207511
86	6	0	5.147241	3.248365	-2.752734
87	1	0	4.884518	4.312002	-2.837236
88	1	0	6.196464	3.179981	-2.414972
89	1	0	5.082810	2.790490	-3.751849
90	6	0	4.589340	0.131640	-2.686171
91	1	0	4.561662	0.532305	-3.710354
92	1	0	5.638404	-0.100165	-2.433580
93	1	0	4.015691	-0.805011	-2.667014
94	1	0	-2.059667	2.314082	-4.334619
95	1	0	2.238433	2.720971	-5.255980
96	6	0	1.643258	-1.741245	-3.955102
97	1	0	1.820067	-1.444272	-2.907635
98	1	0	0.553499	-1.880633	-4.055340
99	1	0	2.140052	-2.703930	-4.142834
100	6	0	-2.791149	-1.834246	-2.257045
101	1	0	-3.638172	-2.474724	-1.970323
102	1	0	-2.136844	-2.394931	-2.945186
103	1	0	-2.192046	-1.610905	-1.357532

Structure 3’

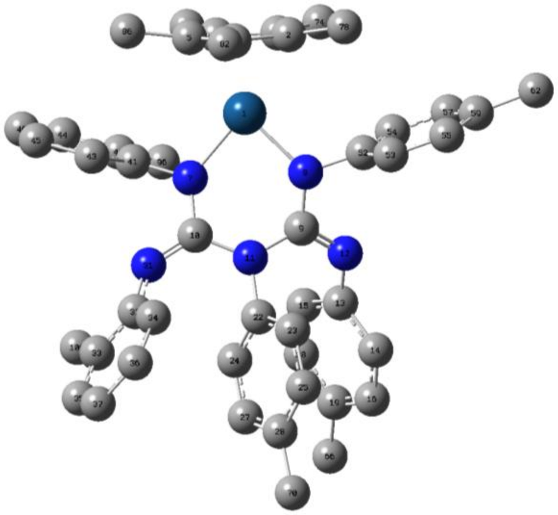

Zero-point correction= 0.826047 (Hartree/Particle)

Thermal correction to Energy= 0.879992

Thermal correction to Enthalpy= 0.880936

Thermal correction to Gibbs Free Energy= 0.733659

Sum of electronic and zero-point Energies= -2196.818998

Sum of electronic and thermal Energies= -2196.765053

Sum of electronic and thermal Enthalpies= -2196.764109

Sum of electronic and thermal Free Energies= -2196.911386

Center Number	Atomic Number	Atomic Type	Coordinates (Angstroms) X	Y	Z
1	77	0	1.933086	2.128485	-1.010403
2	6	0	3.375267	3.316654	0.176351
3	6	0	3.610440	3.470003	-1.248790
4	6	0	3.513790	1.921801	0.483550
5	6	0	4.020620	2.188768	-1.792696
6	6	0	3.905174	1.220948	-0.743238
7	7	0	0.873131	1.220407	-2.457674
8	7	0	0.179353	2.581305	-0.168496

9	6	0	-1.044584	2.008523	-0.467193
10	6	0	-0.452958	1.363226	-2.833628
11	7	0	-1.347704	1.803732	-1.845362
12	7	0	-1.905529	1.784361	0.483496
13	6	0	-2.973046	0.897151	0.345950
14	6	0	-4.285776	1.291319	0.677716
15	6	0	-2.756284	-0.449531	-0.024794
16	6	0	-5.344497	0.387251	0.578425
17	1	0	-4.463283	2.326752	0.977115
18	6	0	-3.822801	-1.348527	-0.102232
19	6	0	-5.139704	-0.946452	0.178491
20	1	0	-6.359144	0.721939	0.817006
21	1	0	-3.631610	-2.385727	-0.395781
22	6	0	-2.738296	2.005177	-2.179523
23	6	0	-3.296918	3.266302	-1.958094
24	6	0	-3.531783	0.963413	-2.670719
25	6	0	-4.654350	3.480520	-2.214884
26	1	0	-2.666499	4.069964	-1.569834
27	6	0	-4.881377	1.189617	-2.936502
28	6	0	-5.468173	2.447123	-2.707074
29	1	0	-5.089932	4.467678	-2.036443
30	1	0	-5.496585	0.370953	-3.320171
31	7	0	-0.795698	0.993474	-4.036392
32	6	0	-1.754432	1.464036	-4.900465
33	6	0	-2.400746	0.536791	-5.770002
34	6	0	-2.032546	2.845851	-5.055494
35	6	0	-3.302111	1.014055	-6.729345
36	6	0	-2.935841	3.295422	-6.020539
37	6	0	-3.583344	2.381948	-6.865763
38	1	0	-3.796518	0.288851	-7.384217

39	1	0	-3.135140	4.366992	-6.112919
40	1	0	-4.290629	2.728562	-7.622878
41	6	0	1.651390	0.509943	-3.432858
42	6	0	1.986826	-0.832109	-3.168377
43	6	0	2.045377	1.125177	-4.628262
44	6	0	2.771053	-1.517160	-4.113589
45	6	0	2.799380	0.420962	-5.569945
46	6	0	3.176133	-0.903568	-5.305714
47	1	0	3.049854	-2.555552	-3.910747
48	1	0	3.102508	0.906378	-6.500789
49	1	0	3.778085	-1.459661	-6.028481
50	1	0	-1.738010	-0.772864	-0.259943
51	1	0	-3.086344	-0.017197	-2.835098
52	6	0	0.207704	3.194956	1.129468
53	6	0	-0.056488	4.565277	1.232152
54	6	0	0.486868	2.457017	2.286199
55	6	0	-0.014741	5.195939	2.481518
56	1	0	-0.287460	5.129207	0.325098
57	6	0	0.531889	3.093168	3.529192
58	1	0	0.665773	1.384673	2.194451
59	6	0	0.288791	4.475475	3.648307
60	1	0	-0.214814	6.269091	2.552409
61	1	0	0.753292	2.508897	4.427490
62	6	0	0.373784	5.162094	4.989417
63	1	0	-0.172289	6.117664	4.982572
64	1	0	-0.040037	4.527857	5.789298
65	1	0	1.422943	5.379814	5.255831
66	6	0	-6.298683	-1.903066	0.035265
67	1	0	-6.802627	-1.773172	-0.939377
68	1	0	-5.962160	-2.949857	0.095267

69	1	0	-7.057118	-1.736658	0.817131
70	6	0	-6.938632	2.664618	-2.962086
71	1	0	-7.230892	2.286823	-3.955396
72	1	0	-7.548510	2.124402	-2.217037
73	1	0	-7.201007	3.731738	-2.904310
74	6	0	3.475856	1.286323	1.833213
75	1	0	2.943151	0.323622	1.803849
76	1	0	4.509601	1.088536	2.167805
77	1	0	2.989227	1.937688	2.569613
78	6	0	3.146169	4.430685	1.142121
79	1	0	2.615142	4.087476	2.040179
80	1	0	4.121062	4.849991	1.447294
81	1	0	2.554783	5.237354	0.687381
82	6	0	3.587227	4.760388	-1.999458
83	1	0	2.853214	5.454255	-1.565605
84	1	0	4.581974	5.238193	-1.951639
85	1	0	3.337972	4.593335	-3.057182
86	6	0	4.596442	1.954240	-3.145641
87	1	0	4.140471	2.607778	-3.901644
88	1	0	5.678912	2.173257	-3.112312
89	1	0	4.461087	0.913256	-3.467666
90	6	0	4.319105	-0.210594	-0.814434
91	1	0	4.159131	-0.621318	-1.820984
92	1	0	5.394247	-0.289716	-0.574200
93	1	0	3.764706	-0.822422	-0.088964
94	1	0	-1.528007	3.555254	-4.394391
95	1	0	1.744263	2.159899	-4.805080
96	6	0	1.471589	-1.512343	-1.927874
97	1	0	1.657029	-0.893889	-1.035600
98	1	0	0.378168	-1.651801	-1.992402

99	1	0	1.941125	-2.498312	-1.794342
100	6	0	-2.115834	-0.932959	-5.619174
101	1	0	-2.624800	-1.517908	-6.400588
102	1	0	-1.030589	-1.129680	-5.667289
103	1	0	-2.448939	-1.304965	-4.633235

Structure 3*'

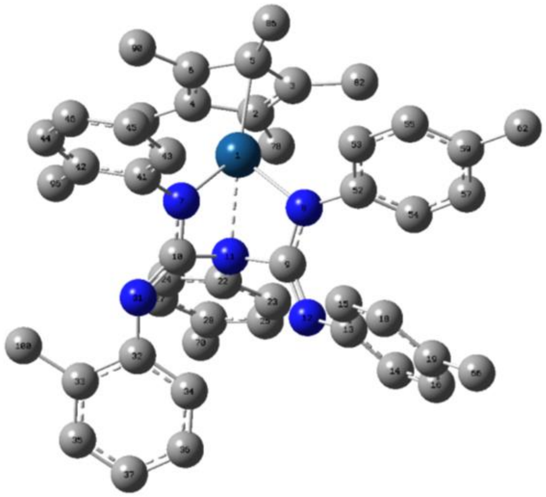

Zero-point correction= 0.826595 (Hartree/Particle)

Thermal correction to Energy= 0.880629

Thermal correction to Enthalpy= 0.881573

Thermal correction to Gibbs Free Energy= 0.734863

Sum of electronic and zero-point Energies= -2196.829189

Sum of electronic and thermal Energies= -2196.775154

Sum of electronic and thermal Enthalpies= -2196.774210

Sum of electronic and thermal Free Energies= -2196.920920

Center Number	Atomic Number	Atomic Type	Coordinates (Angstroms) X	Y	Z
1	77	0	2.028798	2.195785	-1.990044
2	6	0	2.765009	2.191754	0.043340

3	6	0	3.464432	3.200150	-0.704780
4	6	0	3.045745	0.884759	-0.560791
5	6	0	4.134089	2.534625	-1.822534
6	6	0	3.898782	1.101258	-1.695425
7	7	0	1.152705	1.219153	-3.610894
8	7	0	0.912948	3.812643	-2.635304
9	6	0	-0.360346	3.392222	-2.546331
10	6	0	-0.124439	1.029576	-3.225743
11	7	0	-0.236245	1.985704	-2.045573
12	7	0	-1.530324	3.876964	-2.811297
13	6	0	-1.709264	4.987870	-3.643162
14	6	0	-2.666289	5.962730	-3.301071
15	6	0	-1.021112	5.131293	-4.870855
16	6	0	-2.893214	7.062947	-4.135647
17	1	0	-3.217922	5.850798	-2.363978
18	6	0	-1.266866	6.224873	-5.700185
19	6	0	-2.197552	7.221154	-5.345659
20	1	0	-3.631768	7.815944	-3.843709
21	1	0	-0.720828	6.313965	-6.644644
22	6	0	-0.901739	1.570283	-0.852022
23	6	0	-1.448756	2.509132	0.032653
24	6	0	-0.894682	0.208791	-0.506133
25	6	0	-1.997920	2.078181	1.244907
26	1	0	-1.450209	3.566952	-0.232140
27	6	0	-1.452019	-0.202781	0.705236
28	6	0	-2.018086	0.720614	1.603637
29	1	0	-2.421984	2.818559	1.929109
30	1	0	-1.445301	-1.266319	0.960205
31	7	0	-1.006671	0.216879	-3.697106
32	6	0	-2.345512	0.093206	-3.312128

33	6	0	-2.917561	-1.211688	-3.331984
34	6	0	-3.168837	1.189900	-2.973040
35	6	0	-4.265925	-1.369407	-2.978968
36	6	0	-4.509255	1.004036	-2.622521
37	6	0	-5.068776	-0.279671	-2.617961
38	1	0	-4.690964	-2.378039	-2.993015
39	1	0	-5.118943	1.872907	-2.358046
40	1	0	-6.117174	-0.432108	-2.349872
41	6	0	1.806322	0.561240	-4.672078
42	6	0	2.022365	-0.836184	-4.648349
43	6	0	2.366319	1.349189	-5.693224
44	6	0	2.761578	-1.410044	-5.696181
45	6	0	3.104736	0.758941	-6.722601
46	6	0	3.296107	-0.630413	-6.728592
47	1	0	2.939230	-2.489561	-5.680983
48	1	0	3.528775	1.378957	-7.516115
49	1	0	3.873773	-1.103097	-7.526551
50	1	0	-0.287402	4.373388	-5.156901
51	1	0	-0.459998	-0.520002	-1.193027
52	6	0	1.341815	5.112492	-2.940928
53	6	0	2.450172	5.272718	-3.788689
54	6	0	0.742199	6.252362	-2.373959
55	6	0	2.937382	6.549297	-4.080639
56	1	0	2.916460	4.377017	-4.205174
57	6	0	1.235580	7.522738	-2.676970
58	1	0	-0.110263	6.132255	-1.703146
59	6	0	2.335607	7.698655	-3.537338
60	1	0	3.800412	6.658880	-4.744078
61	1	0	0.760502	8.401725	-2.231284
62	6	0	2.835299	9.079437	-3.883726

63	1	0	3.889386	9.055082	-4.200178
64	1	0	2.249943	9.513404	-4.713659
65	1	0	2.742301	9.764918	-3.026644
66	6	0	-2.429137	8.417358	-6.236600
67	1	0	-1.547077	9.081370	-6.249802
68	1	0	-2.617423	8.111421	-7.279126
69	1	0	-3.290333	9.009202	-5.890640
70	6	0	-2.647843	0.260848	2.894745
71	1	0	-3.679799	-0.093382	2.725038
72	1	0	-2.085125	-0.575856	3.337985
73	1	0	-2.694780	1.079611	3.628818
74	6	0	2.595478	-0.420498	0.009165
75	1	0	2.723480	-1.240993	-0.710369
76	1	0	3.188395	-0.659763	0.909062
77	1	0	1.535992	-0.374922	0.306572
78	6	0	1.914528	2.393633	1.255883
79	1	0	1.023939	1.746590	1.219023
80	1	0	2.485574	2.143102	2.166612
81	1	0	1.578135	3.437237	1.332974
82	6	0	3.515210	4.663416	-0.406362
83	1	0	2.623555	4.982913	0.152075
84	1	0	4.407205	4.894516	0.201190
85	1	0	3.564833	5.251254	-1.334613
86	6	0	5.068423	3.172286	-2.799252
87	1	0	4.820704	4.233041	-2.945738
88	1	0	6.106494	3.109052	-2.427638
89	1	0	5.023082	2.662487	-3.773540
90	6	0	4.484548	0.067363	-2.600070
91	1	0	4.401706	0.375517	-3.653420
92	1	0	5.553843	-0.068355	-2.363358

93	1	0	3.976327	-0.899255	-2.487715
94	1	0	-2.741297	2.196347	-2.979740
95	1	0	2.208204	2.430206	-5.657793
96	6	0	1.528816	-1.658518	-3.493764
97	1	0	1.649483	-1.095372	-2.553968
98	1	0	0.452331	-1.860362	-3.593270
99	1	0	2.072745	-2.612570	-3.422699
100	6	0	-2.090454	-2.414715	-3.699653
101	1	0	-2.732981	-3.290597	-3.877652
102	1	0	-1.485909	-2.218750	-4.600242
103	1	0	-1.381181	-2.674023	-2.893460

TS2’

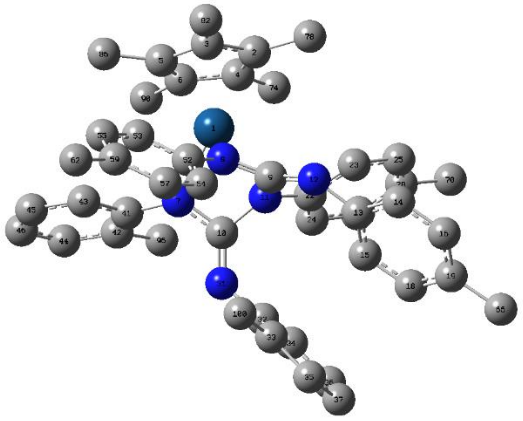

Zero-point correction= 0.824143 (Hartree/Particle)

Thermal correction to Energy= 0.878424

Thermal correction to Enthalpy= 0.879368

Thermal correction to Gibbs Free Energy= 0.733167

Sum of electronic and zero-point Energies= -2196.806787

Sum of electronic and thermal Energies= -2196.752507

Sum of electronic and thermal Enthalpies= -2196.751563

Sum of electronic and thermal Free Energies= -2196.897764

Center Atomic Atomic Coordinates (Angstroms)

Number	Number	Type	X	Y	Z
1	77	0	2.106356	2.386111	-1.688630
2	6	0	2.299468	2.904908	0.431462
3	6	0	3.428566	3.435635	-0.293932
4	6	0	2.306503	1.454370	0.252548
5	6	0	4.143501	2.339085	-0.919800
6	6	0	3.437341	1.109077	-0.577075
7	7	0	1.841837	1.278084	-3.421104
8	7	0	1.530081	4.038412	-2.909589
9	6	0	0.278407	4.229964	-2.682062
10	6	0	0.483429	1.238310	-3.476315
11	7	0	0.074506	2.028045	-2.340973
12	7	0	-0.653790	4.971738	-2.382747
13	6	0	-2.047742	4.973514	-2.470589
14	6	0	-2.720340	6.154280	-2.106758
15	6	0	-2.786789	3.849688	-2.881685
16	6	0	-4.115169	6.206678	-2.165425
17	1	0	-2.137848	7.021462	-1.788005
18	6	0	-4.178435	3.918818	-2.932038
19	6	0	-4.868981	5.092231	-2.574952
20	1	0	-4.631071	7.130538	-1.889713
21	1	0	-4.735335	3.041203	-3.271674
22	6	0	-0.863806	1.520969	-1.423267
23	6	0	-1.362585	2.378334	-0.420136
24	6	0	-1.266517	0.171061	-1.402165
25	6	0	-2.243235	1.903832	0.552542
26	1	0	-1.035495	3.417941	-0.404330
27	6	0	-2.154959	-0.289612	-0.425893
28	6	0	-2.666394	0.561438	0.567811

29	1	0	-2.611329	2.591216	1.320466
30	1	0	-2.449408	-1.343784	-0.429434
31	7	0	-0.219341	0.630107	-4.381115
32	6	0	-1.568359	0.833585	-4.660167
33	6	0	-1.976439	1.983152	-5.396220
34	6	0	-2.528036	-0.164067	-4.376425
35	6	0	-3.319796	2.106510	-5.780139
36	6	0	-3.863814	-0.015247	-4.760913
37	6	0	-4.270277	1.127556	-5.463597
38	1	0	-3.621274	2.998746	-6.337718
39	1	0	-4.586988	-0.797735	-4.515467
40	1	0	-5.311361	1.250242	-5.771782
41	6	0	2.742323	0.494976	-4.152421
42	6	0	2.657100	-0.924173	-4.164586
43	6	0	3.829948	1.132196	-4.783477
44	6	0	3.642631	-1.642252	-4.863243
45	6	0	4.804953	0.394663	-5.458914
46	6	0	4.707186	-1.003360	-5.510081
47	1	0	3.581122	-2.734957	-4.869833
48	1	0	5.634557	0.909945	-5.949836
49	1	0	5.462096	-1.592865	-6.035839
50	1	0	-2.267677	2.934364	-3.157058
51	1	0	-0.863712	-0.515048	-2.147806
52	6	0	2.279319	4.864162	-3.779702
53	6	0	3.659923	4.637545	-3.890155
54	6	0	1.691139	5.899644	-4.531645
55	6	0	4.439945	5.432378	-4.732992
56	1	0	4.100091	3.832639	-3.302021
57	6	0	2.485110	6.682937	-5.372577
58	1	0	0.617821	6.087903	-4.458153

59	6	0	3.870281	6.467987	-5.493858
60	1	0	5.515222	5.243246	-4.801419
61	1	0	2.015562	7.483508	-5.951399
62	6	0	4.707760	7.300716	-6.432104
63	1	0	5.757729	7.336832	-6.103319
64	1	0	4.695320	6.878395	-7.452408
65	1	0	4.325959	8.331318	-6.500313
66	6	0	-6.375541	5.140698	-2.611577
67	1	0	-6.804972	4.652055	-1.719438
68	1	0	-6.742147	6.178071	-2.632866
69	1	0	-6.768754	4.609474	-3.492745
70	6	0	-3.648204	0.063670	1.599678
71	1	0	-4.689058	0.263520	1.288473
72	1	0	-3.552545	-1.023138	1.748859
73	1	0	-3.497167	0.563684	2.569628
74	6	0	1.347024	0.498883	0.882600
75	1	0	1.288875	-0.437351	0.309656
76	1	0	1.678805	0.260314	1.908124
77	1	0	0.336406	0.929801	0.934621
78	6	0	1.362228	3.676008	1.303208
79	1	0	0.392224	3.163679	1.378349
80	1	0	1.776956	3.775811	2.321712
81	1	0	1.191638	4.686957	0.903493
82	6	0	3.806708	4.880575	-0.363884
83	1	0	2.914141	5.520894	-0.312281
84	1	0	4.463772	5.137882	0.484660
85	1	0	4.341220	5.107766	-1.296736
86	6	0	5.448000	2.396016	-1.650359
87	1	0	5.704416	3.426765	-1.935642
88	1	0	6.260373	2.015760	-1.006670

89	1	0	5.418469	1.775117	-2.560694
90	6	0	3.886807	-0.262055	-0.965120
91	1	0	4.212980	-0.283104	-2.015984
92	1	0	4.736503	-0.570302	-0.331934
93	1	0	3.078448	-0.995674	-0.841056
94	1	0	-2.204873	-1.061821	-3.845200
95	1	0	3.879793	2.221989	-4.742346
96	6	0	1.590356	-1.647173	-3.391653
97	1	0	1.422696	-1.151637	-2.420026
98	1	0	0.623130	-1.610514	-3.921113
99	1	0	1.875319	-2.696074	-3.219584
100	6	0	-0.965112	3.037665	-5.754776
101	1	0	-1.405647	3.802222	-6.411908
102	1	0	-0.576663	3.541836	-4.853397
103	1	0	-0.089726	2.587716	-6.253530

Structure 4’

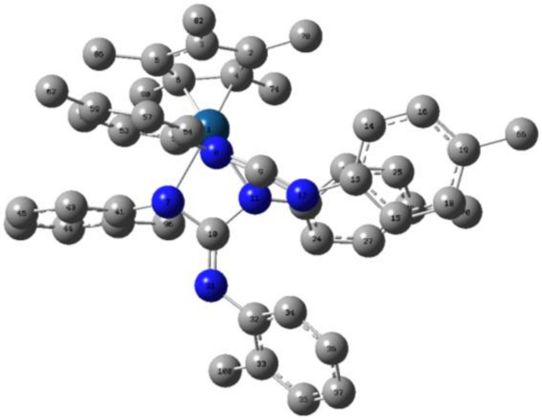

Zero-point correction= 0.824671 (Hartree/Particle)

Thermal correction to Energy= 0.879830

Thermal correction to Enthalpy= 0.880775

Thermal correction to Gibbs Free Energy= 0.729992

Sum of electronic and zero-point Energies= -2196.810541

Sum of electronic and thermal Energies= -2196.755381

Sum of electronic and thermal Enthalpies= -2196.754437

Sum of electronic and thermal Free Energies= -2196.905220

Center Number	Atomic Number	Atomic Type	Coordinates (Angstroms) X	Y	Z
1	77	0	2.109318	2.311763	-1.813511
2	6	0	2.818280	2.796980	0.211812
3	6	0	3.777425	3.293047	-0.756007
4	6	0	2.701935	1.365584	0.007312
5	6	0	4.281445	2.189262	-1.536229
6	6	0	3.608188	0.984756	-1.070335
7	7	0	1.481101	1.549495	-3.663598
8	7	0	1.457337	4.326006	-2.764900
9	6	0	0.341962	4.743316	-2.380746

10	6	0	0.140850	1.400238	-3.309177
11	7	0	0.082352	1.905396	-2.018600
12	7	0	-0.810580	4.983440	-2.078268
13	6	0	-1.425845	5.542084	-0.946802
14	6	0	-0.685642	6.190285	0.059380
15	6	0	-2.814637	5.399222	-0.815322
16	6	0	-1.340365	6.677481	1.190083
17	1	0	0.396188	6.299304	-0.052680
18	6	0	-3.454529	5.891578	0.324578
19	6	0	-2.733890	6.537209	1.344442
20	1	0	-0.761298	7.173945	1.973564
21	1	0	-4.534981	5.764469	0.431121
22	6	0	-0.847551	1.593399	-1.034388
23	6	0	-0.947574	2.446704	0.084758
24	6	0	-1.646798	0.427552	-1.049296
25	6	0	-1.833069	2.170948	1.127182
26	1	0	-0.311829	3.332068	0.115092
27	6	0	-2.538056	0.169220	-0.004513
28	6	0	-2.659451	1.032415	1.099978
29	1	0	-1.891345	2.858780	1.976909
30	1	0	-3.150895	-0.737275	-0.041106
31	7	0	-0.768840	0.919027	-4.112825
32	6	0	-2.131963	1.130373	-3.935551
33	6	0	-3.034149	0.072048	-4.241128
34	6	0	-2.665621	2.382711	-3.545280
35	6	0	-4.414562	0.298410	-4.142144
36	6	0	-4.044662	2.584762	-3.457461
37	6	0	-4.933738	1.541199	-3.753483
38	1	0	-5.095523	-0.526676	-4.375707
39	1	0	-4.430344	3.565939	-3.163207

40	1	0	-6.013788	1.692806	-3.687305
41	6	0	2.158944	0.600663	-4.450634
42	6	0	2.056664	-0.796255	-4.194261
43	6	0	3.031607	1.048280	-5.463586
44	6	0	2.813032	-1.681286	-4.979655
45	6	0	3.787288	0.149378	-6.222939
46	6	0	3.675030	-1.226759	-5.985619
47	1	0	2.736897	-2.753689	-4.773806
48	1	0	4.451697	0.523295	-7.006238
49	1	0	4.256932	-1.940636	-6.573608
50	1	0	-3.371448	4.874184	-1.593696
51	1	0	-1.557516	-0.272971	-1.880369
52	6	0	2.355909	5.106270	-3.552130
53	6	0	3.408478	4.450002	-4.198773
54	6	0	2.224135	6.499608	-3.670098
55	6	0	4.321414	5.181172	-4.962632
56	1	0	3.494550	3.370470	-4.084799
57	6	0	3.144386	7.217971	-4.437028
58	1	0	1.409185	7.016758	-3.156053
59	6	0	4.211154	6.576510	-5.093659
60	1	0	5.137415	4.656196	-5.467544
61	1	0	3.035780	8.302600	-4.523879
62	6	0	5.220007	7.367202	-5.887973
63	1	0	5.699542	6.741929	-6.656272
64	1	0	4.750236	8.232373	-6.380882
65	1	0	6.017903	7.756718	-5.231662
66	6	0	-3.432871	7.088392	2.560752
67	1	0	-3.645475	8.164383	2.434059
68	1	0	-4.392376	6.578513	2.734043
69	1	0	-2.808315	6.982798	3.461364

70	6	0	-3.655240	0.760662	2.201101
71	1	0	-4.625919	1.245452	1.991994
72	1	0	-3.846515	-0.318771	2.309333
73	1	0	-3.298517	1.149298	3.168258
74	6	0	1.859808	0.432425	0.814856
75	1	0	1.640322	-0.484809	0.249129
76	1	0	2.390986	0.151856	1.740725
77	1	0	0.904173	0.905865	1.086074
78	6	0	2.172393	3.593206	1.299644
79	1	0	1.244154	3.111796	1.639384
80	1	0	2.849949	3.678104	2.167694
81	1	0	1.931544	4.611500	0.959405
82	6	0	4.200485	4.721286	-0.869410
83	1	0	3.346647	5.397733	-0.715500
84	1	0	4.954290	4.943459	-0.094221
85	1	0	4.637719	4.934727	-1.853863
86	6	0	5.362588	2.214481	-2.569567
87	1	0	5.547593	3.236399	-2.928890
88	1	0	6.304236	1.822283	-2.147415
89	1	0	5.091052	1.586388	-3.434169
90	6	0	3.959495	-0.398504	-1.505356
91	1	0	4.024970	-0.464155	-2.601847
92	1	0	4.942597	-0.671382	-1.083465
93	1	0	3.218501	-1.130832	-1.159270
94	1	0	-1.974044	3.192609	-3.303025
95	1	0	3.083301	2.119509	-5.666655
96	6	0	1.196770	-1.319359	-3.076169
97	1	0	1.240606	-0.642928	-2.205710
98	1	0	0.139300	-1.361847	-3.384087
99	1	0	1.523259	-2.325166	-2.770684

100	6	0	-2.494680	-1.278984	-4.628668
101	1	0	-3.300399	-1.941738	-4.980216
102	1	0	-1.726404	-1.185993	-5.414288
103	1	0	-1.997475	-1.769530	-3.771775

Structure 5'

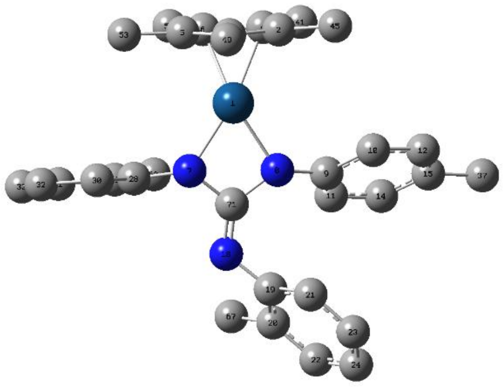

Zero-point correction= 0.579539 (Hartree/Particle)

Thermal correction to Energy= 0.617628

Thermal correction to Enthalpy= 0.618572

Thermal correction to Gibbs Free Energy= 0.507509

Sum of electronic and zero-point Energies= -1508.197411

Sum of electronic and thermal Energies= -1508.159322

Sum of electronic and thermal Enthalpies= -1508.158378

Sum of electronic and thermal Free Energies= -1508.269440

Center Number	Atomic Number	Atomic Type	Coordinates (Angstroms) X	Y	Z
1	77	0	2.110009	1.867687	-1.635476
2	6	0	2.848800	2.750953	0.242009
3	6	0	3.725021	3.099139	-0.869279
4	6	0	2.804253	1.317375	0.321166
5	6	0	4.292712	1.889156	-1.424429

6	6	0	3.696456	0.779065	-0.720983
7	7	0	1.536463	1.373718	-3.479752
8	7	0	0.106074	1.838004	-1.912174
9	6	0	-0.898970	1.583375	-0.970725
10	6	0	-1.077163	2.501458	0.081508
11	6	0	-1.684706	0.412093	-0.979483
12	6	0	-2.027602	2.268696	1.079094
13	1	0	-0.455683	3.399474	0.094721
14	6	0	-2.642179	0.198516	0.014966
15	6	0	-2.838119	1.119333	1.060712
16	1	0	-2.149423	2.996793	1.887033
17	1	0	-3.245637	-0.714142	-0.012600
18	7	0	-0.729196	0.934252	-4.066570
19	6	0	-2.093145	1.173330	-3.871275
20	6	0	-3.013943	0.124704	-4.131828
21	6	0	-2.580737	2.447092	-3.503952
22	6	0	-4.385834	0.380997	-3.994858
23	6	0	-3.951735	2.679767	-3.375996
24	6	0	-4.864202	1.643103	-3.617912
25	1	0	-5.090968	-0.433538	-4.187651
26	1	0	-4.308293	3.671909	-3.086939
27	1	0	-5.938438	1.816655	-3.520067
28	6	0	2.211357	0.673515	-4.499045
29	6	0	2.171267	-0.742998	-4.544268
30	6	0	3.024205	1.386854	-5.395397
31	6	0	2.929851	-1.396057	-5.529263
32	6	0	3.774326	0.715830	-6.366774
33	6	0	3.721631	-0.682552	-6.438730
34	1	0	2.907462	-2.489408	-5.568130
35	1	0	4.397462	1.281869	-7.063487

36	1	0	4.304127	-1.218067	-7.192338
37	6	0	-3.897182	0.891206	2.111409
38	1	0	-4.876145	1.282208	1.781260
39	1	0	-4.029017	-0.182480	2.319502
40	1	0	-3.641152	1.400138	3.053830
41	6	0	2.021497	0.514161	1.309483
42	1	0	1.800480	-0.487053	0.911484
43	1	0	2.589593	0.394070	2.248249
44	1	0	1.066597	1.011156	1.539674
45	6	0	2.153650	3.713720	1.148920
46	1	0	1.250617	3.260153	1.582356
47	1	0	2.819750	4.011153	1.977651
48	1	0	1.858801	4.625643	0.608117
49	6	0	4.037040	4.491042	-1.310997
50	1	0	3.177618	5.156773	-1.145738
51	1	0	4.891377	4.885539	-0.732882
52	1	0	4.303649	4.516755	-2.377128
53	6	0	5.273230	1.793045	-2.549811
54	1	0	5.218167	2.682440	-3.194219
55	1	0	6.304109	1.709449	-2.164914
56	1	0	5.063378	0.912245	-3.175256
57	6	0	3.991071	-0.665260	-0.962920
58	1	0	4.192584	-0.848042	-2.029202
59	1	0	4.879586	-0.973127	-0.384296
60	1	0	3.145098	-1.296354	-0.654922
61	1	0	-1.858841	3.244963	-3.313210
62	1	0	3.053723	2.475907	-5.310850
63	6	0	1.369579	-1.513119	-3.531739
64	1	0	1.517984	-1.083752	-2.525008
65	1	0	0.291142	-1.437999	-3.748628

66	1	0	1.659756	-2.574260	-3.522595
67	6	0	-2.506556	-1.244056	-4.498627
68	1	0	-3.329768	-1.897138	-4.825404
69	1	0	-1.748655	-1.181224	-5.297226
70	1	0	-2.007804	-1.726706	-3.638413
71	6	0	0.167108	1.330491	-3.223110
72	1	0	-1.533387	-0.328921	-1.766491
