# Supplementary material for: Correction to “Carbodiimide Ring-Opening Metathesis Polymerization”
Source: ACS Cent Sci. 2023 Aug 3;9(8):1703–4. doi: 10.1021/acscentsci.3c00873 (PMC10450882; doi:10.1021/acscentsci.3c00873)
Supplement: Supplementary file 1 — oc3c00873_si_001.pdf [file oc3c00873_si_001.pdf]

Correction to Supporting Information for "Carbodiimide Ring-opening Metathesis Polymerization"

J. Drake Johnson,[1] Samuel W. Kaplan,[1] Jozsef Toth,[1] Zian Wang,[1] Mitchell Maw,[1] Sergei S. Sheiko,[1] Aleksandr V. Zhukhovitskiy[1]*

Affiliations:

[1] Department of Chemistry, University of North Carolina at Chapel Hill; Chapel Hill, NC 27599, USA.

*Corresponding author. Email: alexzhuk@email.unc.edu

The revised Figures S2 and S12, as well as their captions, and the revised caption for Figure S15 are provided below.

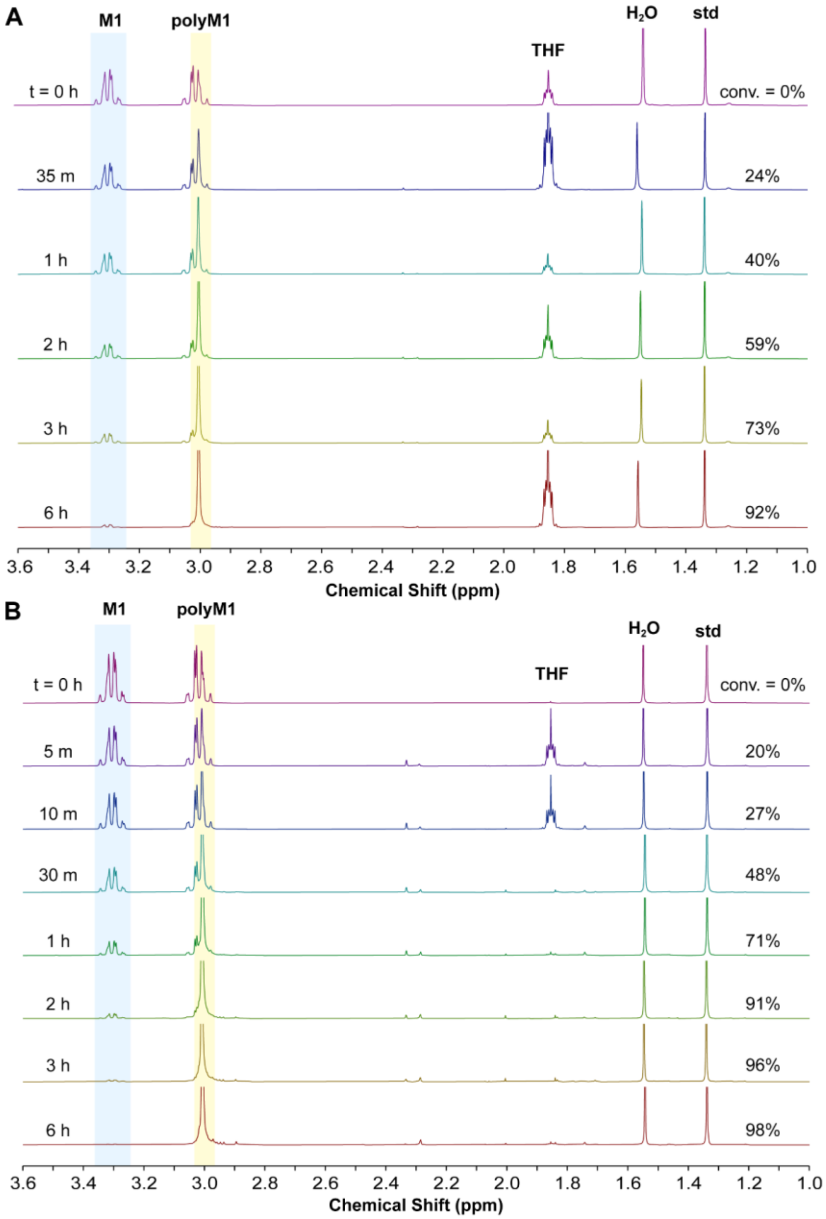

Figure S2. A. ^{1}H NMR (500 MHz, $CDCl_3$, 23 °C) spectra of a 200:1 [**M1**]:[**1**] polymerization at various timepoints (indicated on the left) with conversion of **M1** indicated on the right. B. ^{1}H NMR (500 MHz, $CDCl_3$, 23 °C) spectra of a 100:1 [**M1**]:[**1**] polymerization at various timepoints (indicated on the left) with conversion of **M1** indicated on the right. Residual H_2O originates from the NMR solvent bottle.

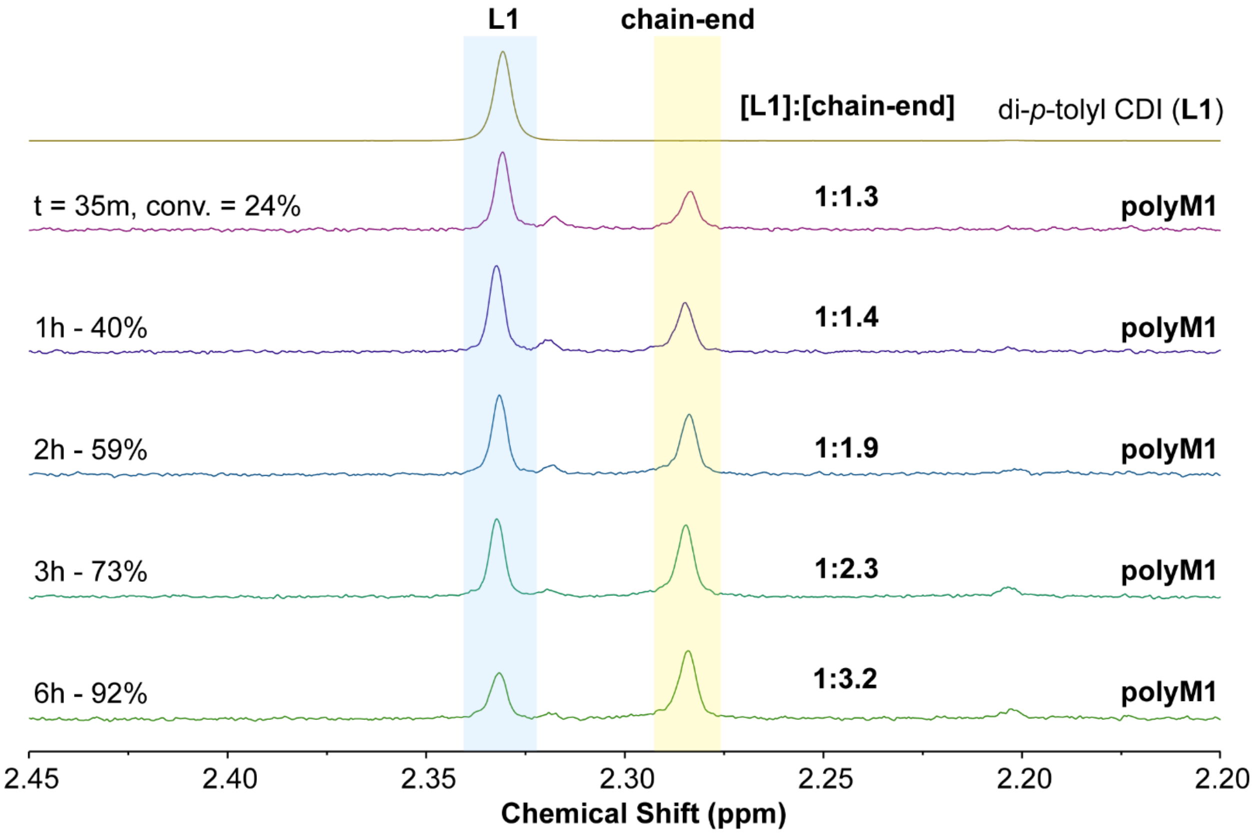

Figure S12. Stacked ^{1}H NMR (500 MHz, $CDCl_3$, 23 °C) spectra comparison of crude polymerization mixtures of a 200:1 [**M1**]:[**1**] experiment (at various times and conversions, listed left) and di-*p*-tolyl CDI **L1** (the chain-transfer agent). Molar ratios between **L1** and the chain-end at each time point are listed in the center.

Figure S15. Stacked ^{1}H NMR (500 MHz, $CDCl_3$, 23 °C) spectra comparison of crude polymerization mixtures of a 200:1 [**M2**]:[**1**] experiment (at various times and conversions, listed left), chain-end model compound N-*p*-tolyl-N'-hexylcarbodiimide **L3**, and di-*p*-tolyl carbodiimide **L1** (the chain-transfer agent). Molar ratios between **L1** and the chain-end at each time point are listed center.
